# Atmospheric Chemistry of 2-Amino-2-methyl-1-propanol: A Theoretical and Experimental Study of the OH-Initiated Degradation under Simulated Atmospheric Conditions

**DOI:** 10.1021/acs.jpca.1c04898

**Published:** 2021-08-23

**Authors:** Wen Tan, Liang Zhu, Tomáš Mikoviny, Claus J. Nielsen, Yizhen Tang, Armin Wisthaler, Philipp Eichler, Markus Müller, Barbara D’Anna, Naomi J. Farren, Jacqueline F. Hamilton, Jan B. C. Pettersson, Mattias Hallquist, Simen Antonsen, Yngve Stenstrøm

**Affiliations:** † Section for Environmental Sciences, Department of Chemistry, University of Oslo, P.O. Box 1033, Blindern, NO-0315 Oslo, Norway; ‡ Institute for Ion Physics and Applied Physics, University of Innsbruck, 6020 Innsbruck, Austria; § Aix Marseille Université, CNRS, LCE, UMR 7376, 13331 Marseille, France; ∥ Wolfson Atmospheric Chemistry Laboratories, Department of Chemistry, University of York, York YO10 5DD, U.K.; ⊥ Atmospheric Science, Department of Chemistry and Molecular Biology, University of Gothenburg, 41296 Gothenburg, Sweden; # Faculty of Chemistry, Biotechnology and Food Science, Norwegian University of Life Sciences, P.O. Box 5003, N-1432 Ås, Norway

## Abstract

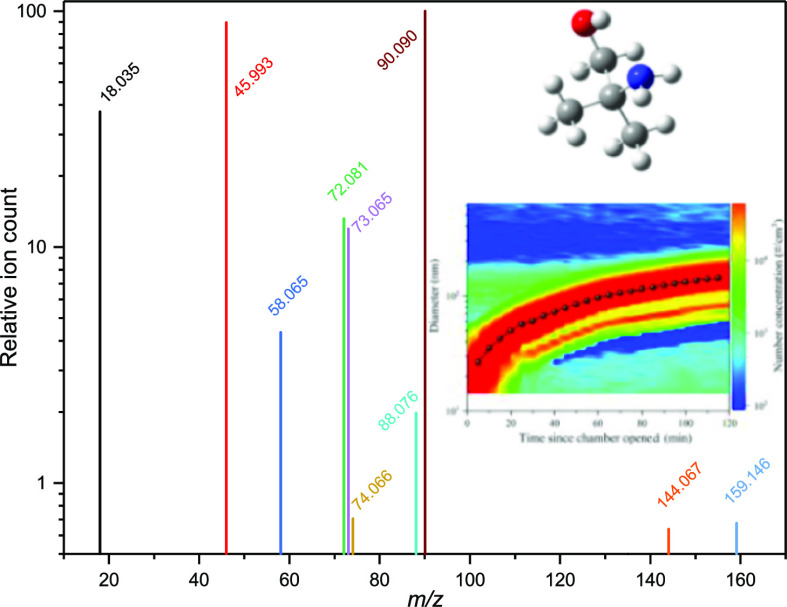

The OH-initiated degradation of 2-amino-2-methyl-1-propanol [CH_3_C(NH_2_)(CH_3_)CH_2_OH, AMP] was investigated in a large atmospheric simulation chamber, employing time-resolved online high-resolution proton-transfer reaction-time-of-flight mass spectrometry (PTR-ToF-MS) and chemical analysis of aerosol online PTR-ToF-MS (CHARON-PTR-ToF-MS) instrumentation, and by theoretical calculations based on M06-2X/aug-cc-pVTZ quantum chemistry results and master equation modeling of the pivotal reaction steps. The quantum chemistry calculations reproduce the experimental rate coefficient of the AMP + OH reaction, aligning *k*(*T*) = 5.2 × 10^–12^ × exp (505/*T*) cm^3^ molecule^–1^ s^–1^ to the experimental value *k*_exp,300K_ = 2.8 × 10^–11^ cm^3^ molecule^–1^ s^–1^. The theoretical calculations predict that the AMP + OH reaction proceeds via hydrogen abstraction from the −CH_3_ groups (5–10%), −CH_2_– group, (>70%) and −NH_2_ group (5–20%), whereas hydrogen abstraction from the −OH group can be disregarded under atmospheric conditions. A detailed mechanism for atmospheric AMP degradation was obtained as part of the theoretical study. The photo-oxidation experiments show 2-amino-2-methylpropanal [CH_3_C(NH_2_)(CH_3_)CHO] as the major gas-phase product and propan-2-imine [(CH_3_)_2_C=NH], 2-iminopropanol [(CH_3_)(CH_2_OH)C=NH], acetamide [CH_3_C(O)NH_2_], formaldehyde (CH_2_O), and nitramine 2-methyl-2-(nitroamino)-1-propanol [AMPNO_2_, CH_3_C(CH_3_)(NHNO_2_)CH_2_OH] as minor primary products; there is no experimental evidence of nitrosamine formation. The branching in the initial H abstraction by OH radicals was derived in analyses of the temporal gas-phase product profiles to be *B*_CH_3__/*B*_CH_2__/*B*_NH_2__ = 6:70:24. Secondary photo-oxidation products and products resulting from particle and surface processing of the primary gas-phase products were also observed and quantified. All the photo-oxidation experiments were accompanied by extensive particle formation that was initiated by the reaction of AMP with nitric acid and that mainly consisted of this salt. Minor amounts of the gas-phase photo-oxidation products, including AMPNO_2_, were detected in the particles by CHARON-PTR-ToF-MS and GC×GC-NCD. Volatility measurements of laboratory-generated AMP nitrate nanoparticles gave Δ_vap_*H* = 80 ± 16 kJ mol^–1^ and an estimated vapor pressure of (1.3 ± 0.3) × 10^–5^ Pa at 298 K. The atmospheric chemistry of AMP is evaluated and a validated chemistry model for implementation in dispersion models is presented.

## 1 Introduction

2-Amino-2-methyl-1-propanol (AMP), CH_3_C(NH_2_)(CH_3_)CH_2_OH, is a common ingredient in many consumer products.^1^ AMP is also one of the promising amines considered for usage in the industrial scale post-combustion CO_2_ capture technology due to its excellent absorption and desorption capacity, high loading capacity, and low replenishment cost.^2−4^ A 40 wt % amine solution with piperazine and AMP in a 1:2 molar ratio has been suggested as the new benchmark solvent for the CO_2_ capture technology, showing a cost reduction of 22% for coal-fired and 15% for gas-fired power plants compared to a 30 wt % ethanolamine-based (MEA) system.^5^

Small amounts of solvent amines invariably escape to the atmosphere during the operation of a large-scale CO_2_ capture facility employing the amine technology. Once in the atmosphere, the amines undergo oxidative degradation, resulting in the formation of imines, amides, and potential carcinogens such as nitrosamines and nitramines.^6^ The Norwegian Institute for Public Health (NIPH) has recommended that the total amount of nitrosamines and nitramines in the atmosphere should be below 0.3 ng m^–3^ in air and below 40 ng dm^3^ in drinking water so not to exceed a cancer risk level of 10^–5^.^7^ Such low levels are extremely difficult to monitor, and it is consequently important to obtain quantitative information on the degradation pathways for the relevant amines under atmospheric conditions and to implement this information in reliable chemistry models for dispersion calculations. Another important consideration is the contribution of amines to the formation of new particles.^8,9^

The rate coefficient for the AMP reaction with OH radicals was reported to be (2.8 ± 0.5) × 10^–11^ cm^3^ molecule^–1^ s^–1^ at 300 ± 2 K, corresponding to an atmospheric lifetime around 10 h.^10^ Environmental chamber experiments with AMP were initially carried out as so-called “incremental reactivity” experiments to assess the ground-level atmospheric ozone impacts of consumer products.^11^ In these experiments, AMP was added to a standard reactive organic gas surrogate—NO_*x*_ mixture, simulating the chemical conditions of polluted urban atmospheres. AMP was characterized as very “sticky” and a “strong inhibitor of gas-phase reactions” causing a “significant slowing of O_3_ formation, NO oxidation, and integrated OH radical levels”.^11^ The experiments mentioned were severely hampered by wall loss and particle formation preventing amine quantification, and only a very simplified mechanism, having 80% H abstraction from the −NH_2_ group and including both nitrosamine and nitramine formation, was added to the SAPRC-07 mechanism.^12,13^ A more detailed mechanism for AMP degradation was outlined from the first principles by Bråten et al.^14^ as part of the Norwegian “CO_2_ and amines screening study for environmental risks”.^15^ Focusing on possible carcinogen formation, preliminary results from the studies of AMP suggested a nitramine yield of (0.4 ± 0.2) % of the reacted AMP per ppbV NO_2_ present in the air.^16^ A recent series of the photo-oxidation experiments with AMP and surrogate hydrocarbon mixtures was carried out in a CSIRO 24.7 m^3^ indoor smog chamber, and a more elaborate mechanism improving their prediction against AMP-VOC-NO_*x*_ experiments was presented.^17^ Also in these experiments, large amounts of AMP-derived secondary aerosols were observed with a reported mass yield of 1.06 ± 0.20.

We have recently presented results from theoretical calculations and experimental photo-oxidation studies of piperazine^18^ —the other component of the suggested new benchmark solvent for the CO_2_ capture technology—and previously reported results from theoretical and experimental photo-oxidation studies of the AMP related, simpler compound, *tert*-butylamine, (CH_3_)_3_C(NH_2_).^19^ The present communication summarizes our results of detailed theoretical calculations of AMP degradation under atmospheric conditions and of photo-oxidation experiments carried out under simulated atmospheric conditions in a 200 m^3^ European Photoreactor (EUPHORE) in Spain. The results allow the first reliable environmental impact assessment of implementing large-scale carbon capture facilities employing AMP-containing solvents.

## 2 Methods

### 2.1 Experimental Methods and Chemicals

A series of experiments were carried out in chamber B of the EUPHORE facility in Valencia, Spain (39°28′12″N, 00°22′35″W). The local time during the experiments was UTC + 2:00. The facility and analytical methods have previously been reported in detail;^20^ special online instrumentation employed in the present experiments includes a high-resolution proton transfer reaction time-of-flight (PTR-TOF) 8000 instrument (*m*/Δ*m* > 3000) from Ionicon Analytik GmbH, a prototype chemical analysis of aerosol online (CHARON) inlet^21,22^ interfaced to a second PTR-TOF 8000 instrument (*m*/Δ*m* > 3000) and a compact TOF aerosol mass spectrometry (C-ToF-AMS) instrument from Aerodyne Research Inc.^23^ Additional information specific to the present work is found in the Supporting Information.

AMP (Sigma-Aldrich, ReagentPlus, ≥99%), ammonium nitrate (Sigma-Aldrich), and 2-methylpropane-1,2-diol (Apollo Scientific Ltd, 99.97%) were used as received. 2-Propyl nitrite (isopropyl nitrite, IPN) was synthesized from isopropanol, hydrochloric acid, and sodium nitrite and purified by repeated washing with ice water. The AMP nitrate salt was prepared by adding an excess of diluted nitric acid (HNO_3_) to diluted AMP followed by rotary evaporation to dryness at 80 °C. 2-Methyl-2-(nitroamino)-1-propanol (AMPNO_2_) was prepared as described by Antonsen et al.,^24^ see the Supporting Information for details.

### 2.2 Computational Methods

Optimized geometries of stationary points on the potential energy surface of the OH reaction with AMP were obtained with the M06-2X hybrid meta-exchange–correlation density functional,^25^ employing the aug-cc-pVTZ basis sets,^26,27^ tight optimization criteria, and ultrafine integration grids. Pre- and postreaction complexes were located by following the reaction path (IRC) from the saddle points. Electronic energies of selected stationary points were improved by explicitly correlated coupled cluster calculations with scaled triples contributions, denoted CCSD(T*)-F12a.^28^ Reaction enthalpies and proton affinities were calculated using the G4 model chemistry.^29^ Dipole moments and isotropic polarizabilities serving as inputs to the prediction of ion–molecule reaction rate coefficients^30^ were obtained in B3LYP^31−34^ and M06-2X calculations. The DFT and G4 calculations were done with Gaussian 09^35^ and Gaussian 16,^36^ CCSD(T*)-F12a calculations were performed employing Molpro 2019.2.^37^

Master equation calculations were carried out using MESMER 3.0^38^ to simulate the reactions under atmospheric conditions. The required input parameters for molecules, intermediate species and products were obtained from the ab initio calculations.

## 3 Results and Discussion

We first report results from a theoretical study of the OH-initiated photo-oxidation of AMP under atmospheric conditions, facilitating the presentation and interpretation of the experimental data. We then show results from gas-phase photo-oxidation experiments, before addressing the results for the particle phase, and finally attending to modeling of the chamber experiments.

### 3.1 Computational Results

AMP exists in several conformations; the lowest energy conformer has the OH and NH_2_ groups in a gauche configuration with an intramolecular H bonding from the OH group to the NH_2_ group. There are two additional AMP conformers within 10 kJ mol^–1^, in which the NH_2_ group is the proton donor, but these conformers only populate a few percent under atmospheric conditions, and they will not be considered here.

#### 3.1.1 Kinetics of and Branching in the AMP + OH Reaction

There are four avenues in the AMP + OH reaction; in decreasing order of reaction exothermicity (in units of kJ mol^–1^ at 298 K), they areΔ*H*^⊖^ = −105:
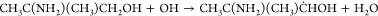
1aΔ*H*^⊖^ = −78:
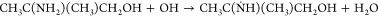
1bΔ*H*^⊖^ = −71:
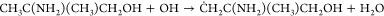
1cΔ*H*^⊖^ = −56:
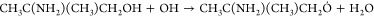
1d

**Figure 1:**
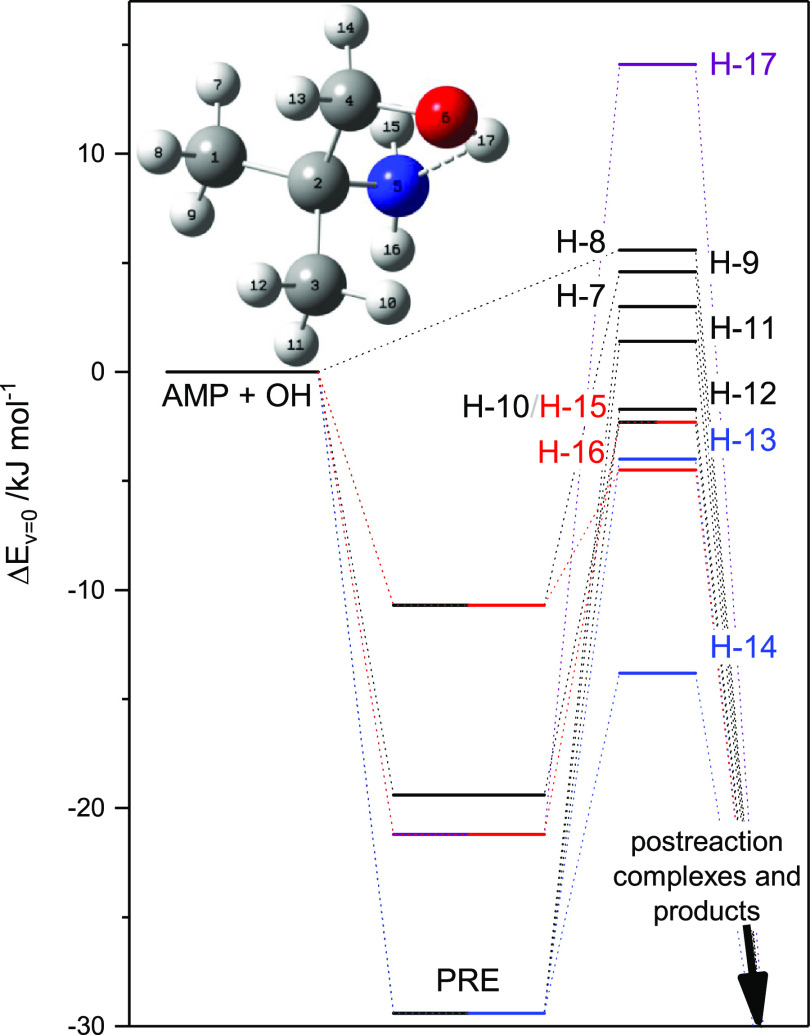
Relative energies of stationary points on the potential energy surface of the AMP + OH reaction. Results from M06-2X/aug-cc-pVTZ calculations. The CH_3_-abstraction routes are outlined in black color, the CH_2_-abstraction routes in blue, the NH_2_-abstraction routes in red, and the OH-abstraction route in purple color. For clarity, the stationary points of postreaction complexes and products are not included in the figure. Additional depictions of the stationary points on the potential energy surface of the AMP + OH reaction are given in Figures S5–S7.

AMP has 11 non-equivalent H-atoms, and a thorough theoretical description of the AMP + OH reaction kinetics is consequently far from trivial. Figure 1 illustrates the relative energies of the stationary points on the entrance side of the potential energy surfaces (PESes) of the four routes—detailed figures, electronic energies, Cartesian coordinates, and vibration-rotation data for all stationary points on the PESes of reactions 1a, 1b, and 1c are collected in Figures S5–S7 and Table S2. The reaction is characterized by pre- and postreaction complexes and several saddle points to the reaction below the entrance energy of the reactants. The barrier to abstraction from the −OH group is calculated to be around 10 kJ mol^–1^, and this route will consequently be of little importance under atmospheric conditions.

The kinetics of reactions 1a, 1b, and 1c was simulated in a master equation model based on the PES illustrated in part in Figure 1 (all vibrational modes were treated as harmonic oscillators). Spin–orbit coupling in the OH radical (139.7 cm^–1^)^39^ was included in the model by lowering the energy of the OH radical with half of the splitting and including the ^2^Π_3/2_ and ^2^Π_1/2_ spin–orbit states in the electronic partition function; it was assumed that the spin–orbit coupling could be neglected in prereaction adducts and in the saddle points. The formation of prereaction complexes and dissociation of postreaction complexes were treated as reversible reactions with rate coefficients approximated by typical values of *k*_association_ = 4 × 10^–10^ × (*T*/298 K)^−1/6^ cm^3^ molecule^–1^ s^–1^ from the long-range transition state theory (LRTST).^40^ Tunneling was included using a one dimensional asymmetric Eckart potential.^41^ The calculations predict an overall rate coefficient *k*_AMP+OH_ = 3.6 × 10^–11^ cm^3^ molecule^–1^ s^–1^ at 298 K, which, by utter fortuity, is close to the experimental value of (2.8 ± 0.5) × 10^–11^ cm^3^ molecule^–1^ s^–1^ at 300 K.^10^ The branching between H abstraction from the −CH_3_ groups (*B*_CH_3__), the −CH_2_– group (*B*_CH_2__), and −NH_2_ group (*B*_NH_2__) is predicted to be 5:90: 5 at 298 K; H abstraction from the −OH group contributes less than 0.1% to the total reactivity and is clearly of little importance under atmospheric conditions. The LRTST value for *k*_association_ is an upper case value, and reducing *k*_association_ by a factor of 4 in the model changes the branching to 7:86:7 and the predicted rate coefficient to 2.7 × 10^–11^ cm^3^ molecule^–1^ s^–1^ at 298 K. The calculated overall rate coefficient has virtually no pressure dependence in the 1–1000 mbar region and shows a negative temperature dependency. The theoretical results can be reasonably well described by the Arrhenius equation in the region 200–400 K, and aligning the theoretical results to the experimental rate coefficient at 300 K results in *k*(*T*) = 5.2 × 10^–12^ × exp (505/*T*) cm^3^ molecule^–1^ s^–1^.

The OH reaction with the related compound, *tert*-butylamine (*t*BA), was previously examined in both M062X and MP2 calculations.^19^ In addition, improved single point energies were obtained in the CCSD(T*)-F12a calculations. In general, the results of the *t*BA + OH reaction obtained in M062X, CCSD(T*)-F12a//M062X, and CCSD(T*)-F12a//MP2 agreed within 2 kJ mol^–1^ when the aug-cc-pVTZ basis set was employed. The exception being the energy of the saddle point to N–H abstraction, which was calculated to be 4 kJ mol^–1^ lower at the CCSD(T*)-F12a//MP2 level.

The sensitivity of the calculated rate coefficient and the branching to variations in the saddle point energies was examined by varying all barrier heights by ±2 kJ mol^–1^. The results show that changing all barriers ±2 kJ mol^–1^ results in a ∓45% change in the calculated rate coefficient at 298 K. At the same time, the branching changed from 5.5:89.5:5.0 to, respectively, 7.6:85.0:7.4 and 4.6:91.2:4.2. Changing only the barriers to H abstraction from the −NH_2_ group by ±4 kJ mol^–1^ alters the rate coefficient by, respectively, −4 and +16%, and the branching correspondingly to 5.6:93.4:1.0 and 4.7:77.3:18.0. The theoretical calculations consequently place conservative upper limits of ∼10% to abstraction from the −CH_3_ groups and ∼20% to abstraction from the −NH_2_ group. The present result for the branching in the AMP + OH reaction therefore differs radically from that currently employed in air quality models, which both adopt 80% abstraction from the −NH_2_ group.^11,17^

#### 3.1.2 Primary Photo-Oxidation Products

**Scheme 1:**
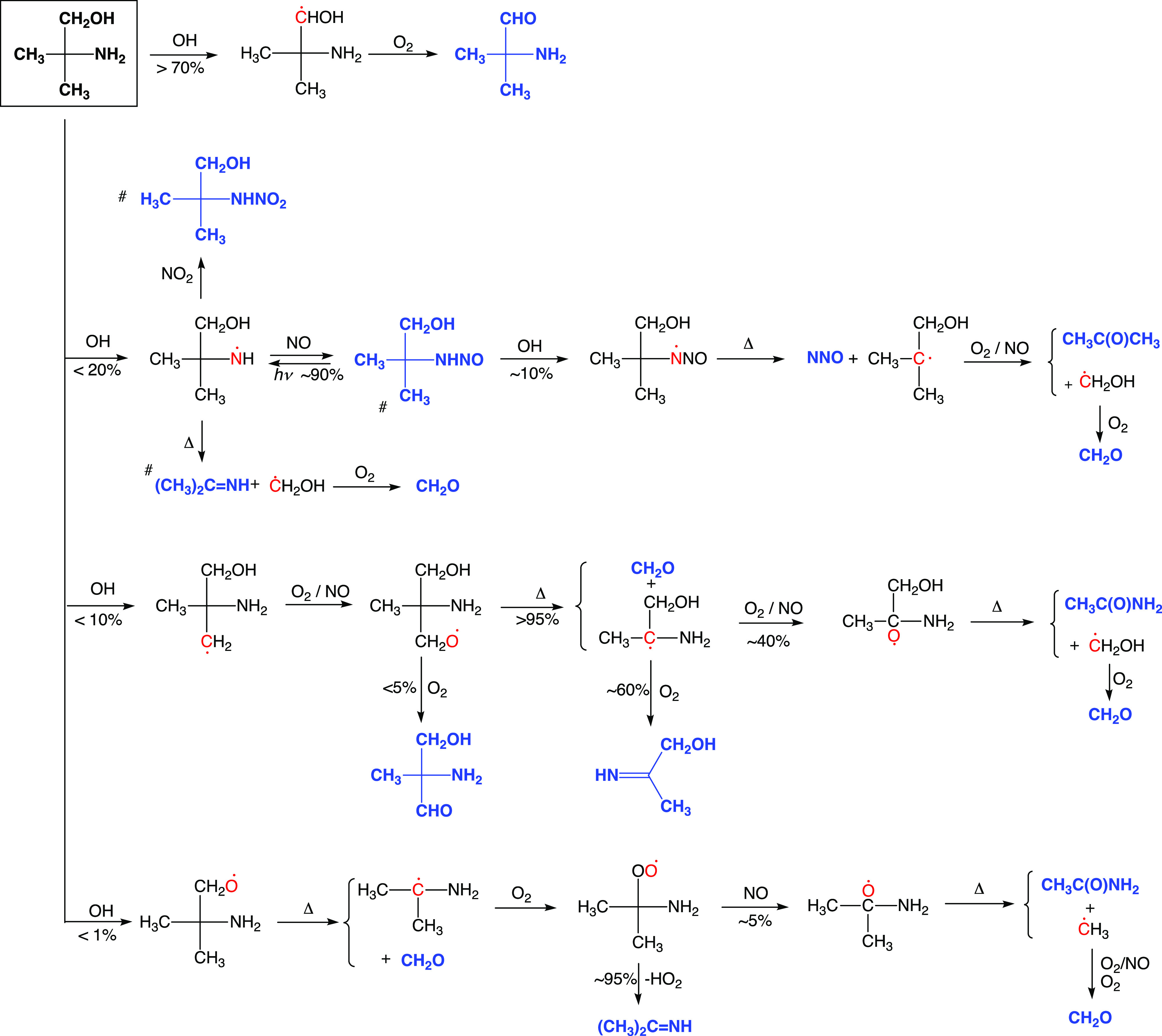
Major Reaction Routes for the OH-Initiated Photo-oxidation of AMP under Atmospheric Conditions Thermally stable products are typeset in bold blue font. Radical sites are indicated in red font. ^#^The branching in the (CH_3_)_2_(CH_2_OH)CṄH radical depends upon the NO and NO_2_ mixing ratios.

A detailed account of our theoretical study of the atmospheric fate of the CH_3_C(ṄH)(CH_3_)CH_2_OH, CH_3_C(NH_2_)(CH_3_)ĊHOH, ĊH_2_C(NH_2_)(CH_3_)CH_2_OH, and CH_3_C(NH_2_)(CH_3_)CH_2_Ȯ radicals is found in the Supporting Information, which includes figures of pivotal reaction steps (Figures S8–S16) and associated tables containing electronic energies, Cartesian coordinates, and vibration-rotation data (Tables S3–S12). The theoretically predicted atmospheric degradation routes are outlined in Scheme 1, from which it can be seen that there are characteristic primary products to each route and that CH_3_C(NH_2_)(CH_3_)CHO is predicted to be the major product in AMP photo-oxidation under atmospheric conditions.

Focusing on nitrosamine and nitramine formation in the atmospheric photo-oxidation of AMP, the theoretical calculations (CCSD(T*)-F12a/aug-cc-pVTZ//M06-2X/aug-cc-pVTZ, Table S3) place the aminyl radical, CH_3_C(ṄH)(CH_3_)(CH_2_OH), with a relatively low barrier to dissociation of 83.7 kJ mol^–1^Δ*H*^⊖^ = +68:
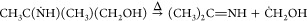
2

RRKM calculations predict reaction 2 with a thermal rate coefficient of 2.3 × 10^–2^ s^–1^ at 298 K, which is comparable to the estimated rates of the competing reactions with NO and NO_2_ under atmospheric conditions.^42,43^ We note that a change in the barrier height by ±4 kJ mol^–1^ results in the change in the calculated rate coefficient by a factor of 5.

The theoretical calculations also show that the O_2_ reaction, due to the lack of hydrogen atoms in the α-position, is not a sink for the aminyl radical under atmospheric conditions and that AMPNO (a *primary* nitrosamine) is thermally stable in the gas phase; the potential energy surface for dissociation reactions via the nitrosamine–hydroxydiazene isomerization^44,45^ is complex (see Figure S8) with two nitrosamine and four low-energy hydroxydiazene conformers and barriers effectively blocking any significant dissociation under atmospheric conditionsΔ*H*^⊖^ = −9:
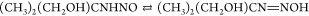
3Δ*H*^⊖^ = −216:
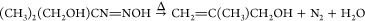
4aΔ*H*^⊖^ = −234:
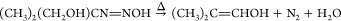
4bΔ*H*^⊖^ = +52:
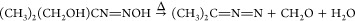
4c

Finally, the theoretical study finds the OH radical to react extremely fast with both nitrosamine and hydroxydiazene, *k*_OH_ > 1 × 10^–10^ cm^3^ molecule^–1^ s^–1^ at 298 K, resulting in CH_3_C(O)CH_3_, CH_2_O, and N_2_O (relative energies of stationary points on the PESes are presented in Figures S9 and S10).

#### 3.1.3 Secondary Photo-Oxidation Products

The major product in atmospheric AMP photo-oxidation is predicted to be CH_3_C(NH_2_)(CH_3_)CHO. Experimental room-temperature rate coefficients for OH reactions with the substituted 2-methylpropanes (CH_3_)_3_CCH_2_OH (*k*_OH_ = 5.2 × 10^–12^ cm^3^ molecule^–1^ s^–1^),^46^ (CH_3_)_3_CCHO (*k*_OH_ = 2.7 × 10^–11^ cm^3^ molecule^–1^ s^–1^),^47^ and (CH_3_)_3_CNH_2_ (*k*_OH_ = 8.4 × 10^–12^^19^ and 1.2 × 10^–11^^48^ cm^3^ molecule^–1^ s^–1^) show the −CHO group being around 5 times more reactive than the −CH_2_OH group and that there is no simple structure–activity model for substituted amines (note that the −CH_2_OH group is the proton donor in AMP, whereas the −NH_2_ group is the proton donor in CH_3_C(NH_2_)(CH_3_)CHO). In any case, CH_3_C(NH_2_)(CH_3_)CHO is expected to react around twice as fast with OH radicals as AMP does and that H abstraction from −CHO and −NH_2_ will be the dominant pathwaysΔ*H*^⊖^ = −125:
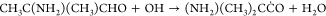
5aΔ*H*^⊖^ = −72:
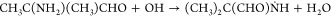
5b

The quantum chemistry calculations predict the barrier to dissociation of the carbonyl radical being only ∼14 kJ mol^–1^ (Figure S17, Table S13), which places the thermal unimolecular dissociation rate coefficient around 6 × 10^8^ s^–1^ at 298 KΔ*H*^⊖^ = +18:
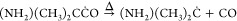
6

A master equation calculation, assuming equipartitioning of the enthalpy in reaction 5aa, shows the lifetime of the energized (NH_2_)(CH_3_)_2_CĊO radical formed in reaction 5a to be less than 10^–10^ s under atmospheric conditions. The rate coefficient for the competing O_2_ reaction, (NH_2_)(CH_3_)_2_CĊO + O_2_ → (NH_2_)(CH_3_)_2_CC(O)OȮ, is around 5 × 10^–12^ cm^3^ molecule^–1^ s^–1^ (k_∞,CH_3_CO+O_2__^49^) making it several orders of magnitude slower than the dissociation. The formation of peroxyacyl radicals, and subsequently peroxyacylnitrate, can consequently be disregarded under atmospheric conditions.

The (CH_3_)_2_(NH_2_)Ċ radical is also pivotal in the reactions following H abstraction from the OH group in AMP, as shown in Scheme 1. Two products arise: ∼95% (CH_3_)_2_C=NH and ∼5% CH_3_C(O)NH_2_.

The (CH_3_)_2_C(CHO)ṄH radical, formed in reaction 5b, is found to be meta-stable with barriers of 101 and 64 kJ mol^–1^ to the ejection of the −CH_3_ and −CHO groups, respectively (the underlying quantum chemistry data are collected in Table S14).Δ*H*^⊖^ = +73:
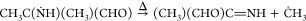
7aΔ*H*^⊖^ = +36:
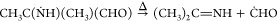
7b

RRKM calculations place the thermal rate constant for the dissociation of CH_3_C(ṄH)(CH_3_)(CHO) to be ∼60 s^–1^ at 298 K, which is 1 to 2 orders of magnitude faster than the competing bimolecular reactions with NO and NO_2_ under atmospheric conditions^42,43^ (for details, see the Supporting Information). A change in the barrier height by ±4 kJ mol^–1^ results in the change in the calculated rate coefficient by a factor of 5. That is, the dissociation rate will still be >10 times larger than the bimolecular rates, and it can therefore be concluded that, by far, the major product in atmospheric CH_3_C(NH_2_)(CH_3_)CHO photo-oxidation is propan-2-imine, (CH_3_)_2_C=NH.

Propane-2-imine, which is also a primary product following abstraction from the −NH_2_ group in AMP, undergoes further photo-oxidation in the atmosphere. There are no experimental data available for imine gas-phase reactions with OH radicals in the literature but two theoretical studies of the atmospheric chemistry of the simplest imine, CH_2_=NH,^50,51^ predict that its rate coefficient for the reaction with OH is ∼3 × 10^–12^ cm^3^ molecule^–1^ s^–1^, which is about 3 times slower than that of CH_2_=CH_2_.^52^ In contrast to CH_2_=CH_2_, the CH_2_=NH + OH reaction is predicted to be completely dominated by hydrogen abstraction with around 50% N–H abstraction,^50^ and N–H abstraction may likely also be an important route in the (CH_3_)_2_C=NH + OH reaction. Again, following the results from the theoretical study on the atmospheric chemistry of CH_2_=NH,^50^ the (CH_3_)_2_C=Ṅ radical may either eject ĊH_3_, resulting in CH_3_CN, or react with NO or NO_2_, resulting in (CH_3_)_2_C=NNO and (CH_3_)_2_C=NNO_2_, respectively. Further, OH addition to the π-system is activated by the σ-electrons donated by the methyl groups, and H abstraction from the methyl groups may also be facilitated due to the H bonding of the OH radical and the formation of a six-membered ring transition state. The OH addition is highly exothermic and may conceivably be followed by internal H transfer and CH_3_ ejection, leading to acetamideΔ*H*^⊖^ = −107:
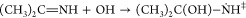
8Δ*H*^⊖^ = −24:
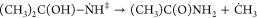
9

H abstraction from the methyl groups results in CH_3_(CHO)C=NH. In summary, the (CH_3_)_2_C=NH + OH rate coefficient is expected to be larger than that of CH_2_=NH but smaller than that of (CH_3_)_2_C=CH_2_ (8.5 × 10^–12^ cm^3^ molecule^–1^ s^–1^^53^).

The major products following H abstraction from the −CH_3_ groups in AMP are HN=C(CH_3_)CH_2_OH and CH_3_C(O)NH_2_ (and CH_2_O). The imine, HN=C(CH_3_)CH_2_OH, is likely more reactive than (CH_3_)_2_C=NH due to the −CH_2_– group being activated by the hydroxyl group,^54^ and atmospheric photo-oxidation consequently results in CH_3_(CHO)C=NH as the major secondary product. In analogy to the above-listed secondary products of (CH_3_)_2_C=NH, one may also expect CH_3_C(O)NH_2_ and CHO(CH_2_OH)C=NH, as well as nitroso- and nitroimine. Finally, acetamide reacts too slowly with OH radicals (*k*_OH_ = 7.5 × 10^–13^ cm^3^ molecule^–1^ s^–1^ at 298 K)^55^ to undergo any significant photo-oxidation.

### 3.2 Experimental Results

Seven photo-oxidation experiments were carried out under different conditions; *p*, *T*, RH, O_3_, NO, and NO_2_ mixing ratios, *j*_NO_2__, and particle mass loadings are detailed in Table S15 and Figures S18–S24. Six of the experiments were analyzed with respect to product formation and quantification, the seventh experiment was carried out employing different instrumental settings to uncover possible artifacts.

**Figure 2:**
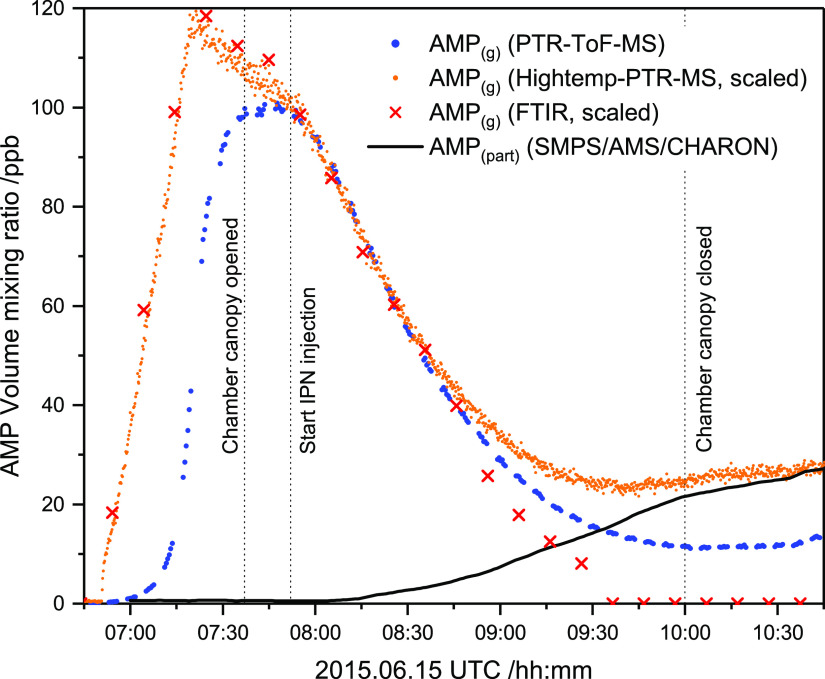
Comparison of the AMP gas-phase and particle-phase time profiles obtained by FTIR, high-temperature PTR-QMS, PTR-ToF-MS, and SMPS/AMS/CHARON.

The experiments are characterized by a low relative humidity between 1.5 and 2% (dew-point temperature around −30 °C). As detailed later in Section 3.2.3, all the experiments were accompanied by extensive particle formation that was initiated by the reaction of AMP with nitric acid, and the particles mainly consisted of this aminium salt. In some experiments, more than 50% of AMP was transferred from the gas phase to the particle phase. Figure 2 illustrates the gas-phase time profiles of AMP, as measured by long-path Fourier transform infrared (FTIR) spectroscopy , by PTR-ToF-MS, and by a high-temperature PTR-quadrupole MS (HT PTR-QMS) instruments, respectively. The figure includes the time profile of the particle-phase AMP content, as measured by the scanning mobility particle sizer (SMPS), AMS, and CHARON-PTR-ToF-MS instruments (same time profile observed by all three analyzers).

Figure 2 documents a significant delay of the response of the PTR-ToF-MS instrument upon the injection of AMP into the chamber. Once the instrumental surfaces of the PTR-ToF-MS instrument are conditioned with AMP, the data from all three analyzers agree well during the initial phase of the photo-oxidation experiments. During the later phase of the experiments, when the particle loading in the chamber is high, both the PTR-ToF-MS and the high-temperature PTR-QMS instruments register an increase in AMP. We explain this by the total (HT PTR-QMS) and partial (PTR-ToF-MS) evaporation of the aminium salt particle in the heated sampling lines and, in particular, in the drift tubes of the two PTR-MS analyzers.^56^ Comparisons of the AMP profiles obtained in the other experiments are presented in Figures S25–S29. We finally note that the PTR-ToF-MS instrument also exhibits a delayed response to AMPNO_2_ (Figure S30). The response time of the PTR-ToF-MS instrument to AMPNO_2_ is approximately 5 min. For reasons unknown, however, the apparent delay in both the HT PTR-QMS and PTR-ToF-MS instruments’ responses when AMPNO2 was injected into the chamber is close to 1 h; a similar instrument response delay was observed in the CSIRO experiments.^17^

#### 3.2.1 Gas-Phase Photo-Oxidation Products

The PTR-ToF-MS instrument was operated by alternating the drift tube electric field between *E*/*N* = 65 and 105 Td (1 Td = 10^–21^ V m^–2^) to recognize ion fragmentation facilitating the interpretation. At *E*/*N* = 65 Td, AMP is detected at *m*/*z* 90.092 (87.7%, C_4_H_12_NO^+^), 73.065 (2.3%, C_4_H_9_O^+^, NH_3_ ejection), 72.081 (1.6%, C_4_H_10_N^+^, H_2_O ejection), and 18.035 (8.4%, NH_4_^+^); at *E*/*N* = 105, the fragmentation is 55.5% *m*/*z* 90.092, 6.0% *m*/*z* 73.065, 5.5% *m*/*z* 72.081, and 33.0% *m*/*z* 18.035 (the relative intensities of low *m*/*z* peaks are not corrected for instrument mass discrimination). Protonated AMPNO_2_ undergoes more extensive fragmentation; calibration experiments show the major ion signal at *m*/*z* 73.065 (62%, C_4_H_9_O^+^, ejection of NH_2_NO_2_), whereas the protonated molecule (C_4_H_11_N_2_O_3_^+^) at *m*/*z* 135.076 only accounts for 38% of the total ion intensity at *E*/*N* = 65 Td (at *E*/*N* = 105 Td the *m*/*z* 135.076 signal was below detection level in the chamber measurements).

**Figure 3:**
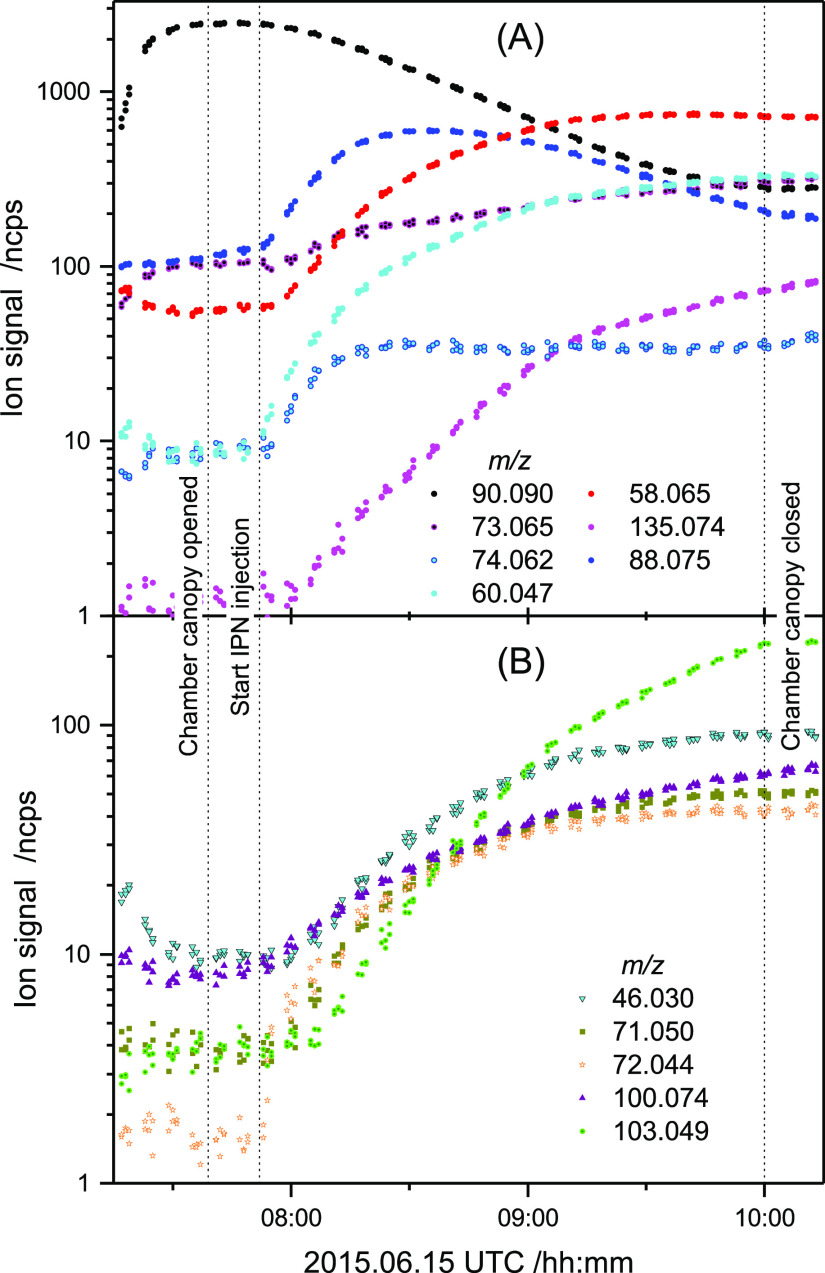
Major ion signals observed at *E*/*N* = 65 Td during the AMP photo-oxidation experiment on 2015.06.15. (A) Ion signal of AMP and primary products in the AMP + OH reaction. For clarity, the AMP fragment ion signal at *m*/*z* 72.081 is omitted. (B) Ion signals of secondary products. The *m*/*z* 74.062 raw signal shown is not corrected for the isotope contribution of *m*/*z* 73.065. Table 1 contains the correspondence between the *m*/*z* ratios shown in the figure and the chemical formulas they represent.

Figure 3 exemplifies the results from an experiment carried out under initial low-NO_*x*_ conditions. In this particular experiment, the initial NO_*x*_ level was around 15 ppbV, which slowly increased throughout the experiment as IPN was injected into the chamber to maintain a reasonably high OH level in the experiments [CH_3_CH(ONO)CH_3_ + *h*ν → CH_3_CH(Ȯ)CH_3_ + NO; CH_3_CH(Ȯ)CH_3_ + O_2_ → CH_3_C(O)CH_3_ + HO_2_; HO_2_ + NO → OH + NO_2_]. Around 10 min after opening the canopy exposing the chamber to solar radiation, IPN was injected with a flow of 0.3 μL min^–1^ in a stream of N_2_ into the chamber for 10 min (∼0.4 ppbV min^–1^). The flow was then reduced to 0.1 μL min^–1^ until the chamber canopy was closed, at which time a total of 16 μL IPN had been added to the chamber. The observed ion signals, relevant to AMP photo-oxidation, are presented in Table 1 together with our interpretation. Only ion signals having an intensity >2% of the decrease in the AMP signal *m*/*z* 90.092 at *E*/*N* = 65 Td, during the time the chamber canopy was open, are included in the table. Results from the other five experiments are illustrated in Figures S31–S35.

**Table 1:** Relevant Mass Peaks Detected by PTR-ToF-MS during AMP Photo-Oxidation Experiments^a^

		interpretation^b^		
*m*/*z*	ion sum formula	neutral molecule	origin	comments
18.034	NH_4_^+^	NH_3_	F,H	fragment from [AMP]H^+^ and NH_3_ from imine hydrolysis.
31.018	CH_3_O^+^	CH_2_O	P,S	product in multiple reactions
42.034	C_2_H_4_N^+^	CH_3_CN	S	from (CH_3_)_2_C=NH + OH; detected in three of six experiments.
44.014	CH_2_NO^+^	HNCO	S,H	from CHONH_2_ + OH and CH_3_C(O)NH_2_ + OH; observed in a few experiments at a high *E*/*N*
46.029	CH_4_NO^+^	CHONH_2_	H	product from AMP + HCOOH condensation
58.065	C_3_H_8_N^+^	(CH_3_)_2_C=NH	P,S	from NH_2_ abstraction in AMP, a secondary product from NH_2_ abstraction in (CH_3_)_2_(NH_2_)CCHO
59.049	C_3_H_7_O^+^	(CH_3_)_2_CO	H,F	(CH_3_)_2_CHONO (IPN), (CH_3_)_2_CO from IPN, from the hydrolysis of (CH_3_)_2_C=NH, product from AMPNO + OH?
60.044	C_2_H_6_NO^+^	CH_3_C(O)NH_2_	P,S	from CH_3_ abstraction in AMP, a secondary product of the (CH_3_)_2_(NH_2_)CCHO + OH reaction
71.049	C_4_H_7_O^+^		F,H	NH_2_NO_2_ ejection from [(CH_3_)_2_(CHO)CNHNO_2_]H^+^
72.044	C_3_H_6_NO^+^	CHO(CH_3_)C=NH	S	from HOCH_2_(CH_3_)C=NH + OH and (CH_3_)_2_C=NH + OH
72.081	C_4_H_10_N^+^	CH_3_C(NH_2_)(CH_3_)CH_2_OH	F	H_2_O ejection from [AMP]H^+^
73.065	C_4_H_9_O^+^	CH_2_(O)C(CH_3_)_2_	H,F	NH_3_ ejection from [AMP]H^+^, NH_2_NO_2_ ejection from [AMPNO_2_]H^+^, H_2_O ejection from [CH_3_C(OH)(CH_3_)CH_2_OH]H^+^, and fragment from [AMPNO]H^+^
74.060	C_3_H_8_NO^+^	HOCH_2_(CH_3_)C=NH	P	from CH_3_ abstraction in AMP
75.043	C_3_H_7_O_2_^+^	CH_3_C(O)CH_2_OH	H	from hydrolysis of HOCH_2_(CH_3_)C=NH, a possible CH_3_CH_2_COOH chamber artifact
88.039	C_3_H_6_NO_2_^+^	CHO(CH_2_OH)C=NH	S	from HOCH_2_(CH_3_)C=NH + OH
88.076	C_4_H_10_NO^+^	CH_3_C(NH_2_)(CH_3_)CHO	P	from −CH_2_ abstraction in AMP
90.092	C_4_H_12_NO^+^	CH_3_C(NH_2_)(CH_3_)CH_2_OH		AMP
100.075	C_5_H_10_NO^+^	(CH_3_)_2_(CHO)C–N=CH_2_	H	condensation product between (CH_3_)_2_(NH_2_)CCHO and CH_2_O
102.089	C_5_H_12_NO^+^	(CH_3_)_2_(CH_2_OH)C–N=CH_2_	H	condensation product between AMP and CH_2_O
103.049	C_3_H_7_N_2_O_2_^+^	(CH_3_)_2_C=NNO_2_	H,S	from (CH_3_)_2_C=NH; detected in five of six experiments
116.070	C_5_H_10_NO_2_^+^	(CH_3_)_2_(NH_2_)CCH_2_OC(O)H	H	formic acid ester of AMP
135.076	C_4_H_11_N_2_O_3_^+^	(CH_3_)_2_(CH_2_OH)CNHNO_2_	P	AMPNO_2_

a Only ion signals having an intensity >2% of the decrease in the AMP signal with *m*/*z* 90.092 at *E*/*N* = 65 Td during the time the chamber canopy was open are included in the table.

b Abbreviations: P, primary product; S, secondary product; H, product from heterogeneous chemistry (see text); and F, fragment ion.

It is instantly recognized from Figure 3 that three of the ion signals, growing during the AMP photo-oxidation, have very distinct temporal profiles: *m*/*z* 73.065, 135.074, and 103.049. It is obvious that there have to be supplement contributors to *m*/*z* 73.065 in addition to AMP and AMPNO_2_, see above. The two other signals, of which *m*/*z* 135.074 is indicative of AMPNO_2_, appear slightly delayed relative to the other ion signals, and both grow in intensity throughout every experiment—even after closing the chamber canopy to solar radiation—and heterogeneous chemistry reactions in the chamber and/or in the instrument sampling lines cannot be excluded. As mentioned above, there is also clear evidence in experiments with high-particle loading that particles evaporate in the heated sampling lines and in the instrument drift tube, resulting in quite deceptive readings toward the end of the experiments.

The NO_3_ radical may likely contribute to the chamber reactions under dark conditions, whereas the NO_3_ radical photolyzes quickly under sunlight conditions, never reaching significant levels (NO_3_ + *h*ν → NO + O_2_); the NO_3_ radical concentration builds up under dark conditions upon closing the chamber canopy. The NO_3_ radical concentration, calculated from the observed NO, NO_2_, and O_3_ concentrations (see Figures S18–S24),^57^ is ∼4 × 10^7^ cm^–3^, which just after closing the chamber canopy increases to ∼7 × 10^7^ cm^–3^ within 10 min and then decreases to ∼6 × 10^7^ cm^–3^ in the next 30 min. There is no experimental value for *k*_NO_3_+AMP_, but the empirical correlation between OH and NO_3_ rate coefficients for the reaction with amines implies *k*_NO_3_+AMP_ ≈ 3.7 × 10^–14^ cm^3^ molecule^–1^ s^–1^ at 298 K,^6^ making the reaction an order of magnitude too slow to explain all the continued nitramine formation.

##### 3.2.1.1 H Abstraction from the −CH_2_– Group in AMP

H abstraction from the −CH_2_– group in AMP is predicted in the theoretical calculations to account for more than 70% of the AMP + OH reaction. In accordance, one of the largest ion signals observed in all the experiments, *m*/*z* 88.076 (C_4_H_10_NO^+^), is attributed to 2-amino-2-methylpropanal, CH_3_C(NH_2_)(CH_3_)CHO.

A distinct transient profile of *m*/*z* 88.076 is typical for a reactive product. As already detailed in Section 3.1.3, CH_3_C(NH_2_)(CH_3_)CHO is expected to react around twice as fast with OH radicals as AMP does. The *m*/*z* 88.076 profile gives a somewhat deceptive visual impression of the actual −CH_2_– abstraction yield—the maximum signal intensity, occurring after around 1 h of reaction, is actually less than half of what it would have been, had the compound not reacted with OH. There is no evidence of the compound fragmenting in the PTR-MS instruments; the *m*/*z* 71.049 ion signal (C_4_H_7_O^+^), that in principle could derive from [CH_3_C(NH_2_)(CH_3_)CHO]H^+^ → [CH_3_C(CH_3_)CHO]^+^ + NH_3_, is not correlated with *m*/*z* 88.076 but has a typical time profile of secondary products.

The theoretical study further shows that aldehydic H abstraction from CH_3_C(NH_2_)(CH_3_)CHO leads to the tertiary (CH_3_)_2_(NH_2_)Ċ radical and not to the formation of an intermediate peroxyacylnitrate, (CH_3_)_2_(NH_2_)CC(O)OONO_2_. This is corroborated by titration with NO toward the end of each photo-oxidation experiment, where the addition of excess NO only produces insignificant correlated changes in the ion signals observed. The theoretical study also shows that N–H abstraction from CH_3_C(NH_2_)(CH_3_)CHO results in > 99% (CH_3_)_2_C=NH. In agreement, the second largest product signal observed, *m*/*z* 58.065 (C_3_H_8_N^+^), is attributed to (CH_3_)_2_C=NH. The other product, acetamide, is identified at *m*/*z* 60.044 (C_2_H_6_NO^+^) and tentatively quantified despite the strong ion signals, caused by the use of IPN as an OH precursor [*m*/*z* 59.049 (C_3_H_7_O^+^, 100%) and isotopes 60.053 (3.3%) and 61.056 (0.1%)], complicating the spectral interpretation; the acetone isotopes were taken into consideration when estimating the concentration of acetamide.

While acetamide reacts slowly with OH radicals (*k*_OH_ = 7.5 × 10^–13^ cm^3^ molecule^–1^ s^–1^ at 298 K),^55^ propane-2-imine undergoes further reaction during the experiments. The theoretical study points to CH_3_CN, (CH_3_)_2_C=NNO, (CH_3_)_2_C=NNO_2_, CH_3_(CHO)C=NH, and CH_3_C(O)NH_2_ as possible products. 2-Iminopropanal, CH_3_(CHO)C=NH, is also an expected secondary product following H abstraction from the methyl groups in AMP, see below. Acetonitrile is a frequent background contaminant in many laboratories running HPLC instrumentation. However, *m*/*z* 42.034 was detected within the 2% cutoff limit in 3 of 6 experiments with temporal profiles consistent with CH_3_CN being a secondary product in the AMP photo-oxidation. The two other potential products, (CH_3_)_2_C=NNO and (CH_3_)_2_C=NNO_2_, are expected to show up in the PTR-MS instrument as the protonated molecules at *m*/*z* 87.056 (calculated fragmentation: (CH_3_)_2_C=NH + NO^+^) and 103.049 (calculated fragmentation: (CH_3_)_2_C=NH + NO_2_^+^), respectively. The *m*/*z* 87.056 was not detected in any of the present experiments, whereas the *m*/*z* 103.049 signal was detected within the 2% cutoff limit in 5 of 6 experiments; in all instances with a temporal profile slightly delayed relative to the other ion signals and growing in intensity throughout the experiments (Figure 3).

H abstraction from the −CH_3_ groups in CH_3_C(NH_2_)(CH_3_)CHO is, in all likelihood, only a minor route in the CH_3_C(NH_2_)(CH_3_)CHO + OH reaction. In any case, H abstraction from the −CH_3_ groups is expected to give CH_3_C(NH_2_)(CHO)_2_, which was not detected by the PTR-MS instrument in any of the present experiments within the 2% cutoff limit at the expected *m*/*z* 102.056 (C_4_H_8_NO_2_^+^).

##### 3.2.1.2 H Abstraction from the −NH_2_ Group in AMP

H abstraction from the −NH_2_ group in AMP is projected by the theoretical calculations to account for between 1 and 20% of the AMP + OH reaction; the anticipated products are: CH_2_O, (CH_3_)_2_C=NH, AMPNO_2_, and AMPNO. Formaldehyde, which is a common chamber artifact, was detected at *m*/*z* 31.018 (CH_3_O^+^) by the PTR MS instrument operated with *E*/*N* = 105 Td. The temporal profiles of formaldehyde clearly show the compound to be a primary product in all the experiments. However, formaldehyde is not a product unique to the N–H abstraction route. The same is true for propan-2-imine, (CH_3_)_2_C=NH, which is also a secondary photo-oxidation product following H abstraction from the −CH_2_– group in AMP, as explained above.

The PTR-MS signals of AMPNO_2_, *m*/*z* 73.065 (C_4_H_9_O^+^) and 135.076 (C_4_H_11_N_2_O_3_^+^), were detected in all the experiments. As mentioned, the *m*/*z* 135.076 ion signal grows in intensity throughout every experiment, which is not consistent with its origin being a molecular species only produced in the gas phase. The *m*/*z* 73.065 ion signal, which has contributions from AMP as well as other species (see below), does therefore not constitute an unambiguous identification of AMPNO_2_ resulting from gas-phase chemistry. We note that Li et al.^17^ “identified” AMPNO_2_ in their AMP photo-oxidation experiments by SIFT-QMS through *m*/*z* 164, which is the ion–molecule product of AMPNO_2_ with reagent ion NO^+^.

Concerning AMPNO, it is known from aqueous-phase chemistry that nitrosamines from primary amines are very unstable^58^ and that they quickly react (acid-catalyzed) to the corresponding alcohols: (R–NHNO_(aq)_ ⇆ R–N=NOH_(aq)_; R–N=NOH_(aq)_ + H_(aq)_^+^ ⇆ R–N=*N*OH_2(aq)_^+^ → R_(aq)_^+^ + N_2_ + H_2_O → ROH_(aq)_ + N_2_ + H_(aq)_^+^). The theoretical calculations, however, predict AMPNO to be thermally stable in the gas phase and indicate a lifetime of around 500 s in the chamber experiments, see the Supporting Information. The theoretical study also indicates that around 50% of the AMPNO formed reacts with OH radicals under the conditions in the chamber experiments resulting in CH_3_C(O)CH_3_, CH_2_O, and N_2_O.

It is not possible to verify the formation and the existence of AMPNO in the gas phase explicitly by PTR-MS in any of the present experiments. Both acetone and formaldehyde are common chamber artifacts and both also have other sources in the AMP photo-oxidation. Nitrous oxide cannot be detected by PTR, and the FTIR employed was not sensitive enough to reveal single digit ppbV amounts of N_2_O being formed in the photo-oxidation experiments.

One possible explanation to the failure of PTR-MS instrument detecting the protonated molecule is that protonated AMPNO fragments readily; quantum chemistry calculations show that protonation takes place at the nitroso group and that there is no electronic barrier in addition to Δ_fragment_*E*_0_ to ejection of HNNOHΔ*H*^⊖^ = −182:
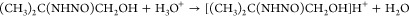
10Δ*H*^⊖^ = +154:
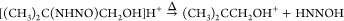
11aΔ*H*^⊖^ = +509:
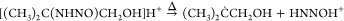
11b

Further, the resulting cation signal [(CH_3_)_2_CCH_2_OH^+^, *m*/*z* 73.065] has also contributions from both protonated AMP and AMPNO_2_, nullifying this ion signal as evidence for nitrosamine formation.

A second explanation is linked to nitrosamine hydrolysis in the chamber and/or in the PTR-MS detection system, in the present case resulting in 2-methylpropane-1,2-diol. Laboratory experiments, employing a validated CH_3_C(OH)(CH_3_)CH_2_OH sample show two ion signals *m*/*z* 91.076 (∼30%) and 73.065 (∼70%, H_2_O ejection) at *E*/*N* = 65 Td. At the resolving power of the PTR-MS instrument, the AMP isotope signals coalesce at *m*/*z* 91.092, and the only ion signal observed in the vicinity of *m*/*z* 91.076 is an extremely weak peak at *m*/*z* 91.051 that shows a relatively flat temporal profile. The *m*/*z* 73.065 (C_4_H_9_O^+^) temporal profile differs from all the other ion signals shown in Figure 3. However, after subtracting the known contributions, the signal profile resembles that of a photo-stable product growing alike the identified products. It can therefore unambiguously be concluded that *m*/*z* 73.065 does not result from protonated AMPNO (reaction 11a), in which case the signal should have shown a transient profile. It can also be ruled out that *m*/*z* 73.065 originates from AMPNO hydrolyzed in the PTR-MS detection system or in the chamber, in which case it should have been accompanied by *m*/*z* 91.076.

A third explanation is that the nitrosamine does undergo rearrangement and dissociation according to eqs 3 and 4a, 4b, and 4c, that is the barriers involved are significantly lower than calculated (see Section 3.1.2). In that case, one should observe either protonated CH_2_=C(CH_3_)CH_2_OH (C_4_H_9_O^+^, *m*/*z* 73.065), (CH_3_)_2_C=CHOH (C_4_H_9_O^+^, *m*/*z* 73.065) or (CH_3_)_2_C=N=N (C_3_H_7_N_2_^+^, *m*/*z* 71.060). The *m*/*z* 73.065 ion signal has quantified contributions from protonated AMP and AMPNO_2_, and the profile leaves little evidence for an additional contribution that by necessity is time correlated to AMPNO_2_. 2-Diazopropane is expected to react equally fast with OH as diazomethane does, *k*_OH+CH_2_NN_ = 1.7 × 10^–10^ cm^3^ molecule^–1^ s^–1^,^59^ and it will therefore not build up during the present photo-oxidation experiments. The observed signal at *m*/*z* 71.049 neither displays any skewness toward higher values nor a transient profile. Diazomethane shows significant fragmentation upon the proton reaction transfer reaction with H_3_O^+^ under similar instrumental conditions (CH_2_NN + H_3_O^+^ → CH_3_N_2_^+^ + H_2_O; CH_3_^+^ + N_2_ + H_2_O; CH_5_O^+^ + N_2_) and other ion signals that could indicate the presence of 2-diazopropane are consequently *m*/*z* 43.055 (C_3_H_7_^+^) and *m*/*z* 61.065 (C_3_H_9_O^+^). The C_3_H_7_^+^ ion has multiple origins, it is always observed in chamber experiments, it grows throughout the experiments and its time profiles are never correlated to any known species related to AMP photo-oxidation. The C_3_H_9_O^+^ ion is not observed in any experiment with an intensity >1% of the decrease in the AMP signal during the time the chamber canopy was open.

Finally, a fourth explanation is simply that the nitrosamine level in the experiments was below the PTR-MS detection limit (∼50 pptV). In any case, the *m*/*z* 135.076 ion signal is the only experimental evidence of H abstraction from the −NH_2_ group in the gas phase.

##### 3.2.1.3 H Abstraction from the −CH_3_ Groups in AMP

H abstraction from the −CH_3_ groups in AMP is predicted to account for between 5 and 10% of the AMP + OH reaction. Four products are anticipated to appear in this route: CH_2_O, CH_3_C(O)NH_2_, HN=C(CH_3_)CH_2_OH, and (CH_3_)(CHO)(CH_2_OH)CNH_2_; the latter two are unique to this path. The imine, HN=C(CH_3_)CH_2_OH, is recognized by the ion signal at *m*/*z* 74.060 (C_3_H_8_NO^+^) that shows a temporal profile, indicating secondary reactions during the experiments; the *m*/*z* 74.060 peak is corrected for the C_4_H_8_O isotope contribution (parent peak, C_4_H_9_O^+^*m*/*z* 73.065). The minor product, HOCH_2_C(NH_2_)(CH_3_)CHO, that is predicted to account for less than 5% of the products following H abstraction from the −CH_3_ groups, was not observed in any of the present experiments. The theoretical study suggests that the HN=C(CH_3_)CH_2_OH reaction with OH primarily leads to (CH_3_)(CHO)C=NH, which we tentatively ascribe to the PTR-MS signal *m*/*z* 72.044 (C_3_H_6_NO^+^) having a secondary product time profile. The weak ion signal *m*/*z* 88.039 (C_3_H_6_NO_2_^+^), also having a secondary product profile, is tentatively attributed to CHO(CH_2_OH)C=NH.

#### 3.2.2 Heterogeneous Chemistry Products

6 of the 21 ion signals listed in Table 1 (*m*/*z* 44.014, 46.029, 75.043, 100.075, 102.089, and 116.070)—all detected in more than half of the experiments with an intensity above the 2% intensity cutoff—cannot be reconciled with AMP gas-phase photo-oxidation only. Three of these six minor ion signals at *m*/*z* (C_5_H_10_NO^+^), 102.089 (C_5_H_12_NO^+^), and 116.070 (C_5_H_10_NO_2_^+^) correspond to species having one more carbon atom than AMP itself, suggesting that heterogeneous processing takes place in the chamber and/or in the heated sampling lines to the gas-phase analyzers.

Amines are known to form imines in condensation reactions with carbonyl compounds in solution,^60^ and on surfaces.^61^ Primary amines attached to a tertiary alkyl group give “stable” imines with primary aldehydes as steric hindrance making aldol condensations difficult.^60^ The *m*/*z* 100.075 and 116.070 signals are delayed relative to gas-phase product signals and grow in intensity throughout the experiments, whereas *m*/z 102.089 appears early and decreases again later in the experiments. The *m*/z 102.089 is recognized from laboratory experiments as the AMP condensation product with formaldehyde, and the condensation may well take place in the PTR-MS instrument inlet lines^62^
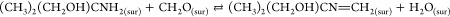
12

Similarly, the *m*/*z* 100.075 is attributed to condensation between the major, primary product (CH_3_)_2_(CHO)CNH_2_, and formaldehyde
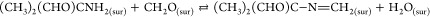
13

The *m*/*z* 116.070 (C_5_H_10_NO_2_^+^) is tentatively ascribed to the formic acid ester of AMP
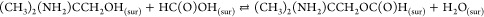
14

Amines also undergo addition reactions with organic acids. Formic acid is omnipresent in chamber experiments, and its reaction with AMP results in the formation of an oxirane (2,2-dimethyloxirane) and formamide, see Scheme S5. The oxirane can also be formed in an intramolecular AMP reaction
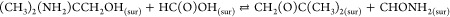
15
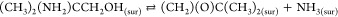
16

In PTR-MS, the oxirane (C_4_H_8_O) shows up as the protonated molecule at *m*/*z* 73.065, which also has contributions from both AMP and AMPNO_2_. The presence of formamide, to which there are no obvious chemical routes in AMP gas-phase photo-oxidation, is evidenced by *m*/*z* 46.029 that was observed in five of the six experiments with temporal profiles resembling those of secondary products, Figure 3.

As mentioned, the 2 ion signals *m*/*z* 103.049 (C_3_H_7_N_2_O_2_^+^) and 135.074 (C_4_H_11_N_2_O_3_^+^) show very similar temporal profiles, indicating that a possible contribution from heterogeneous chemistry reactions in the chamber and/or in the heated sampling lines cannot be ignored. The former signal is attributed to (CH_3_)_2_C=NNO_2_, the latter to AMPNO_2_. In addition to the abovementioned gas-phase routes leading to these two compounds, we speculate that simple surface reactions (mechanisms illustrated in Scheme S5) involving HNO_3_ may take place
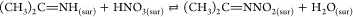
17
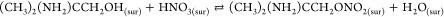
18a
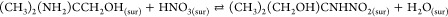
18b

AMPNO_2_ can be formed either directly in the reaction between AMP and HNO_3_ on surfaces or indirectly via the nitrate ester of AMP, which subsequently acts as a nitro donor.

Alkylnitrates fragment severely upon protonation,^63^ and the nitric acid ester, (CH_3_)_2_(NH_2_)CCH_2_ONO_2_, should it be formed on the particles or in the sampling lines, is expected to primarily show up at *m*/*z* 88.076 (NO_2_-ejection) and 72.081 (HNO_3_-ejection), whereas the protonated molecule signal at *m*/*z* 135.076 should be almost 2 orders of magnitude smaller. The signals at *m*/*z* 88.076 and 72.081 also have contributions from 2-amino-2-methylpropanal and AMP, respectively, and it is consequently not possible to resolve by PTR-MS, if 2-amino-2-methylpropyl nitrate, AMPNO_2_, or both are actually formed in the particle phase in the present experiments.

Reactions similar to 17 and 18a, 18b are also foreseen to occur for both (CH_3_)(CH_2_OH)=NH and (CH_3_)_2_(CHO)CNH_2_ resulting in, respectively, (CH_3_)(CH_2_OH)=NNO_2_ and (CH_3_)_2_(CHO)CNHNO_2_, of which the latter was not detected in the present experiments.

Imines undergo addition reactions with water and amines.^60^ The two imines formed in the AMP photo-oxidation, (CH_3_)_2_C=NH and (CH_3_)(CH_2_OH)C=NH, are expected to react with water on particles and chamber walls to give NH_3_ and the corresponding ketones (CH_3_)_2_CO and (CH_3_)(CH_2_OH)CO, respectively. The use of IPN as an OH precursor, resulting in acetone, hinders the verification of (CH_3_)_2_C=NH hydrolysis in the chamber, but the *m*/*z* 75.043 (C_3_H_7_O_2_^+^) signal, ascribed to hydroxyacetone, is observed in all the experiments. We note that *m*/*z* 75.043 also could be due to propanoic acid—a common chamber artifact. However, the temporal signal profile of *m*/*z* 75.043 aligns with those of the other products.

The imine exchange reactions with the two primary amines present during the chamber experiments, (CH_3_)_2_(CH_2_OH)CNH_2_ (AMP) and the primary product (CH_3_)_2_(CHO)CNH_2_, results in (CH_3_)_2_C=NC(CH_3_)_2_CH_2_OH (expected PTR-MS signals at *m*/*z* 130.123/112.113), (CH_3_)_2_C=NC(CH_3_)_2_CHO (expected PTR-MS signal at *m*/*z* 128.108), (CH_3_)(CH_2_OH)C=N–C(CH_3_)_2_CH_2_OH (expected PTR-MS signals at *m*/*z* 146.118/128.108), and (CH_3_)(CH_2_OH)C=N–C(CH_3_)_2_CHO (expected PTR-MS signals at *m*/*z* 144.102/126.092). None of these imines were detected in the gas phase with a ion signal intensity larger than 2% of the decrease in the AMP signal during the experiments.

#### 3.2.3 Particle-Phase Characterization

**Figure 4:**
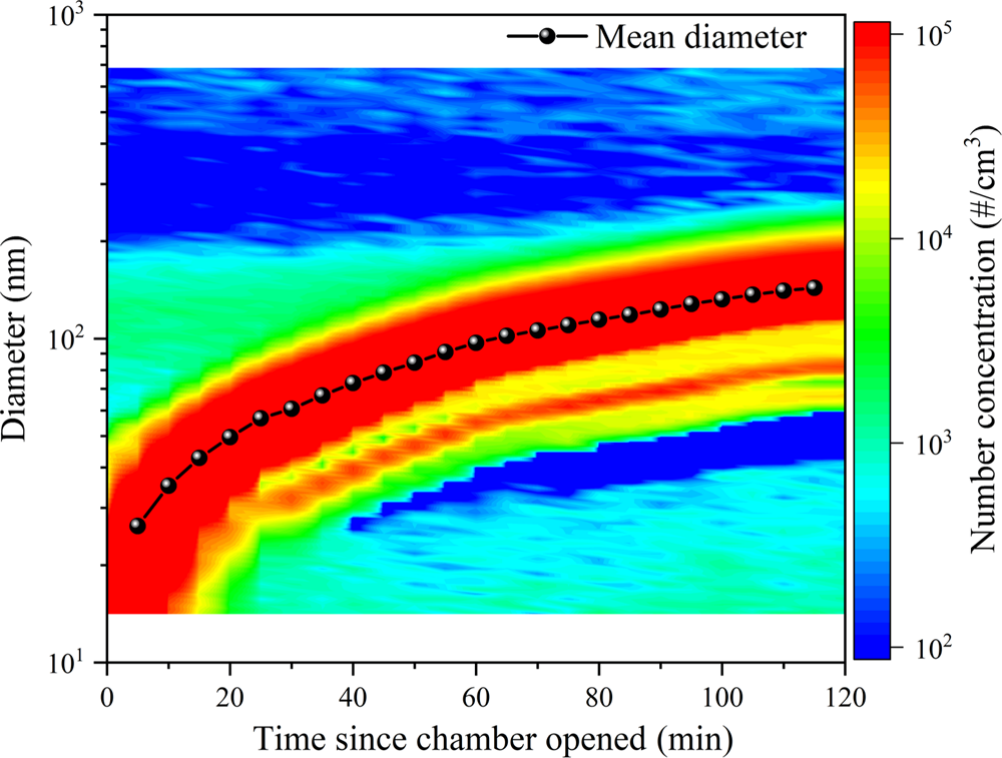
Particle number concentration and particle size distribution as a function of time during the photo-oxidation experiment on 2015.06.15.

Strong particle formation was observed in all the present AMP photo-oxidation experiments alike in the previously reported trials carried out in the UCR EPA^11^ and CSIRO^17^ indoor environmental chambers. We reiterate that the present experiments are characterized by a low relative humidity of < 2%, which slows the particle growth. Figure 4 illustrates the time evolution of particles, measured by SMPS, displaying number concentrations reaching 10^5^ cm^–3^ and a continuous size growth throughout the experiments.

A non-negligent number of particles were already formed during the reactant mixing in the chamber before opening the canopy to solar radiation; these particles were formed in the acid–base reaction of AMP with traces of HNO_3_ initially injected together with the NO/NO_2_ as an impurity and later resulting from the NO_2_ reaction with OH radicals. The AMS shows that AMP nitric acid salt accounts for >80% of the total particle mass and that ammonium nitrate only makes up 3–5% of the mass, see Table S16. The particle yields in high-NO_*x*_ and low-NO_*x*_ experiments are illustrated in Figure S36.

**Figure 5:**
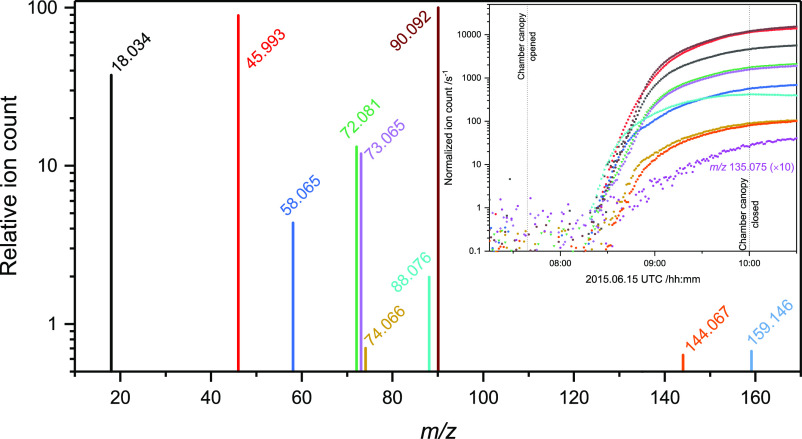
CHARON-PTR-ToF mass spectrum and its time evolution. Ion signals with intensity less than 0.5% of the AMP signal *m*/*z* 90.090 at 10:00 UTC, and ion signals related to the ion source and to isotopes are excluded. Data from the AMP photo-oxidation experiment conducted on 2015.06.15.

Figure 5 shows the time profiles of the relative ion signals obtained by CHARON PTR-ToF-MS; the instrument size range bias (100–750 nm) is revealed as a plain signal delay by around 45 min, cf. particle growth curve, as shown in Figure 4. The mass spectrum is very simple considering that the cutoff in the ion signal is 0.5% of the *m*/*z* 90.092 AMP signal. The AMP nitrate salt [recognizable by *m*/*z* 90.092, 73.065, 72.081, 18.034, and 45.993 (NO_2_^+^)] is by far the most dominant particle component. It is also evident from the relative ion intensities that the particles contain very little of the major AMP photo-oxidation products 2-amino-2-methylpropanal (*m*/*z* 88.076) and propan-2-imine (*m*/*z* 58.065). The latter is both a primary and secondary photo-oxidation product, but it is a stronger base than both AMP and 2-amino-2-methylpropanal, and may therefore displace these compounds in the particle phase. Also, the minor gas-phase product from −CH_3_ abstraction, (CH_3_)(CH_2_OH)C=NH, is identified in the aerosol at *m*/*z* 74.066 (peak corrected for the *m*/*z* 73.065, C_4_H_9_O^+^, isotope contribution). The *m*/*z* 144.067 and 159.146 peaks, for which we find no obvious corresponding chemical formulae, are the strongest of a plethora high mass ion signals evidencing particle processing. Only very small amounts of AMPNO_2_ (*m*/*z* 135.076) were detected in the particles by CHARON-PTR-MS; Figure 5 includes a 10-fold amplified *m*/*z* 135.075 signal. Calibration experiments with nanoparticles containing AMP nitrate and AMPNO_2_ place an upper limit of 110 ng/m^3^ AMPNO_2_ in the particles formed during the illustrated photo-oxidation experiment.

A closer inspection of the ion-signal time profiles displayed as the inset in Figure 5 shows that the profiles of *m*/*z* 58.065, 74.066, 135.075, and 159.146 all show features indicating aerosol processing. The growths of the *m*/*z* 58.065 and 74.066 signals from the two imine photo-oxidation products, (CH_3_)_2_C=NH and (CH_3_)(CH_2_OH)C=NH, initially resemble that of AMP (*m*/*z* 90.090) but then change their slope. Although very speculative, we suggest that this could be a sign of commencing gas-phase water transfer to the particle phase resulting in the hydrolysis of the two imines to give acetone, hydroxyacetone, and NH_3_ of which the former two are then released to the gas phase, see above. The Figure 5 inset as well as the corresponding plots from the other experiments are collected in Figures S37–S43.

**Figure 6:**
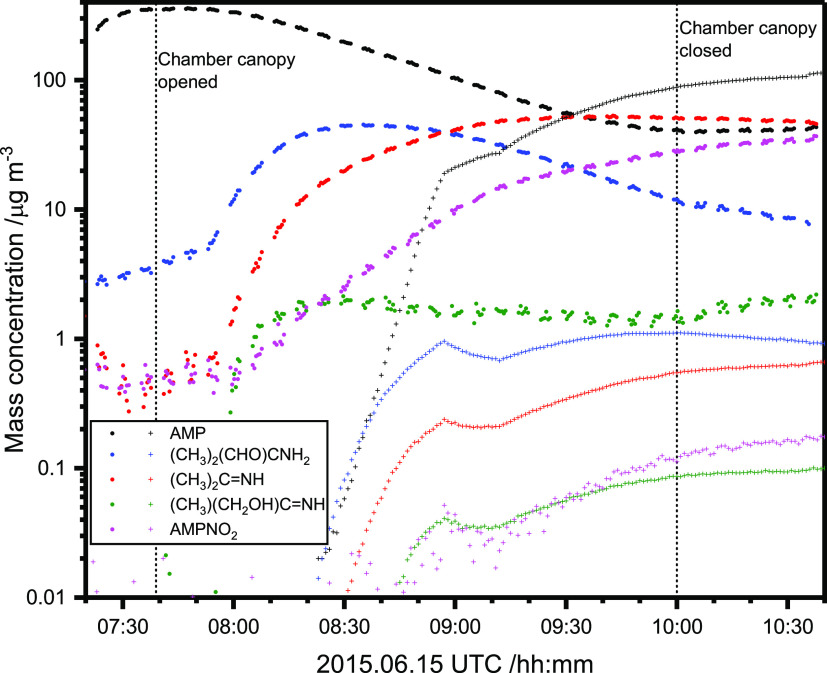
Time evolution of AMP (*m*/*z* 90.090), CH_3_C(NH_2_)(CH_3_)CHO (*m*/*z* 88.076), (CH_3_)_2_C=NH (*m*/*z* 58.065), HOCH_2_(CH_3_)C=NH (*m*/*z* 74.063), and AMPNO_2_ (*m*/*z* 135.094) in the gas phase (•) and the particle phase (+) during the photo-oxidation experiment on 2015.06.15.

The −NHNO_2_ group is acidic and HNO_3_ will therefore not drive AMPNO_2_ partitioning to the particle phase. Figure 6 compares the temporal profiles of AMP and the photo-oxidation products (CH_3_)_2_(CHO)CNH_2_, (CH_3_)_2_C=NH, (CH_3_)(CH_2_OH)C=NH, and AMPNO_2_ in the gas and particle phases. It can be seen that AMPNO_2_ initially increases in the gas phase very much like other primary photo-oxidation products, but in contrast to the other compounds that level off as AMP decreases, the AMPNO_2_ signal continues to increase—even after the chamber canopy is closed blocking further photo-oxidation. Similar time profiles are observed in some of the other experiments, suggesting that at least some production of AMPNO_2_ occurs on the particles.

##### 3.2.3.1 Aerosol Filter Samples

**Figure 7:**
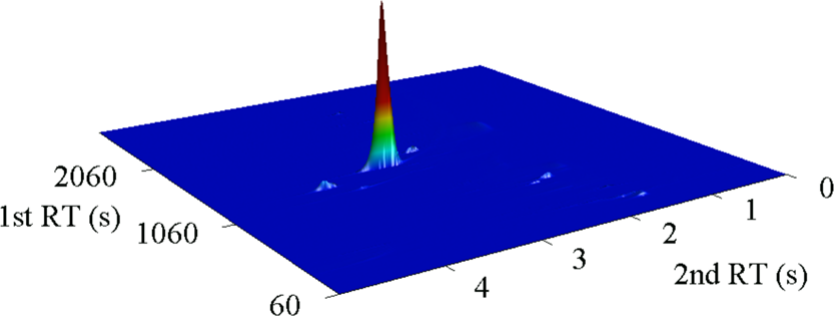
GC×GC-NCD chromatogram showing the detection of AMPNO_2_ in an aerosol sample collected at the end of the AMP photo-oxidation experiment on 2015.06.15. Several minor organic nitrogen species are also present in the particle phase.

Aerosol filter samples were collected at the end of each photo-oxidation experiment. Figure 7 shows the GC×GC-NCD chromatogram for the aerosol filter sample collected at the end of the AMP photo-oxidation experiment on 2015.06.15. The main peak shown is AMPNO_2_, which was easily detected within the complex matrix due to the combination of two-dimensional GC separation and nitrogen-specific detection. AMPNO_2_ was detected in all the aerosol samples collected at the end of the AMP photo-oxidation experiments; additional chromatograms from the other experiments can be found in Figures S44–S46; the amounts of AMPNO_2_ recovered from the collected aerosol filter samples are included in Table S16. The aerosol filter and the CHARON online results for AMPNO_2_ are apparently incommensurable, and we tentatively suggest that heterogeneous reactions, outlined in Section 3.2.2, also take place on the aerosol filters during sampling.

##### 3.2.3.2 AMP Nitrate Volatility

**Figure 8:**
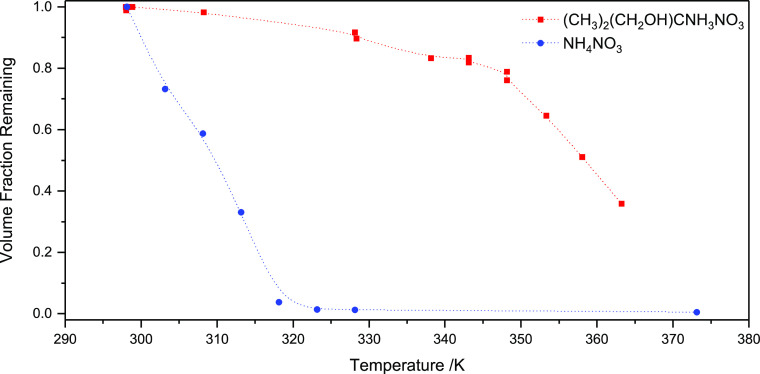
Volume fraction remaining of AMP nitrate (red) and ammonium nitrate (blue) in nanoparticles, as measured by VTDMA.

AMP nitrate volatility was studied using a home-made volatility tandem differential mobility analyzer (VTDMA)^64^ employing pure ammonium nitrate as reference. Figure 8 compares the results from volatility measurements of ammonium nitrate and AMP nitrate. The apparent change in the VFR curvature for the AMP–nitrate particles between 330 and 345 K may likely be related to the transition from deliquescent to dry particles. The vapor pressure of AMP nitrate, *p*^0^ = (1.3 ± 0.3) × 10^–5^ Pa at 298 K, and the enthalpy of vaporization, Δ_vap_*H* = 80 ± 16 kJ mol^–1^, were derived assuming the evaporation takes place in a liquid and not from a crystalline phase, see Salo et al. for a details.^65^

### 3.3 Modeling the Chamber Photo-oxidation

The present theoretical calculations can only place conservative limits on the initial branching in the AMP + OH reaction; the product distribution within each of the abstraction routes, as shown in Scheme 1, is associated with far lesser uncertainty. We have included subsequent photo-oxidation of the primary products in the chamber model; the rate coefficients employed in the model have already been discussed in Section 3.1.3 and are compiled in Table 2. The most important secondary reaction, (CH_3_)_2_(CHO)CNH_2_ + OH, is assumed to result in 95% (CH_3_)_2_C=NH and 5% CH_3_C(O)NH_2_, see Section 3.2.1.

**Table 2:** Rate Coefficients and Branching Employed in Modeling the OH-Initiated Degradation of AMP under Atmospheric Conditions

reaction	rate coefficient^a^	reference
(CH_3_)_2_(CH_2_OH)CNH_2_ + OH → 0.42·(CH_3_)(CH_2_OH)C=NH + 0.28·CH_3_C(O)NH_2_ + 0.06·(CH_3_)_2_(CHO)CNH_2_+ 0.24·(CH_3_)_2_(CH_2_OH)CṄH	2.8 × 10^–11^	(10)
(CH_3_)_2_(CHO)CNH_2_ + OH → 0.95·(CH_3_)_2_C=NH + 0.05·CH_3_C(O)NH_2_	7.0 × 10^–11^	estimated
(CH_3_)_2_C=NH + OH → CH_3_CN + CH_2_O	2.0 × 10^–11^	estimated
(CH_3_)(CH_2_OH)C=NH + OH → (CH_3_)(CHO)C=NH	2.0 × 10^–11^	estimated
(CH_3_)_2_(CH_2_OH)CṄH → (CH_3_)_2_C=NH + CH_2_O	4.6 × 10^–3^	estimated^b^
(CH_3_)_2_(CH_2_OH)CṄH + NO → (CH_3_)_2_(CH_2_OH)CNHNO	(8.5 ± 1.4) × 10^–14^	(43)
(CH_3_)_2_(CH_2_OH)CṄH + NO_2_ → (CH_3_)_2_(CH_2_OH)CNHNO_2_	(3.2 ± 0.5) × 10^–13^	(43)
(CH_3_)_2_(CH_2_OH)CNHNO + OH → CH_3_C(O)CH_3_ + CH_2_O + N_2_O	1.0 × 10^–10^	calculated
(CH_3_)_2_(CH_2_OH)CNHNO + *h*ν → (CH_3_)_2_(CH_2_OH)CṄH + NO	0.34 × *j*_NO_2__	(6)
(CH_3_)_2_(CH_2_OH)CNHNO_2_ + OH → (CH_3_)_2_(CHO)CNHNO_2_	1.4 × 10^–11^	estimated
CH_3_C(O)NH_2_ + OH → HNCO	(7.5 ± 3.5) × 10^–13^	(55) and (66)

a Bimolecular rate coefficients in units of cm^3^ molecule^–1^ s^–1^ and unimolecular rate coefficients in units of s^–1^.

b 0.2 × *k*_dissociation_ = 2.3 × 10^–2^ from the theoretical study.

AMP has large surface affinity and the “natural” lifetime of AMP in the chamber was derived from the gas-phase time profiles of AMP prior to opening the chamber canopy. Assuming the apparent AMP removal to be of first order, the wall loss rate coefficient, *k*_wall_, was found to be in the range 4–5 × 10^–5^ s^–1^, corresponding to a lifetime of around 6 h in the chamber. The AMP loss to the chamber walls is significant; in this respect, the low-NO_*x*_ experiment (2015.06.17) is extreme showing ∼50% wall loss, as listed in Table S17, while the other experiments show “moderate” wall losses between 7 and 30%.

The prominent particle formation in the photo-oxidation experiments dictates AMP gas-to-particle transfer being built-in explicitly in the chamber chemistry model; Table S17 includes the volume fraction AMP being transferred to the particle phase in the individual experiments. The particle-phase characterization (Section 3.2.3) has established the AMP nitrate salt as by far the most dominant particle component, and the VTDMA study shows that the AMP nitrate salt to some degree vaporizes in the heated sample transfer lines and indisputably in the PTR-ToF-MS drift tube (Figures 2 and S25–S29). The manifested AMP reading will therefore be biased toward higher values which, in particular, under heavy-particle loading, makes modeling based on the plane instrument readings somewhat uncertain. To a first approximation, we have therefore corrected the plain PTR-ToF-MS gas-phase values by a fraction of the CHARON-PTR-ToF-MS particle-phase values such that the FTIR and PTR values for AMP align as well as possible. The corresponding corrections from particle evaporation to the plain PTR-ToF-MS gas-phase values for the AMP photo-oxidation products are < 3% of their apparent values.

The OH radical concentration during photo-oxidation was extracted from the corrected time profiles of the AMP gas and particle phases according to eq 19 and then used in modeling the subsequent degradation reactions of the primary AMP photo-oxidation products.
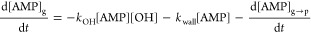
19

The AMP photo-oxidation experiments were, with one exception, fueled by a constant injection of the OH precursor, IPN, after an initiation period (Table S13). This is also reflected in the OH levels derived from eq 19 until AMP is close to depletion, at which time the OH level was assumed to be constant in the chamber chemistry model until the chamber canopy was closed.

The theoretical study advises that H abstraction from the CH_2_ group accounts for the major AMP reactivity, *B*_CH_2__ > 0.7, and that the resulting primary product, (CH_3_)_2_(CHO)CNH_2_, should be at least twice as reactive toward OH radicals as AMP. The, by far, most dominant product in the (CH_3_)_2_(CHO)CNH_2_ + OH reaction, (CH_3_)_2_C=NH, is expected to react relatively fast with OH, but most likely not as fast as the corresponding alkene (*k*_OH+(CH_3_)_2_C=CH_2__ = 5.1 × 10^–11^ cm^3^ molecule^–1^ s^–1^).^53^ The two dominant products can be reasonably reproduced with *B*_CH_2__ = 0.70 ± 0.07, *k*_OH+(CH_3_)_2_(CHO)CNH_2__ = 7.0 × 10^–11^, and *k*_OH+(CH_3_)_2_C=NH_ = 2.0 × 10^–11^ cm^3^ molecule^–1^ s^–1^.

H abstraction from the CH_3_ groups in AMP is predicted as a minor route, *B*_CH_3__ < 0.10. To reproduce the (CH_3_)(CH_2_OH)C=NH time profiles requires a branching *B*_CH_3__ = 0.06 ± 0.01 and a (CH_3_)(CH_2_OH)C=NH reactivity toward OH of the same magnitude as that of (CH_3_)_2_C=NH.

Concerning H abstraction from the NH_2_ group in AMP, there are two critical issues in modeling the subsequent gas-phase reactions in the chamber. The first is related to AMPNO_2_ being the only unique product to this route, that there is a significant time delay in the AMPNO_2_ detector signal, and that it is not possible to exclude that heterogeneous surface reactions on particles and on the walls of the sampling lines contribute to its apparent continuous increase in the chamber experiments. The nitrosamine, AMPNO, was not detected in any of the experiments, and the third product following NH abstraction, (CH_3_)_2_C=NH, is also the major product in the (CH_3_)_2_(CHO)CNH_2_ + OH reaction, see above. The other critical issue is related to the rate coefficients of the competing (CH_3_)_2_(CH_2_OH)CṄH radical reactions: (i) the thermal dissociation of the (CH_3_)_2_(CH_2_OH)CṄH radical is calculated from first principles and may be a factor of 5 off, and (ii) the rate coefficients for the (CH_3_)_2_(CH_2_OH)CṄH radical reactions with NO and NO_2_ are assumed to be the same as those for the (CH_3_)_2_Ṅ radical^43^ and may conceivably also be a factor of 5 off. As the branching in the (CH_3_)_2_(CH_2_OH)CṄH radical reactions is modeled employing the steady-state approximation, the rate coefficient for the thermal dissociation of (CH_3_)_2_(CH_2_OH)CṄH was simply scaled to optimize the agreement between the observed and modeled nitramine formation in the experiments. The best overall agreement was obtained with a scaling factor of 0.2, which is well within the limit set by the estimated uncertainties in the reaction rate coefficients. It should be noted that there is an uncertainty of 5–10% in the NO and NO_2_ monitored values employed in the modeling (see the Supporting Information) and that this uncertainty translates approximately linearly to the modeled nitrosamine and nitramine yields.

**Figure 9:**
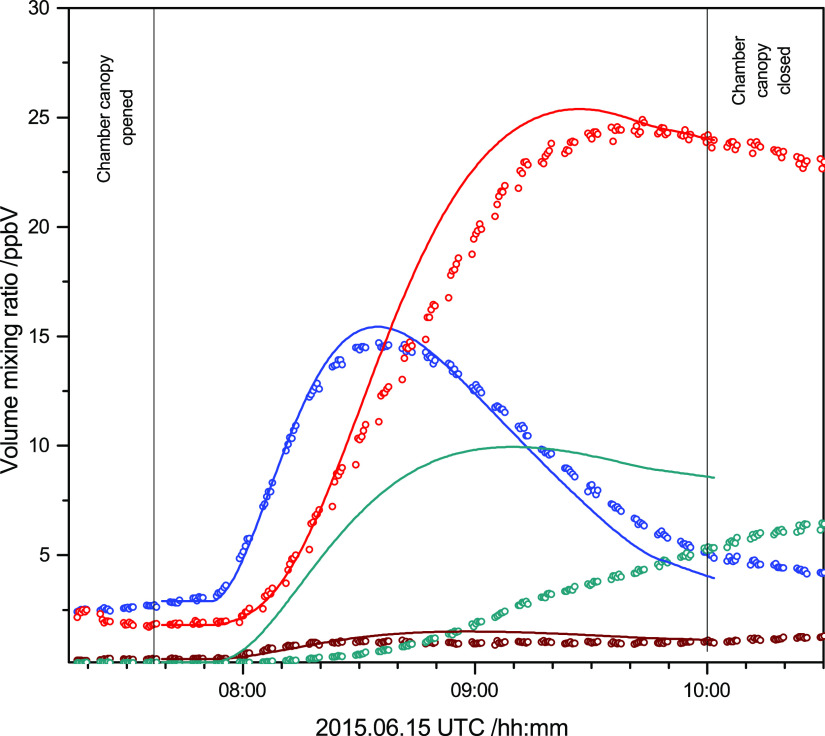
Observed and modeled temporal profiles of products in the OH-initiated AMP photo-oxidation experiment on 2015.06.15. (CH_3_)_2_(CHO)CNH_2_ (blue color), (CH_3_)_2_C=NH (red color), CH_3_(CH_2_OH)C=NH (wine color), and (CH_3_)_2_(CH_2_OH)NHNO_2_ (dark cyan color).

Restating, the PTR-MS response was calibrated with respect to CH_2_O, AMP, and AMPNO_2_, whereas theoretically derived instrumental response factors, as summarized in Table S1, were used for other compounds. The uncertainties in the derived volume mixing ratios are estimated to be better than ±10% for the calibrated compounds and ±25% for other compounds, provided that extensive fragmentation does not take place in the PTR instruments [i.e., the main AMP photo-oxidation product, (CH_3_)_2_(CHO)CNH_2_]. The product distribution was modeled employing the branching ratios *B*_CH_3__/*B*_CH_2__/*B*_NH_2__ = 6:70:24, and the rate coefficients collected in Table 2. Figure 9 shows the observed and modeled mixing ratio time profiles of AMP and the primary photo-oxidation products. The results from the analyses of the other experiments are documented in Figures S47–S51.

## 4 Conclusions

To the best of our knowledge, there are no natural emissions of AMP to the atmosphere. Minor anthropogenic emissions may arise from the use of consumer products containing AMP,^1^ but the implementation of large-scale CO_2_ capture facilities employing AMP-containing solvents may likely result in emissions of a different measure. Once in the atmospheric compartment, AMP partitions between the gas phase and the aerosol phase and undergoes photo-oxidation; AMP may also form new particles in homogeneous nucleation with various acidic atmospheric constituents. The competition between the gas-phase removal processes mentioned strongly depends on the local conditions.

The atmospheric lifetime of AMP with respect to the gas-phase reaction with OH during daytime, τ_OH_, is typically ∼10 h (*k*_OH+AMP_ ≈ 2.8 × 10^–11^ cm^3^ molecule^–1^ s^–1^).^10^ The night-time chemistry of AMP is likely dominated by the NO_3_ radical and with an assumed average night-time NO_3_ concentration around 5 × 10^8^ cm^–3^,^57,67^ τ_NO_3__ for AMP is ∼15 h (*k*_NO3+AMP_ ≈ 3.7 × 10^–14^ cm^3^ molecule^–1^ s^–1^ at 298 K, see Section 3.2.1).

Considering the uptake coefficients for methylamines on 59–82 wt % sulfuric acid (γ ∼ 2 × 10^–2^)^68^ as the expected level for the amine uptake on particles in general, the aqueous particle uptake of AMP is diffusion-controlled under atmospheric conditions. The Henry volatility of AMP was reported to be *K*_H_^p*x*^ = 258 Pa at 40 C (Henry’s law solubility, *H*^cp^ = 215 mol m^–3^ Pa^–1^).^69^ Under non-reactive equilibrium conditions and assuming the liquid water content in clouds, fog, and urban aerosol to be, respectively, 3, 0.2, and 10^–4^ cm^3^ m^–3^,^70^ AMP partitions roughly 60, 10, and ≪1% to the aqueous particle phase in the three cases. However, urban clouds, fog, and deliquescent particles are in general acidic, and the AMP partitioning will therefore shift additionally toward the aqueous phase.

There are no experimental rate coefficients for AMP reactions in the aqueous phase; the group contribution method by Minakata et al.^71^ predicts *k*_OH,aq_ = 5.6 × 10^9^ M^–1^ s^–1^. Assuming [OH]_av_ = 3.5 × 10^–15^ in urban clouds and 4.4 × 10^–13^ in urban deliquescent particles,^6^ the estimated lifetime of AMP in clouds is around 14 h but only 7 min in deliquescent urban particles. The high reactivity in the deliquescent particle phase consequently drives the additional uptake to the aerosol, and a significant amount of AMP may actually be oxidized in deliquescent particles. There are no experimental results from the mechanistic studies of aqueous-phase AMP reactions and only speculations on the possible aqueous-phase degradation of AMP have been offered.^72^ It should be noted that AMP is in its protonated form in the urban clouds, fog, and deliquescent particles. This reduces H abstraction at the protonated amino group significantly,^73−75^ diminishing the possible formation of AMPNO and AMPNO_2_. The former will in any case hydrolyze directly to 2-methylpropane-1,2-diol.

The major product in atmospheric AMP photo-oxidation, (CH_3_)_2_(CHO)CNH_2_, is found to react ∼2 times faster than AMP with OH radicals resulting primarily in (CH_3_)_2_C=NH. In turn, (CH_3_)_2_C=NH and the other primary imine product, CH_3_(CH_2_OH)C=NH are found to react slightly slower with OH radicals than AMP, and their major atmospheric sink is therefore expected to by hydrolysis on aqueous particles resulting in acetone, hydroxyacetone, and ammonia. Regarding the photo-oxidation products with respect to health concerns, AMPNO and AMPNO_2_, the former will never build up in the atmosphere due to very fast photolysis and very fast reaction with OH, leading to nitrous oxide with a yield of <0.1% under atmospheric conditions. Should AMPNO transfer to the atmospheric aqueous phase, it will hydrolyze to CH_3_C(OH)(CH_3_)CH_2_OH. AMPNO_2_ is expected to react nearly equally as fast with OH as AMP, that is, the atmospheric lifetime with respect to gas-phase photo-oxidation is estimated to be around 10 h. There are no data for the Henry’s law solubility constants for nitramines but to a first approximation they are expected to be the same as those of the nitrosamines, which is 3–10 times larger than the corresponding amine.^76^ Consequently, a large fraction of AMPNO_2_ is expected to transfer to the atmospheric aqueous phase and undergo at least some processing there before surface deposition.

The strong particle growth observed during AMP photo-oxidation experiments suggests that new particle formation may constitute an important gas-phase removal process for AMP under atmospheric conditions. The VTDMA studies support this by revealing a low vapor pressure of the AMP nitric acid salt, *p*^0^ = (1.3 ± 0.3) ×10^–5^ Pa at 298 K. There is only one other alkanolamine nitrate for which similar experimental data are available; the vapor pressure of the MEA nitric acid salt is reported to be around 5 times higher, *p*^0^ = (9.0 ± 0.4) × 10^–5^ Pa at 298 K,^77^ which in part can be rationalized by the difference in basicity (p*K*_b,MEA_ = 4.56; p*K*_b,AMP_ = 4.32).^78^ Inferring from the experimental vapor pressures of MEA nitrate^77^ and other MEA salts including the sulfate,^79^ it is obvious that AMP has a large potential to form new particles.

A detailed atmospheric gas-phase chemistry model for the OH-initiated photo-oxidation of AMP was developed by combining the results from quantum chemistry-based theoretical calculations and photo-oxidation experiments carried out in a large atmospheric simulation chamber. The best agreement with the experimental data was obtained with ∼70% H abstraction from the −CH_2_– group, ∼6% H abstraction from −CH_3_ groups, and ∼24% H abstraction from the −NH_2_ group.

Given that both new particle formation and phase transfer to aqueous and deliquescent particles are important atmospheric loss processes for AMP, a computationally costly 3D chemistry transport multiphase model is required to describe the atmospheric fate of AMP appropriately. Realizing that the atmospheric aqueous phase constitutes either a time delay of AMP degradation or an irreversible sink allows a worst-case scenario calculation to be based on pure gas-phase chemistry. A simple comparison between results from the application of atmospheric photo-oxidation models for AMP and MEA^80^ shows that the amount of carcinogens formed during a given time span is around 4 times lower for AMP, which primarily is due to the OH reaction being 3 times slower.

## Supporting Information Available

Details on instrumentation and methodologies including chemical synthesis, atmospheric chemistry of AMP from first principles, AMP photo-oxidation study, and particle formation and analysis (PDF)
