# Supplementary material for: Atmospheric Chemistry of 2-Amino-2-methyl-1-propanol: A Theoretical and Experimental Study of the OH-Initiated Degradation under Simulated Atmospheric Conditions
Source: J Phys Chem A. 2021 Aug 23;125(34):7502–19. doi: 10.1021/acs.jpca.1c04898 (PMC8419843; doi:10.1021/acs.jpca.1c04898)
Supplement: Supplementary file 1 — jp1c04898_si_001.pdf [file jp1c04898_si_001.pdf]

Atmospheric Chemistry of 2-Amino-2-Methyl-1-Propanol – A Theoretical and Experimental Study of the OH-Initiated Degradation under Simulated Atmospheric Conditions

Supporting Information

Wen Tan,[1] Liang Zhu,[1] Tomáš Mikoviny,[1] Claus J. Nielsen,[1] Yizhen Tang,[1] Armin Wisthaler,[1] Philipp Eichler,[2] Markus Müller,[2] Barbara D′Anna,[3] Naomi J. Farren,[4] Jacqueline F. Hamilton,[4] Jan B. C. Pettersson,[5] Mattias Hallquist,[5] Simen Antonsen[6] and Yngve Stenstrøm[6]*

[1] Section for Environmental Sciences, Department of Chemistry, University of Oslo, P.O.Box. 1033 Blindern, NO-0315 Oslo, Norway.

[2] Institute of Ion Physics and Applied Physics, University of Innsbruck, 6020 Innsbruck, Austria

[3] Aix Marseille Univ, CNRS, LCE, UMR 7376, 13331, Marseille, France

[4] Wolfson Atmospheric Chemistry Laboratories, Department of Chemistry, University of York, York, YO10 5DD, United Kingdom.

[5] University of Gothenburg, Department of Chemistry and Molecular Biology, Atmospheric Science, 41296 Gothenburg, Sweden

[7] Faculty of Chemistry, Biotechnology and Food Science, Norwegian University of Life Sciences, P.O. Box 5003, N-1432 Ås, Norway.

INSTRUMENTATION AND METHODOLOGIES 4
Table S1 5
Chemical synthesis 8
Figure S1 8
Figure S2 9
Figure S3 9
Figure S4 10
ATMOSPHERIC PHOTO-OXIDATION OF AMP FROM FIRST PRINCIPLES 11
Figure S5 11
Figure S6 12
Figure S7 12
Scheme S1 13
Figure S8 16
Figure S9 17
Figure S10 18
Figure S11 20
Scheme S2 21
Figure S12 23
Figure S13 24
Figure S14 25
Scheme S3 26
Figure S15 27
Figure S16 28
Scheme S4 29
Table S2 30
Table S3 44
Table S4 45
Table S5 51
Table S6 55
Table S7 65
Table S8 69
Table S9 78
Table S10 81
Table S11 86
Table S12 89
Figure S17 93
Table S13 93
Table S14 96
EXPERIMENTAL RESULTS 97
Table S15 97
Figure S18 98
Figure S19 99
Figure S20 100
Figure S21 101
Figure S22 102
Figure S23 103
Figure S24 104
Figure S25 105
Figure S26 105
Figure S27 106
Figure S28 106
Figure S29 107

Figure S30 107
Figure S31 108
Figure S32 109
Figure S33 110
Figure S34 111
Figure S35 112
Scheme S5 113
Table S16 114
Figure S36 114
Figure S37 115
Figure S38 115
Figure S39 116
Figure S40 116
Figure S41 117
Figure S42 117
Figure S43 118
Figure S44 118
Figure S45 119
Figure S46 119
Table S17 120
Figure S47 120
Figure S48 121
Figure S49 121
Figure S50 122
Figure S51 122

REFERENCES 123

INSTRUMENTATION AND METHODOLOGIES

European Photoreactor (EUPHORE).

A series of experiments was carried out in chamber B of the EUPHORE facility in Valencia, Spain. The 200 m^3 polytetrafluoroethylene (PTFE) atmosphere simulation chamber has been described in detail elsewhere[1] and only the details pertinent to this work are given here. A syringe pump was used to inject known amounts of AMP into the chamber via a heated (120 °C) transfer line made of passivated stainless steel. The line was flushed with nitrogen during and after injection. Nitric oxide (NO) was injected using a gas-tight syringe. A basic scrubber was inserted into the injection line for removing trace nitric acid. Nitrogen dioxide (NO_2) was generated from NO via addition of ozone (O_3). After opening the chamber canopy, isopropyl nitrite (IPN) was continuously added to the chamber in a flow of nitrogen as an efficient OH radical precursor. Acetonitrile was used as a virtually inert dilution tracer in the kinetic study. The EUPHORE facility is equipped with standard monitors for pressure (p), temperature (T), relative humidity, NO, NO_2, O_3, NO_2 photolysis frequency (j_{NO2}) and sub-micrometer particle size distribution. The latter was measured using a Scanning Mobility Particle Sizer (SMPS) consisting of an electrostatic classifier (Model 3080, impactor: 0.0457 cm) with a long differential mobility analyzer (DMA, Model 3081), and a condensation particle counter (CPC, Model 3022A). For the experiments described herein, we deployed a series of additional analyzers/analytical methods, which are described in more detail below.

Fourier Transform Infrared Spectroscopy (FT-IR).

The EUPHORE Chamber B is equipped with a Nicolet 6700 FTIR spectrometer coupled with White multi-reflection mirror system for in situ analysis adjusted to give an optical path length of 553.5 m. FTIR spectra were recorded every ten minutes by co-adding 370 interferograms with a resolution of 0.25 cm^{-1}. Boxcar apodization was used in the Fourier transformation. The interferograms were saved for possible later re-processing.

Retrieval of trace gas volume mixing ratios was carried out semi-automatically employing the MALT program.[2] This method simulates the spectrum of the mixture of absorbing species from a set of initial concentrations and reference spectra and then varies the concentrations iteratively to minimize the residual between the measured and simulated spectrum. For spectrum calculation, true absorption coefficients were used if available. In the absence of true absorption coefficients, high-resolution spectra can be used as a good approximation. The spectral data needed in the fitting procedure were taken from the HITRAN 2008 database (H_2O, CO, NO, NO_2, CO_2, CH_4).[3] Retrieval of chemical components from the FT-IR spectra is sensitive to detector non-linearity and interference of compounds with overlapping spectra. For compounds whose spectra only show *PQR*-structure the retrieval procedure has an estimated uncertainty of ±20 %. For compounds showing rotational fine structure the retrieval procedure has an estimated uncertainty of ±5 %. NO and NO_2 values, used for inter-calibration of the NOx-monitors, were obtained from analyses of the 1898.5–1914.0 and 1589.9–1614.0 cm^{-1} regions, respectively.

Proton-Transfer-Reaction Time-of-Flight Mass Spectrometry (PTR-ToF-MS).

A PTR-TOF 8000 instrument (Ionicon Analytik GmbH, Innsbruck, Austria) was used for measuring PZ and its photochemical oxidation products in the gas phase. AMP and its photochemical oxidation products in the gas phase. Detailed description to chamber facility, sample injection system into the chamber, operation conditions of both PTR-TOF 8000 for online gaseous and aerosol analysis, calibration procedures has been reported elsewhere [4] and thus only the details pertinent to this study are described here. The drift tube was kept at a temperature of 100 °C and a pressure of 2.30 mbar. The electric field applied to the drift tube was periodically switched in 80 s intervals, i.e. measurements were performed at alternating E/N-values of 60 and 105 Td (1 Td = 10^{-17} V cm^{-2} $molecule^{-1}$), respectively. The PTR-TOF 8000 instrument was interfaced to the chamber using Siltek®/Sulfinert®-treated stainless steel tubing (total length: 143 cm, 60 cm extending into the chamber, ID: 4.57 mm, temperature: 100 °C, flow: 20 lpm). The flow to the

instrument was subsampled through PEEK® (polyetheretherketone) capillary tubing (OD: 1.59 mm). The instrument was regularly calibrated against a gaseous reference standard containing 13 hydrocarbons and oxygenated hydrocarbons, to gain response factors of representative over mass range between 30 and 150 Th, therefore determining the instrumental mass discrimination function. Additionally, PTR-TOF 8000 for gas analysis was further calibrated for AMP and AMP-nitramine using a liquid calibration unit (LCU; Ionicon Analytik GmbH, Innsbruck, Austria) for evaporation of gravimetrically prepared aqueous standards in nitrogen. The fragmentation pattern of 1-nitroso-piperazine was taken from measurement of 1-nitroso-piperazine in the chamber prior to canopy opening. The calibration factors of other photochemical byproducts were derived relying on experimental mass discrimination function and calculated reaction rate coefficient (Table S1), since in PTRMS ionization happens at the collisional rate.[5]

High-Temperature Proton-Transfer-Reaction Mass Spectrometer (HT-PTR-MS).

In addition to a conventional PTR-ToF-MS instrument, a custom-built HT-PTR-MS instrument[6] was used for on-line monitoring of organic trace gases. In this instrument, surface losses and memory effects are reduced due to elevated temperatures (160 °C) of the inlet orifice and the drift tube. Previous work[7] has shown that this instrument is well-suited for kinetic measurements of "sticky" reagent gases. The HT-PTR-MS instrument is, however, not capable of resolving isobaric ions with the same nominal *m/z* value. The instrument was interfaced to the EUPHORE chamber using Siltek®/Sulfinert®-treated stainless steel tubing (total length: 125 cm, 50 cm extending into the chamber, ID: 4.57 mm) which was connected to the 35 µm Siltek®/Sulfinert®-treated inlet orifice via a Siltek®/Sulfinert®-treated T-piece. Inlet temperature and flow were kept at 100 °C and 20 lpm, respectively. The instrument was operated at 95 Td in a 12 s cycle mode during which 1-second integration was carried out on selected m/z. Calibration procedures were the same as for the PTR-TOF 8000 instrument.

Chemical Analysis of Aerosol Online (CHARON)

A prototype CHARON inlet [8,9] was interfaced to a second PTR-TOF 8000 instrument (Ionicon Analytik GmbH, Innsbruck, Austria) for measuring particle chemical composition. The CHARON inlet strips off gas-phase analytes, enriches the particle concentration in the PTR-ToF-MS subsampling flow and vaporizes the particles prior to ionization and mass spectrometric analysis. In this study, the vaporization temperature was set to 140 °C. The CHARON inlet was interfaced to the EUPHORE chamber using Siltek®/Sulfinert®-treated stainless steel tubing (total length: 415 cm, 40 cm extending into the chamber, ID: 4.57 mm). The PTR-ToF-MS drift tube was kept at a voltage of 350 V, a temperature of 130 °C and a pressure of 2.40 mbar (100 Td). The instrument was regularly calibrated against a gaseous reference standard containing 13 pure and oxygenated hydrocarbons. A reference mass spectrum was obtained from the AMP-aminium nitrate salt.

Table S1 Dipole moments and isotropic polarizabilities from B3LYP/aug-cc-pVTZ calculations and derived collisional rate coefficients, $k_{H_3O^+\text{-analyte molecule}}$(100 °C), used for determining the PTR-ToF-MS response factors.

	Method	Dipole moment	Polarizability	k /10^{-9} cm^3 molecule^{-1} s^{-1}	
		D	Å^3	*E/N* 65	*E/N* 105
$NH_2C(CH_3)_2CH_2OH$	B3LYP	2.95	9.92	3.573	3.212
	M06-2X	2.94	9.57	3.540	3.323
$NH_2C(CH_3)_2CHO$	B3LYP	3.00	9.50	3.585	3.366
	M06-2X	2.96	9.13	3.529	3.313
$ON\text{-}NHC(CH_3)_2CH_2OH$	B3LYP	4.25	10.07	4.445	4.093
	M06-2X	4.08	11.40	4.397	4.083
$O_2N\text{-}NHC(CH_3)_2CH_2OH$	B3LYP	4.61	12.32	4.802	4.428
	M06-2X	4.42	11.88	4.635	4.283
$NH{=}C(CH_3)_2$	B3LYP	2.49	7.15	3.181	2.999

	M06-2X	2.50	6.92	3.168	2.986
$NH=(CH_3)CH_2OH$	B3LYP	3.76	7.74	4.113	3.790
	M06-2X	3.75	7.46	4.085	3.759
$CHOC(CH_3)(NH_2)CH_2OH$	B3LYP	3.65	10.06	4.041	3.766
	M06-2X	3.66	9.68	4.023	3.744
$CH_3C(O)NH_2$	B3LYP	3.89	5.93	4.201	3.824
	M06-2X	3.92	5.68	4.205	3.821
$CHONH_2$	B3LYP	3.95	4.19	4.275	3.860
	M06-2X	3.97	3.99	4.270	3.851
$CH_3C(O)CH_3$	B3LYP	3.08	6.34	4.383	3.998
	M06-2X	3.10	6.11	4.389	3.995
CH_3CN	B3LYP	4.04	4.42	4.450	4.023
	M06-2X	4.05	4.30	4.446	4.017
$CH_3C(O)CH_2OH$	B3LYP	3.27	6.96	3.659	3.395
	M06-2X	3.25	6.79	3.630	3.367
$NH=C(CH_3)CHO$	B3LYP	2.81	7.25	3.343	3.137
	M06-2X	2.87	6.99	3.367	3.153
$O_2N\text{-}NHC(CH_3)_2CHO$	B3LYP	4.72	11.87	4.865	4.470
	M06-2X	4.68	11.42	4.810	4.416
$CH_2(O)C(CH_3)_2$	B3LYP	2.05	7.93	2.851	2.640
	M06-2X	2.02	7.69	2.807	2.599

Aerosol Mass Spectrometry

A compact time-of-flight Aerosol Mass Spectrometer (C-ToF-AMS, Aerodyne Research Inc., Billerica, MA, U.S.A.) [10] was used for measuring total particle mass loading and particle chemical composition. A reference spectrum was obtained from the piperazinium nitrate salt. The cToF-AMS data set was processed using a cumulative peak fitting analysis which allows to separate multiple isobaric peaks that are not taken into account in the traditional analysis of unit mass resolution data. [11] Uncertainties in the major chemical species from the cToF-AMS are typically of the order of ±30%. [10]

Aerosol filter sample collection and extraction

Aerosol formed in the chamber was collected onto pre-baked (550 °C, 6 h) 47 mm diameter quartz microfibre filters (Whatman, Maidstone, U.K.) at the end of each experiment using a high-volume sampling pump (flow rate: 48 L min^{-1}, sampling time: 60 min, total volume sampled: 2.88 m^3). After collection the filter samples were stored at -18 °C until analysis. Three-quarters of each filter was extracted into EtOAc using an accelerated solvent extraction (ASE) system, prior to evaporation to 1 mL under a gentle stream of nitrogen. Full details of the ASE method can be found elsewhere.[12] The extracts were analysed using two-dimension gas chromatography - nitrogen chemiluminescence detection (GCxGC-NCD). The remaining quarter of each filter was dissolved in 2 mL milli-Q water (18 MΩ cm^{-1}) and extracted by sonication (30 min). The extracts were analysed using ion chromatography (IC) to detect nitrate (NO_3^-) and protonated AMP (AMP^+).

Two-dimensional gas chromatography – nitrogen chemiluminescence detection (GC×GC-NCD)

Chromatographic analysis was carried out on a GC×GC-NCD system comprised of an Agilent 7890 gas chromatograph and an Agilent 255 NCD system (Palo Alto, CA, United States). The first column was a non-polar Ultra Inert DB5 (30 m × 0.32 mm i.d. × 0.25 µm film thickness) and the second column a mid-polarity DB-17 (3 m × 0.10 m i.d. × 0.10 µm film thickness). Both columns were purchased from Agilent Technologies Ltd., Stockport, U.K. The initial temperature of the first dimension column was 40 °C for 2 min, followed by a heating rate of 7 °C min^{-1} to 100 °C for 8 min and then further heating at 7 °C

min^{-1} until 270 °C was reached and held isothermally for a further 5 min. A temperature offset of 30 °C was applied to the second-dimension column throughout the GC temperature program. A liquid nitrogen two-stage cold jet modulation system was used, with a modulation period of 5 s and a +15 °C offset from the secondary GC oven temperature. Data was collected at 200 Hz over the entire course of the analysis, and hydrogen was used as a carrier gas at 1.4 mL min^{-1}. Injections of 1 µL were performed in splitless mode at an injection temperature of 200 °C using an automated liquid injector (Gerstel, Mülheim an der Ruhr, Germany). Pyrolysis of the analytes in the dual plasma burner was carried out at 900 °C under a hydrogen flow rate of 4 mL min^{-1} and an oxygen flow rate of 10 mL min^{-1}. Recovery of the AMP nitramine was calculated by comparing the peak areas obtained from spiked filters with peak areas acquired from the standard directly injected to the GC×GC-NCD. The recovery tests were performed in triplicate and the average recovery level was 81.8% (RSD = 3.2%).

Ion chromatography (IC).

Chromatographic analysis was carried out using an ICS-110 integrated IC system equipped with an AS-DV autosampler (Dionex Corporation, CA, United States). The column configuration used for anion exchange consisted of an IonPac AG14A guard column (4 × 50 mm) and an IonPac AS14A analytical column (4 × 250 mm). Cation exchange chromatography was performed using an IonPac CG12A guard column (4 × 50 mm) and an IonPac CS12A analytical column (4 × 250 mm). AERS 500 and CSRS 300 self-regenerating suppressors (4 mm) were used for anion and cation exchange respectively. The IC was operated in isocratic mode, with a flow rate of 1 mL min^{-1} and suppressor current of 45 and 59 mA for anion and cation exchange respectively. A solution of 8 mM Na_2CO_3 /1 mM $NaHCO_3$ was used as the eluent for anion-exchange chromatography and a 20 mM solution of methane sulfonic acid was used as the eluent for cation-exchange chromatography. Recoveries of AMP^+ and NO_3^- were calculated by comparing the peak areas obtained from spiked filters with peak areas acquired from standard directly injected to the IC. The recovery tests were performed in triplicate and average recovery levels of 75.2% (RSD = 1.2%) and 98.8% (RSD = 0.3%) were achieved for AMP^+ and NO_3^- respectively.

Volatility tandem differential mobility analyzer (VTDMA)

The VTDMA is described by Jonsson et al 2007[13] while the method to extract thermal properties can be found in Salo et al, 2010.[14] The VTDMA was used to characterise the nitrate salt of AMP. A solution of the salt was nebulised and resulting aerosol particles were dried before entering the VTDMA unit. The median residence time in the oven unit for the particles in the selected size (ca 100 nm) was 2 s at 298 K and evaporation occurred under non-equilibrated conditions, i.e. evaporation dominates during the residence time and there is limited condensation. The following equation was used to derive the saturation vapour pressure (p^0) of compound i (in this case AMP-nitrate) at evaporative temperature T:

$$\frac{\rho_i RT}{4D_{i,air}\Delta t M_i}\int_{D_{p,i}}^{D_{p,f}}\frac{D_p}{f(Kn_i,\alpha)}\exp\left(\frac{-4\gamma_i M_i}{D_p\rho_i RT}\right)dD_p$$

Where ρ_i is the particle density, R is the gas constant, T is the temperature, $D_{i,air}$ is the diffusivity of molecule i in air which can be calculated with parameters σ_{ii} and ε_{ii}, Δt is the median residence time in the oven calculated by assuming laminar flow, M_i is the molar mass. In the second term $D_{p,i}$ is the initial and $D_{p,f}$ is the final particle diameter, $f(Kn_i, \alpha)$ is a correction term for particle diameters in the transition regime calculated using the Knudsen number (Kn_i) and the accommodation coefficient (α) In the third term γ_i is the surface free energy. Most of the physical data are unknown for AMP-nitrate so they were assumed to be similar to trimethylaminium nitrate as described by Salo et al, 2011.[15] Further details on how to derive the vapour pressures from these data and associated uncertainties are described in Salo et al. 2010.[14] It should be noted that the applied analysis is assuming evaporation of a liquid and not from a crystalline phase and the actual phase of the aerosols can be either liquid or crystalline. Another uncertainty is the

assumption on surface free energy. Here we assumed 0.1 J m^{-2} that seems reasonable from the relative few literature data that are available. In addition, an uncertainty analysis was also done in that previous study. On the basis of experimental conditions and the input parameters to the equation given above, systematic errors of around ±20 % are inferred.

Chemical synthesis

2-Methyl-2-(nitroamino)-1-propanol

The AMP-nitramine was prepared as described by Antonsen et al.[16] A solution of 4,4-dimethyloxazolidin-2-one (13,2g, 115 mmol) in 72 mL Ac_2O was added slowly with stirring to a cooled (5–10 ◦C) suspension of finely-ground $Cu(NO_3)_2{\cdot}(H_2O)_3$ (10.5 g; 43 mmol) in 85 mL Ac_2O. After 1 h, an additional portion of finely-ground $Cu(NO_3)_2{\cdot}(H_2O)_3$ (10.5 g; 43 mmol) was added. The resulting mixture was cooled to 0–5 °C (ice/water) and stirred for another 4 h while slowly being heated to room temperature. The mixture was filtered through Celite 545. Water (50 mL) was added, and the water phase was extracted with DCM (3 × 50 mL) to get rid of additional dissolved copper salts. Drying (MgSO4) and evaporation gave a crystalline compound that was recrystallized from toluene to give the product as off-white crystals. m.p. 121–122 °C.[17] ^{1}H-NMR (400 MHz, $CDCl_3$): δ 4.11 (2H, s), 1.69 (6H, s). ^{13}C-NMR (100 MHz, $CDCl_3$): δ 23.08, 62.89, 73.17, 147.64. IR: 3005, 2992, 2949, 1772, 1565 cm^{-1}.

The crystals were dissolved in H_2O and heated to 35°C for 2 hours to give the AMP nitramines in quantitative yield. ^{1}H-NMR (400 MHz, D_2O): δ 3.32 (2H, s), 0.98 (6H, s). ^{13}C-NMR (100 MHz, D_2O): δ 20.06, 20.14, 59.14, 68.42.

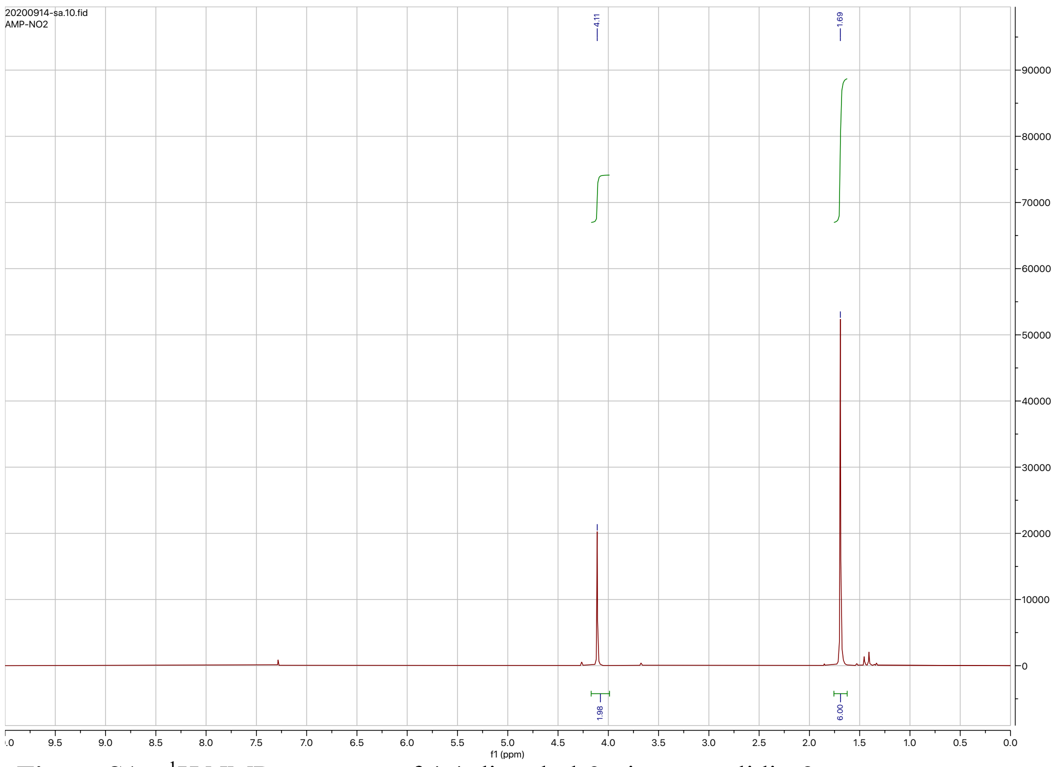

Figure S1 ^{1}H NMR spectrum of 4,4-dimethyl-3-nitrooxazolidin-2-one.

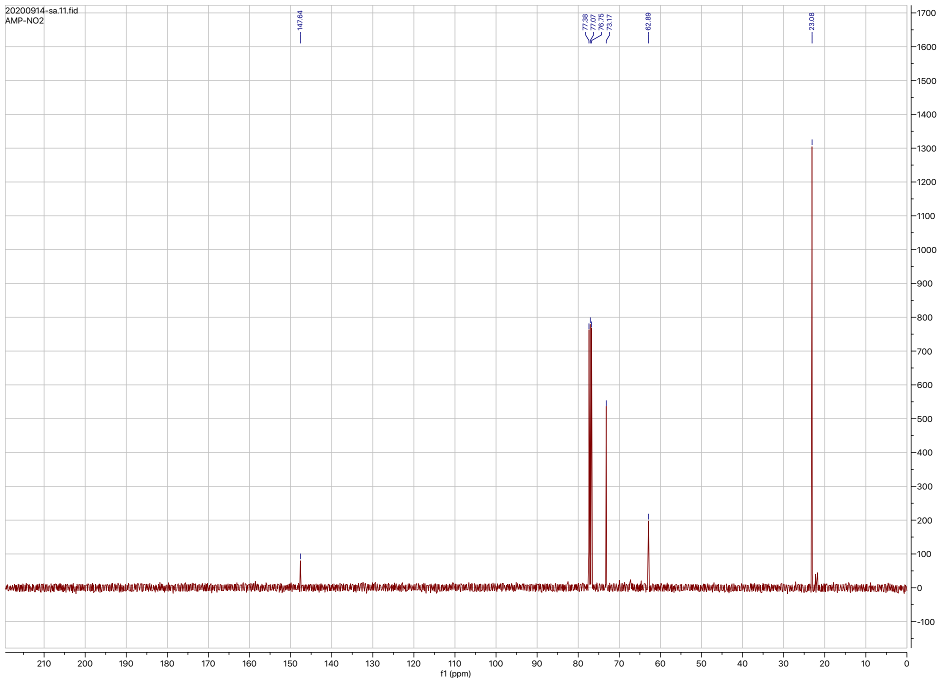

Figure S2 ^{13}C NMR spectrum of 4,4-dimethyl-3-nitrooxazolidin-2-one.

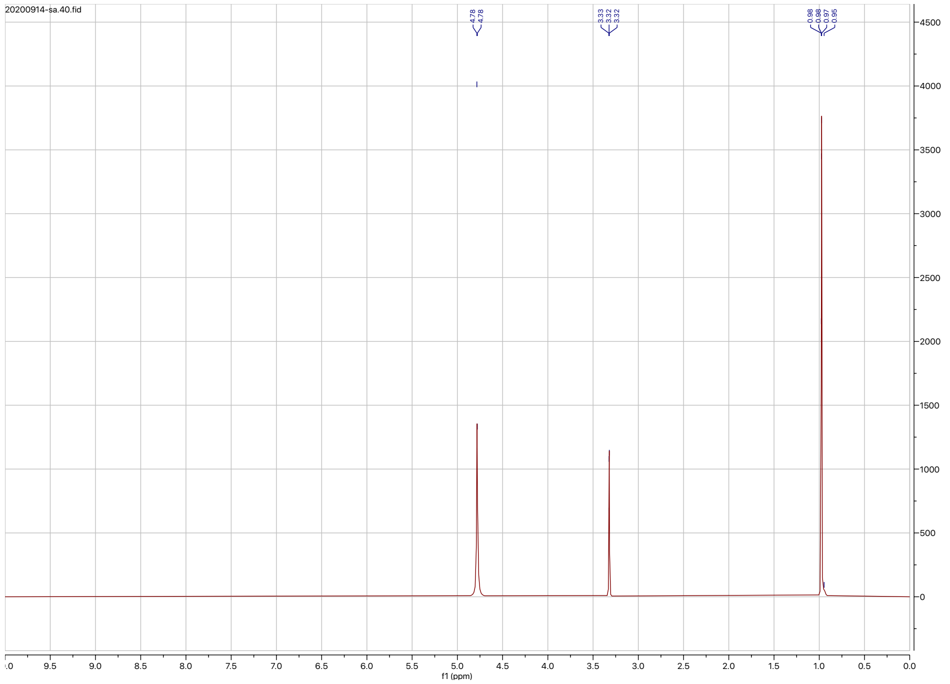

Figure S3 ^{1}H NMR spectrum of 2-Methyl-2-(nitroamino)-1-propanol.

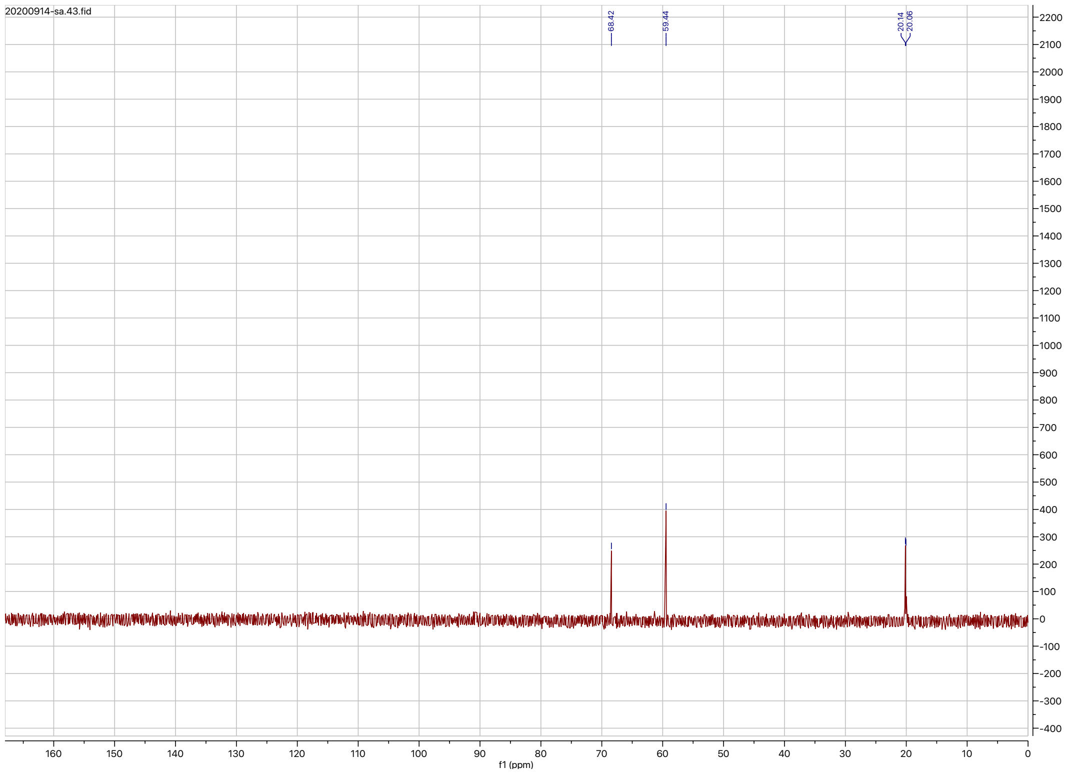

Figure S4 ^{13}C NMR spectrum of 2-Methyl-2-(nitroamino)-1-propanol.

ATMOSPHERIC PHOTO-OXIDATION OF AMP FROM FIRST PRINCIPLES

The AMP reaction with OH radicals proceeds via H-abstraction from either one of the $–CH_3$ groups, from the $–CH_2OH$ group, or from the $–NH_2$ group. In the order of decreasing reaction enthalpies (298 K, results from G4 calculations [18]), the four possible routes in the AMP + OH reaction are:

$\Delta H^{\Theta} = -105$	$(CH_3)_2C(NH_2)CH_2OH + OH \rightarrow$	$(CH_3)_2C(NH_2)\dot{C}HOH + H_2O$	(1a)
$\Delta H^{\Theta} = -78$	$\rightarrow$	$(CH_3)_2C(CH_2OH)C\dot{N}H + H_2O$	(1b)
$\Delta H^{\Theta} = -71$	$\rightarrow$	$\dot{C}H_2C(CH_3)(NH_2)CH_2OH + H_2O$	(1c)
$\Delta H^{\Theta} = -56$	$\rightarrow$	$(CH_3)_2C(NH_2)CH_2\dot{O} + H_2O$	(1d)

AMP has 11 non-equivalent hydrogen atoms. Figures S1-S3 illustrate the stationary points on the potential energy surfaces (PES) of the routes – energies, Cartesian coordinates and vibration-rotation data for all stationary points on the PES's of reaction 1 are collected in Table S2.

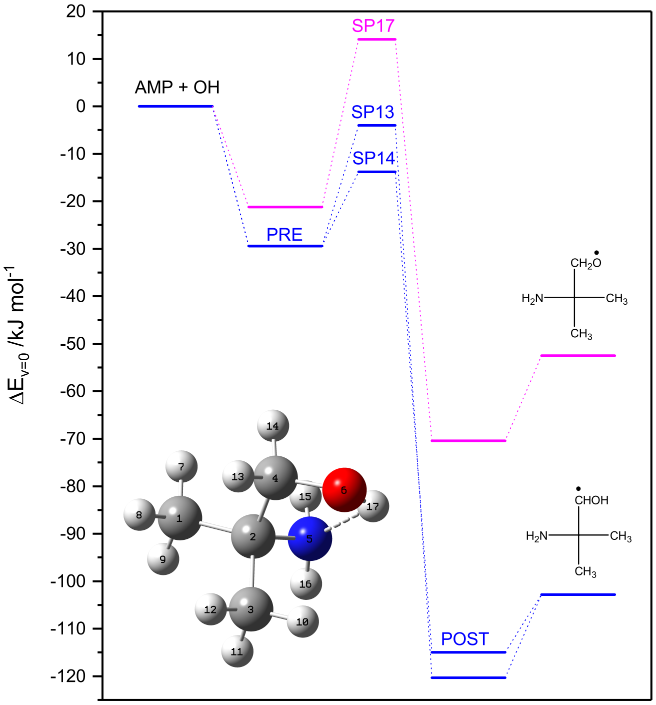

Figure S5 Relative energies of stationary points on the potential energy surface of relevance to H-abstraction by OH radicals from the CH_2OH-group in $(CH_3)_2(CH_2OH)CNH_2$. Results from M06-2X/aug-cc-pVTZ calculations.

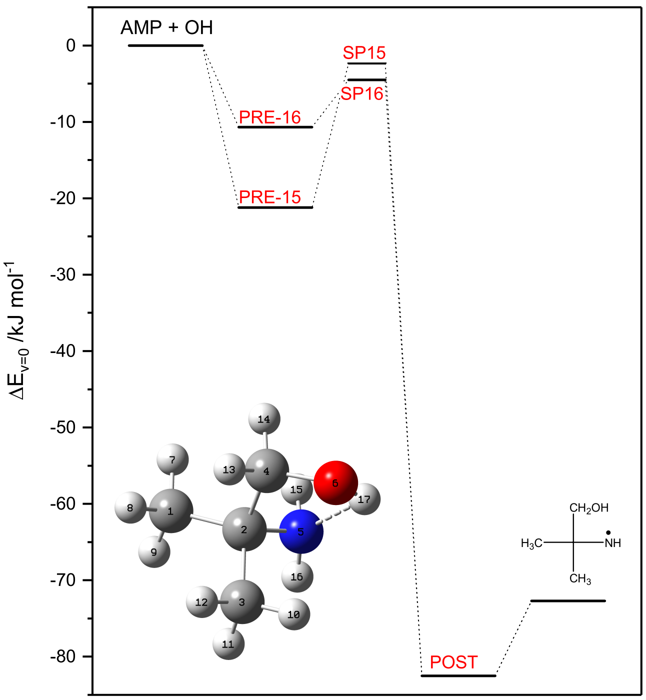

Figure S6 Relative energies of stationary points on the potential energy surface of relevance to H-abstraction by OH radicals from the NH_2-group in $(CH_3)_2(CH_2OH)CNH_2$. Results from M06-2X/aug-cc-pVTZ calculations.

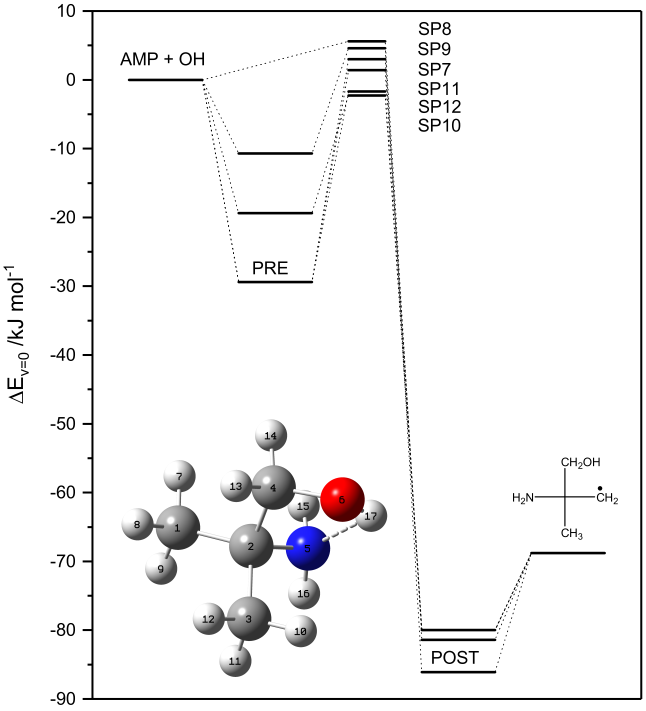

Figure S7 Relative energies of stationary points on the potential energy surface of relevance to H-abstraction by OH radicals from the CH_3-groups in $(CH_3)_2(CH_2OH)CNH_2$. Results from M06-2X/aug-cc-pVTZ calculations.

The following sections details the quantum chemistry investigation of the atmospheric chemistry of AMP. The peroxy-radical reaction routes $RO\dot{O} + RO\dot{O} \rightarrow$ ……, $RO\dot{O} + HO\dot{O} \rightarrow$ …… and $RO\dot{O} + NO \rightarrow RONO_2$ are not considered.

Atmospheric fate of the $(CH_3)_2C(NH_2)\dot{C}HOH$ radical. The hydroxyalkyl radical formed in reaction (1a) will undergo H-abstraction by O_2 leading to 2-amino-2-methylpropanal:

$\Delta H^{\ominus} = -105$ $\quad (CH_3)_2C(NH_2)CH_2OH + OH \rightarrow (CH_3)_2C(NH_2)\dot{C}HOH + H_2O$ (1a)

$\Delta H^{\ominus} = -76$ $\quad (CH_3)_2C(NH_2)\dot{C}HOH + O_2 \rightarrow (CH_3)_2C(NH_2)CHO + HO_2$ (2)

The reaction appears to proceed via an activated peroxy radical ($E^{\ddagger}$ ~150 kJ mol^{-1}) and barrier of ~70 kJ mol^{-1}. The competing internal 1,5 H-shift reactions involving the NH_2- and CH_3-groups lead to intermediate radicals, $\dot{R}CH(OOH)OH$, and have somewhat higher barriers of respectively ~100 and ~125 kJ mol^{-1}. Although all barriers are situated below the entrance energy of reactants, the two 1,5 H-shift reactions, having the higher barriers, will be of little importance under atmospheric conditions.

Dissociation of the $(CH_3)_2C(NH_2)\dot{C}HOH$ radical is also conceivable. However, the two possible dissociation routes are both endothermic, and with additional electronic barriers of 40 – 45 kJ mol^{-1} above the entrance energy of the reactants in reaction 1, they are of little importance under atmospheric conditions.

$\Delta H^{\ominus}=+110$ $\quad (CH_3)_2C(NH_2)\dot{C}HOH \xrightarrow{\Delta} (CH_3)(NH_2)C{=}CHOH + \dot{C}H_3$ (3a)

$\Delta H^{\ominus}=+115$ $\quad \xrightarrow{\Delta} (CH_3)_2C{=}CHOH + \dot{N}H_2$ (3b)

The major primary product formed following C-H abstraction from the CH_2-group in AMP will consequently be 2-amino-2-methyl-1-propanal, $(CH_3)_2C(NH_2)CHO$, Scheme S1.

Scheme S1 Major routes of atmospheric reactions following H-abstraction from the CH_2-group in AMP.

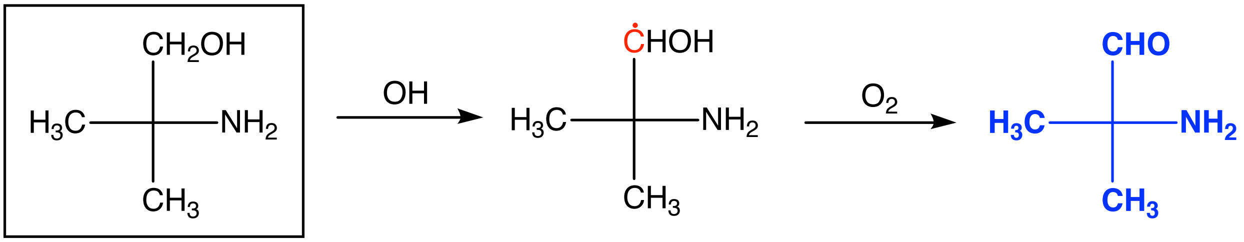

Atmospheric fate of the $(CH_3)_2C(CH_2OH)C\dot{N}H$ radical. In general, amino radicals may react with O_2, NO, NO_2 and O_3. We note that the amino radical reactions with NO and NO_2 proceed on crossing singlet/triplet surfaces, and that a theoretical calculation of the kinetics of these reactions is outside the scope of the present study. Under atmospheric conditions, the dominant reaction is normally H-abstraction from the carbon atom in α-position by O_2 leading to imines, whereas reactions with NO, NO_2 and O_3 constitute minor loss routes. Lazarou et al.[19] reported rate coefficients for the $(CH_3)_2\dot{N}$ radical reactions with NO and NO_2 at low pressure to be respectively $(8.5 \pm 1.4) \times 10^{-14}$ and $(9.1 \pm 1.4) \times 10^{-13}$ cm^3 molecule^{-1}

s^{-1} at 298 K. Lindley et al.[20] reported relative rates of the $(CH_3)_2\dot{N}$ radical reaction with NO, NO_2 and O_2 – the latter derived to be 3.9×10^{-7} times slower than the corresponding NO_2 reaction. The available kinetic data thus suggest a $(CH_3)_2\dot{N}$ + O_2 rate coefficient in the order of 10^{-19} cm^3 $molecule^{-1}$ s^{-1}. There is no information available on aminyl radical reactions with O_3, but the $\dot{N}H_2$ radical is reported to react relatively fast with O_3 ($k = 1.7\times10^{-13}$ cm^3 $molecule^{-1}s^{-1}$, extensive review [21]). Peiró–García et al.[9] characterized the $\dot{N}H_2$ + O_3 reaction in MP2, QCISD, QCISD(T), CCSD(T), CASSCF, and CASPT2 calculations with various basis sets corroborating that the reaction is an oxygen atom transfer from O_3: $\dot{N}H_2 + O_3 \rightarrow H_2N\dot{O} + O_2(^1\Delta_g)$.

Assuming that the $(CH_3)_2(CH_2OH)C\dot{N}H$ radical has rate coefficients for reactions with O_2, NO, NO_2 and O_3 alike those mentioned above, the $(CH_3)_2(CH_2OH)C\dot{N}H$ loss rates for reaction with these species will be around 5.0×10^{-1}, 1.1×10^{-2}, 2.3×10^{-1} and 1.7×10^{-1} s^{-1} under atmospheric conditions with 5 ppb NO, 10 ppb NO_2 and 40 ppb O_3. The $(CH_3)_2(CH_2OH)C\dot{N}H$ radical lifetime with respect to bimolecular reactions is therefore expected to be ~1 s, and dissociation of the radical is therefore of potential importance:

$\Delta H^{\Theta} = -73$	$(CH_3)_2C(NH_2)CH_2OH + OH$	$\rightarrow$	$(CH_3)_2(CH_2OH)C\dot{N}H + H_2O$	(1b)
$\Delta H^{\Theta} = +68$	$(CH_3)_2(CH_2OH)C\dot{N}H$	$\xrightarrow{\Delta}$	$HN{=}C(CH_3)CH_2OH + \dot{C}H_3$	(4a)
$\Delta H^{\Theta} = +65$		$\xrightarrow{\Delta}$	$HN{=}C(CH_3)_2 + \dot{C}H_2OH$	(4b)

M06-2X/aug-cc-pVTZ calculations place the two dissociation routes with barriers of respectively 41.6 and 8.8 kJ mol^{-1} above the entrance energy of the initial reactants, and RRKM calculations predict reactions (4a) and (4b) with rate coefficients of ~2×10^{-7} and 2.3×10^{-2} s^{-1}, respectively, at 298 K. The underlying quantum chemistry data are collected in Table S3. Reaction (4a) is clearly of little relevance, but the rate of reaction (4b) is comparable to those of the bimolecular reactions with NO, NO_2 and O_3 under atmospheric conditions (the $\dot{C}H_2OH$ radical will react with O_2 resulting in formaldehyde, CH_2O).

We first consider the apparently barrierless reaction between the $(CH_3)_2(CH_2OH)C\dot{N}H$ radical and O_2:

$\Delta H^{\Theta} = -36$	$(CH_3)_2(CH_2OH)C\dot{N}H + O_2$	$\rightarrow$	$(CH_3)_2(CH_2OH)CNHO\dot{O}$	(5)

The lifetime of $(CH_3)_2(CH_2OH)CNHO\dot{O}$ with respect to dissociation is calculated to be less than 10^{-7} s, and only unimolecular reactions – such as internal H-transfer reactions – may compete with back-dissociation:

$\Delta H^{\Theta} = +29$	$(CH_3)_2(CH_2OH)CNHO\dot{O}$	$\rightarrow$	$(CH_3)_2(CH_2OH)C\dot{N}OOH$	(6a)
$\Delta H^{\Theta} = +73$		$\rightarrow$	$(CH_3)(\dot{C}H_2)(CH_2OH)CNHOOH$	(6b)
$\Delta H^{\Theta} = +86$		$\rightarrow$	$(CH_3)_2(CH_2\dot{O})CNHOOH$	(6c)
$\Delta H^{\Theta} = +45$		$\rightarrow$	$(CH_3)_2(\dot{C}HOH)CNHOOH$	(6d)

The barriers to the internal H-transfer reactions are all more than 70 kJ mol above the entrance energy reactants, and they will consequently be blocked under atmospheric conditions. It can therefore be concluded unambiguously that reaction with O_2 will not represent a sink for the $(CH_3)_2(CH_2OH)C\dot{N}H$ radical.

We next consider the $(CH_3)_2(CH_2OH)C\dot{N}H$ radical reaction with NO resulting in a vibrationally excited primary nitrosamine (AMPNO):

$\Delta H^{\Theta} = -182$ $(CH_3)_2(CH_2OH)C\dot{N}H + NO \rightarrow (CH_3)_2(CH_2OH)CNHNO^{\ddagger}$ (7)

AMPNO exists in two conformations, in which the –HNNO moiety is either *syn(clinal)* or *anti(clinal)*. The energy difference between the two conformers is only a few kJ mol^{-1}, and rotational inter-conversion between the conformers is via a barrier of around 84 kJ mol^{-1} – well below the entrance energy of the reactants. Tang et al. mapped the energetics of the $CH_3\dot{N}H$ and $CH_3CH_2\dot{N}H$ reactions with NO,[22-23] which was later corrected by da Silva showing that the primary nitrosamine will not convert to the corresponding imine, but isomerize to the corresponding hydroxydiazene.[24] In the present case, the hydroxydiazene conformational manifold is very complex. The lowest energy conformers are characterized by intramolecular hydrogen bonding and can be labeled according to the (HO)CCNN, CNNO and NNOH dihedral angles (*s*, *syn-clinal*; *a*, *anti-clinal*). The nitrosamine-hydroxydiazene isomerization involves the *syn* conformation of AMPNO and the *sas* conformation of $(CH_3)_2(CH_2OH)CN{=}NOH$ via a barrier of around 125 kJ mol^{-1}:

$\Delta H^{\Theta} = -9$ $(CH_3)_2(CH_2OH)CNHNO^{\ddagger} \rightleftarrows (CH_3)_2(CH_2OH)CN{=}NOH^{\ddagger}$ (8)

The vibrationally excited hydroxydiazene may subsequently initiate coordinated internal H-abstraction and dissociation reactions:

$\Delta H^{\Theta} = -216$ $(CH_3)_2(CH_2OH)CN{=}NOH^{\ddagger} \rightarrow CH_2{=}C(CH_3)CH_2OH + N_2 + H_2O$ (9a)

$\Delta H^{\Theta} = -234$ $\rightarrow (CH_3)_2C{=}CHOH + N_2 + H_2O$ (9b)

$\Delta H^{\Theta} = +52$ $\rightarrow (CH_3)_2C{=}N{=}N + CH_2O + H_2O$ (9c)

The barrier to route (9c) is calculated to be around 70 kJ mol^{-1}. Unfortunately, the barriers to routes (9a) and (9b) could not be located exactly in M062X-calculations due to multiconfiguration issues. However, inspections of the potential energy surfaces (PES) locate the barriers to be above 100 kJ mol^{-1}. For comparison, the barriers to the related reactions $CH_3NNOH \rightarrow CH_2NN + H_2O$ and $(CH_3)_3CNNOH \rightarrow (CH_3)_2C{=}CH_2 + N_2 + H_2O$ were reported to be around 105 [24] and 100 kJ mol^{-1},[25] respectively. In any case, reaction (9c) proceeds via the *ssa* conformation of $(CH_3)_2(CH_2OH)CN{=}NOH$, and the connection between the initially formed *sas* conformer of $(CH_3)_2(CH_2OH)CN{=}NOH$ and the *ssa* conformer involves a relatively low barrier of 30-40 kJ mol^{-1} to rotation of the OH group (i.e. *ssa* ⇄ *sss* or *saa* ⇄ *sas*), and a high barrier of ~120 kJ mol^{-1} to rotation around the N=N bond (i.e. *sss* ⇄ *sas* or *ssa* ⇄ *saa*). Obviously, other routes may be envisaged, but they all involve at least one relatively high barrier.

The relative energies of stationary points on the PES relevant to the $(CH_3)_2(CH_2OH)C\dot{N}H + NO$ reaction are illustrated in Figure S8; the underlying quantum chemistry data are collected in Table S4 (the complex linking between the multitude of hydroxydiazene conformers is not included in Figure 8 and Table S4). The barriers to rotation around the C–N bond are generally below 10 kJ mol^{-1} (i.e. *ssa* ⇄ *asa*; *sss* ⇄ *ass*; *saa* ⇄ *aaa*; *sas* ⇄ *aas*) and many conformations will be involved in the isomerization, but even a simplified master equation calculation based on the PES indicated in Figure S8 suggests that very little 2-diazopropane will result in the AMPNO isomerization under atmospheric conditions.

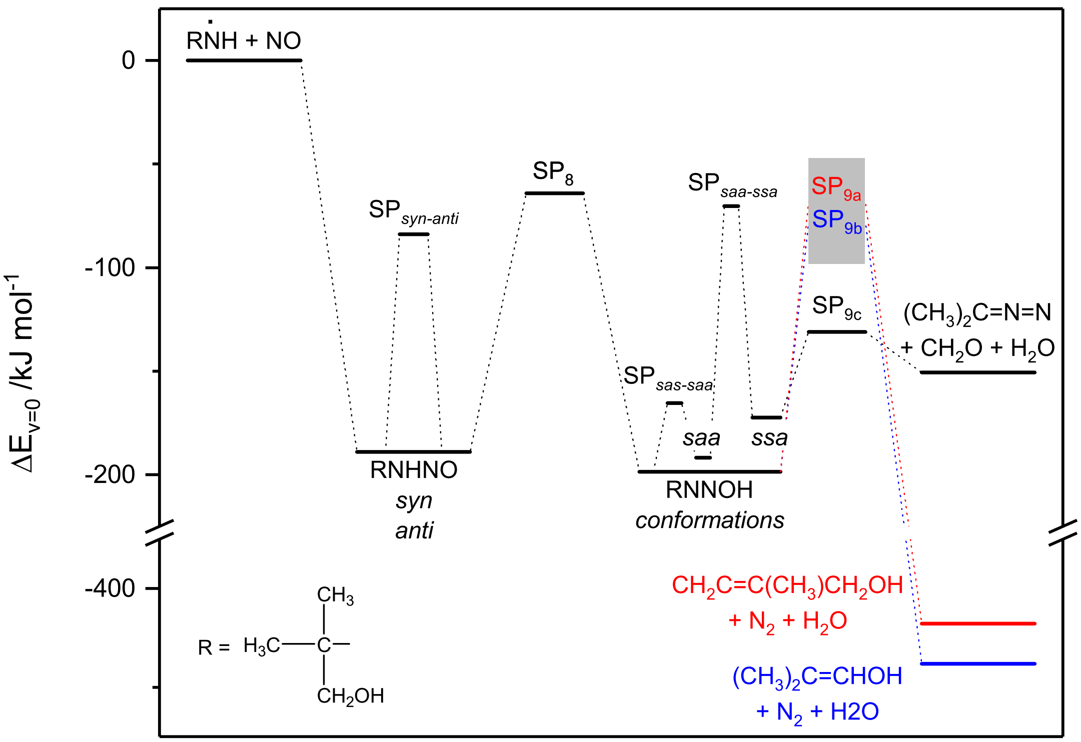

Figure S8 Relative energies of stationary points on the potential energy surface of relevance to the $(CH_3)_2(CH_2OH)C\dot{N}H$ + NO reaction, results from M06-2X/aug-cc-pVTZ calculations. The grey-shaded box covers the expected range of the saddlepoint energies of reactions (9a) and (9b), see text.

There are no experimental data on the possible atmospheric reactions of hydroxydiazenes. In contrast to the nitrosamines, hydroxydiazenes will not undergo photolysis under tropospheric conditions. B3LYP/aug-cc-pVTZ TD-DFT calculations places the first vertical singlet excitations in $(CH_3)_2(CH_2OH)CN{=}NOH$ at 280, 225 and 198 nm, which compares to 400, 204 and 185 nm in $(CH_3)_2(CH_2OH)CNHNO$).

An inspection of the bonding in $(CH_3)_2(CH_2OH)CN{=}NOH$ reveals that the (N)O-H bond enthalpy is only 348 kJ mol^{-1}, which compares to $–CH_2–$, $–NH_2$ and $–CH_3$ and –OH bond enthalpies of respectively 389, 416, 424 and 438 kJ mol^{-1} in AMP (values from G4 calculations). Similarly, the N-H bond enthalpy in $(CH_3)_2(CH_2OH)CNHNO$ is only 338 kJ mol^{-1}. Both the primary nitrosamine and the hydroxydiazenes are consequently expected to undergo very fast reactions with the OH radical:

$\Delta H^{\Theta} = -156$ $\quad (CH_3)_2(CH_2OH)CNHNO + OH \rightarrow (CH_3)_2(CH_2OH)C\dot{N}NO + H_2O$ (10)

$\Delta H^{\Theta} = -147$ $\quad (CH_3)_2(CH_2OH)CN{=}NOH + OH \rightarrow (CH_3)_2(CH_2OH)C\dot{N}NO + H_2O$ (11)

Reaction (10) proceeds via a pre-reaction complex and a submerged barrier for the *syn* conformer of $(CH_3)_3CNHNO$, whereas there is a barrier of around 14 kJ mol^{-1} above the entrance energy of reactants for the *anti* conformer. Reaction (11) also proceeds via a pre-reaction complex and a submerged barrier for the *sas* and *aas* conformers, whereas the other conformers have barriers of around 15 kJ mol^{-1} above the entrance energy of reactants.

The $(CH_3)_2(CH_2OH)C\dot{N}NO$ radical is formed with considerable internal energy and may possibly dissociate fast resulting in the formation nitrous oxide and a tertiary alkyl radical.

$\Delta H^{\ominus} = +39$ $\quad (CH_3)_2(CH_2OH)C\dot{N}NO \rightarrow (CH_3)_2(CH_2OH)\dot{C} + NNO$ (12)

The dissociation occurs via a barrier of around 80 kJ mol^{-1}, which is well below the entrance energy of the reactants in reactions (10) and (11). The PES´es of reaction sequences (10)/(12) and (11)/(12) are illustrated in Figures S9 and S10, respectively (the underlying quantum chemistry data are collected in Tables S5 and S6).

The tertiary alkyl radical formed in (12) will add O_2, react with NO and dissociate:

$\Delta H^{\ominus} = -163$ $\quad (CH_3)_2(CH_2OH)\dot{C} + O_2 \rightarrow (CH_3)_2(CH_2OH)CO\dot{O}$ (13)

$\Delta H^{\ominus} = -45$ $\quad (CH_3)_2(CH_2OH)CO\dot{O} + NO \rightarrow (CH_3)_2(CH_2OH)C\dot{O} + NO_2$ (14)

$\Delta H^{\ominus} = +13$ $\quad (CH_3)_2(CH_2OH)C\dot{O} \rightarrow (CH_3)_2CO + \dot{C}H_2OH$ (15a)

$\Delta H^{\ominus} = +20$ $\quad \rightarrow CH_3C(O)CH_2OH + \dot{C}H_3$ (15b)

The barriers to reaction (15a) and (15b) are calculated to be around 30 and 70 kJ mol^{-1}, respectively, making the latter route of little importance under atmospheric conditions (the $\dot{C}H_2OH$ radical will react with O_2 resulting in formaldehyde).

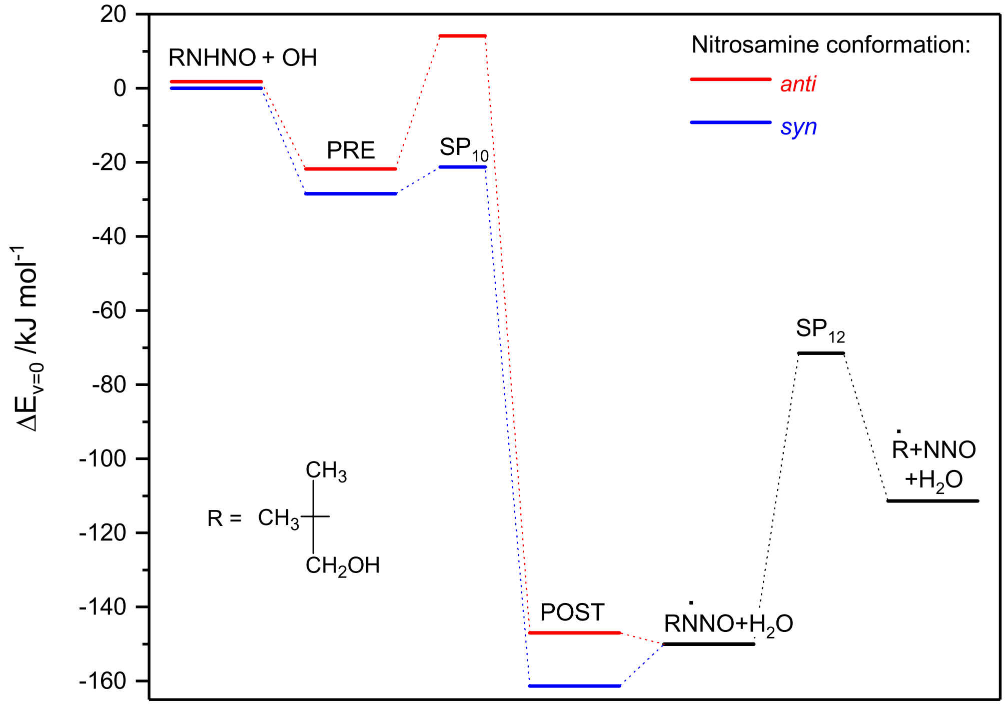

Figure S9 Relative energies of stationary points on the potential energy surface of the $(CH_3)_2(CH_2OH)CNHNO + OH$ reaction. Results from M06-2X/aug-cc-pVTZ calculations.

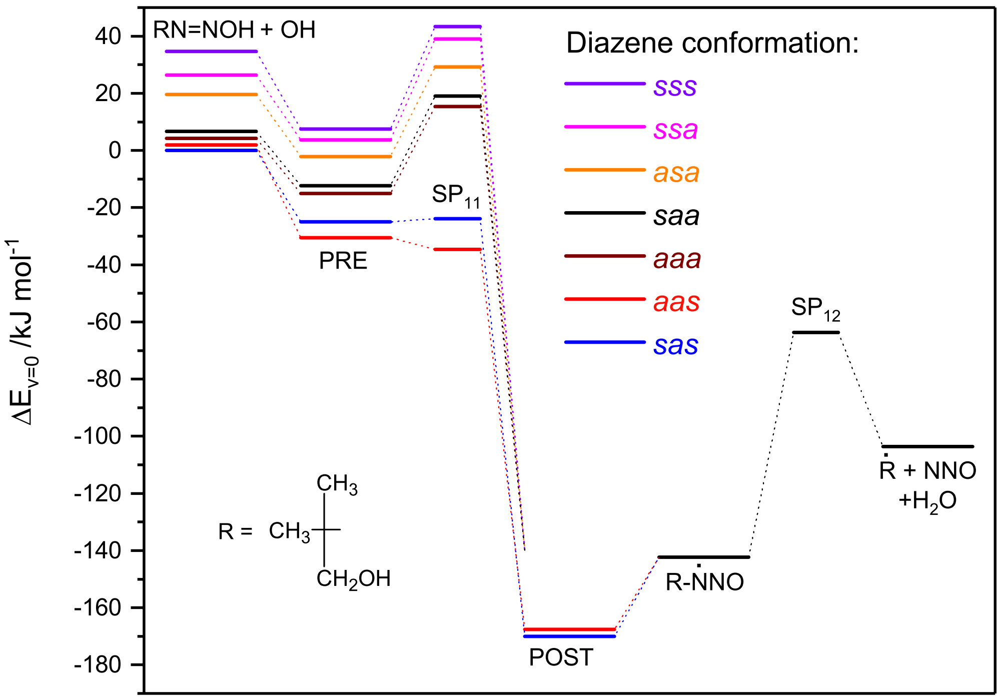

Figure S10 Relative energies of stationary points on the potential energy surface of the $(CH_3)_2(CH_2OH)CN{=}NOH$ + OH reaction. Results from M06-2X/aug-cc-pVTZ calculations.

The reaction sequences (10)/(12) and (11)/(12) were modeled in Master equation calculations in which the adduct association reaction was treated as a reversible reaction with rate coefficients approximated by typical values of $k_{association} = 4 \times 10^{-10} \times (T/298K)^{-1/6}$ cm^3 molecule^{-1} s^{-1} from Long-range transition state theory;[26] the post-reaction adduct dissociations were also treated as reversible and described similarly to the association. The calculations predict rate coefficients >1 × 10^{-10} cm^3 molecule^{-1} s^{-1} for reactions (10) and (11) and $k_{12} \approx 0.5$ s^{-1}, corresponding to an atmospheric lifetime of the $(CH_3)_2(CH_2OH)C\dot{N}NO$ radical of ~2 s. The rate coefficients for the OH radical reactions with the other conformations of the nitrosamine and hydroxyldiazene are orders of magnitude smaller and of little importance under atmospheric conditions.

The atmospheric fate of AMPNO was investigated in master equation calculations based on the PES illustrated in Figure S8 and including $k_{OH+nitrosamine} = k_{OH+hydroxydiazene} = 1 \times 10^{-10}$ cm^3 molecule^{-1} s^{-1}, and <OH> = 10^7 cm^{-1} and $\langle j_{Nitrosamine} \rangle = 2 \times 10^{-3}$ s^{-1} corresponding to the average conditions in the EUPHORE chamber experiments. The calculations show that the fate of AMPNO is very sensitive to pressure. At 1 mbar the lifetime of AMPNO is ~100 s and as much as 40% of AMPNO will isomerize and dissociate to 2-diazopropane. At 1000 mbar and $\langle \Delta E_{down} \rangle = 200$ cm^{-1} the lifetime is around 500 s, the 2-diazopropane yield is less then 0.2%, and ~50% of AMPNO will undergo photolysis and ~50% react with OH. At atmospheric conditions with <OH> = 10^6 cm^{-1} and $\langle j_{Nitrosamine} \rangle = 1 \times 10^{-3}$ s^{-1} the gas phase lifetime of AMPNO is calculated to be ~1000 s, ~90% of AMPNO will undergo photolysis and ~10% will react with OH. We

finally note that the numbers presented above will not change significantly should the all conformational issues be omitted in the model.

The $(CH_3)_2(CH_2OH)C\dot{N}H$ radical reaction with NO_2 can proceed via 2 routes leading to either a highly vibrationally excited nitramine ($AMPNO_2$) or a highly vibrationally excited *N*-nitrosooxy compound:

$\Delta H^{\Theta} = -195$ $(CH_3)_2(CH_2OH)C\dot{N}H + NO_2 \rightarrow (CH_3)_2(CH_2OH)CNHNO_2^{\ddagger}$ (16a)

$\Delta H^{\Theta} = -116$ $\rightarrow (CH_3)_2(CH_2OH)CNHONO^{\ddagger}$ (16b)

The quantum chemistry calculations show that the *N*-nitrosooxy compound will dissociate without any additional electronic barrier:

$\Delta H^{\Theta} = 35$ $(CH_3)_2(CH_2OH)CNHONO^{\ddagger} \rightarrow (CH_3)_2(CH_2OH)CNH\dot{O} + NO$ (17)

The atmospheric fate of the $(CH_3)_2(CH_2OH)CNH\dot{O}$ radical will be addressed later.

The vibrationally excited $AMPNO_2$ may initiate internal H-transfer reactions. The quantum chemistry calculations show that H-transfer may take place from one of the CH_3-groups via a barrier ca. 18 kJ mol^{-1} below the entrance energy of the initial reactants, or from the CH_2-group via a barrier ca. 3 kJ mol^{-1} below the entrance energy of the reactants. The H-transfer is in both cases orchestrating dissociation leading to an alkene/enol and HNN(O)OH, which is an isomer of nitroamide:

$\Delta H^{\Theta} = +127$ $(CH_3)_2(CH_2OH)CNHNO_2^{\ddagger} \rightarrow (CH_3)(CH_2OH)C{=}CH_2 + HNN(O)OH$ (18a)

$\Delta H^{\Theta} = +109$ $\rightarrow (CH_3)_2C{=}CHOH + HNN(O)OH$ (18b)

$\Delta H^{\Theta} = -39$ $HNN(O)OH \rightarrow H_2N\text{-}NO_2$ (19)

The energetics of the $(CH_3)_2(CH_2OH)C\dot{N}H+NO_2$ reactions is illustrated in Figure S11; the underlying quantum chemistry data are collected in Table S7. Master equation calculations based on the PES shown in Figure S11 predict $k_{15} \sim 5 \times 10^{-22}$ s^{-1}, which implies that routes (18a) and (18b) are of little importance under atmospheric conditions.

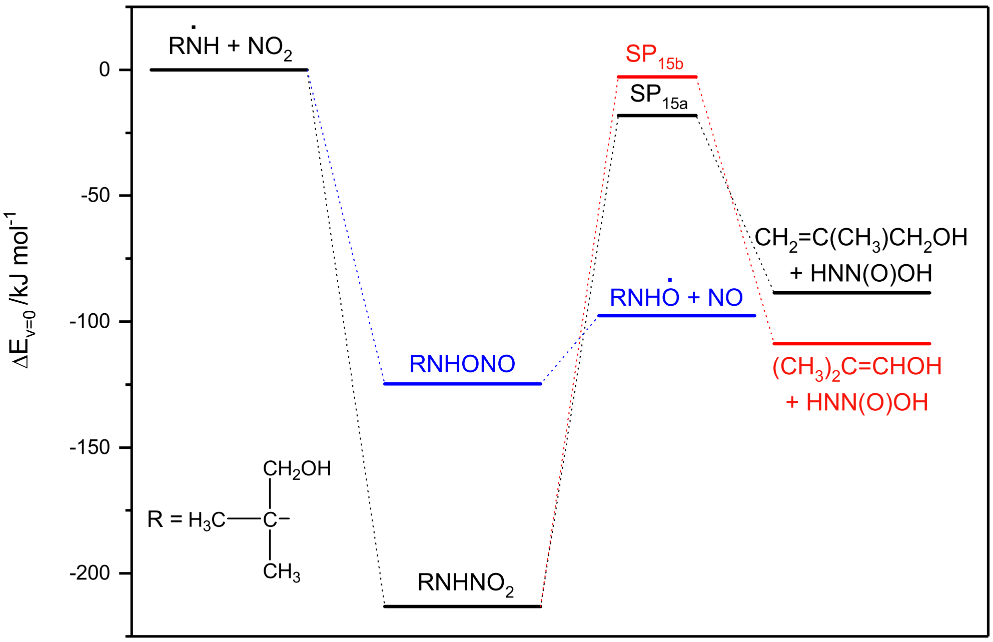

Figure S11 Relative energies of stationary points on the potential energy surface of relevance to $(CH_3)_2(CH_2OH)C\dot{N}H + NO_2$ reaction. Results from M06-2X/aug-cc-pVTZ calculations.

We next consider the $(CH_3)_2(CH_2OH)C\dot{N}H$ + O_3 reaction. All attempts to carry out single-reference calculations similar to those presented for $\dot{N}H_2 + O_3$ [9] were unsuccessful. The simpler $CH_3\dot{N}H + O_3$ system can, however, be described reasonably well at the MP2 level. The calculations indicate ~7 kJ mol^{-1} lower barrier than that calculated for the $\dot{N}H_2 + O_3$ reaction. The $CH_3\dot{N}H + O_3$ reaction is therefore expected to be considerable faster, and we suggest that this will also apply to the $(CH_3)_2(CH_2OH)C\dot{N}H + O_3$ reaction:

$\Delta H^{\Theta} = -279$ $(CH_3)_2(CH_2OH)C\dot{N}H + O_3 \rightarrow (CH_3)_2(CH_2OH)CNH\dot{O}^{\ddagger} + O_2$ (20)

The activated $(CH_3)_2(CH_2OH)CNH\dot{O}^{\ddagger}$ radical may dissociate before possible bimolecular reactions such as H-abstraction by O_2:

$\Delta H^{\Theta} = +258$ $(CH_3)_2(CH_2OH)CNH\dot{O}^{\ddagger} \rightarrow (CH_3)_2(CH_2OH)CNO + H$ (21a)

$\Delta H^{\Theta} = +225$ $\rightarrow (CH_3)_2(CH_2OH)\dot{C} + HNO$ (21b)

$\Delta H^{\Theta} = +26$ $\rightarrow (CH_3)_2(CH_2OH)CH + NO$ (21c)

$\Delta H^{\Theta} = +51$ $(CH_3)_2(CH_2OH)CNH\dot{O} + O_2 \rightarrow (CH_3)_2(CH_2OH)CNO + HO_2$ (22)

There are apparently no additional electronic barriers to reactions (21a) and (21b) and only the latter route may therefore be relevant under atmospheric conditions. A master equation calculation indicates <1% dissociation yield assuming equipartitioning of the enthalpy of reaction (20). Reaction (21c) is apparently a branch of reaction (21b) and will therefore also be blocked under atmospheric conditions.

Reaction (9) proceeds via a weakly bound pre-reaction complex, $(CH_3)_2(CH_2OH)CNH\dot{O}{\bullet}OO$, a barrier of around 80 kJ mol^{-1} and a post-reaction complex, $(CH_3)_2(CH_2OH)CNO{\bullet}HO\dot{O}$; the rate coefficient is estimated to be < 10^{-26} cm^3 molecule^{-1} s^{-1} from conventional transition state theory including tunneling

corrections. Reaction (22) will therefore not represent an important atmospheric sink for the $(CH_3)_3CNH\dot{O}$ radical.

Other relevant loss processes for the $(CH_3)_2(CH_2OH)CNH\dot{O}$ radical includes reaction with O_3. The experimental study on the $\dot{N}H_2 + O_3$ reaction by Bulatov et al.[4] also included a best estimate for the $H_2N\dot{O}$ + O_3 reaction: $k_{H_2N\dot{O}+O_3 \rightarrow \dot{N}H_2+2O_2} = (2.0 \pm 1.5) \times 10^{-14}$ cm^3 molecule^{-1} s^{-1} at room temperature in agreement with the upper limit of 5×10^{-14} cm^3 molecule^{-1} s^{-1} deducted in experiments by Patrick and Golden.[7] The computational problems related to the $(CH_3)_2(CH_2OH)C\dot{N}H + O_3$ reaction also applies to the $(CH_3)_2(CH_2OH)CNH\dot{O} + O_3$ reaction. Again, results from MP2 calculations on the $CH_3NH\dot{O}$ and $H_2N\dot{O}$ reactions with O_3 show the same lowering of the barrier as found for the corresponding $CH_3\dot{N}H$ and $\dot{N}H_2$ reactions. We confidently suggest that the $(CH_3)_2(CH_2OH)CNH\dot{O}$ reaction with O_3 will be considerably faster than that of $H_2N\dot{O}$.

$\Delta H^{\Theta} = -41$ $\quad (CH_3)_2(CH_2OH)CNH\dot{O} + O_3 \rightarrow (CH_3)_2(CH_2OH)CNHO\dot{O} + O_2$ (23)

$\Delta H^{\Theta} = +36$ $\quad (CH_3)_2(CH_2OH)CNHO\dot{O} \rightarrow (CH_3)_2(CH_2OH)C\dot{N}H + O_2$ (24)

We conclude from the above that the primary aminyl radical and the corresponding nitroxide will simply react with O_3 in a cyclic manner ($R\dot{N}H + O_3 \rightarrow RNH\dot{O} + O_2$; $RNH\dot{O} + O_3 \rightarrow R\dot{N}H + 2\ O_2$). Consequently, we estimate that under atmospheric conditions with 5 ppb NO and 10 ppb NO_2, 10-15% of the $(CH_3)_2(CH_2OH)C\dot{N}H$ radical will dissociate forming the imine, $HN{=}C(CH_3)_2$.

An initial hydrogen abstraction from the amino group in AMP will lead to imine and nitramine formation. In addition, a nitrosamine in equilibrium with hydroxydiazene will also be formed. The main reaction routes are shown in Scheme S2.

Scheme S2 Major routes of atmospheric reactions following H-abstraction from the NH_2-group in AMP.

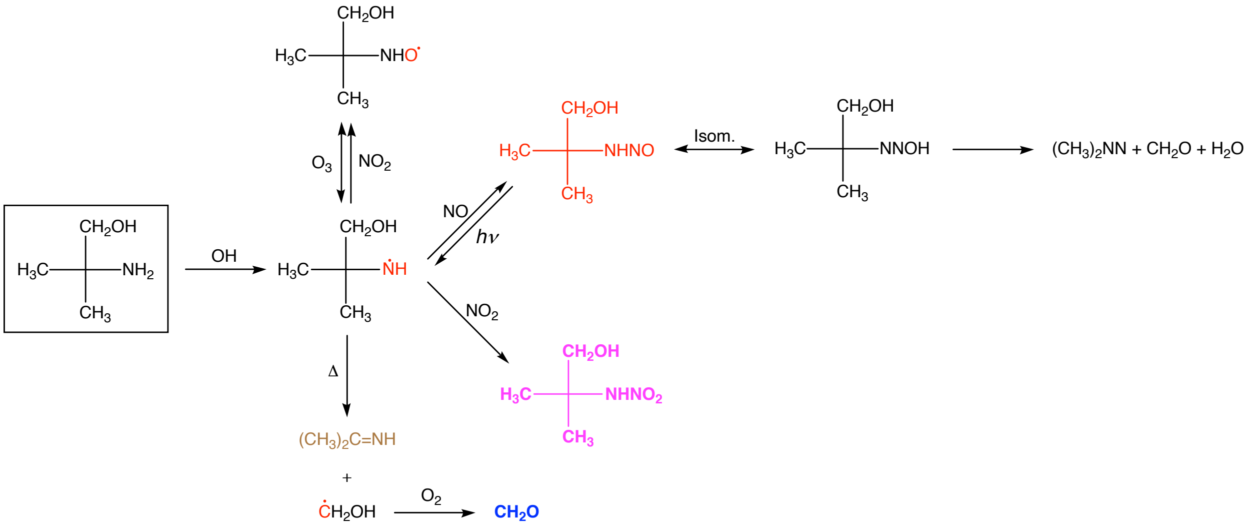

Atmospheric fate of the $\boldsymbol{(CH_3)(CH_2OH)C(NH_2)\dot{C}H_2}$ ***radical.*** The alkyl radical formed following the initial hydrogen abstraction from one of the CH_3-groups will add O_2:

$\Delta H^{\Theta} = -71$ $(CH_3)_2C(NH_2)CH_2OH + OH \rightarrow \dot{C}H_2C(CH_3)(NH_2)CH_2OH + H_2O$ (1c)

$\Delta H^{\Theta} = -143$ $\dot{C}H_2C(CH_3)(NH_2)CH_2OH + O_2 \rightarrow \dot{O}OCH_2C(CH_3)(NH_2)CH_2OH^{\ddagger}$ (25)

The vibrationally excited peroxy radical may initiate consecutive internal H-transfer reactions, possibly intervened by bimolecular reactions or dissociations, before reacting with NO to give the corresponding oxy radical:

$\Delta H^{\Theta} = +78$ $\dot{O}OCH_2C(CH_3)(NH_2)CH_2OH^{\ddagger} \rightleftarrows HOOCH_2C(CH_3)(NH_2)CH_2\dot{O}^{\ddagger}$ (26a)

$\Delta H^{\Theta} = +24$ $\rightleftarrows HOOCH_2C(CH_3)(NH_2)\dot{C}HOH^{\ddagger}$ (26b)

$\Delta H^{\Theta} = +56$ $\rightleftarrows HOOCH_2C(CH_3)(\dot{N}H)CH_2OH^{\ddagger}$ (26c)

$\Delta H^{\Theta} = +75$ $\rightleftarrows HOOCH_2C(\dot{C}H_2)(NH_2)CH_2OH^{\ddagger}$ (26d)

$\Delta H^{\Theta} = -61$ $\dot{O}OCH_2C(CH_3)(NH_2)CH_2OH + NO \rightarrow \dot{O}CH_2C(CH_3)(NH_2)CH_2OH + NO_2$ (27)

The barriers to reactions (26a)-(26d) are calculated to be 99, 101, 84 and 109 kJ mol^{-1}, respectively – all situated well below the entrance energy of the reactants forming the peroxy radical.

The vibrational excited $HOOCH_2C(CH_3)(NH_2)CH_2\dot{O}^{\ddagger}$ radical formed in (26a) may dissociate or initiate other H-transfer reactions before reacting with O_2 to give the corresponding aldehyde:

$\Delta H^{\Theta} = +22$ $HOOCH_2C(CH_3)(NH_2)CH_2\dot{O}^{\ddagger} \rightarrow HOOCH_2\dot{C}(CH_3)(NH_2) + CH_2O$ (28)

$\Delta H^{\Theta} = -22$ $HOOCH_2C(CH_3)(NH_2)CH_2\dot{O}^{\ddagger} \rightleftarrows HOOCH_2C(CH_3)(\dot{N}H)CH_2OH^{\ddagger}$ (29a)

$\Delta H^{\Theta} = -5$ $\rightleftarrows HOOCH_2C(\dot{C}H_2)(NH_2)CH_2OH^{\ddagger}$ (29b)

$\Delta H^{\Theta} = -22$ $\rightleftarrows HOO\dot{C}HC(CH_3)(NH_2)CH_2OH^{\ddagger}$ (29c)

$\Delta H^{\Theta} = -131$ $HOOCH_2C(CH_3)(NH_2)CH_2\dot{O} + O_2 \rightarrow HOOCH_2C(CH_3)(NH_2)CHO + HO_2$ (30)

The barrier to the dissociation reaction (28) and to the H-transfer reactions (29a)-(29d) are calculated to be 10, 74, 99 and 67 kJ mol^{-1}, respectively, and it can be concluded that three H-transfer reactions will be of little importance under atmospheric conditions.

The hydroxyalkyl radical, $HOOCH_2C(CH_3)(NH_2)\dot{C}HOH$, formed in (26b) will undergo H-abstraction by O_2 with a rate coefficient $k_{29} \sim 2 \times 10^{-11}$ cm^3 $mole^{-1}$ s^{-1}:[27]

$\Delta H^{\Theta} = -77$ $HOOCH_2C(CH_3)(NH_2)\dot{C}HOH + O_2 \rightarrow HOOCH_2C(CH_3)(NH_2)CHO + HO_2$ (31)

Finally, the $HOOCH_2C(CH_3)(\dot{N}H)CH_2OH$ radical formed in (26c) may react with NO, NO_2, O_2 and O_3 (slow reactions, see above), whereas the $HOOCH_2C(\dot{C}H_2)(NH_2)CH_2OH$ radical will add O_2 ($k_{25} \sim 10^{-11}$ cm^3 $molecule^{-1}$ s^{-1}):[27]

$\Delta H^{\Theta} = -153$ $HOOCH_2C(\dot{C}H_2)(NH_2)CH_2OH + O_2 \rightarrow HOOCH_2C(CH_2O\dot{O})(NH_2)CH_2OH$ (32)

The PES of the first steps in the $\dot{C}H_2C(CH_3)(NH_2)CH_2OH + O_2$ reaction sequence (25)-(32) is illustrated in Figure S12; the underlying quantum chemistry data are collected in Table S8.

The atmospheric fate of the $\dot{C}H_2C(CH_3)(NH_2)CH_2OH$ radical was investigated in master equation calculations based on the PES shown in Figure S12 assuming an RO_2 + NO rate coefficient $k_{21} \sim 9 \times 10^{-12}$ cm^3 $molecule^{-1}$ s^{-1},[27] and a fictitious aminyl radical loss rate of 10^3 s^{-1}. The calculations show that >99% of

the $\dot{O}OCH_2C(CH_3)(NH_2)CH_2OH$ radical will be converted to $\dot{O}CH_2C(CH_3)(NH_2)CH_2OH$ virtually independent on the NO mixing ratio.

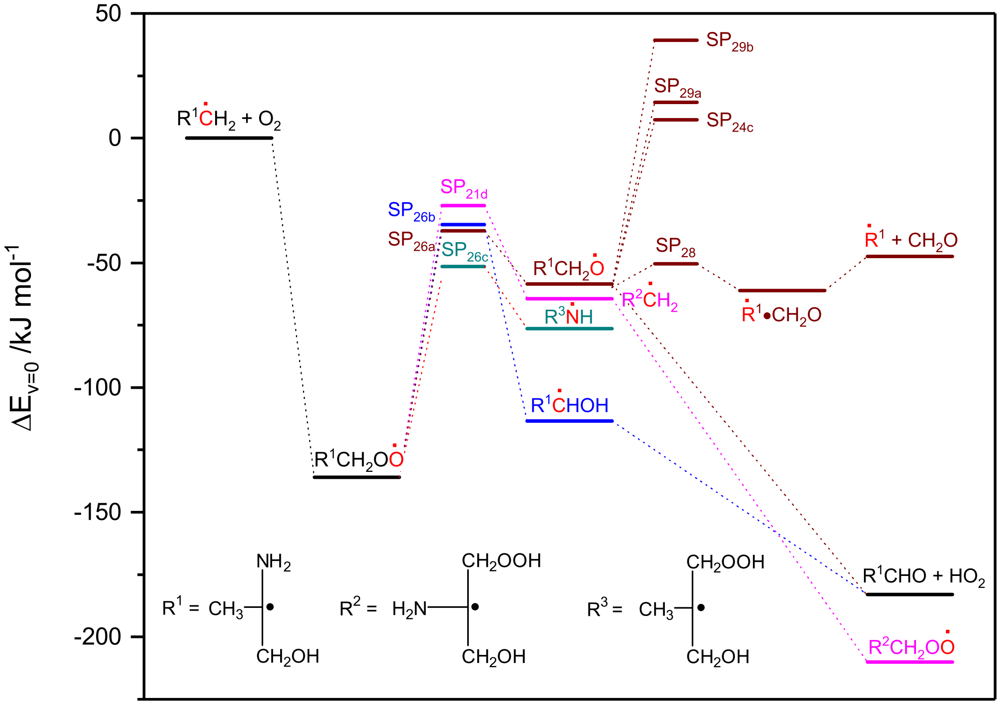

Figure S12 Relative energies of stationary points on the potential energy surface of relevance to the first steps in the $(CH_3)(CH_2OH)(NH_2)C\dot{C}H_2 + O_2$ reaction. Results from M06-2X/aug-cc-pVTZ calculations.

The oxy-radical formed in reaction (27) may either react with O_2, undergo internal H-transfer reactions, or dissociate. The reaction with O_2 ($k_{27} \sim 10^{-14}$ cm^3 molecule^{-1} s^{-1})[27] leads to the corresponding aldehyde, 2-amino-3-hydroxy-2-methylpropanal:

ΔH^{Θ} =-132 $\qquad \dot{O}CH_2C(CH_3)(NH_2)CH_2OH + O_2 \rightarrow CHOC(CH_3)(NH_2)CH_2OH + HO_2$ (33)

The internal H-transfer reactions include three different 1,4-hydrogen shift reactions ($H_2\underbrace{CH\cdots\cdots OC}_{C}H_2$, $H\underbrace{NH\cdots\cdots OC}_{C}H_2$, $HOH\underbrace{CH\cdots\cdots OC}_{C}H_2$) and one 1,5-hydrogen shift reaction ($H_2\underbrace{COH\cdots\cdots OC}_{C}H_2$) connecting two identical radicals. The barriers to the three 1,4-hydrogen shift reactions are calculated to be around 70, 80 and 85 kJ mol^{-1}, respectively – all well above the entrance energy of the reactants in reaction (27).

ΔH^{Θ} = -50 $\qquad \dot{O}CH_2C(CH_3)(NH_2)CH_2OH \rightleftarrows HOCH_2C(CH_3)(NH_2)\dot{C}HOH$ (34a)

ΔH^{Θ} = -18 $\qquad \rightleftarrows HOCH_2C(CH_3)(\dot{N}H)CH_2OH$ (34b)

ΔH^{Θ} = -13 $\qquad \rightleftarrows HOCH_2C(\dot{C}H_2)(NH_2)CH_2OH$ (34c)

Finally, dissociation of $\dot{O}CH_2C(CH_3)(NH_2)CH_2OH$ leads to a tertiary alkyl-radical and formaldehyde:

ΔH^{Θ} =+29 $\qquad \dot{O}CH_2C(CH_3)(NH_2)CH_2OH \xrightarrow{\Delta} \dot{C}(CH_3)(NH_2)CH_2OH + CH_2O$ (35)

Figure S13 illustrates the stationary points on the PES related to atmospheric fate of the $\dot{O}CH_2C(CH_3)(NH_2)CH_2OH$ radical; the underlying quantum chemistry data are collected in Table S9.

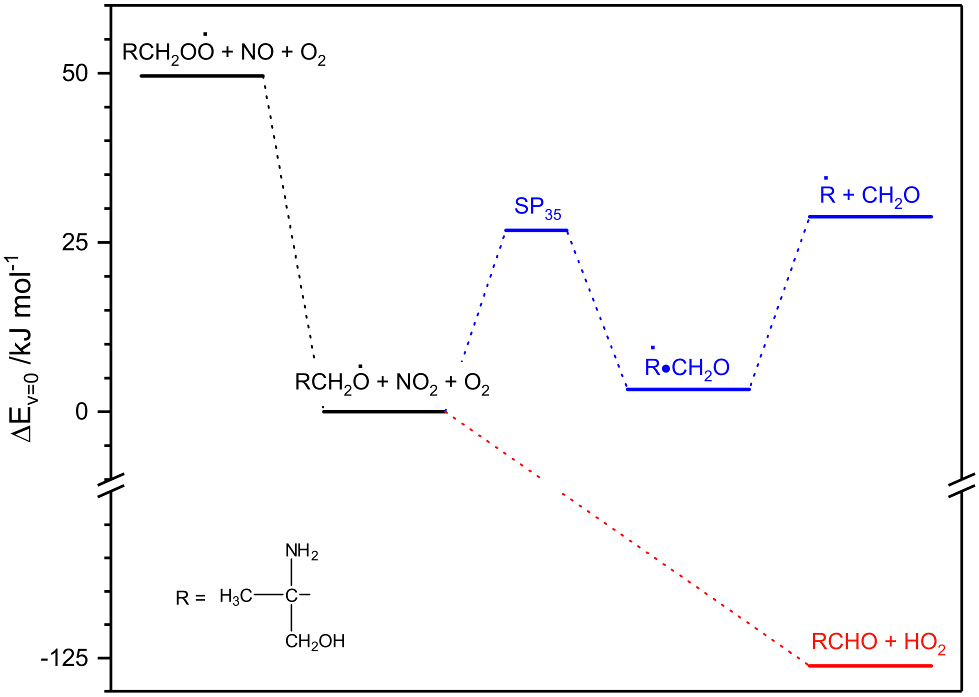

Figure S13 Relative energies of stationary points on the potential energy surface of relevance to the Ȯ$CH_2C(CH_3)(NH_2)CH_2OH$ dissociation. Energy levels in blue color include the energies of O_2 and NO_2; energy level in red color includes the energy of NO_2. Results from M06-2X/aug-cc-pVTZ calculations.

The branching between the hydrogen abstraction and the dissociation reaction was examined using a master equation model based on the potential energy surface shown in Figure S13 (the 1,4-H shift reactions can safely be neglected as their barriers are much higher than that of the dissociation). The calculations reveal that the branching between reaction (33) and (35) depends strongly on the energy portioning in reaction (27) and the rate of reaction (33). Assuming equipartitioning of the reaction enthalpy in reaction (27), and $k_{22} = 4.7 \times 10^{-12}$ cm^3 molecule^{-1} s^{-1} (which is the value reported for $(CH_3)_3CCH_2$Ȯ + NO [28]) results in <1 % yield of 2-amino-3-hydroxy-2-methylpropanal. There are no experimental data in the literature on how the energy is distributed in ROO + NO reactions. If the product NO_2 is vibrationally excited by one quantum of the stretching vibration (~19 kJ mol^{-1}) the yield of 2-amino-3-hydroxy-2-methylpropanal reaches 1.7% (intuitively, a reaction of the type AB + C→A – B ……C→A……B – C→A + BC will result in the deposit of quantum of stretching vibration in the product BC). We suggest a conservative upper limit yield of 5% 2-amino-3-hydroxy-2-methylpropanal.

The Ċ$(CH_3)(NH_2)CH_2OH$ radical will add O_2 leading to a vibrationally excited peroxy radical that may initiate internal H-transfer reactions before reacting with NO:

ΔH^{Θ}=-159 Ċ$(CH_3)(NH_2)CH_2OH + O_2 \rightarrow$ Ȯ$OC(CH_3)(NH_2)CH_2OH^{\ddagger}$ (36)

ΔH^{Θ}=+89	$\dot{O}OC(CH_3)(NH_2)CH_2OH^{\ddagger}$	$\rightarrow$	$HN{=}C(CH_3)CH_2OH + HO_2$	(37a)
ΔH^{Θ}=+119		$\rightarrow$	$CH_2{=}C(NH_2)CH_2OH + HO_2$	(37b)
ΔH^{Θ}=+104		$\rightarrow$	$CH_3(NH_2)C{=}CHOH + HO_2$	(37c)
ΔH^{Θ}=-48	$\dot{O}OC(CH_3)(NH_2)CH_2OH + NO$	$\rightarrow$	$\dot{O}C(CH_3)(NH_2)CH_2OH + NO_2$	(38)

Figure S14 illustrates the stationary points on the PES relevant to the fate of the $\dot{C}(CH_3)(NH_2)CH_2OH$ radical; the underlying quantum chemistry data are collected in Table S10. The branching between the internal H-transfer reactions and the peroxy radical reaction with NO was examined using a master equation model based on the PES shown in Figure S14. The calculation reveals that reactions (37b) and (37c) are of no importance under atmospheric conditions, and that the branching between reaction (37a) and (38) is around 60 : 40 – virtually independent on the NO mixing ratio in the range 2 – 50 ppbV.

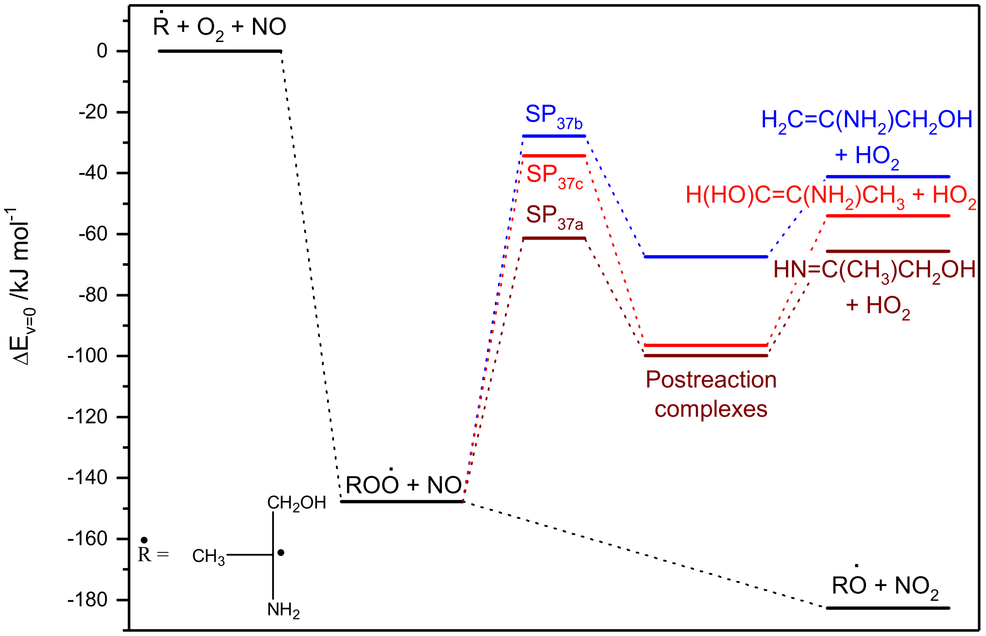

Figure S14 Relative energies of stationary points on the potential energy surface of relevance to the $\dot{C}(CH_3)(NH_2)CH_2OH + O_2$ reaction. Color coded stationary points include the energy of NO. Results from M06-2X/aug-cc-pVTZ calculations.

The tertiary oxy-radical formed in reaction (38) can only undergo dissociation reactions:

ΔH^{Θ} = -30	$\dot{O}C(CH_3)(NH_2)CH_2OH$	$\xrightarrow{\Delta}$	$CH_3C(O)NH_2 + \dot{C}H_2OH$	(39a)
ΔH^{Θ} = +39		$\xrightarrow{\Delta}$	$CH_3C(O)CH_2OH + \dot{N}H_2$	(39b)
ΔH^{Θ} = -15		$\xrightarrow{\Delta}$	$NH_2C(O)CH_2OH + \dot{C}H_3$	(39c)

Reactions (39a)-(39c) are calculated with barriers of respectively 29, 59 and 61 kJ mol^{-1}, and it can be concluded that the only product resulting from $\dot{O}C(CH_3)(NH_2)CH_2OH$ dissociation under atmospheric conditions will be acetamide, $CH_3C(O)NH_2$. The atmospheric chemistry of the $\dot{C}H_2OH$ radical is well established – it reacts with O_2 to give formaldehyde, CH_2O.

It is concluded from the theoretical study that the products formed following H-abstraction from one of the -CH_3 groups in AMP will be CH_2O (formaldehyde), $CH_3C(CH_2OH)(NH_2)CHO$ (2-amino-3-hydroxy-2-methylpropanal), $HN{=}C(CH_3)CH_2OH$ (2-iminopropan-1-ol) and $CH_3C(O)NH_2$ (acetamide), see Scheme S3.

Scheme S3 Major routes of atmospheric reactions following H-abstraction from one of the CH_3-groups in AMP.

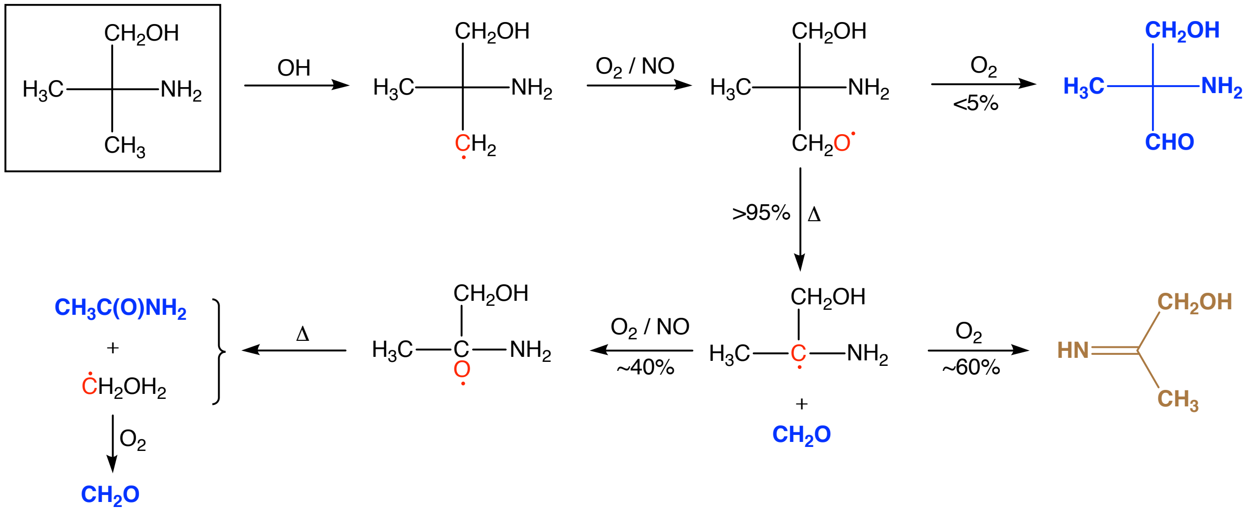

Atmospheric fate of the $(CH_3)_2C(NH_2)CH_2\dot{O}$ radical. The generic reactions of alkoxy radicals include isomerization, dissociation and H-abstraction by O_2:

$\Delta H^{\Theta} = -56$	$(CH_3)_2C(NH_2)CH_2OH + OH$	$\rightarrow$	$(CH_3)_2C(NH_2)CH_2\dot{O} + OH$	(1d)
$\Delta H^{\Theta} = +26$	$(CH_3)_2C(NH_2)CH_2\dot{O}$	$\xrightarrow{\Delta}$	$(CH_3)_2\dot{C}(NH_2) + CH_2O$	(40)
$\Delta H^{\Theta} = -130$	$(CH_3)_2C(NH_2)CH_2\dot{O} + O_2$	$\rightarrow$	$(CH_3)_2C(NH_2)CHO + HO_2$	(41)

The two possible isomerization reactions are both 1,4-hydrogen shift reactions, $H_2\underbrace{CH\cdots\cdots OC}_{C}H_2$ and $H\underbrace{NH\cdots\cdots OC}_{C}H_2$; they are calculated with barriers of around 40 kJ mol^{-1} above the entrance energy of the reaction in reaction (1d). The dissociation reaction (40) proceeds via a relatively low barrier and a post reaction adduct on the exit side, see Figure S15 (the underlying quantum chemistry data are collected in Table S11). The low barrier to dissociation makes the isomerization reactions of little importance under atmospheric conditions.

The branching between hydrogen abstraction and dissociation was examined using a master equation model based on the potential energy surface shown in Figure S15. The calculations reveal that, virtually independent on the energy partitioning in reaction (1d), dissociation will completely dominate the fate of the $(CH_3)_2C(NH_2)CH_2\dot{O}$ radical under atmospheric conditions.

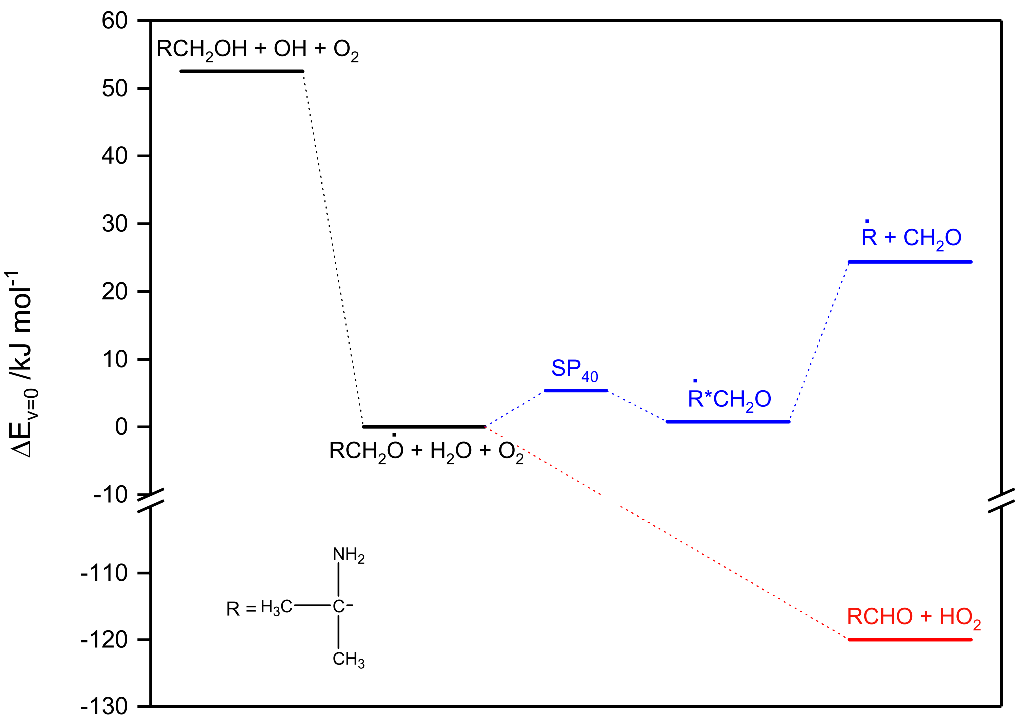

Figure S15 Relative energies of stationary points on the potential energy surface of relevance to the $(CH_3)_2(NH_2)CCH_2\dot{O}$ radical dissociation. Results from M06-2X/aug-cc-pVTZ calculations.

The $(CH_3)_2\dot{C}(NH_2)$ radical is assumed react along the generic routes established for tertiary alkyl-radicals:

$\Delta H^{\Theta} = -161$	$(CH_3)_2\dot{C}(NH_2) + O_2$	$\rightarrow$	$(CH_3)_2(NH_2)CO\dot{O}^{\ddagger}$	(42)
$\Delta H^{\Theta} = +95$	$(CH_3)_2(NH_2)CO\dot{O}^{\ddagger}$	$\rightarrow$	$(CH_3)_2C{=}NH + HO_2$	(43a)
$\Delta H^{\Theta} = +113$		$\rightarrow$	$(CH_3)(NH_2)C{=}CH_2 + HO_2$	(43b)
$\Delta H\Theta = -39$	$(CH_3)_2(NH_2)CO\dot{O} + NO$	$\rightarrow$	$(CH_3)_2(NH_2)C\dot{O} + NO_2$	(44)

Reactions (43a) and (43b) are calculated with carriers well below the entrance energy of the reactants in reaction (42) and to proceed via post reaction complexes on the exit side. Figure S16 illustrates the stationary points of the PES of $(CH_3)_2\dot{C}(NH_2) + O_2$ reaction; the underlying quantum chemistry data are collected in Table S12.

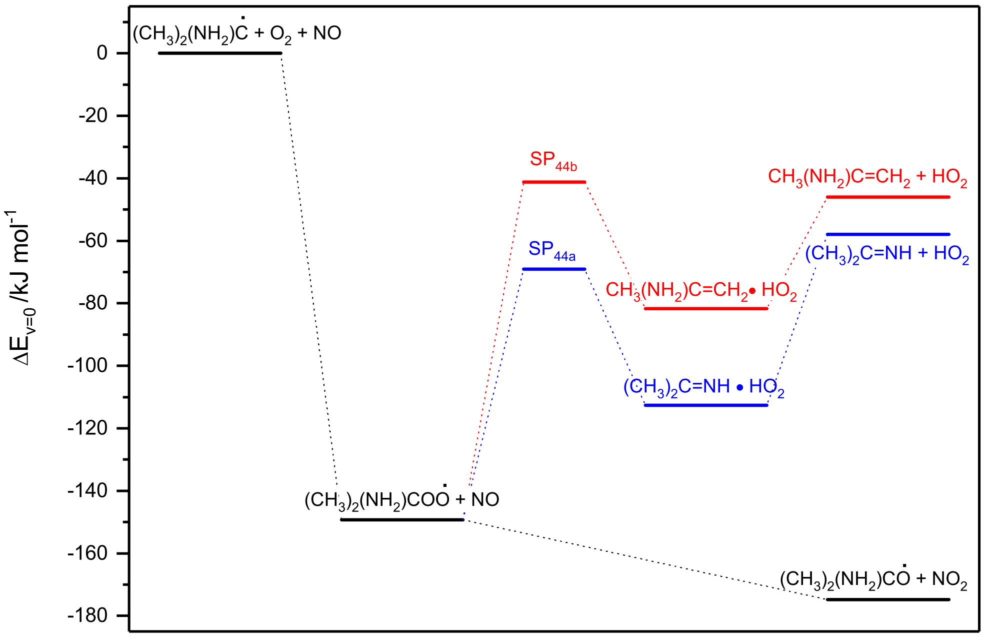

Figure S16 Relative energies of stationary points on the potential energy surface of the $(CH_3)_2(NH_2)\dot{C} + O_2$ reaction. Levels in blue color include the energy of NO. Results from M06-2X/aug-cc-pVTZ calculations.

The branching between reactions (43a), (43b) and (44) was examined using a master equation model based on the potential energy surface shown in Figure S16, and assuming $k_{34} \approx 4 \times 10^{-12}$ cm^3 molecule^{-1} s^{-1}, which is the value for the analogue $(CH_3)_3CO\dot{O}$ + NO reaction.[29-30] The results show <1 % yield of $(CH_3)(NH_2)C{=}CH_2$, 92 % yield of $(CH_3)_2C{=}NH$, and 7-8 % yield of the $(CH_3)_2(NH_2)C\dot{O}$ radical for an NO mixing ratio of 2 ppb. The calculated branching does not change significantly by increasing the NO mixing ratio to 20 ppb.

The only route available to tertiary alkoxy radicals is dissociation:

$\Delta H^{\Theta} = +33$ $(CH_3)_2(NH_2)C\dot{O} \xrightarrow{\Delta} CH_3C(O)CH_3 + \dot{N}H_2$ (45a)

$\Delta H^{\Theta} = -29$ $\xrightarrow{\Delta} CH_3C(O)NH_2 + \dot{C}H_3$ (45b)

The barriers to the dissociation reactions (45a) and (45b) are calculated to be respectively around 40 and 30 kJ mol^{-1} above the entrance energy of the reactants in reaction (44), and it can therefore be concluded that acetamide, $CH_3C(O)NH_2$, will be the only product from $(CH_3)_2(NH_2)C\dot{O}$ radical dissociation. The atmospheric chemistry of the $\dot{C}H_3$ radical is well established: formaldehyde is the dominant product.

The theoretical study indicates that the major products resulting upon H-abstraction from the OH-group in AMP will be CH_2O (formaldehyde), $(CH_3)_2C{=}NH$ (propan-2-imine) and $CH_3C(O)NH_2$ (acetamide):

Scheme S4 Major routes of atmospheric reactions following H-abstraction from the OH-group in AMP.

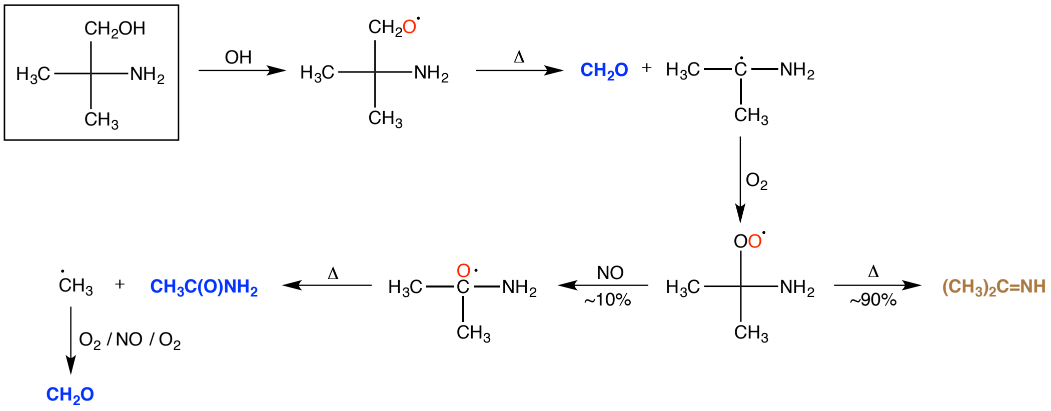

Table S2 Quantum chemistry results for the $(CH_3)_2C(NH_2)CH_2OH$ + OH reaction. Electronic energies of reactants, intermediates and products (/Hartree), and relative energies including Zero Point Energies, $\Delta E_{v=0}$ (/kJ mol^{-1}), of stationary points on the potential energy surface.

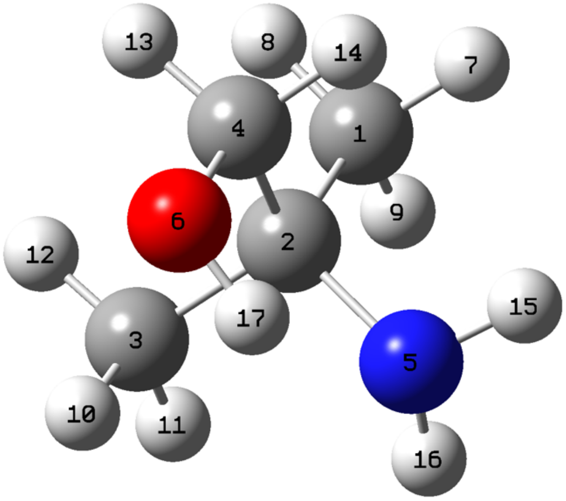

	M06-2X/aug-cc-pVTZ		
Species	E_{Elec}	E_{ZPE}	$\Delta E_{v=0}$
$(CH_3)_2C(NH_2)CH_2OH$	-289.007907	0.155148	
OH	-75.733810	0.008588	
Sum Reactants	-364.741717	0.163736	0
PRE-H7	-364.738586	0.162016	-29.4
SP-H7	-364.738736	0.161901	3.0
POST-H7	-364.774867	0.164091	-86.1
SP-H8	-364.737522	0.161665	5.6
POST-H8	-364.772996	0.164021	-81.4
PRE-H9	-364.747521	0.165477	-10.7
SP-H9	-364.738069	0.161822	4.6
POST-H9	-364.774907	0.164347	-85.5
PRE-H10	-364.752350	0.166991	-19.4
SP-H10	-364.741477	0.162634	-2.3
POST-H10	-364.777256	0.165298	-89.2
PRE-H11	-364.756102	0.166809	-29.7
SP-H11	-364.739798	0.162357	1.4
POST-H11	-364.775019	0.164796	-84.7
PRE-H12 (PRE-H13)	-364.756128	0.166967	-29.4
SP-H12	-364.740383	0.161742	-1.7
POST-H12	-364.778529	0.165168	-92.9
$(CH_3)(CH_2OH)(NH_2)C\dot{C}H_2$	-288.335946	0.140329	
H_2O	-76.430107	0.021551	
Sum Products	-364.766053	0.161880	-68.8
PRE-H13 (PRE-H12)	-364.756128	0.166967	-29.4
SP-H13	-364.741876	0.162369	-4.0

POST-H13	-364.789703	0.165911	-120.3
PRE-H14	-364.756128	0.166967	-29.4
SP-H14	-364.746983	0.163757	-13.8
POST-H14	-364.791988	0.166124	-125.7
$(CH_3)_2(NH_2)C\dot{C}HOH$	-288.350220	0.141657	
H_2O	-76.430107	0.021551	
Sum Products	-364.780327	0.163208	-102.8
PRE-H17	-364.752559	0.166519	-21.2
SP-H17	-364.733654	0.161043	14.1
POST-H17	-364.769725	0.164919	-70.4
$(CH_3)_2(NH_2)CCH_2\dot{O}$	-288.329715	0.140288	
H_2O	-76.430107	0.021551	
Sum Products	-364.759822	0.161839	-52.5
PRE-H15	-364.752559	0.166521	-21.2
SP-H15	-364.741883	0.163014	-2.3
POST-H15	-364.783081	0.165837	-103.1
PRE-H16	-364.747521	0.165473	-10.7
SP-H16	-364.742319	0.162632	-4.5
POST-H16	-364.774023	0.164621	-82.5
$(CH_3)_2(CH_2OH)C\dot{N}H$	-288.337782	0.140666	
H_2O	-76.430107	0.021551	
Sum Products	-364.767889	0.162217	-72.7

Table S2, continued: Vibrational frequencies (/cm^{-1}), Rotational constants (B /GHZ) and Cartesian Coordinates (/Å) of the species listed above. Results from M06-2X/aug-cc-pVTZ calculations.

Species	Coordinates
$(CH_3)_2C(NH_2)CH_2OH$<br><br>152.3, 223.0, 250.0, 260.7, 285.3, 348.6, 369.4, 415.9, 444.4, 524.9, 571.3, 779.0, 872.0, 916.4, 950.0, 958.0, 995.5, 1048.4, 1097.6, 1128.4, 1204.4, 1240.0, 1295.1, 1351.3, 1376.2, 1404.4, 1417.9, 1443.9, 1484.9, 1486.7, 1502.9, 1511.5, 1516.9, 1645.1, 2997.1, 3048.0, 3059.1, 3099.4, 3119.6, 3122.9, 3128.7, 3153.7, 3513.2, 3597.8, 3790.1<br><br>B: 4.5514025 2.7033291 2.6683275	C -1.618997 -0.812180 -0.391283<br>C -0.392830 0.004617 0.007282<br>C -0.477049 1.410149 -0.573757<br>C 0.870655 -0.703054 -0.492733<br>N -0.235875 0.124321 1.465523<br>O 2.042510 -0.032764 -0.100073<br>H -1.575536 -1.812755 0.043301<br>H -1.685999 -0.916200 -1.475631<br>H -2.530242 -0.326384 -0.039464<br>H 0.412768 1.983011 -0.319887<br>H -1.354675 1.930794 -0.185306<br>H -0.563283 1.366947 -1.659331<br>H 0.865200 -0.748464 -1.582807<br>H 0.866794 -1.734170 -0.112844<br>H -0.396947 -0.770393 1.915290<br>H -0.908038 0.777629 1.848907<br>H 1.890326 0.244657 0.812639
OH<br>3769.6; B: 564.2863408	O 0.000000 0.000000 0.107992<br>H 0.000000 0.000000 -0.863937
PRE-H7	C -1.915426 0.699098 -0.889179<br>C -0.818125 0.133175 0.007241

$\tilde{\upsilon}$: 53.4, 100.9, 140.2, 230.3, 249.5, 257.6, 270.8, 306.2, 353.5, 371.1, 416.8, 444.1, 488.2, 554.2, 592.2, 692.4, 780.7, 871.2, 918.2, 950.9, 957.0, 996.4, 1049.2, 1092.2, 1114.3, 1204.2, 1240.1, 1292.3, 1347.1, 1375.1, 1406.1, 1420.5, 1445.6, 1486.5, 1488.9, 1506.6, 1512.5, 1517.9, 1645.7, 3024.6,<br>3049.4, 3058.7, 3110.4, 3121.0, 3125.9, 3130.2, 3141.2, 3513.9, 3527.7, 3598.8, 3746.9<br><br>B: 3.3000000 0.0049596 1.5882542	C -0.032525 1.261766 0.664551<br>C 0.109660 -0.747783 -0.833663<br>N -1.337424 -0.740913 1.071668<br>O 1.092817 -1.374069 -0.030268<br>H -2.493931 -0.104503 -1.348976<br>H -1.491712 1.311961 -1.686384<br>H -2.596234 1.321238 -0.307185<br>H 0.727986 0.869432 1.339211<br>H -0.702731 1.902682 1.240553<br>H 0.462814 1.874663 -0.088537<br>H 0.630909 -0.146377 -1.579342<br>H -0.486312 -1.506144 -1.354446<br>H -2.074530 -1.340466 0.717148<br>H -1.730911 -0.195804 1.828842<br>H 0.634900 -1.628432 0.784461<br>O 2.904255 0.734312 -0.064014<br>H 2.443631 -0.131344 0.013537
SP-H7<br><br>$\tilde{\upsilon}$: -743.8, 83.7, 101.2, 139.2, 159.0, 236.4, 258.7, 304.5, 323.4, 335.2, 372.9, 413.2, 444.8, 533.8, 575.5, 776.4, 793.3, 875.1, 907.2, 918.0, 962.6, 980.2, 1029.7, 1091.7, 1132.4, 1180.5, 1233.8, 1272.5, 1318.8, 1327.5,1351.1, 1377.4, 1402.6, 1435.5, 1444.0, 1480.7, 1493.0, 1500.5, 1513.6, 1651.6, 3003.5, 3060.0, 3078.5, 3097.4, 3130.1, 3147.9, 3151.1, 3507.7, 3598.0, 3779.7, 3781.0<br><br>B: 2.9800000 0.0088480 1.7083193	C -0.938873 1.056992 -0.654097<br>C 0.283206 0.432933 -0.003063<br>C 1.475438 1.376449 -0.136992<br>C 0.595619 -0.908293 -0.678401<br>N 0.078521 0.135141 1.418865<br>O 1.676298 -1.561758 -0.064712<br>H -1.868376 0.407873 -0.377784<br>H -0.884145 1.097170 -1.742440<br>H -1.178410 2.038929 -0.245396<br>H 2.366195 0.910748 0.281264<br>H 1.283063 2.312362 0.390268<br>H 1.663904 1.608169 -1.185122<br>H 0.857337 -0.746931 -1.725463<br>H -0.306675 -1.533658 -0.642627<br>H -0.838946 -0.277342 1.558027<br>H 0.126688 0.976329 1.979981<br>H 1.543963 -1.443944 0.885518<br>O -2.806385 -0.549596 0.111532<br>H -2.765891 -1.153342 -0.647520
POST-H7<br><br>$\tilde{\upsilon}$: 47.4, 82.5, 104.2, 128.0, 140.5, 161.1, 210.0, 244.3, 262.5, 313.5, 327.9, 352.0, 370.9, 410.3, 447.4, 540.0, 576.3, 611.8, 782.0, 890.8, 907.2, 949.5, 981.7, 1020.4, 1079.4, 1129.3, 1189.1, 1233.1, 1292.6, 1339.0, 1371.7, 1398.8, 1434.2, 1456.5, 1489.8, 1495.5, 1512.3, 1607.9, 1655.8, 3007.9, 3059.8, 3097.8, 3132.3, 3143.3, 3148.8, 3248.7, 3504.3, 3599.8, 3778.2, 3818.7, 3940.9<br><br>B: 2.8600000 0.0063291 1.6060853	C -0.459202 1.360378 -0.822522<br>C 0.422868 0.455433 -0.022623<br>C 1.792703 1.113527 0.176767<br>C 0.588246 -0.880022 -0.766076<br>N -0.119377 0.119994 1.299339<br>O 1.384042 -1.781908 -0.042028<br>H -2.579489 0.146255 -0.629295<br>H -0.353476 1.407901 -1.899141<br>H -0.983177 2.167504 -0.328279<br>H 2.448415 0.440758 0.727884<br>H 1.686660 2.045852 0.733439<br>H 2.250803 1.340434 -0.785281<br>H 1.069046 -0.711672 -1.731181<br>H -0.410310 -1.298954 -0.947895<br>H -1.107420 -0.106474 1.232670<br>H -0.015327 0.893031 1.944146<br>H 1.093647 -1.700373 0.876400<br>O -2.989804 -0.308218 0.114466

	H -3.485338 -1.039114 -0.261612
SP-H8<br><br>$\tilde{\upsilon}$: -703.6, 63.6, 79.9, 118.1, 155.6, 234.5, 254.0, 293.4, 322.6, 354.6, 359.4, 418.8, 444.4, 516.5, 568.5, 769.2, 787.8, 858.2, 912.0, 948.2, 971.3, 998.1, 1025.7, 1093.2, 1120.3, 1175.1, 1205.6, 1255.7, 1294.9, 1334.2, 1363.7, 1378.4, 1408.5, 1437.7, 1442.9, 1471.8, 1496.0, 1503.9, 1520.4, 1643.0, 2987.0, 3059.5, 3076.7, 3114.1, 3131.3, 3143.9, 3157.8, 3511.5, 3599.5, 3784.4, 3798.9<br><br>B: 3.0000000 0.0067682 1.6181481	C -0.985599 -0.955151 -0.648135<br>C 0.287595 -0.425657 -0.010064<br>C 0.197430 -0.513130 1.506850<br>C 0.517263 1.022432 -0.452636<br>N 1.468431 -1.184156 -0.452862<br>O 1.727149 1.538507 0.042499<br>H -0.983396 -0.868574 -1.735573<br>H -1.880767 -0.321617 -0.252277<br>H -1.212162 -1.978641 -0.348766<br>H 1.094713 -0.100907 1.963957<br>H 0.085073 -1.552189 1.822967<br>H -0.667824 0.046530 1.860690<br>H -0.292246 1.650671 -0.080461<br>H 0.500820 1.058192 -1.551657<br>H 1.458261 -1.318427 -1.458242<br>H 1.496566 -2.100191 -0.021812<br>H 2.377891 0.831668 -0.051884<br>O -2.889032 0.610494 0.131987<br>H -3.061023 0.979606 -0.748878
POST-H8<br><br>$\tilde{\upsilon}$: 49.3, 82.0, 113.6, 130.4, 153.4, 177.4, 220.5, 249.0, 265.3, 290.0, 311.8, 338.4, 364.4, 407.2, 436.6, 507.7, 568.9, 620.7, 781.9, 879.1, 911.8, 946.9, 988.4, 1008.8, 1089.2, 1123.1, 1176.5, 1242.1, 1260.5, 1349.2, 1378.4, 1418.3, 1441.8, 1459.1, 1495.3, 1505.7, 1516.8, 1611.0, 1640.2, 2988.1, 3059.4, 3114.4, 3133.7, 3140.8, 3159.2, 3245.1, 3505.4, 3592.8, 3796.2, 3810.9, 3939.9<br><br>B: 2.8300000 0.0076883 1.5569662	C -0.474897 -1.338264 -0.819127<br>C 0.424609 -0.453817 -0.020470<br>C 0.015907 -0.428836 1.446766<br>C 0.425011 0.963245 -0.607616<br>N 1.829825 -0.924795 -0.097641<br>O 1.357157 1.795491 0.034986<br>H -0.490329 -1.255401 -1.898949<br>H -2.526233 -0.223586 -0.408534<br>H -0.878561 -2.238921 -0.375724<br>H 0.692700 0.209445 2.011302<br>H 0.048020 -1.436121 1.867247<br>H -0.997968 -0.043582 1.548959<br>H -0.562482 1.408894 -0.485641<br>H 0.641525 0.893487 -1.683367<br>H 2.077169 -1.146268 -1.056635<br>H 1.950127 -1.771518 0.445985<br>H 2.146623 1.257192 0.173201<br>O -3.037695 0.423286 0.089552<br>H -3.808844 0.615760 -0.447980
PRE-H9<br><br>$\tilde{\upsilon}$: 37.0, 93.8, 125.0, 150.2, 158.7, 238.0, 254.6, 288.6, 320.3, 343.2, 372.9, 379.1, 420.5, 446.2, 523.3, 573.6, 779.4, 833.8, 912.0, 950.4, 959.8, 995.0, 1045.6, 1088.8, 1128.9, 1203.8, 1231.4, 1294.9, 1347.0, 1373.6, 1396.2, 1417.0, 1441.5, 1487.7, 1489.3, 1509.2, 1512.8, 1517.8, 1637.6, 2993.9, 3036.1, 3059.6, 3099.5, 3106.5, 3116.8, 3128.3, 3155.6, 3508.1, 3596.4, 3765.4, 3791.6<br><br>B: 3.4400000 0.0051580 1.3963825	C -0.647263 1.183528 -0.903951<br>C 0.242677 0.336246 0.004990<br>C 0.760000 1.176735 1.165481<br>C 1.412132 -0.215112 -0.818576<br>N -0.443963 -0.833752 0.561743<br>O 2.249519 -1.048316 -0.057667<br>H -1.074716 0.573278 -1.703466<br>H -0.081448 1.993759 -1.366860<br>H -1.455298 1.635668 -0.323901<br>H 1.440669 0.594913 1.783604<br>H -0.070203 1.522106 1.784637<br>H 1.291876 2.051908 0.792507<br>H 2.021145 0.607790 -1.195653<br>H 1.004955 -0.756993 -1.683666

	H -0.950630 -1.356975 -0.144593<br>H -1.095349 -0.590599 1.298193<br>H 1.662298 -1.588982 0.486053<br>O -3.143319 -0.682599 0.074346<br>H -3.040438 0.109324 -0.480154
SP-H9<br><br>ṽ: -767.7, 68.4, 83.0, 134.7, 151.4, 236.6, 240.5, 298.0, 328.2, 349.5, 375.8, 410.6, 443.4, 524.4, 571.0, 750.3, 792.4, 889.7, 896.7, 941.4, 964.0, 996.3, 1027.5, 1088.2, 1125.1, 1190.3, 1208.5, 1258.2, 1305.6, 1333.0, 1365.0, 1377.8, 1414.2, 1438.0, 1453.1, 1485.6, 1498.1, 1506.4, 1515.9, 1652.1, 2996.7, 3065.6, 3078.1, 3102.1, 3134.7, 3147.0, 3158.9, 3502.8, 3584.4, 3785.5, 3787.3<br><br>B: 4.4100000 0.0051494 1.3325021	C -0.892919 1.000274 0.612484<br>C 0.172140 0.049304 0.093386<br>C 0.078712 -1.288840 0.816157<br>C 1.554830 0.684363 0.306256<br>N 0.055807 -0.198336 -1.348474<br>O 2.582016 -0.125420 -0.205925<br>H -0.911591 1.951899 0.080358<br>H -0.853069 1.161925 1.689981<br>H -1.915411 0.474834 0.412385<br>H 0.861904 -1.959884 0.470373<br>H -0.893357 -1.747590 0.630017<br>H 0.190820 -1.148126 1.891204<br>H 1.741715 0.823668 1.371809<br>H 1.563306 1.673708 -0.171760<br>H -0.038527 0.678369 -1.851012<br>H -0.781862 -0.737809 -1.537924<br>H 2.249422 -0.478011 -1.041664<br>O -3.035100 -0.195367 -0.160501<br>H -3.455912 0.591048 -0.542742
POST-H9<br><br>ṽ: 64.9, 92.6, 117.5, 127.0, 144.8, 164.3, 215.2, 246.9, 269.7, 306.1, 330.9, 363.1, 374.1, 413.8, 442.6, 541.8, 587.4, 603.5, 784.3, 916.0, 923.3, 931.8, 994.1, 1015.6, 1080.9, 1120.4, 1198.9, 1211.4, 1278.4, 1342.7, 1375.7, 1411.6, 1434.5, 1460.1, 1495.2, 1502.8, 1513.9, 1611.3, 1655.2, 3002.9, 3066.0, 3104.9, 3136.3, 3143.9, 3158.9, 3247.9, 3504.7, 3583.6, 3776.5, 3810.8, 3939.5<br><br>B: 4.0800000 0.0009448 1.2955202	C -0.536249 1.459390 -0.404691<br>C 0.240261 0.249349 0.004958<br>C 0.041361 -0.043235 1.489793<br>C 1.736354 0.495103 -0.288870<br>N -0.117473 -0.957921 -0.755297<br>O 2.512961 -0.635986 0.011314<br>H -0.732625 1.637834 -1.454253<br>H -0.663238 2.287824 0.279415<br>H -2.699003 0.430269 -0.124715<br>H 0.679188 -0.869792 1.795133<br>H -0.998502 -0.307116 1.685583<br>H 0.298088 0.833252 2.084925<br>H 2.102728 1.324828 0.316590<br>H 1.840217 0.772896 -1.346224<br>H -0.136722 -0.756496 -1.749627<br>H -1.047983 -1.271677 -0.499358<br>H 1.993680 -1.395347 -0.286104<br>O -3.102475 -0.443516 -0.072970<br>H -3.987775 -0.308645 0.271822
PRE-H10<br><br>ṽ: 64.3, 95.8, 134.1, 193.3, 237.6, 243.4, 264.2, 311.0, 347.5, 369.4, 416.9, 446.0, 472.3, 527.1, 579.5, 708.7, 786.6, 890.3, 926.8, 950.6, 969.4, 995.9, 1025.8, 1090.7, 1121.1, 1193.9, 1283.9, 1288.3, 1332.5, 1396.8, 1412.7, 1417.1, 1431.2, 1481.4, 1488.7, 1504.1, 1508.4, 1510.5, 1655.9, 3047.5, 3050.8, 3056.3, 3116.7, 3121.0, 3125.5, 3129.5, 3134.6, 3505.7, 3536.3, 3596.9, 3806.3	C 2.300242 -0.110326 0.019798<br>C 0.778002 -0.136535 0.007164<br>C 0.283348 -1.248723 -0.917199<br>C 0.274253 1.232806 -0.473727<br>N 0.292477 -0.300806 1.385997<br>O -1.077163 1.463974 -0.100877<br>H 2.662305 0.607890 0.755934<br>H 2.687676 0.172626 -0.959704<br>H 2.698810 -1.095701 0.266312<br>H -0.806416 -1.255730 -0.988917

B: 3.2100000 0.0078350 1.6906761	H 0.598113 -2.221035 -0.536397<br>H 0.685395 -1.126310 -1.924194<br>H 0.318420 1.300777 -1.560388<br>H 0.904936 2.018089 -0.048231<br>H 0.912013 -0.903451 1.912617<br>H -0.622824 -0.740868 1.388843<br>H -1.075699 1.480679 0.864417<br>O -2.733733 -0.793494 0.036733<br>H -2.337969 0.081508 -0.175344
SP-H10<br>$\tilde{\upsilon}$: -1052.0, 83.8, 138.7, 190.9, 219.7, 250.1, 285.2, 308.7, 359.4, 387.5, 414.2, 449.0, 476.0, 549.0, 640.0, 758.9, 804.4, 884.9, 912.0, 961.6, 985.3, 1002.3, 1024.3, 1042.1, 1113.2, 1139.0, 1200.1, 1265.0, 1293.0, 1315.2, 1337.3, 1399.4, 1407.1, 1419.1, 1426.5, 1463.0, 1495.0, 1503.3, 1508.5, 1654.3, 3048.6, 3056.0, 3083.3, 3117.7, 3130.1, 3134.6, 3157.1, 3510.0, 3601.6, 3663.5, 3818.6<br><br>B: 3.0300000 0.0017141 1.9320859	C 2.209120 -0.272269 -0.202417<br>C 0.703770 -0.140714 0.007449<br>C -0.009322 -1.273231 -0.711257<br>C 0.263245 1.227188 -0.537389<br>N 0.412543 -0.140328 1.447916<br>O -1.020150 1.604909 -0.072086<br>H 2.735152 0.479751 0.386939<br>H 2.465582 -0.131296 -1.252800<br>H 2.555475 -1.261009 0.101038<br>H -1.175853 -1.218553 -0.475981<br>H 0.279845 -2.252670 -0.330567<br>H 0.078150 -1.231133 -1.796318<br>H 0.211064 1.207603 -1.626081<br>H 1.000438 1.977206 -0.237083<br>H 1.076902 -0.712774 1.952482<br>H -0.510103 -0.529268 1.615690<br>H -0.944690 1.670710 0.887372<br>O -2.402299 -0.895727 0.000992<br>H -2.281050 0.064431 -0.129668
POST-H10<br><br>$\tilde{\upsilon}$: 77.4, 105.4, 125.3, 148.8, 166.2, 197.3, 254.9, 268.9, 307.0, 324.5, 332.4, 378.5, 419.3, 458.7, 501.9, 504.5, 588.9, 644.4, 805.0, 895.6, 918.4, 963.1, 982.0, 1009.9, 1055.0, 1112.6, 1172.0, 1272.4, 1284.9, 1321.0, 1395.0, 1401.5, 1413.3, 1461.6, 1491.3, 1499.6, 1507.9, 1636.2, 1664.1, 3052.0, 3056.6, 3118.3, 3128.5, 3135.5, 3155.5, 3261.7, 3497.7, 3598.7, 3733.5, 3822.7, 3929.8<br><br>B: 3.2000000 0.0007666 1.6853969	C 2.325644 -0.308166 0.047630<br>C 0.801014 -0.180465 -0.025037<br>C 0.266627 -1.034323 -1.131982<br>C 0.456918 1.294408 -0.273140<br>N 0.236895 -0.535555 1.284831<br>O -0.915107 1.565204 -0.046635<br>H 2.704143 0.249294 0.905938<br>H 2.787906 0.082166 -0.859069<br>H 2.614894 -1.354549 0.150787<br>H -2.777006 -1.292533 -0.630756<br>H 0.143171 -2.095728 -0.968784<br>H 0.211632 -0.659933 -2.144667<br>H 0.665889 1.565277 -1.308220<br>H 1.072867 1.914970 0.382917<br>H 0.761738 -1.288030 1.711538<br>H -0.724464 -0.848162 1.181169<br>H -1.059887 1.460492 0.901115<br>O -2.703487 -0.653404 0.080759<br>H -2.211614 0.092504 -0.293603
PRE-H11<br><br>$\tilde{\upsilon}$: 31.2, 107.3, 159.7, 202.7, 240.7, 257.4, 281.1, 314.0, 351.4, 387.1, 410.5, 425.1, 449.5, 562.2, 658.8, 771.8, 875.5, 914.7, 950.3, 956.3, 990.4, 1002.6, 1050.1, 1115.0, 1147.1, 1203.2, 1240.2, 1302.7, 1365.2, 1394.7, 1407.1, 1425.0,	C 2.188672 0.281826 -0.535281<br>C 0.770818 0.127544 0.011457<br>C 0.783556 0.058885 1.530998<br>C 0.156743 -1.147419 -0.582674<br>N -0.077122 1.275909 -0.385563<br>O -1.022432 -1.582810 0.038411

1437.7, 1482.7, 1486.9, 1499.7, 1504.9, 1514.7, 1639.6, 2998.7, 3048.8, 3059.8, 3098.5, 3118.0, 3128.7, 3132.6, 3145.4, 3155.6, 3490.1, 3567.9, 3759.8<br><br>B: 3.1100000 0.0039161 1.6862696	H 2.186173 0.279826 -1.627717<br>H 2.829848 -0.532135 -0.196279<br>H 2.626243 1.222368 -0.197490<br>H -0.229907 -0.019645 1.919356<br>H 1.258999 0.948569 1.948924<br>H 1.342586 -0.815065 1.864156<br>H 0.878848 -1.957478 -0.468723<br>H 0.009356 -0.990923 -1.660338<br>H 0.025503 1.445407 -1.382247<br>H 0.247814 2.117250 0.080643<br>H -1.740174 -0.953967 -0.116436<br>O -2.784895 0.750049 -0.026887<br>H -1.835554 1.061493 -0.104091
SP-H11<br><br>ῦ: -725.9, 61.8, 114.3, 150.8, 173.6, 234.7, 260.7, 297.2, 349.2, 368.2, 371.9, 407.2, 448.9, 529.6, 574.1, 756.2, 788.7, 902.0, 916.1, 943.7, 961.7, 970.6, 1028.9, 1106.0, 1127.9, 1204.8, 1215.6, 1246.7, 1312.1, 1336.8, 1362.6, 1384.5, 1409.4, 1435.9, 1454.0, 1498.6, 1501.5, 1515.7, 1553.1, 1654.6, 2997.0, 3050.8, 3100.7, 3102.9, 3122.1, 3131.9, 3175.7, 3504.6, 3584.4, 3782.3, 3784.7<br><br>B: 3.4800000 0.0054534 1.5245901	C 0.207764 1.808863 -0.148012<br>C -0.213015 0.348939 -0.003174<br>C 0.539830 -0.518353 -0.992019<br>C -1.722974 0.218448 -0.253816<br>N 0.028120 -0.191390 1.340933<br>O -2.176640 -1.096733 -0.059475<br>H -0.313202 2.430500 0.582069<br>H -0.023347 2.188722 -1.144848<br>H 1.281101 1.907523 0.017872<br>H 0.247919 -1.564341 -0.937611<br>H 1.664805 -0.452766 -0.696999<br>H 0.484925 -0.145552 -2.013723<br>H -1.959824 0.504563 -1.279452<br>H -2.240366 0.916009 0.419370<br>H -0.312963 0.456472 2.042942<br>H 1.025389 -0.303070 1.491779<br>H -1.713955 -1.418823 0.725431<br>O 2.896261 -0.510939 0.041193<br>H 3.036079 -1.465514 -0.064975
POST-H11<br><br>ῦ: 63.1, 107.4, 126.1, 150.3, 161.9, 198.1, 245.5, 253.8, 268.3, 320.2, 344.4, 371.3, 398.1, 412.8, 453.3, 528.9, 562.7, 664.8, 793.2, 910.3, 922.4, 945.1, 965.7, 1011.9, 1110.0, 1129.0, 1193.1, 1219.7, 1278.6, 1335.6, 1376.7, 1406.4, 1432.5, 1458.8, 1496.0, 1500.4, 1514.6, 1613.1, 1662.4, 2991.9, 3051.0, 3104.0, 3122.2, 3135.0, 3160.4, 3267.4, 3499.5, 3577.4, 3782.5, 3803.1, 3935.6<br><br>B: 3.7400000 0.0093043 1.3596243	C 0.293679 1.714419 0.073386<br>C -0.257705 0.290939 -0.025961<br>C 0.212312 -0.370600 -1.278732<br>C -1.799939 0.346268 -0.027371<br>N 0.106699 -0.556596 1.118597<br>O -2.368768 -0.936863 -0.057709<br>H -0.083042 2.203062 0.973436<br>H -0.005315 2.312118 -0.789623<br>H 1.382504 1.693415 0.123925<br>H 0.164989 -1.446964 -1.357773<br>H 2.533816 -0.487557 -0.724796<br>H 0.270162 0.209087 -2.189526<br>H -2.154074 0.891135 -0.903049<br>H -2.118384 0.897218 0.869000<br>H -0.110778 -0.071171 1.982773<br>H 1.107154 -0.729731 1.119308<br>H -1.822629 -1.479194 0.526538<br>O 3.063042 -0.439373 0.079893<br>H 3.844423 -0.971506 -0.085797
PRE-H12 = PRE-H13	C 1.915248 -0.699110 -0.889168<br>C 0.817978 -0.133137 0.007236

$\tilde{\upsilon}$: 53.4, 101.2, 140.2, 230.4, 249.5, 257.5, 270.8, 306.2, 353.6, 371.1, 416.8, 444.1, 488.3, 554.2, 592.2, 692.6, 780.7, 871.1, 918.2, 950.9, 957.0, 996.4, 1049.2, 1092.2, 1114.3, 1204.2, 1240.1, 1292.3, 1347.1, 1375.1, 1406.1, 1420.5, 1445.6, 1486.5, 1488.9, 1506.6, 1512.5, 1517.9, 1645.7, 3024.7, 3049.5, 3058.7, 3110.4, 3121.1, 3126.0, 3130.3, 3141.3, 3513.9, 3527.3, 3598.8, 3747.0<br><br>B: 3.3000000 0.0048582 1.5887383	C 0.032369 -1.261668 0.664611<br>C -0.109809 0.747776 -0.833694<br>N 1.337301 0.740979 1.071627<br>O -1.092946 1.374100 -0.030303<br>H 2.493801 0.104471 -1.348918<br>H 1.491525 -1.311935 -1.686390<br>H 2.596008 -1.321291 -0.307177<br>H -0.727904 -0.869275 1.339500<br>H 0.702602 -1.902737 1.240395<br>H -0.463287 -1.874388 -0.088402<br>H -0.631084 0.146313 -1.579298<br>H 0.486150 1.506082 -1.354548<br>H 2.074659 1.340250 0.717174<br>H 1.730445 0.195880 1.828974<br>H -0.634958 1.628680 0.784306<br>O -2.903373 -0.734529 -0.064039<br>H -2.443227 0.131368 0.013820
SP-H12<br><br>$\tilde{\upsilon}$: -1240.7, 47.3, 118.3, 192.0, 208.1, 250.3, 272.8, 309.2, 360.4, 377.5, 422.0, 434.2, 474.2, 557.5, 665.4, 752.0, 787.2, 829.3, 858.8, 934.8, 950.9, 990.8, 1021.2, 1061.0, 1097.5, 1125.4, 1195.1, 1223.7, 1275.7, 1318.3, 1353.2, 1385.0, 1401.7, 1430.1, 1443.8, 1467.7, 1495.5, 1498.5, 1516.0, 1646.3, 3016.6, 3049.6, 3086.1, 3107.2, 3121.4, 3132.6, 3164.3, 3530.7, 3617.3, 3689.6, 3732.4<br><br>B: 3.0900000 0.0086265 1.8293618	C -2.171574 0.400089 -0.442922<br>C -0.732281 0.146061 0.012430<br>C 0.128524 1.318003 -0.411056<br>C -0.236037 -1.163953 -0.622176<br>N -0.623200 -0.036816 1.466356<br>O 0.997146 -1.572910 -0.075137<br>H -2.824180 -0.405976 -0.101743<br>H -2.234666 0.449525 -1.531012<br>H -2.541500 1.340477 -0.034002<br>H -0.130457 2.241698 0.107695<br>H 0.123468 1.469417 -1.488250<br>H 1.286589 1.148823 -0.132647<br>H -0.099737 -1.036020 -1.696573<br>H -0.995513 -1.939421 -0.464818<br>H -1.452230 -0.480001 1.843101<br>H -0.494897 0.843240 1.948002<br>H 0.914000 -1.401426 0.875502<br>O 2.562014 0.817579 -0.004817<br>H 2.406448 -0.131174 -0.167766
POST-H12<br><br>$\tilde{\upsilon}$: 44.1, 82.1, 126.3, 139.0, 179.5, 224.8, 252.2, 259.2, 285.7, 343.8, 363.8, 381.0, 410.9, 447.6, 530.6, 551.5, 614.4, 664.5, 794.3, 874.5, 927.2, 937.0, 962.3, 1010.6, 1091.7, 1117.3, 1186.7, 1213.8, 1277.1, 1329.0, 1375.9, 1402.4, 1437.5, 1462.3, 1492.0, 1499.2, 1514.0, 1640.6, 1646.2, 3014.4, 3051.3, 3117.0, 3124.7, 3133.7, 3177.4, 3284.7, 3510.9, 3596.4, 3705.6, 3757.8, 3933.7<br><br>B: 3.3700000 0.0022014 1.5072851	C 1.916859 -0.922615 -0.802541<br>C 0.889978 -0.175366 0.049556<br>C 0.112888 -1.131049 0.889642<br>C -0.053381 0.607177 -0.884405<br>N 1.491958 0.836327 0.934445<br>O -0.973369 1.383776 -0.145029<br>H 2.487013 -0.223849 -1.417587<br>H 1.424982 -1.637992 -1.463160<br>H 2.610048 -1.468812 -0.162868<br>H -0.329678 -0.800121 1.816960<br>H -0.237633 -2.054982 0.456244<br>H -3.786998 -0.382554 -0.205306<br>H -0.626347 -0.083833 -1.502596<br>H 0.548206 1.251219 -1.537938<br>H 2.202354 1.363041 0.437834<br>H 1.942440 0.390743 1.724633<br>H -0.473939 1.726954 0.609906<br>O -2.905641 -0.675478 0.032226

	H -2.370137 0.130626 0.091678
$(CH_3)(CH_2OH)(NH_2)C\dot{C}H_2$<br><br>$\tilde{\nu}$: 143.2, 167.1, 248.2, 255.0, 281.3, 344.5, 366.4, 404.7, 445.0, 513.5, 529.1, 603.8, 791.4, 871.8, 919.6, 937.8, 959.2, 1009.9, 1096.0, 1130.4, 1192.1, 1213.3, 1269.8, 1331.2, 1378.0, 1403.7, 1432.9, 1462.3, 1492.4, 1499.4, 1513.0, 1641.2, 2988.4, 3051.5, 3106.2, 3124.4, 3130.8, 3170.4, 3278.6, 3510.3, 3597.3, 3792.6<br><br>B: 4.7179851 2.7572390 2.7116290	C 1.667865 -0.799616 -0.157911<br>C 0.410506 0.048114 0.036793<br>C 0.443990 0.741319 1.355212<br>C -0.828718 -0.866607 -0.054554<br>N 0.226590 1.063887 -1.013604<br>O -2.025088 -0.141592 0.056045<br>H 1.643675 -1.303347 -1.125725<br>H 1.749514 -1.560085 0.620418<br>H 2.557679 -0.171029 -0.116644<br>H 0.900441 0.253118 2.203262<br>H -0.195786 1.593203 1.528115<br>H -0.807076 -1.600793 0.751208<br>H -0.783502 -1.407559 -1.010756<br>H 0.390992 0.657025 -1.928198<br>H 0.889651 1.820221 -0.894396<br>H -1.892868 0.665514 -0.457667
H_2O<br>1616.2, 3871.3, 3975.3<br>B: 834.0336966 430.9641048 284.1416681	O 0.000000 0.000000 0.116362<br>H 0.000000 0.762747 -0.465449<br>H 0.000000 -0.762747 -0.465449
SP-H13<br><br>$\tilde{\nu}$: -934.8, 63.7, 90.4, 153.5, 230.0, 253.4, 267.5, 286.1, 294.3, 340.1, 371.8, 413.0, 431.9, 550.9, 581.7, 728.0, 786.4, 876.1, 915.6, 932.3, 956.8, 987.1, 1028.5, 1070.4, 1096.6, 1196.4, 1243.2, 1272.0, 1312.8, 1357.9, 1386.4, 1392.9, 1411.8, 1431.9, 1488.8, 1494.0, 1508.0, 1520.2, 1653.5, 1721.9, 3028.0, 3057.2, 3067.9, 3135.6, 3141.9, 3146.2, 3171.7, 3529.0, 3624.4, 3732.3, 3771.9<br><br>B: 2.9900000 0.0021535 1.8134450	C 1.274522 -1.319307 -0.833128<br>C 0.740505 -0.155745 0.002857<br>C 0.388000 -0.627677 1.414078<br>C -0.499353 0.433434 -0.682184<br>N 1.688562 0.966172 0.125594<br>O -0.948190 1.604956 -0.074169<br>H 1.570319 -0.981006 -1.832424<br>H 0.511250 -2.096341 -0.948468<br>H 2.148711 -1.766869 -0.349481<br>H -0.026584 0.194311 2.004195<br>H 1.283273 -1.004871 1.920909<br>H -0.354316 -1.428134 1.371695<br>H -1.364268 -0.344943 -0.591131<br>H -0.352609 0.567777 -1.765952<br>H 2.135613 1.167470 -0.766053<br>H 2.423109 0.750912 0.793745<br>H -0.157037 2.026982 0.304851<br>O -2.582724 -0.836930 -0.026455<br>H -2.812126 0.023064 0.374208
POST-H13<br><br>$\tilde{\nu}$: 32.8, 64.5, 119.8, 136.3, 204.1, 231.8, 254.4, 268.8, 298.2, 311.7, 353.0, 366.9, 408.2, 457.2, 513.1, 577.0, 685.4, 733.4, 792.9, 887.3, 929.1, 943.2, 979.8, 1025.5, 1090.1, 1183.1, 1223.1, 1265.0, 1301.5, 1342.6, 1397.8, 1416.6, 1458.2, 1479.8, 1483.3, 1501.0, 1510.3, 1628.3, 1639.4, 3049.5, 3053.2, 3125.6, 3128.0, 3130.2, 3151.7, 3206.8, 3515.2, 3604.2, 3658.8, 3768.3, 3940.5<br><br>B: 3.3800000 0.0021024 1.4662802	C 2.026126 -0.636779 -0.912530<br>C 0.906848 -0.118099 -0.015084<br>C 0.224326 -1.282259 0.713110<br>C -0.096823 0.636071 -0.841882<br>N 1.373648 0.833479 1.009303<br>O -0.973469 1.416479 -0.148193<br>H 2.543732 0.192712 -1.396020<br>H 1.624739 -1.286793 -1.691530<br>H 2.746681 -1.209546 -0.327307<br>H -0.587157 -0.920605 1.343670<br>H 0.946759 -1.815955 1.335498<br>H -0.199385 -1.981926 -0.005357<br>H -3.879613 -0.408874 -0.174839

	H -0.520221 0.193960 -1.733521<br>H 2.045157 1.480418 0.610523<br>H 1.827372 0.348095 1.773020<br>H -0.515346 1.635841 0.682626<br>O -2.968678 -0.646378 0.005984<br>H -2.473941 0.183908 -0.005898
PRE-H14<br><br>ῦ: 53.5, 101.1, 140.4, 230.4, 249.6, 257.5, 270.8, 306.2, 353.6, 371.1, 416.8, 444.1, 488.4, 554.2, 592.3, 692.6, 780.7, 871.1, 918.2, 950.9, 957.0, 996.4, 1049.2, 1092.1, 1114.1, 1204.1, 1240.1, 1292.2, 1347.1, 1375.0, 1406.1, 1420.5, 1445.6, 1486.5, 1488.9, 1506.6, 1512.5, 1517.9, 1645.7, 3024.5, 3049.5, 3058.7, 3110.4, 3121.1, 3126.0, 3130.3, 3141.3, 3513.9, 3527.3, 3598.8, 3747.1<br><br>B: 3.3000000 0.0050544 1.5885688	C 1.915324 0.699203 0.889096<br>C 0.818032 0.133141 -0.007224<br>C 0.032498 1.261597 -0.664818<br>C -0.109830 -0.747578 0.833822<br>N 1.337327 -0.741198 -1.071452<br>O -1.092934 -1.373970 0.030276<br>H 2.493814 -0.104336 1.348996<br>H 1.491635 1.312207 1.686199<br>H 2.596136 1.321228 0.306997<br>H -0.727758 0.869110 -1.339666<br>H 0.702775 1.902543 -1.240687<br>H -0.463158 1.874461 0.088080<br>H -0.631136 -0.145986 1.579298<br>H 0.486102 -1.505843 1.354816<br>H 2.074616 -1.340478 -0.716867<br>H 1.730560 -0.196257 -1.828868<br>H -0.634752 -1.628591 -0.784210<br>O -2.903668 0.734424 0.064003<br>H -2.443455 -0.131483 -0.013405
SP-H14<br><br>ῦ: -703.9, 64.7, 107.8, 138.9, 226.1, 242.8, 257.5, 281.8, 311.7, 347.8, 375.4, 404.6, 451.4, 565.7, 620.4, 678.3, 785.5, 876.3, 893.8, 931.1, 955.2, 989.6, 1031.4, 1110.0, 1134.3, 1200.0, 1236.6, 1288.3, 1340.8, 1366.6, 1400.9, 1418.2, 1421.7, 1454.0, 1487.3, 1488.1, 1504.1, 1515.0, 1635.4, 2015.7, 3050.3, 3059.2, 3095.3, 3121.8, 3129.3, 3131.0, 3150.4, 3508.3, 3601.3, 3712.1, 3767.3<br><br>B: 2.9000000 0.0026961 1.9369139	C -0.798832 -1.600077 -0.596029<br>C -0.687480 -0.204663 0.010529<br>C -2.024941 0.524787 -0.059677<br>C 0.379657 0.588786 -0.746226<br>N -0.240443 -0.215099 1.409395<br>O 0.680709 1.804327 -0.141974<br>H 0.151929 -2.128053 -0.512161<br>H -1.072514 -1.547987 -1.651571<br>H -1.564340 -2.176984 -0.075496<br>H -1.931876 1.537818 0.328477<br>H -2.774964 -0.007795 0.528183<br>H -2.377687 0.576052 -1.089473<br>H 0.105301 0.762515 -1.788248<br>H 1.303840 -0.078834 -0.766879<br>H 0.569842 -0.819106 1.508137<br>H -0.970368 -0.558328 2.020815<br>H 0.549786 1.654458 0.807702<br>O 2.517659 -0.763626 0.014621<br>H 2.896776 0.113332 0.191995
POST-H14<br><br>ῦ: 66.6, 104.3, 124.0, 143.0, 170.7, 236.6, 256.0, 278.3, 290.2, 340.3, 355.8, 377.2, 407.3, 451.0, 490.7, 580.5, 698.1, 749.3, 793.6, 900.3, 933.0, 946.4, 984.7, 1024.2, 1104.7, 1190.0, 1228.7, 1270.5, 1314.2, 1359.6, 1394.9, 1415.4, 1460.5, 1484.6, 1486.4, 1503.4, 1513.2, 1618.2, 1648.2, 3049.4, 3053.0, 3120.0, 3125.0, 3137.4, 3146.3, 3192.0, 3498.9, 3602.5, 3639.3, 3734.4, 3927.1	C -0.453563 -1.621550 -0.681254<br>C -0.668472 -0.269749 -0.004126<br>C -2.168126 0.028181 0.118233<br>C 0.001701 0.818424 -0.805657<br>N -0.087718 -0.190160 1.345046<br>O 0.226948 1.987687 -0.163265<br>H 0.605387 -1.879759 -0.693722<br>H -0.817379 -1.599466 -1.710329<br>H -0.997276 -2.400805 -0.145172

B: 2.7800000 0.0078568 1.8352465	H -2.325492 1.008029 0.567970<br>H -2.655817 -0.725798 0.740566<br>H -2.638834 0.014084 -0.864021<br>H -0.210799 0.927873 -1.861776<br>H 2.126625 0.036967 -0.519622<br>H 0.848727 -0.584630 1.348367<br>H -0.654417 -0.698177 2.011561<br>H 0.223826 1.759589 0.784498<br>O 2.690134 -0.517200 0.038771<br>H 3.503588 -0.022514 0.159128
$(CH_3)_2(NH_2)C\dot{C}HOH$<br><br>$\tilde{\upsilon}$: 146.3, 228.6, 249.6, 269.1, 306.8, 347.0, 364.5, 405.5, 458.4, 572.8, 638.4, 691.7, 789.4, 884.2, 927.3, 940.5, 978.3, 1021.0, 1092.6, 1186.4, 1227.6, 1263.4, 1307.9, 1353.7, 1392.6, 1411.6, 1456.9, 1481.1, 1485.2, 1499.7, 1511.8, 1639.0, 3048.4, 3052.2, 3121.1, 3126.0, 3128.8, 3147.9, 3203.6, 3511.7, 3599.8, 3711.6<br><br>B: 4.6649330 2.7363275 2.7095601	C -1.561274 -0.960087 -0.143738<br>C -0.369262 -0.018808 0.005992<br>C -0.597859 1.257385 -0.813881<br>C 0.884918 -0.715459 -0.442083<br>N -0.124547 0.394814 1.398750<br>O 2.061006 -0.133559 -0.106001<br>H -1.427567 -1.844620 0.479967<br>H -1.667198 -1.288159 -1.179159<br>H -2.483193 -0.456631 0.151038<br>H 0.267512 1.914886 -0.740022<br>H -1.478298 1.794485 -0.452573<br>H -0.757898 1.008004 -1.861968<br>H 0.910387 -1.247733 -1.383362<br>H -0.168613 -0.410402 2.014081<br>H -0.825547 1.057193 1.707193<br>H 1.875056 0.399572 0.683818
PRE-H15<br><br>$\tilde{\upsilon}$: 54.3, 69.6, 148.2, 195.0, 231.4, 251.6, 280.5, 300.7, 342.2, 356.5, 383.3, 414.0, 451.7, 507.9, 564.4, 713.9, 782.3, 869.2, 922.1, 950.0, 965.2, 994.4, 1054.6, 1095.6, 1112.8, 1221.7, 1245.3, 1295.4, 1336.7, 1373.3, 1406.1, 1421.8, 1454.6, 1484.8, 1489.1, 1505.3, 1512.6, 1524.0, 1637.1, 3029.7, 3049.7, 3059.6, 3098.7, 3121.7, 3126.6, 3129.5, 3145.8, 3506.2, 3599.6, 3608.8, 3699.1<br><br>B: 3.3500000 0.0022732 1.5766759	C -1.562240 -1.202528 -0.792478<br>C -0.825601 -0.133223 0.008588<br>C -1.812353 0.879383 0.579468<br>C 0.189279 0.566174 -0.903005<br>N -0.043743 -0.678949 1.126439<br>O 1.069002 1.393484 -0.159917<br>H -0.866497 -1.963537 -1.148700<br>H -2.064473 -0.766398 -1.658052<br>H -2.316452 -1.689166 -0.172636<br>H -1.289638 1.687315 1.089915<br>H -2.482294 0.396445 1.293169<br>H -2.419890 1.307797 -0.217222<br>H -0.329991 1.183971 -1.637172<br>H 0.765694 -0.195666 -1.440248<br>H 0.548265 -1.444468 0.818791<br>H -0.646729 -1.026231 1.861583<br>H 0.999729 1.076632 0.755075<br>O 2.911702 -0.724854 0.083251<br>H 2.628347 0.178078 -0.171689
SP-H15<br><br>$\tilde{\upsilon}$: -490.4, 55.8, 131.5, 154.1, 168.2, 232.9, 245.8, 281.8, 310.1, 349.8, 401.3, 411.9, 439.1, 455.8, 558.7, 697.9, 737.4, 784.3, 918.1, 945.4, 954.6, 992.2, 1041.0, 1086.3, 1123.1, 1186.3, 1211.5, 1291.0, 1339.1, 1377.8, 1392.4, 1409.1, 1433.8, 1482.8, 1488.2, 1500.8, 1510.1, 1517.7, 1564.6,	C 0.467376 -1.420493 -0.910803<br>C -0.294766 -0.453237 -0.004418<br>C -1.466071 -1.173084 0.662301<br>C -0.785696 0.734048 -0.834569<br>N 0.533865 0.107047 1.063364<br>O -1.531233 1.658238 -0.076850<br>H 1.282005 -0.904181 -1.417366<br>H -0.200426 -1.847337 -1.660247

2373.2, 3025.8, 3052.5, 3060.7, 3109.8, 3121.5, 3130.3, 3143.6, 3160.1, 3550.6, 3805.7, 3838.7<br><br>B: 2.8100000 0.0058902 1.8285998	H 0.882710 -2.241302 -0.322979<br>H -2.037343 -0.486135 1.282359<br>H -1.104131 -1.995151 1.282645<br>H -2.127117 -1.587784 -0.098482<br>H -1.438947 0.375335 -1.630450<br>H 0.085050 1.214829 -1.296829<br>H 1.362760 0.677408 0.730603<br>H 0.856197 -0.606576 1.708924<br>H -1.019746 1.858593 0.714240<br>O 2.670669 0.696779 0.030163<br>H 3.081382 -0.170573 0.162469
POST-H15<br><br>$\tilde{\upsilon}$: 50.8, 128.3, 169.9, 191.8, 199.2, 227.7, 247.2, 257.6, 286.5, 339.7, 367.1, 411.2, 419.7, 441.3, 561.2, 633.1, 738.0, 786.2, 914.1, 937.4, 951.3, 989.9, 1016.9, 1137.9, 1154.6, 1171.4, 1197.9, 1304.8, 1349.8, 1386.4, 1398.1, 1424.2, 1444.4, 1478.4, 1486.3, 1496.6, 1499.8, 1515.4, 1623.2, 3024.2, 3056.4, 3059.1, 3088.7, 3127.1, 3132.2, 3136.2, 3159.5, 3435.4, 3575.1, 3729.8, 3935.2<br><br>B: 3.2300000 0.0073162 1.6007320	C 1.902120 -0.773380 0.885989<br>C 0.827698 -0.143648 -0.007567<br>C 1.470728 0.585749 -1.190530<br>C -0.018329 0.828525 0.823807<br>N -0.031529 -1.198575 -0.506159<br>O -0.969477 1.525016 0.064710<br>H 1.448386 -1.315260 1.715956<br>H 2.546911 0.006637 1.291751<br>H 2.521697 -1.465989 0.315263<br>H 0.702655 1.015415 -1.829560<br>H 2.090423 -0.096979 -1.773765<br>H 2.101767 1.394667 -0.820479<br>H 0.647201 1.568814 1.273321<br>H -0.492217 0.265168 1.636121<br>H -1.930414 -1.089967 -0.257340<br>H 0.539053 -1.819620 -1.085226<br>H -1.755168 0.966647 -0.030882<br>O -2.785280 -0.638512 -0.129576<br>H -3.254838 -1.115010 0.556691
PRE-H16<br><br>$\tilde{\upsilon}$: 36.8, 93.7, 125.0, 148.6, 158.8, 238.0, 254.5, 287.8, 320.2, 343.4, 373.0, 379.2, 420.5, 446.2, 523.3, 573.6, 779.4, 833.7, 912.0, 950.4, 959.8, 995.0, 1045.6, 1088.8, 1128.9, 1203.8, 1231.4, 1294.9, 1346.9, 1373.5, 1396.2, 1417.0, 1441.5, 1487.7, 1489.3, 1509.2, 1512.8, 1517.8, 1637.6, 2993.8, 3036.2, 3059.6, 3099.5, 3106.5, 3116.8, 3128.3, 3155.6, 3508.0, 3596.3, 3766.0, 3791.6<br><br>B: 3.4400000 0.0053939 1.3962949	C 0.646963 1.183977 -0.903757<br>C -1.412585 -0.214389 -0.818391<br>C -0.759271 1.175926 1.166368<br>C -0.242647 0.336185 0.005020<br>N 0.444054 -0.834315 0.560655<br>H 1.455243 1.635777 -0.323812<br>H 0.080943 1.994467 -1.365963<br>H 1.074103 0.574217 -1.703811<br>H -1.005918 -0.755672 -1.684095<br>H -2.021663 0.608888 -1.194540<br>H -1.439814 0.593802 1.784345<br>H -1.291096 2.051511 0.794287<br>H 0.071287 1.520617 1.785425<br>H 1.095716 -0.591834 1.297104<br>H 0.950345 -1.357173 -0.146217<br>O 3.143401 -0.682538 0.074232<br>H 3.040563 0.109283 -0.480346<br>O -2.249719 -1.048049 -0.057701<br>H -1.662309 -1.589182 0.485349
SP-H16<br><br>$\tilde{\upsilon}$: -665.7, 80.8, 119.6, 145.3, 163.6, 227.0, 236.4, 270.9, 310.8, 357.5, 362.8, 421.5, 443.2, 459.7, 559.8, 714.1, 758.1,	C -0.436163 1.666158 -0.378651<br>C 0.161101 0.323094 0.035277<br>C 0.028027 0.109603 1.536340<br>C 1.637765 0.285028 -0.393267

796.7, 911.6, 943.6, 959.4, 999.1, 1039.9, 1091.0, 1126.5, 1189.2, 1231.0, 1276.2, 1329.6, 1374.4, 1397.1, 1413.4, 1427.9, 1482.7, 1491.2, 1503.2, 1508.5, 1514.7, 1570.5, 2199.3, 3003.9, 3051.9, 3072.1, 3107.5, 3122.5, 3143.2, 3146.8, 3157.3, 3523.0, 3814.5, 3836.6<br><br>B: 3.5600000 0.0013939 1.5525035	N -0.473945 -0.801299 -0.652587<br>O 2.283094 -0.914580 -0.045270<br>H -0.360086 1.810707 -1.458584<br>H 0.088850 2.486842 0.111625<br>H -1.486635 1.701709 -0.092217<br>H 0.477138 -0.836976 1.830588<br>H -1.024405 0.109478 1.818102<br>H 0.535152 0.911731 2.072111<br>H 2.172732 1.093370 0.106103<br>H 1.693522 0.460877 -1.475784<br>H -0.599065 -0.604625 -1.642477<br>H -1.413883 -1.108935 -0.227990<br>H 1.705653 -1.639180 -0.308447<br>O -2.748043 -0.672478 0.028266<br>H -3.096150 -0.382746 -0.827083
$(CH_3)_2(CH_2OH)C\dot{N}H$<br><br>$\tilde{\upsilon}$: 141.3, 214.6, 240.0, 264.1, 287.5, 336.7, 377.9, 402.7, 428.3, 477.5, 568.0, 785.0, 918.1, 931.8, 955.0, 982.7, 1021.2, 1113.3, 1135.6, 1182.3, 1195.6, 1286.7, 1343.4, 1384.0, 1389.8, 1405.1, 1438.0, 1480.5, 1486.9, 1498.3, 1511.1, 1515.1, 3025.9, 3054.2, 3059.9, 3105.9, 3126.2, 3130.4, 3134.3, 3155.8, 3430.7, 3823.8<br><br>B: 4.7912622 2.7527182 2.6614024	C -1.612958 -0.865260 -0.270175<br>C -0.389776 -0.004193 0.043587<br>C -0.521020 1.379283 -0.605891<br>C 0.881788 -0.704632 -0.445419<br>N -0.242437 0.171942 1.473533<br>O 2.043661 -0.016132 -0.051089<br>H -1.563550 -1.814633 0.263252<br>H -1.660565 -1.073182 -1.340161<br>H -2.530393 -0.350362 0.017841<br>H 0.368307 1.975388 -0.411935<br>H -1.395539 1.904471 -0.220080<br>H -0.635317 1.265015 -1.684590<br>H 0.888281 -0.749098 -1.535245<br>H 0.885224 -1.730713 -0.059721<br>H -1.126842 0.541682 1.832209<br>H 1.969963 0.125702 0.899800
PRE-H17<br><br>$\tilde{\upsilon}$: 54.0, 69.6, 148.3, 195.0, 231.3, 251.6, 280.4, 300.5, 341.8, 355.9, 383.2, 413.9, 451.6, 507.5, 564.4, 713.6, 782.3, 869.1, 922.1, 950.0, 965.1, 994.4, 1054.6, 1095.6, 1112.8, 1221.7, 1245.3, 1295.4, 1336.7, 1373.3, 1406.1, 1421.8, 1454.6, 1484.8, 1489.1, 1505.3, 1512.6, 1524.0, 1637.0, 3029.9, 3049.8, 3059.6, 3098.8, 3121.8, 3126.7, 3129.6, 3145.9, 3506.4, 3599.7, 3609.6, 3699.1<br><br>B: 3.3500000 0.0021803 1.5770681	C 1.561702 -1.202979 0.792238<br>C 0.825494 -0.133208 -0.008580<br>C 1.812543 0.879551 -0.578640<br>C -0.189618 0.565898 0.902966<br>N 0.043965 -0.678333 -1.126952<br>O -1.069044 1.393518 0.159892<br>H 0.865709 -1.964044 1.147832<br>H 2.063675 -0.767341 1.658197<br>H 2.316063 -1.689423 0.172440<br>H 1.290069 1.687855 -1.088727<br>H 2.482582 0.396930 -1.292448<br>H 2.419938 1.307402 0.218449<br>H 0.329475 1.183395 1.637494<br>H -0.766202 -0.196109 1.439760<br>H -0.548273 -1.443892 -0.819883<br>H 0.647158 -1.025342 -1.862042<br>H -0.999516 1.076889 -0.755149<br>O -2.910999 -0.724998 -0.083238<br>H -2.628817 0.178270 0.171607
SP-H17	C 1.944007 -0.622525 0.916383<br>C 0.858583 -0.083562 -0.018044<br>C 1.448131 0.940226 -0.981169

$\tilde{\nu}$: -1568.1, 73.7, 100.2, 199.7, 223.6, 231.0, 260.2, 277.7, 288.6, 344.1, 368.9, 412.6, 423.3, 447.5, 561.1, 774.7, 794.1, 831.1, 910.3, 936.0, 951.3, 994.5, 1038.6, 1099.9, 1111.6, 1208.5, 1216.6, 1294.1, 1324.2, 1361.6, 1396.4, 1403.1, 1419.5, 1462.2, 1484.3, 1488.3, 1503.2, 1512.6, 1648.8, 1662.2, 3018.0, 3046.3, 3058.2, 3071.3, 3117.4, 3122.5, 3126.1, 3151.1, 3513.6, 3602.0, 3823.4<br><br>B: 3.7400000 0.0021699 1.5658083	C -0.223534 0.570889 0.842876<br>N 0.210178 -1.125022 -0.813612<br>O -1.299200 1.083821 0.104640<br>H 1.533927 -1.378948 1.587969<br>H 2.383595 0.172714 1.521786<br>H 2.742837 -1.083014 0.333499<br>H 0.661002 1.370157 -1.597783<br>H 2.179988 0.463382 -1.635654<br>H 1.952050 1.739333 -0.436416<br>H 0.197754 1.413765 1.398807<br>H -0.594613 -0.150219 1.581138<br>H -0.230357 -1.822979 -0.224224<br>H 0.883811 -1.596679 -1.404492<br>H -1.841860 0.262187 -0.363403<br>O -2.710553 -0.612570 -0.098486<br>H -3.424483 -0.114715 0.324553
POST-H17<br><br>$\tilde{\nu}$: 43.0, 112.2, 135.6, 163.2, 216.6, 232.5, 252.3, 260.6, 279.2, 335.2, 351.8, 400.5, 413.6, 452.4, 569.4, 589.2, 675.9, 782.6, 902.3, 927.3, 950.9, 982.0, 1018.9, 1086.8, 1107.5, 1113.6, 1227.2, 1297.3, 1356.8, 1365.2, 1394.7, 1404.2, 1427.1, 1485.9, 1488.5, 1503.5, 1514.0, 1644.2, 1649.5, 2959.8, 2990.1, 3047.5, 3059.9, 3117.3, 3125.5, 3130.2, 3153.0, 3499.1, 3586.1, 3688.9, 3920.3<br><br>B: 3.2900000 0.0029548 1.5781918	C -1.965026 -0.583356 -0.904732<br>C -0.831088 -0.122614 0.010539<br>C -1.384214 0.562628 1.251736<br>C 0.057815 0.853825 -0.768167<br>N 0.023907 -1.236993 0.444542<br>O 1.025840 1.480643 -0.033139<br>H -1.567859 -1.052850 -1.807406<br>H -2.597800 0.252742 -1.206780<br>H -2.590050 -1.313525 -0.389617<br>H -0.569558 0.888813 1.895469<br>H -2.023251 -0.123649 1.810148<br>H -1.983137 1.430944 0.975874<br>H -0.551972 1.646846 -1.231842<br>H 0.554240 0.343058 -1.609121<br>H 0.234439 -1.848941 -0.338241<br>H -0.452318 -1.799261 1.140230<br>H 1.997958 -0.775455 0.425566<br>O 2.850813 -0.667593 -0.023876<br>H 3.103820 0.242919 0.143790
$(CH_3)_2(NH_2)CCH_2\dot{O}$<br><br>$\tilde{\nu}$: 142.9, 224.0, 238.3, 277.2, 310.2, 337.2, 363.2, 414.6, 447.0, 566.8, 724.5, 778.9, 879.9, 911.2, 956.2, 1004.8, 1016.2, 1068.7, 1082.4, 1136.8, 1230.0, 1250.4, 1343.4, 1359.9, 1381.5, 1405.9, 1417.0, 1484.9, 1492.4, 1500.8, 1510.3, 1654.1, 2925.2, 2993.9, 3052.3, 3058.8, 3120.4, 3127.7, 3143.7, 3147.9, 3507.9, 3590.4<br><br>B: 4.5934692 2.7587965 2.7233931	C -1.561315 -0.760062 -0.516583<br>C -0.371762 0.013929 0.031556<br>C -0.454540 1.485022 -0.363210<br>C 0.922038 -0.578964 -0.557903<br>N -0.386842 -0.066061 1.488515<br>O 2.066953 -0.038668 -0.045779<br>H -1.516807 -1.808698 -0.213585<br>H -1.583952 -0.721914 -1.606278<br>H -2.487220 -0.333739 -0.133361<br>H 0.403228 2.030808 0.033160<br>H -1.363035 1.925874 0.043661<br>H -0.459281 1.596913 -1.448229<br>H 0.935115 -0.544719 -1.657738<br>H 0.981499 -1.651354 -0.290994<br>H 0.458818 0.351694 1.861862<br>H -0.402627 -1.032641 1.794963
H_2O<br>$\tilde{\nu}$: 1619.7, 3868.5, 3971.5<br>B: 833.6235141 431.1572925 284.1779820	O 0.000000 0.000000 0.116391<br>H 0.000000 0.762576 -0.465563<br>H 0.000000 -0.762576 -0.465563

Table S3 Quantum chemistry results for the $(CH_3)_2C(\dot{N}H)CH_2OH$ dissociation. Electronic energies of reactants, intermediates and products (/Hartree), and relative energies including Zero Point Energies, $\Delta E_{v=0}$ (/kJ mol^{-1}), of stationary points on the potential energy surface.

	M06-2X/aug-cc-pVTZ			CCSD(T*)/ aug-cc-pVTZ	
Species	E_{Elec}	E_{ZPE}	$\Delta E_{v=0}$	E_{Elec}	$\Delta E_{v=0}$
$(CH_3)_2C(\dot{N}H)CH_2OH$	-288.337782	0.140666	0	-288.011828	0.0
SP CH_3 ejection	-288.290414	0.136845	114.3		
SP CH_2OH ejection	-288.304151	0.138064	81.5	-287.977359	83.7

Table S3, continued: Vibrational frequencies (/cm^{-1}), Rotational constants (B /GHZ) and Cartesian Coordinates (/Å) of the species listed above. Results from M06-2X/aug-cc-pVTZ calculations.

$(CH_3)_2(CH_2OH)C\dot{N}H$<br><br>$T_1$ = 0.0125, D_1 = 0.0454<br>$\tilde{\upsilon}$: 141.3, 214.6, 240.0, 264.1, 287.5, 336.7, 377.9, 402.7, 428.3, 477.5, 568.0, 785.0, 918.1, 931.8, 955.0, 982.7, 1021.2, 1113.3, 1135.6, 1182.3, 1195.6, 1286.7, 1343.4, 1384.0, 1389.8, 1405.1, 1438.0, 1480.5, 1486.9, 1498.3, 1511.1, 1515.1, 3025.9, 3054.2, 3059.9, 3105.9, 3126.2, 3130.4, 3134.3, 3155.8, 3430.7, 3823.8<br><br>B: 4.7912622 2.7527182 2.6614024	C -1.612958 -0.865260 -0.270175<br>C -0.389776 -0.004193 0.043587<br>C -0.521020 1.379283 -0.605891<br>C 0.881788 -0.704632 -0.445419<br>N -0.242437 0.171942 1.473533<br>O 2.043661 -0.016132 -0.051089<br>H -1.563550 -1.814633 0.263252<br>H -1.660565 -1.073182 -1.340161<br>H -2.530393 -0.350362 0.017841<br>H 0.368307 1.975388 -0.411935<br>H -1.395539 1.904471 -0.220080<br>H -0.635317 1.265015 -1.684590<br>H 0.888281 -0.749098 -1.535245<br>H 0.885224 -1.730713 -0.059721<br>H -1.126842 0.541682 1.832209<br>H 1.969963 0.125702 0.899800
SP CH_3 ejection<br><br>$\tilde{\upsilon}$: -590.2, 90.4, 148.7, 181.1, 190.9, 230.2, 284.4, 368.3, 436.4, 466.2, 533.5, 579.6, 592.5, 784.5, 834.3, 920.3, 943.8, 1039.4, 1051.1, 1099.7, 1123.2, 1210.9, 1359.5, 1375.3, 1404.0, 1421.5, 1430.1, 1433.8, 1480.5, 1482.4, 1505.3, 1544.6, 3062.6, 3066.1, 3111.0, 3131.7, 3133.0, 3151.0, 3269.2, 3282.7, 3466.0, 3850.4<br><br>B: 4.3400000 0.0083114 2.4726646	C -1.895682 -1.031060 -0.217069<br>C -0.178578 0.248089 0.187527<br>C -0.513604 1.363278 -0.778542<br>C 0.853780 -0.760437 -0.292123<br>N -0.260497 0.369352 1.478721<br>O 2.146896 -0.198715 -0.171049<br>H -1.774193 -1.791054 0.539187<br>H -1.689211 -1.314018 -1.240090<br>H -2.692706 -0.318673 -0.062802<br>H 0.381530 1.972143 -0.918516<br>H -1.308041 1.995523 -0.384515<br>H -0.811655 0.982832 -1.753845<br>H 0.720719 -0.997597 -1.346346<br>H 0.762613 -1.673949 0.299528<br>H -0.803054 1.195701 1.730752<br>H 2.266808 0.034128 0.755231

SP CH_2OH ejection<br><br>T_1 = 0.0202, D_1 = 0.0922<br>$\tilde{\upsilon}$: -534.8, 125.6, 177.8, 190.7, 225.6, 255.2, 271.0, 394.6, 461.1, 502.0, 563.3, 639.5, 763.7, 812.4, 944.5, 978.8, 1008.9, 1072.6, 1080.3, 1130.7, 1133.8, 1232.1, 1362.8, 1377.8, 1406.8, 1413.5, 1464.5, 1476.8, 1485.5, 1493.8, 1509.7, 1541.2, 3052.8, 3062.8, 3088.4, 3118.8, 3129.1, 3139.9, 3168.2, 3211.2, 3462.8, 3672.8<br><br>B: 4.5800000 0.0034420 2.5360990	C -1.649633 -0.837366 -0.441397<br>C -0.653514 0.095984 0.209567<br>C -0.573200 1.481337 -0.392243<br>C 1.127374 -0.654607 -0.643029<br>N -0.291352 -0.022888 1.464225<br>O 2.084646 0.041925 0.002364<br>H -1.574275 -1.842129 -0.026396<br>H -1.506074 -0.888190 -1.520790<br>H -2.661308 -0.467120 -0.260208<br>H 0.306867 2.001516 -0.022550<br>H -1.458016 2.041853 -0.083166<br>H -0.552955 1.453443 -1.480561<br>H 1.090468 -0.441178 -1.705223<br>H 0.971075 -1.688367 -0.344563<br>H -0.595707 -0.923177 1.835264<br>H 1.836056 0.066080 0.942322

Table S4 Quantum chemistry results for the $(CH_3)_2C(\dot{N}H)CH_2OH$ + NO reaction. Electronic energies of reactants, intermediates and products (/Hartree), and relative energies including Zero Point Energies, $\Delta E_{v=0}$ (/kJ mol^{-1}), of stationary points on the potential energy surface.

	M06-2X/aug-cc-pVTZ		
Species	E_{Elec}	E_{ZPE}	$\Delta E_{v=0}$
$(CH_3)_2C(\dot{N}H)CH_2OH$	-288.337782	0.140666	
NO	-129.893351	0.004707	
Sum Reactants	-418.231134	0.145373	0
$ONNHC(CH_3)_2CH_2OH$ (*anti*)	-418.311135	0.153403	-189.0
SP *anti-syn*	-418.268629	0.150943	-83.8
$ONNHC(CH_3)_2CH_2OH$ (*syn*)	-418.310813	0.152400	-190.7
SP-8	-418.257935	0.147712	-64.2
$HONNC(CH_3)_2CH_2OH$ (*sas*)	-418.313896	0.152522	-198.5
SP *sas-saa*	-418.299637	0.150879	-165.4
$HONNC(CH_3)_2CH_2OH$ (*saa*)	-418.311213	0.152402	-191.8
SP *saa-ssa*	-418.261427	0.148888	-70.3
$HONNC(CH_3)_2CH_2OH$ (*ssa*)	-418.303718	0.152351	-172.3
SP-9c	-418.280462	0.144783	-131.1
$(CH_3)_2C{=}N{=}N$	-227.352571	0.089893	
CH_2O	-114.498970	0.027013	
H_2O	-76.430092	0.021558	
Sum products	-418.281634	0.138464	-150.7

Table S4, continued: Vibrational frequencies (/cm^{-1}), Rotational constants (B /GHZ) and Cartesian Coordinates (/Å) of the species listed above. Results from M06-2X/aug-cc-pVTZ calculations.

$(CH_3)_2(CH_2OH)C\dot{N}H$<br><br>$\tilde{\upsilon}$: 141.3, 214.6, 240.0, 264.1, 287.5, 336.7, 377.9, 402.7, 428.3, 477.5, 568.0, 785.0, 918.1, 931.8, 955.0, 982.7, 1021.2, 1113.3, 1135.6, 1182.3, 1195.6, 1286.7, 1343.4, 1384.0, 1389.8, 1405.1, 1438.0, 1480.5, 1486.9, 1498.3, 1511.1, 1515.1, 3025.9, 3054.2, 3059.9, 3105.9, 3126.2, 3130.4, 3134.3, 3155.8, 3430.7, 3823.8<br><br>B: 4.7912622 2.7527182 2.6614024	C -1.612958 -0.865260 -0.270175<br>C -0.389776 -0.004193 0.043587<br>C -0.521020 1.379283 -0.605891<br>C 0.881788 -0.704632 -0.445419<br>N -0.242437 0.171942 1.473533<br>O 2.043661 -0.016132 -0.051089<br>H -1.563550 -1.814633 0.263252<br>H -1.660565 -1.073182 -1.340161<br>H -2.530393 -0.350362 0.017841<br>H 0.368307 1.975388 -0.411935<br>H -1.395539 1.904471 -0.220080<br>H -0.635317 1.265015 -1.684590<br>H 0.888281 -0.749098 -1.535245<br>H 0.885224 -1.730713 -0.059721<br>H -1.126842 0.541682 1.832209<br>H 1.969963 0.125702 0.899800
NO<br>$\tilde{\upsilon}$: 2066.0<br>B: 0.0000000 52.3561936 52.3561936	N 0.000000 0.000000 -0.606410<br>O 0.000000 0.000000 0.530609
$ONNHC(CH_3)_2CH_2OH$ (*anti*)<br><br>$\tilde{\upsilon}$: 101.5, 153.9, 233.1, 244.0, 247.8, 281.0, 302.4, 332.6, 368.5, 414.5, 488.6, 527.2, 584.9, 643.7, 726.1, 863.8, 903.7, 947.0, 962.4, 1022.7, 1045.1, 1153.9, 1189.7, 1236.4, 1258.6, 1264.7, 1290.9, 1386.4, 1405.6, 1423.5, 1442.0, 1462.6, 1486.8, 1488.7, 1499.1, 1511.4, 1515.2, 1635.7, 3027.2, 3056.6, 3071.3, 3101.4, 3124.9, 3142.4, 3151.8, 3171.3, 3627.9, 3815.7<br><br>B: 2.4600000 0.0068488 2.0102390	C -0.703052 -0.159139 0.034232<br>C -2.051348 -0.681767 -0.452396<br>C -0.371545 1.128945 -0.757720<br>N 0.251408 -1.250885 -0.265763<br>C -0.731604 0.102843 1.536463<br>N 1.552731 -1.239114 -0.184465<br>O 2.061762 -0.170431 0.100869<br>O 0.442023 2.053360 -0.094996<br>H -2.281110 -1.650643 -0.005913<br>H -2.060753 -0.783764 -1.539016<br>H -2.836834 0.013988 -0.164056<br>H -1.316406 1.642847 -0.939129<br>H 0.042056 0.838831 -1.730416<br>H -0.104339 -2.168579 -0.485777<br>H -1.386464 0.948843 1.742986<br>H 0.258720 0.348257 1.910425<br>H -1.106232 -0.775608 2.061903<br>H 1.277389 1.607099 0.090122
SP *anti-syn*<br>$\tilde{\upsilon}$: -421.4, 84.0, 129.0, 222.5, 255.1, 257.1, 324.0, 329.8, 356.5, 400.2, 418.3, 439.0, 531.7, 637.6, 758.4, 804.8, 907.4, 925.8, 962.8, 971.0, 989.6, 1038.3, 1116.7, 1173.4, 1214.3, 1257.5, 1286.6, 1362.5, 1392.3, 1401.8, 1409.5, 1432.5, 1484.7, 1492.0, 1502.2, 1505.1, 1519.8, 1765.8, 3050.1, 3057.8, 3065.5, 3110.1, 3128.6, 3134.8, 3142.0, 3163.7, 3485.3, 3858.9<br><br>B: 2.5300000 0.0046284 1.8605424	C -0.688825 -0.255040 0.012103<br>C -1.902624 -1.022631 -0.501495<br>C -0.984647 1.245359 -0.059601<br>N 0.380585 -0.489669 -0.992230<br>C -0.399139 -0.662553 1.458954<br>N 1.775562 -0.176181 -0.626198<br>O 2.133192 -0.623386 0.397628<br>O 0.171015 2.025161 0.163078<br>H -1.753418 -2.097581 -0.387777<br>H -2.084920 -0.800383 -1.552591<br>H -2.787284 -0.746210 0.072584<br>H -1.698570 1.511200 0.721380<br>H -1.428119 1.475358 -1.032189

	H 0.413024 -1.470750 -1.265205<br>H -1.326308 -0.580431 2.028784<br>H 0.340240 -0.013040 1.920641<br>H -0.047046 -1.691102 1.520017<br>H 0.697127 2.028876 -0.642062
ONNHC(CH_3)$_2$$CH_2$OH (*syn*)<br><br>ṽ: 33.9, 142.1, 184.2, 206.7, 237.1, 266.4, 278.9, 344.0, 365.1, 397.4, 472.5, 509.3, 582.6, 612.9, 687.6, 840.1, 913.1, 954.6, 989.4, 1004.9, 1037.0, 1138.5, 1186.5, 1223.1, 1251.9, 1282.2, 1320.1, 1396.1, 1406.4, 1419.2, 1433.3, 1478.9, 1485.0, 1485.8, 1499.6, 1512.9, 1517.3, 1620.9, 3049.0, 3061.3, 3065.4, 3111.9, 3131.6, 3135.5, 3146.7, 3162.2, 3478.6, 3836.3<br><br>B: 2.5900000 0.0092776 1.6325047	C -0.727122 -0.344568 0.007591<br>C -1.437877 -1.404064 -0.830066<br>C -0.833632 1.005189 -0.710236<br>N 0.684399 -0.736182 0.111627<br>C -1.325991 -0.264927 1.408826<br>N 1.670983 0.088498 -0.120115<br>O 2.777856 -0.368192 0.061118<br>O -0.349408 2.096968 0.027943<br>H -1.319465 -2.391372 -0.381769<br>H -1.033006 -1.434013 -1.841509<br>H -2.503881 -1.185632 -0.882215<br>H -1.894168 1.190693 -0.883502<br>H -0.340626 0.928274 -1.684115<br>H 0.948995 -1.652171 0.470688<br>H -2.386226 -0.019408 1.347129<br>H -0.825104 0.505429 1.990496<br>H -1.225772 -1.222971 1.920944<br>H 0.611741 2.034972 0.034090
SP-8<br><br>ṽ: -1890.0, 84.8, 144.5, 171.9, 201.4, 231.8, 260.9, 281.1, 346.0, 360.1, 408.9, 456.5, 500.7, 597.2, 647.8, 860.7, 916.3, 960.5, 1002.2, 1035.5, 1123.4, 1146.5, 1189.9, 1199.3, 1222.7, 1268.9, 1309.3, 1312.4, 1395.6, 1404.7, 1413.7, 1433.0, 1483.2, 1487.6, 1499.8, 1503.2, 1515.0, 1564.9, 2149.4, 3048.8, 3065.6, 3070.1, 3110.8, 3140.7, 3142.8, 3146.9, 3162.5, 3858.5<br><br>B: 2.5700000 0.0062945 1.6545544	C -0.679034 -0.399284 0.006153<br>C -1.185184 -1.554912 -0.851446<br>C -0.999373 0.927121 -0.690262<br>N 0.776704 -0.564086 0.140907<br>C -1.281765 -0.448933 1.405936<br>N 1.613250 0.306519 -0.188294<br>O 2.750030 -0.230695 0.085329<br>O -0.646398 2.066676 0.052579<br>H -0.915503 -2.508750 -0.399629<br>H -0.756673 -1.512641 -1.852716<br>H -2.270870 -1.507348 -0.931336<br>H -2.080464 0.966246 -0.830056<br>H -0.531166 0.933234 -1.679283<br>H 1.866370 -1.182745 0.451792<br>H -2.368813 -0.409047 1.337275<br>H -0.939081 0.397724 1.996432<br>H -0.999459 -1.374656 1.906676<br>H 0.309068 2.169146 0.007004
HONNC(CH_3)$_2$$CH_2$OH (*sas*)<br>ṽ: 86.2, 159.9, 200.6, 214.6, 237.6, 259.6, 283.9, 339.6, 363.6, 405.7, 462.4, 513.5, 583.3, 591.5, 616.5, 836.0, 910.7, 962.5, 970.6, 995.6, 1031.9, 1079.4, 1131.0, 1181.2, 1225.8, 1287.1, 1300.9, 1392.2, 1401.9, 1410.7, 1428.1, 1434.7, 1481.6, 1488.0, 1500.5, 1502.2, 1515.7, 1733.8, 3047.6, 3064.8, 3070.8, 3109.0, 3138.4, 3144.4, 3150.5, 3158.8, 3680.8, 3863.6<br><br>B: 2.5800000 0.0037462 1.6202958	C -0.704269 -0.385856 0.007550<br>C -1.256422 -1.524291 -0.844284<br>C -0.956280 0.944064 -0.706336<br>N 0.734741 -0.693871 0.201662<br>C -1.328357 -0.394065 1.398250<br>N 1.501263 0.176314 -0.157401<br>O 2.810075 -0.159838 0.037194<br>O -0.587026 2.080377 0.038022<br>H -1.047925 -2.483392 -0.372977<br>H -0.805075 -1.521223 -1.836945<br>H -2.335409 -1.415098 -0.951278<br>H -2.029771 1.023603 -0.882431

	H -0.458460 0.928515 -1.680712<br>H 2.801252 -1.050898 0.430018<br>H -2.408715 -0.277181 1.312979<br>H -0.939210 0.428056 1.995230<br>H -1.114739 -1.336492 1.900825<br>H 0.373609 2.123590 0.042657
SP *sas-saa*<br><br>ṽ: -488.8, 89.8, 157.0, 197.0, 214.3, 236.2, 256.3, 283.5, 337.4, 360.6, 406.9, 468.9, 514.9, 571.0, 594.7, 827.3, 908.8, 937.8, 964.9, 990.2, 1025.7, 1048.3, 1131.7, 1179.0, 1219.0, 1278.9, 1286.3, 1306.4, 1391.5, 1401.2, 1411.7, 1434.5, 1480.8, 1487.8, 1500.1, 1501.5, 1515.3, 1738.7, 3044.0, 3064.9, 3072.1, 3107.2, 3137.7, 3146.8, 3153.0, 3161.3, 3829.0, 3856.5<br><br>B: 2.5800000 0.0000383 1.5984869	C -0.711813 -0.386663 0.009644<br>C -1.247630 -1.545392 -0.824646<br>C -0.964394 0.929740 -0.728015<br>N 0.727547 -0.695092 0.228236<br>C -1.340294 -0.376588 1.398625<br>N 1.483935 0.173308 -0.133074<br>O 2.828216 -0.179823 0.160085<br>O -0.606884 2.079780 -0.000672<br>H -1.034924 -2.493785 -0.334470<br>H -0.791446 -1.556628 -1.815243<br>H -2.326424 -1.444587 -0.940431<br>H -2.036905 1.000939 -0.913978<br>H -0.459792 0.900131 -1.698913<br>H 3.131425 -0.672772 -0.612211<br>H -2.420169 -0.262412 1.304337<br>H -0.955803 0.455162 1.984978<br>H -1.126159 -1.310660 1.915650<br>H 0.353956 2.110858 0.035193
$HONNC(CH_3)_2CH_2OH$ (*saa*)<br><br>ṽ: 61.5, 123.5, 193.0, 207.5, 244.5, 276.7, 279.3, 328.5, 355.3, 381.5, 421.5, 515.8, 571.1, 604.7, 613.7, 832.4, 920.8, 957.8, 962.4, 1006.1, 1037.3, 1067.0, 1127.6, 1183.6, 1247.2, 1277.1, 1286.4, 1380.2, 1402.8, 1414.8, 1418.0, 1438.8, 1482.7, 1487.1, 1503.1, 1508.2, 1514.9, 1734.4, 3029.3, 3067.9, 3071.4, 3113.8, 3140.1, 3143.0, 3157.3, 3160.5, 3684.2, 3855.4<br><br>B: 2.8200000 0.0013506 1.4390873	C 0.508353 0.449250 0.019371<br>C 0.305004 1.750760 -0.739366<br>C 1.517224 -0.432019 -0.726098<br>N -0.715440 -0.384184 0.114133<br>C 0.969427 0.704570 1.451189<br>N -1.755471 0.203110 -0.104550<br>O -2.848720 -0.603707 0.011163<br>O 1.775598 -1.652597 -0.075162<br>H -0.395562 2.402542 -0.222221<br>H -0.082480 1.565391 -1.740971<br>H 1.261994 2.265635 -0.823054<br>H 2.466975 0.099564 -0.788521<br>H 1.151971 -0.597990 -1.746028<br>H 1.914805 1.247018 1.436275<br>H 1.117555 -0.232109 1.983888<br>H 0.229186 1.304281 1.980183<br>H -2.514124 -1.483301 0.260394<br>H 0.930981 -2.088441 0.074381
SP *saa-ssa*<br><br>ṽ: -331.1, 63.1, 122.4, 147.6, 221.6, 240.4, 262.0, 304.0, 328.8, 354.5, 374.2, 383.9, 506.4, 519.6, 530.8, 557.2, 728.1, 868.8, 911.7, 961.2, 997.7, 1046.4, 1132.8, 1142.0, 1184.9, 1220.4, 1251.6, 1280.7, 1389.8, 1401.0, 1414.1, 1433.7, 1481.4, 1489.8, 1499.0, 1507.8, 1515.0, 2055.5, 3032.7, 3069.4, 3077.6, 3109.9, 3145.4, 3155.0, 3157.4, 3170.9, 3755.5, 3850.8<br><br>B: 2.5400000 0.0095745 1.5913110	C 0.839665 0.260768 -0.006496<br>C 1.640771 1.265189 -0.825507<br>C 0.896154 -1.142688 -0.642062<br>N -0.552754 0.628821 0.019655<br>C 1.298095 0.214616 1.449420<br>N -1.602761 1.107396 0.046157<br>O -2.678689 -0.065608 -0.050916<br>O 0.061237 -2.053779 0.011888<br>H 1.618413 2.245864 -0.354284<br>H 1.239303 1.348447 -1.834402<br>H 2.675657 0.928177 -0.890076

	H 1.916834 -1.519581 -0.563107<br>H 0.642869 -1.051214 -1.703990<br>H 2.352045 -0.060725 1.464166<br>H 0.731078 -0.531230 2.000962<br>H 1.179349 1.189111 1.918992<br>H -3.527330 0.389201 -0.007569<br>H -0.848102 -1.723791 -0.031277
HONNC(CH3)2CH2OH (*ssa*)<br><br>ṽ: 101.2, 162.3, 228.0, 243.4, 265.4, 301.8, 310.4, 325.3, 374.6, 404.9, 443.5, 499.2, 552.8, 634.3, 681.6, 704.2, 824.7, 876.0, 909.5, 963.5, 1020.4, 1036.5, 1146.5, 1177.6, 1225.9, 1259.2, 1281.9, 1313.5, 1386.8, 1404.0, 1421.9, 1451.8, 1482.0, 1489.5, 1498.5, 1502.4, 1515.8, 1809.7, 3037.6, 3064.2, 3076.5, 3113.6, 3137.2, 3146.2, 3155.5, 3178.3, 3866.2, 3868.8<br><br>B: 2.4200000 0.0056189 2.0338391	C 0.719279 -0.175470 0.020971<br>C 0.704211 0.027016 1.532612<br>C 0.335487 1.112617 -0.752209<br>N -0.142959 -1.353531 -0.302612<br>C 2.106961 -0.610353 -0.443110<br>N -1.339843 -1.392896 -0.273679<br>O -1.985495 -0.148106 0.091728<br>O -0.419374 2.060429 -0.048288<br>H 1.059836 -0.874885 2.029578<br>H -0.290193 0.269815 1.895356<br>H 1.368190 0.853994 1.781419<br>H -0.144559 0.826610 -1.694353<br>H 1.269877 1.615202 -1.000403<br>H 2.848093 0.127821 -0.139477<br>H 2.134017 -0.706215 -1.529538<br>H 2.364673 -1.574972 -0.010018<br>H -2.916139 -0.390988 0.056624<br>H -1.270862 1.657161 0.147740
SP-9c<br><br>ṽ: -354.0, 71.1, 137.2, 192.8, 213.3, 232.8, 257.5, 279.1, 289.2, 351.4, 371.1, 421.7, 458.0, 540.6, 547.5, 592.3, 665.1, 753.9, 886.8, 970.6, 1023.1, 1043.9, 1142.1, 1181.3, 1226.4, 1231.4, 1242.9, 1262.6, 1347.4, 1406.5, 1424.7, 1465.2, 1480.3, 1485.9, 1496.2, 1507.0, 1556.8, 1745.0, 2376.7, 2953.6, 3016.8, 3073.0, 3074.2, 3150.3, 3153.7, 3161.3, 3175.4, 3916.7<br><br>B: 2.4900000 0.0049107 1.8155257	C -0.864518 0.004717 0.059814<br>C -0.720102 -0.195642 1.557997<br>C -0.209225 -1.163807 -0.758663<br>N -0.052900 1.239645 -0.243115<br>C -2.278144 0.294154 -0.407502<br>N 0.863595 1.831724 -0.373854<br>O 2.243845 0.230200 0.181737<br>O 0.879554 -1.726385 -0.185737<br>H -1.220768 0.595673 2.113985<br>H 0.333232 -0.255308 1.821942<br>H -1.187766 -1.152308 1.788593<br>H -0.044759 -0.778068 -1.779148<br>H -1.031015 -1.890501 -0.817925<br>H -2.904567 -0.540376 -0.098747<br>H -2.321711 0.376487 -1.493266<br>H -2.671798 1.205153 0.039848<br>H 3.197753 0.192587 0.098492<br>H 1.621282 -0.919973 -0.032864
(CH3)2C=N=N<br><br>ṽ: 125.3, 145.5, 220.1, 233.8, 319.4, 570.1, 648.5, 765.0, 995.0, 1003.3, 1037.0, 1091.2, 1288.3, 1411.8, 1422.9, 1441.1, 1473.1, 1484.5, 1499.0, 1539.9, 2187.9, 3039.8, 3041.1, 3083.8, 3088.5, 3151.1, 3151.4<br><br>B: 8.2300000 0.0017817 4.3253356	C -0.000000 1.308892 -1.035818<br>C 0.000000 -0.000000 -0.306524<br>N -0.000000 0.000000 0.979708<br>C -0.000000 -1.308892 -1.035818<br>N -0.000000 0.000000 2.114368<br>H -0.881052 1.396002 -1.676130<br>H 0.881052 1.396002 -1.676130<br>H -0.000000 2.146993 -0.342523<br>H -0.000000 -2.146993 -0.342523

	H 0.881052 -1.396002 -1.676130<br>H -0.881052 -1.396002 -1.676130
CH_2O<br><br>$\tilde{\upsilon}$: 1213.6, 1273.5, 1539.9, 1869.1, 2945.5, 3015.7<br>B: 284.7518606 39.4556659 34.6539588	C 0.000000 0.000000 -0.525505<br>H 0.000000 0.938356 -1.105549<br>H 0.000000 -0.938356 -1.105549<br>O 0.000000 0.000000 0.670516
H_2O<br>$\tilde{\upsilon}$: 1619.7, 3868.5, 3971.5<br>B: 833.6235141 431.1572925 284.1779820	O 0.000000 0.000000 0.116391<br>H 0.000000 0.762576 -0.465563<br>H 0.000000 -0.762576 -0.465563

Table S5 Quantum chemistry results for reactions relevant to the $ONNHC(CH_3)_2CH_2OH$ + OH reaction. Electronic energies of reactants, intermediates and products (/Hartree), and relative energies including Zero Point Energies, $\Delta E_{v=0}$ (/kJ mol^{-1}), of stationary points on the potential energy surface.

	M06-2X/aug-cc-pVTZ		
Species	E_{Elec}	E_{ZPE}	$\Delta E_{v=0}$
$ONNHC(CH_3)_2CH_2OH$ (*syn*)	-418.310813	0.152400	
OH	-75.733810	0.008588	
Sum Reactants	-494.044623	0.160988	0
PRE-10 *syn*	-494.057642	0.163142	-28.5
SP-10 *syn*	-494.052364	0.160625	-21.3
POST-10 *syn*	-494.108900	0.163834	-161.3
$ON\dot{N}$-$C(CH_3)_2CH_2OH$	-417.672099	0.139873	
H_2O	-76.430107	0.021551	
Sum products	-494.102206	0.161424	-150.0
$ONNHC(CH_3)_2CH_2OH$ (*anti*)	-418.311135	0.153403	
OH	-75.733810	0.008588	
Sum Reactants	-494.044945	0.161991	1.8
PRE-10 *anti*	-494.055209	0.163712	-20.6
SP-10 *anti*	-494.03705	0.158804	14.1
POST-10 *anti*	-494.103809	0.164165	-147.0

Table S5, continued: Vibrational frequencies (/cm^{-1}), Rotational constants (B /GHZ) and Cartesian Coordinates (/Å) of the species listed above. Results from M06-2X/aug-cc-pVTZ calculations.

$ONNHC(CH_3)_2CH_2OH$ (*syn*)<br><br>$\tilde{\upsilon}$: 33.9, 142.1, 184.2, 206.7, 237.1, 266.4, 278.9, 344.0, 365.1, 397.4, 472.5, 509.3, 582.6, 612.9, 687.6, 840.1, 913.1, 954.6, 989.4, 1004.9, 1037.0, 1138.5, 1186.5, 1223.1, 1251.9, 1282.2, 1320.1, 1396.1, 1406.4, 1419.2, 1433.3, 1478.9, 1485.0, 1485.8, 1499.6, 1512.9, 1517.3, 1620.9, 3049.0, 3061.3, 3065.4, 3111.9, 3131.6, 3135.5, 3146.7, 3162.2, 3478.6, 3836.3<br><br>B: 2.5900000 0.0092776 1.6325047	C -0.727122 -0.344568 0.007591<br>C -1.437877 -1.404064 -0.830066<br>C -0.833632 1.005189 -0.710236<br>N 0.684399 -0.736182 0.111627<br>C -1.325991 -0.264927 1.408826<br>N 1.670983 0.088498 -0.120115<br>O 2.777856 -0.368192 0.061118<br>O -0.349408 2.096968 0.027943<br>H -1.319465 -2.391372 -0.381769<br>H -1.033006 -1.434013 -1.841509<br>H -2.503881 -1.185632 -0.882215<br>H -1.894168 1.190693 -0.883502<br>H -0.340626 0.928274 -1.684115<br>H 0.948995 -1.652171 0.470688<br>H -2.386226 -0.019408 1.347129<br>H -0.825104 0.505429 1.990496<br>H -1.225772 -1.222971 1.920944<br>H 0.611741 2.034972 0.034090
OH<br>$\tilde{\upsilon}$: 3769.6; B: 564.2863408	O 0.000000 0.000000 0.107992<br>H 0.000000 0.000000 -0.863937
PRE-10 (*syn*)	C 0.887253 0.565575 -0.040955

ṽ: 20.9, 68.8, 93.2, 104.9, 144.4, 165.4, 211.4, 224.6, 237.8, 266.8, 285.4, 343.2, 369.4, 388.9, 410.1, 481.9, 493.7, 585.7, 616.0, 773.0, 843.8, 915.2, 956.8, 996.1, 1007.4, 1037.6, 1137.4, 1184.5, 1222.8, 1282.9, 1286.4, 1339.8, 1396.6, 1407.0, 1416.7, 1433.8, 1483.9, 1485.7, 1490.0, 1499.6, 1514.2, 1519.4, 1582.6, 3052.7, 3063.1, 3067.0, 3114.0, 3134.8, 3138.4, 3146.7, 3162.5, 3370.3, 3792.1, 3844.0<br><br>B: 2.0800000 0.0017786 1.0503846	C 0.992061 1.729295 -1.023450<br>C 1.783524 -0.577599 -0.525210<br>N -0.515173 0.126796 -0.022634<br>C 1.272251 1.003531 1.368718<br>N -0.872367 -1.090589 -0.261360<br>O -2.070084 -1.315016 -0.210723<br>O 1.859190 -1.667644 0.357347<br>H 0.314877 2.534488 -0.737280<br>H 0.737097 1.407978 -2.033130<br>H 2.007027 2.125313 -1.023916<br>H 2.792663 -0.173875 -0.612987<br>H 1.457600 -0.890103 -1.521533<br>H -1.272616 0.774403 0.219152<br>H 2.288190 1.398087 1.368133<br>H 1.226923 0.159174 2.052665<br>H 0.600610 1.787383 1.720904<br>H 1.032532 -2.155684 0.286002<br>O -3.211256 0.903868 0.250958<br>H -3.705457 0.084916 0.414681
SP-10 (*syn*)<br><br>ṽ: -1057.9, 33.0, 106.9, 130.3, 163.7, 213.9, 231.4, 253.5, 272.8, 335.3, 336.5, 347.6, 365.0, 385.9, 440.1, 477.7, 564.9, 602.3, 638.0, 677.9, 856.9, 918.4, 959.4, 1001.3, 1036.1, 1092.2, 1138.9, 1188.9, 1201.6, 1225.3, 1287.7, 1318.6, 1393.7, 1398.7, 1406.3, 1414.2, 1435.3, 1484.0, 1487.3, 1499.5, 1503.9, 1513.5, 1516.4, 2038.3, 3053.5, 3065.8, 3070.0, 3115.1, 3140.5, 3142.9, 3148.1, 3162.9, 3851.9, 3862.3<br><br>B: 2.1100000 0.0068315 1.1345460	C 0.863932 0.538400 -0.049903<br>C 0.990407 1.682509 -1.052635<br>C 1.735236 -0.633513 -0.508334<br>N -0.553131 0.147670 -0.018176<br>C 1.246162 0.994361 1.354545<br>N -0.940829 -1.016990 -0.298520<br>O -2.152835 -1.217328 -0.249662<br>O 1.767398 -1.713110 0.391307<br>H 0.330249 2.504727 -0.777962<br>H 0.726365 1.349702 -2.056250<br>H 2.014702 2.053626 -1.061407<br>H 2.756627 -0.259974 -0.587449<br>H 1.415193 -0.951563 -1.504780<br>H -1.458747 0.757172 0.245998<br>H 2.267909 1.373085 1.349957<br>H 1.186071 0.162897 2.053130<br>H 0.583677 1.793317 1.687748<br>H 0.950630 -2.210919 0.293020<br>O -2.861938 0.892459 0.263293<br>H -3.350366 0.326473 0.873317
POST-10 (*syn*)<br><br>ṽ: 48.4, 70.4, 77.4, 100.2, 131.5, 132.1, 204.8, 234.5, 260.1, 274.8, 300.6, 311.4, 323.3, 339.0, 381.5, 399.9, 449.3, 492.1, 583.4, 591.0, 834.8, 916.7, 953.5, 965.6, 1002.7, 1043.0, 1126.1, 1166.2, 1220.9, 1272.7, 1295.4, 1390.2, 1402.3, 1407.4, 1411.6, 1435.0, 1484.2, 1491.4, 1499.9, 1508.1, 1517.1, 1622.5, 1747.3, 3043.5, 3063.1, 3072.1, 3112.8, 3137.4, 3147.5, 3153.4, 3162.5, 3778.9, 3885.2, 3938.1<br><br>B: 1.6100000 0.0011526 1.2188069	C 0.138419 -0.731137 0.154062<br>C -0.619157 -1.335575 1.332210<br>C 1.642034 -0.811633 0.421821<br>N -0.345173 0.673256 0.027937<br>C -0.231856 -1.429075 -1.149194<br>N 0.470447 1.567982 0.156393<br>O 0.409977 2.766221 0.118364<br>O 2.442862 -0.368412 -0.649788<br>H -1.693013 -1.247277 1.174092<br>H -0.357609 -0.830809 2.262936<br>H -0.365566 -2.391528 1.424031<br>H 1.885239 -1.862913 0.578815<br>H 1.877486 -0.273317 1.345362<br>H -2.420289 0.589000 -0.266936<br>H 0.026687 -2.485530 -1.076504

	H 0.316269 -0.995823 -1.983147<br>H -1.302503 -1.340679 -1.328739<br>H 2.536791 0.585860 -0.607797<br>O -3.231470 0.067866 -0.319866<br>H -3.928010 0.683478 -0.555496
ONṄ-C(CH_3)$_2$$CH_2$OH<br><br>ῡ: 50.9, 124.7, 186.4, 230.8, 253.0, 269.1, 279.7, 337.6, 360.3, 406.8, 421.7, 476.8, 576.6, 608.4, 828.6, 925.2, 940.6, 959.9, 1009.4, 1042.2, 1125.5, 1174.9, 1228.7, 1270.9, 1294.8, 1386.1, 1401.7, 1411.4, 1423.3, 1435.8, 1484.2, 1486.5, 1503.8, 1507.3, 1513.5, 1747.1, 3037.9, 3063.0, 3067.9, 3117.0, 3137.1, 3141.1, 3142.4, 3159.1, 3847.4<br><br>B: 3.0000000 0.0075208 1.4313304	C 1.681561 -0.216153 -0.572180<br>C 0.173481 1.769820 -0.691337<br>C 0.555313 0.615631 1.518472<br>C 0.431575 0.443499 0.010336<br>N -0.659662 -0.526881 -0.297197<br>N -1.781540 -0.174552 0.024039<br>O -2.868917 -0.666125 -0.060655<br>O 1.946218 -1.477078 -0.007595<br>H 2.544837 0.413935 -0.357717<br>H 1.569031 -0.286197 -1.659297<br>H -0.714748 2.251730 -0.283861<br>H 0.029296 1.621071 -1.761377<br>H 1.023351 2.436257 -0.543436<br>H -0.360310 1.042972 1.927222<br>H 1.382594 1.289238 1.742518<br>H 0.749546 -0.341581 1.997834<br>H 1.194828 -2.048554 -0.195520
H_2O<br>ῡ: 1619.7, 3868.5, 3971.5<br>B: 833.6235141 431.1572925 284.1779820	O 0.000000 0.000000 0.116391<br>H 0.000000 0.762576 -0.465563<br>H 0.000000 -0.762576 -0.465563
ONNHC(CH_3)$_2$$CH_2$OH (*anti*)<br><br>ῡ: 101.5, 153.9, 233.1, 244.0, 247.8, 281.0, 302.4, 332.6, 368.5, 414.5, 488.6, 527.2, 584.9, 643.7, 726.1, 863.8, 903.7, 947.0, 962.4, 1022.7, 1045.1, 1153.9, 1189.7, 1236.4, 1258.6, 1264.7, 1290.9, 1386.4, 1405.6, 1423.5, 1442.0, 1462.6, 1486.8, 1488.7, 1499.1, 1511.4, 1515.2, 1635.7, 3027.2, 3056.6, 3071.3, 3101.4, 3124.9, 3142.4, 3151.8, 3171.3, 3627.9, 3815.7<br><br>B: 2.4600000 0.0068488 2.0102390	C -0.703052 -0.159139 0.034232<br>C -2.051348 -0.681767 -0.452396<br>C -0.371545 1.128945 -0.757720<br>N 0.251408 -1.250885 -0.265763<br>C -0.731604 0.102843 1.536463<br>N 1.552731 -1.239114 -0.184465<br>O 2.061762 -0.170431 0.100869<br>O 0.442023 2.053360 -0.094996<br>H -2.281110 -1.650643 -0.005913<br>H -2.060753 -0.783764 -1.539016<br>H -2.836834 0.013988 -0.164056<br>H -1.316406 1.642847 -0.939129<br>H 0.042056 0.838831 -1.730416<br>H -0.104339 -2.168579 -0.485777<br>H -1.386464 0.948843 1.742986<br>H 0.258720 0.348257 1.910425<br>H -1.106232 -0.775608 2.061903<br>H 1.277389 1.607099 0.090122
PRE-10 *anti*<br><br>ῡ: -36.3, 82.6, 105.5, 152.5, 168.8, 237.8, 245.4, 249.3, 273.4, 295.4, 309.3, 333.4, 372.8, 413.8, 499.8, 506.7, 530.6, 584.0, 669.9, 731.2, 863.3, 904.7, 948.0, 960.0, 1020.6, 1042.1, 1164.3, 1187.5, 1241.3, 1266.5, 1279.2, 1305.8, 1384.8, 1409.8, 1426.3, 1447.0, 1482.3, 1492.6, 1498.0, 1502.4, 1519.8, 1524.3, 1663.1, 3035.5, 3066.3, 3080.4,	C 0.614790 0.684237 0.063428<br>C 0.525480 2.146879 -0.376999<br>C 1.576645 -0.058249 -0.903546<br>N -0.774362 0.176839 -0.046309<br>C 1.086905 0.576874 1.514278<br>N -1.190421 -1.053709 0.063557<br>O -0.332973 -1.917635 0.202163<br>O 2.267097 -1.145159 -0.354654<br>H -0.227142 2.690437 0.203531

3117.6, 3142.1, 3159.6, 3166.6, 3184.3, 3580.9, 3669.9, 3835.8<br><br>B: 2.0300000 0.0036793 1.0593154	H 0.272335 2.222834 -1.440014<br>H 1.489089 2.635239 -0.215633<br>H 2.348989 0.659382 -1.198221<br>H 1.016142 -0.335784 -1.808956<br>H -1.552812 0.824300 -0.141344<br>H 2.131331 0.893999 1.579266<br>H 1.028555 -0.451332 1.874105<br>H 0.475805 1.218722 2.155509<br>H 1.606802 -1.803834 -0.097093<br>O -3.725278 0.320219 -0.188103<br>H -3.329298 -0.573725 -0.090098
SP-10 (*anti*)<br><br>$\tilde{\upsilon}$: -1646.3, 53.2, 123.0, 129.5, 156.3, 233.0, 239.6, 252.6, 275.9, 291.1, 306.5, 320.8, 371.6, 380.2, 438.9, 515.1, 534.0, 572.6, 700.1, 807.5, 834.0, 897.8, 909.4, 963.8, 1012.4, 1039.2, 1089.0, 1157.7, 1175.0, 1230.6, 1239.1, 1278.1, 1330.3, 1380.6, 1404.5, 1422.6, 1446.7, 1483.3, 1489.8, 1496.1, 1508.7, 1518.3, 1530.9, 1656.0, 3038.6, 3067.4, 3073.0, 3109.8, 3143.4, 3145.6, 3157.7, 3170.2, 3793.0, 3812.6<br><br>B: 1.9900000 0.0052060 1.3437054	C -0.167409 -0.693958 0.022947<br>C 0.485254 -1.992431 -0.442265<br>C -1.561758 -0.592593 -0.664205<br>N 0.723958 0.379335 -0.471977<br>C -0.281288 -0.644973 1.540896<br>N 0.612379 1.668126 -0.229257<br>O -0.503186 2.068359 0.040506<br>O -2.518066 0.161454 0.016185<br>H 1.508579 -2.062936 -0.076652<br>H 0.495378 -2.050973 -1.531507<br>H -0.080795 -2.836774 -0.053160<br>H -1.952074 -1.610134 -0.701012<br>H -1.415496 -0.250188 -1.693933<br>H 1.855492 0.119255 -0.558669<br>H -0.974404 -1.418249 1.870332<br>H -0.665257 0.311830 1.884124<br>H 0.696903 -0.828049 1.985525<br>H -2.186789 1.064708 0.077201<br>O 2.949786 0.005115 0.016103<br>H 3.087039 0.893592 0.379800
POST-10 (*anti*)<br><br>$\tilde{\upsilon}$: 45.1, 59.0, 81.6, 128.4, 136.6, 165.3, 222.9, 234.7, 255.1, 278.5, 314.4, 322.3, 337.9, 373.8, 396.9, 416.6, 464.7, 518.2, 599.4, 674.4, 839.5, 858.1, 925.2, 964.8, 998.3, 1041.8, 1125.0, 1161.9, 1204.9, 1260.6, 1297.4, 1329.3, 1393.4, 1402.7, 1408.7, 1434.0, 1483.7, 1492.4, 1506.4, 1506.7, 1516.5, 1622.9, 1777.0, 3059.2, 3065.7, 3072.3, 3107.6, 3133.0, 3147.1, 3151.1, 3163.0, 3774.6, 3870.8, 3938.7<br><br>B: 1.9400000 0.0028709 1.1413231	C -0.008627 -0.630498 0.211298<br>C -0.705332 -0.746882 1.561057<br>C 1.494361 -0.860225 0.366121<br>N -0.342440 0.755095 -0.313195<br>C -0.600253 -1.586426 -0.813555<br>N 0.416867 1.676219 -0.162673<br>O 1.516548 1.909122 0.293964<br>O 2.209930 -0.657870 -0.830839<br>H -1.776749 -0.588007 1.447928<br>H -0.308814 -0.018241 2.269007<br>H -0.540383 -1.746590 1.964583<br>H 1.615750 -1.908841 0.646594<br>H 1.898023 -0.256784 1.180466<br>H -2.409220 0.826668 -0.524951<br>H -0.482400 -2.609477 -0.455589<br>H -0.081011 -1.489055 -1.765075<br>H -1.660801 -1.383426 -0.952060<br>H 2.516843 0.252578 -0.853145<br>O -3.283590 0.472022 -0.318299<br>H -3.916222 1.059975 -0.734815

Table S6 Quantum chemistry results for reactions relevant to the atmospheric fate of the $HONNC(CH_3)_2CH_2OH$ + OH reaction. Electronic energies of reactants, intermediates and products (/Hartree), and relative energies including Zero Point Energies, $\Delta E_{v=0}$ (/kJ mol^{-1}), of stationary points on the potential energy surface.

	M06-2X/aug-cc-pVTZ		
Species	E_{Elec}	E_{ZPE}	$\Delta E_{v=0}$
$HONNC(CH_3)_2CH_2OH$ (*sas*)	-418.313896	0.152522	
OH	-75.733810	0.008588	
Sum Reactants	-494.047706	0.161110	0
PRE-11 (*sas*)	-494.060028	0.163909	-25.0
SP-11 (*sas*)	-494.056482	0.160772	-23.9
POST-11 (*sas*)	-494.116250	0.164874	-170.1
$ON\dot{N}$-$C(CH_3)_2CH_2OH$	-417.672099	0.139873	
H_2O	-76.430107	0.021551	
Sum products	-494.102206	0.161424	-142.3
$ONNHC(CH_3)_2CH_2OH$ (*asa*)	-418.306207	0.152282	
OH	-75.733810	0.008588	
Sum Reactants	-494.040017	0.160870	19.6
PRE-11 (*asa*)	-494.050875	0.163432	-2.2
SP-11 (*asa*)	-494.032726	0.157239	29.2
POST-11 (*asa*)	-494.104360	0.163357	-142.8
$ONNHC(CH_3)_2CH_2OH$ (*aas*)	-418.312821	0.152163	
OH	-75.733810	0.008588	
Sum Reactants	-494.046631	0.160751	1.9
PRE-11 (*aas*)	-494.063080	0.164830	-30.6
SP-11 (*aas*)	-494.061365	0.161582	-34.6
POST-11 (*aas*)	-494.115187	0.164752	-167.6
$ONNHC(CH_3)_2CH_2OH$ (*aaa*)	-418.312125	0.152349	
OH	-75.733810	0.008588	
Sum Reactants	-494.045935	0.160937	4.2
PRE-11 (*aaa*)	-494.05575	0.163385	-15.1
SP-11 (*aaa*)	-494.038056	0.157289	15.3
POST-11 (*aaa*)	-494.108851	0.163424	-154.5
$ONNHC(CH_3)_2CH_2OH$ (*ssa*)	-418.303718	0.152351	
OH	-75.733810	0.008588	
Sum Reactants	-494.037528	0.160939	26.3
PRE-11 (*ssa*)	-494.048601	0.163416	3.7
SP-11 (*ssa*)	-494.028967	0.157224	39.0
POST-11 (*ssa*)	-494.108598	0.164381	-151.3
$ONNHC(CH_3)_2CH_2OH$ (*sss*)	-418.303718	0.152351	
OH	-75.733810	0.008588	
Sum Reactants	-494.037528	0.160939	26.3

PRE-11 (*sss*)	-494.045287	0.163197	11.8
SP-11 (*sss*)	-494.026870	0.156766	43.3
POST-11 (*sss*)	-494.099946	0.163228	-131.6

Table S6, continued: Vibrational frequencies (/cm^{-1}), Rotational constants (B /GHZ) and Cartesian Coordinates (/Å) of the species listed above. Results from M06-2X/aug-cc-pVTZ calculations.

$HONNC(CH_3)_2CH_2OH$ (*sas*)<br><br>$\tilde{\nu}$: 86.2, 159.9, 200.6, 214.6, 237.6, 259.6, 283.9, 339.6, 363.6, 405.7, 462.4, 513.5, 583.3, 591.5, 616.5, 836.0, 910.7, 962.5, 970.6, 995.6, 1031.9, 1079.4, 1131.0, 1181.2, 1225.8, 1287.1, 1300.9, 1392.2, 1401.9, 1410.7, 1428.1, 1434.7, 1481.6, 1488.0, 1500.5, 1502.2, 1515.7, 1733.8, 3047.6, 3064.8, 3070.8, 3109.0, 3138.4, 3144.4, 3150.5, 3158.8, 3680.8, 3863.6<br><br>B: 2.5800000 0.0037462 1.6202958	C -0.704269 -0.385856 0.007550<br>C -1.256422 -1.524291 -0.844284<br>C -0.956280 0.944064 -0.706336<br>N 0.734741 -0.693871 0.201662<br>C -1.328357 -0.394065 1.398250<br>N 1.501263 0.176314 -0.157401<br>O 2.810075 -0.159838 0.037194<br>O -0.587026 2.080377 0.038022<br>H -1.047925 -2.483392 -0.372977<br>H -0.805075 -1.521223 -1.836945<br>H -2.335409 -1.415098 -0.951278<br>H -2.029771 1.023603 -0.882431<br>H -0.458460 0.928515 -1.680712<br>H 2.801252 -1.050898 0.430018<br>H -2.408715 -0.277181 1.312979<br>H -0.939210 0.428056 1.995230<br>H -1.114739 -1.336492 1.900825<br>H 0.373609 2.123590 0.042657
OH<br>3769.6; B: 564.2863408	O 0.000000 0.000000 0.107992<br>H 0.000000 0.000000 -0.863937
PRE(*sas*)<br><br>$\tilde{\nu}$: 31.5, 91.3, 119.0, 142.4, 169.4, 215.5, 227.0, 257.4, 259.4, 297.0, 313.3, 340.0, 371.5, 415.5, 456.7, 479.6, 517.1, 590.1, 595.2, 793.0, 840.0, 912.5, 960.6, 989.2, 998.1, 1032.3, 1112.9, 1131.0, 1179.8, 1223.1, 1285.9, 1297.6, 1392.8, 1402.2, 1411.2, 1430.9, 1481.4, 1486.3, 1492.6, 1499.6, 1501.2, 1514.6, 1717.1, 3050.8, 3064.6, 3067.9, 3109.9, 3138.6, 3140.3, 3148.6, 3158.4, 3438.6, 3793.0, 3862.6<br><br>B: 1.9700000 0.0068974 1.1729779	C 1.794604 -0.471769 -0.520001<br>C 0.685120 1.682530 -1.112457<br>C 1.102116 1.126198 1.302978<br>C 0.763772 0.567895 -0.074018<br>N -0.609025 0.019633 0.026480<br>N -0.741838 -1.160210 -0.237618<br>O -2.016709 -1.595547 -0.166006<br>H 2.748272 0.044130 -0.639311<br>H 1.504118 -0.869570 -1.496565<br>H -0.093925 2.394365 -0.843005<br>H 0.452654 1.277798 -2.097673<br>H 1.639146 2.206696 -1.164067<br>H 0.349090 1.851515 1.611855<br>H 2.069339 1.626994 1.266437<br>H 1.155712 0.325622 2.037794<br>H -2.570802 -0.806072 0.033291<br>O -2.827238 1.010824 0.224715<br>H -2.426080 1.310565 1.055617<br>O 2.007480 -1.504710 0.411420<br>H 1.246572 -2.091667 0.373553
TS(*sas*)<br><br>$\tilde{\nu}$: -1068.6, 36.9, 95.9, 136.8, 156.5, 210.9, 225.5, 257.0, 289.0, 337.4, 341.5, 366.2, 422.3, 432.2, 458.0, 496.1, 571.8,	C 1.732592 -0.576428 -0.552820<br>C 0.784666 1.679399 -1.076170<br>C 1.214787 1.039849 1.321203<br>C 0.805705 0.542933 -0.059262

593.4, 665.2, 762.8, 845.0, 911.9, 961.8, 999.6, 1013.7, 1033.9, 1136.3, 1177.2, 1219.0, 1264.4, 1273.2, 1327.3, 1372.9, 1392.9, 1403.9, 1409.0, 1429.2, 1480.4, 1487.2, 1496.8, 1500.7, 1514.3, 1637.5, 1814.9, 3053.2, 3068.4, 3071.7, 3110.7, 3143.6, 3145.7, 3154.5, 3162.1, 3845.8, 3856.9<br><br>B: 2.0700000 0.0024906 1.2055512	N -0.582819 0.063084 0.048911<br>N -0.904011 -1.081053 -0.217606<br>O -2.174532 -1.338019 -0.158568<br>H 2.723951 -0.137462 -0.674131<br>H 1.387738 -0.916242 -1.533392<br>H 0.063024 2.436926 -0.775511<br>H 0.504264 1.311302 -2.062818<br>H 1.773187 2.133799 -1.135374<br>H 0.517773 1.802524 1.667274<br>H 2.210721 1.478607 1.267443<br>H 1.235627 0.217123 2.032511<br>H -2.601316 -0.313842 0.055334<br>O -2.602801 0.979865 0.202453<br>H -2.415564 1.207083 1.121580<br>O 1.866506 -1.645392 0.346704<br>H 1.068517 -2.180185 0.295523
POST(*sas*)<br><br>$\tilde{\upsilon}$: 45.5, 79.1, 126.3, 139.8, 183.4, 204.2, 211.0, 236.7, 244.8, 279.1, 313.2, 329.1, 352.8, 380.1, 396.3, 405.5, 490.5, 584.0, 597.1, 646.9, 830.2, 920.7, 945.0, 962.1, 1008.9, 1038.9, 1146.8, 1193.4, 1236.1, 1254.9, 1315.2, 1391.2, 1402.3, 1415.3, 1429.3, 1452.8, 1484.1, 1488.2, 1500.9, 1503.7, 1514.3, 1630.6, 1742.0, 3035.8, 3066.6, 3070.5, 3096.5, 3140.4, 3144.4, 3146.1, 3159.2, 3715.6, 3829.7, 3914.4<br><br>B: 1.8700000 0.0028766 1.2373601	C -0.814677 -1.420571 0.018325<br>C -2.162723 0.295996 1.240385<br>C -1.626573 0.629738 -1.198137<br>C -1.141548 0.079769 0.133550<br>N 0.117135 0.717819 0.592275<br>N 0.802848 1.254569 -0.257753<br>O 1.891906 1.764306 -0.241075<br>H -1.732903 -1.938297 -0.266845<br>H -0.510097 -1.790722 1.002978<br>H -2.410441 1.352854 1.332118<br>H -1.774555 -0.051861 2.197150<br>H -3.075435 -0.253537 1.011373<br>H -1.750405 1.711476 -1.144930<br>H -2.591139 0.183120 -1.437922<br>H -0.925495 0.387408 -1.994081<br>H 3.062289 -0.455671 0.100862<br>O 2.530324 -1.106990 0.568836<br>H 2.092565 -0.602039 1.262741<br>O 0.154937 -1.689848 -0.954954<br>H 1.031533 -1.598784 -0.552288
$HONNC(CH_3)_2CH_2OH$ (*asa*)<br><br>$\tilde{\upsilon}$: 61.5, 123.5, 193.0, 207.5, 244.5, 276.7, 279.3, 328.5, 355.3, 381.5, 421.5, 515.8, 571.1, 604.7, 613.7, 832.4, 920.8, 957.8, 962.4, 1006.1, 1037.3, 1067.0, 1127.6, 1183.6, 1247.2, 1277.1, 1286.4, 1380.2, 1402.8, 1414.8, 1418.0, 1438.8, 1482.7, 1487.1, 1503.1, 1508.2, 1514.9, 1734.4, 3029.3, 3067.9, 3071.4, 3113.8, 3140.1, 3143.0, 3157.3, 3160.5, 3684.2, 3855.4<br><br>B: 2.8200000 0.0013506 1.4390873	C 0.508353 0.449250 0.019371<br>C 0.305004 1.750760 -0.739366<br>C 1.517224 -0.432019 -0.726098<br>N -0.715440 -0.384184 0.114133<br>C 0.969427 0.704570 1.451189<br>N -1.755471 0.203110 -0.104550<br>O -2.848720 -0.603707 0.011163<br>O 1.775598 -1.652597 -0.075162<br>H -0.395562 2.402542 -0.222221<br>H -0.082480 1.565391 -1.740971<br>H 1.261994 2.265635 -0.823054<br>H 2.466975 0.099564 -0.788521<br>H 1.151971 -0.597990 -1.746028<br>H 1.914805 1.247018 1.436275<br>H 1.117555 -0.232109 1.983888<br>H 0.229186 1.304281 1.980183<br>H -2.514124 -1.483301 0.260394<br>H 0.930981 -2.088441 0.074381

PRE(*asa*)<br><br>$\tilde{\nu}$: 39.4, 79.3, 87.6, 148.7, 161.3, 195.5, 216.7, 222.6, 227.4, 293.9, 308.5, 337.8, 382.0, 403.5, 457.1, 527.9, 545.8, 574.0, 657.3, 711.5, 818.6, 878.0, 923.7, 931.1, 967.7, 1000.9, 1046.6, 1128.4, 1181.7, 1225.7, 1269.5, 1285.9, 1383.8, 1399.2, 1410.3, 1428.2, 1435.5, 1484.9, 1494.6, 1502.6, 1509.0, 1515.4, 1792.7, 3029.2, 3067.5, 3074.7, 3113.9, 3131.1, 3146.3, 3178.8, 3185.0, 3639.9, 3738.5, 3841.5<br><br>B: 2.7200000 0.0025713 0.8029173	C 0.953253 0.370960 -0.032434<br>C 0.761628 1.642661 -0.857299<br>C 2.237933 -0.321778 -0.507151<br>N -0.092058 -0.654444 -0.301208<br>C 1.036798 0.648462 1.466911<br>N -1.280665 -0.476135 -0.172418<br>O -1.669572 0.794753 0.258222<br>O 2.454162 -1.561594 0.118509<br>H 0.038857 2.314749 -0.407753<br>H 0.436264 1.402898 -1.869745<br>H 1.722440 2.155551 -0.925317<br>H 3.090580 0.311326 -0.261281<br>H 2.195035 -0.431896 -1.596717<br>H 1.864757 1.336322 1.642875<br>H 1.238322 -0.271595 2.011940<br>H 0.121817 1.101476 1.836788<br>H -2.635551 0.713175 0.292199<br>H 1.682676 -2.109300 -0.061148<br>O -4.129191 -0.666153 -0.063181<br>H -3.326998 -1.186529 -0.275014
TS(*asa*)<br><br>$\tilde{\nu}$: -2339.1, 56.6, 86.2, 99.9, 151.6, 181.4, 209.0, 216.8, 234.8, 247.7, 299.2, 317.2, 381.5, 388.7, 452.4, 463.4, 555.7, 629.6, 695.8, 777.9, 805.3, 825.5, 891.9, 926.0, 966.5, 995.6, 1044.1, 1095.6, 1128.0, 1181.6, 1224.9, 1270.2, 1287.5, 1385.3, 1401.3, 1411.8, 1436.4, 1484.9, 1491.6, 1502.6, 1509.3, 1514.0, 1536.4, 1735.5, 3030.4, 3066.2, 3073.7, 3114.7, 3131.7, 3146.1, 3169.9, 3174.3, 3776.3, 3839.6<br><br>B: 3.0700000 0.0034588 0.8137719	C 0.888506 0.388338 -0.017113<br>C 0.505073 1.650331 -0.787261<br>C 2.282312 -0.057874 -0.477818<br>N 0.033870 -0.777998 -0.371590<br>C 0.882012 0.598761 1.494948<br>N -1.174481 -0.795073 -0.259508<br>O -1.765895 0.353668 0.213322<br>O 2.683600 -1.274473 0.099991<br>H -0.348401 2.156091 -0.346825<br>H 0.276452 1.416196 -1.827015<br>H 1.356269 2.333083 -0.773923<br>H 3.011415 0.693886 -0.175104<br>H 2.286955 -0.119757 -1.571900<br>H 1.572240 1.408592 1.734223<br>H 1.221337 -0.300729 2.004568<br>H -0.107207 0.870851 1.854116<br>H -2.822662 0.086058 0.363132<br>H 2.022211 -1.933926 -0.135094<br>O -3.934181 -0.326357 0.005414<br>H -3.699939 -1.098886 -0.534844
POST(*asa*)<br><br>$\tilde{\nu}$: 41.7, 70.0, 84.2, 119.0, 128.0, 139.2, 187.4, 196.0, 214.5, 234.8, 244.4, 301.2, 312.9, 334.2, 381.0, 400.4, 423.3, 542.0, 585.1, 686.5, 829.7, 862.6, 923.6, 962.4, 997.0, 1035.1, 1122.9, 1163.6, 1220.1, 1258.8, 1290.0, 1344.2, 1387.3, 1407.0, 1413.9, 1436.3, 1482.4, 1484.2, 1503.2, 1504.6, 1510.2, 1613.9, 1775.7, 3036.5, 3067.4, 3074.9, 3114.6, 3135.1, 3145.8, 3165.0, 3169.5, 3842.0, 3849.1, 3950.9<br><br>B: 2.3400000 0.0038227 0.9517378	C 0.626786 -0.305958 0.187691<br>C -0.179166 -0.816687 1.371439<br>C 2.111727 -0.597168 0.395957<br>N 0.575987 1.211099 0.141315<br>C 0.130380 -0.855603 -1.140494<br>N -0.496908 1.745550 0.050345<br>O -1.669882 1.448142 -0.022128<br>O 2.922924 -0.056521 -0.616702<br>H -1.247941 -0.677848 1.224857<br>H 0.131375 -0.325470 2.293558<br>H -0.001983 -1.888098 1.474689<br>H 2.259215 -1.677381 0.378373<br>H 2.409232 -0.225382 1.382182

	H 0.187759 -1.944207 -1.108063<br>H 0.754019 -0.498902 -1.957391<br>H -0.909962 -0.590418 -1.318444<br>H -3.036322 -0.090690 -0.267036<br>H 2.798128 0.897934 -0.613725<br>O -3.234477 -1.032316 -0.276430<br>H -4.183956 -1.098033 -0.396089
HONNC$(CH_3)_2CH_2$OH (*aas*)<br><br>$\tilde{\nu}$: 61.5, 123.5, 193.0, 207.5, 244.5, 276.7, 279.3, 328.5, 355.3, 381.5, 421.5, 515.8, 571.1, 604.7, 613.7, 832.4, 920.8, 957.8, 962.4, 1006.1, 1037.3, 1067.0, 1127.6, 1183.6, 1247.2, 1277.1, 1286.4, 1380.2, 1402.8, 1414.8, 1418.0, 1438.8, 1482.7, 1487.1, 1503.1, 1508.2, 1514.9, 1734.4, 3029.3, 3067.9, 3071.4, 3113.8, 3140.1, 3143.0, 3157.3, 3160.5, 3684.2, 3855.4<br><br>B: 2.8200000 0.0013506 1.4390873	C 0.508353 0.449250 0.019371<br>C 0.305004 1.750760 -0.739366<br>C 1.517224 -0.432019 -0.726098<br>N -0.715440 -0.384184 0.114133<br>C 0.969427 0.704570 1.451189<br>N -1.755471 0.203110 -0.104550<br>O -2.848720 -0.603707 0.011163<br>O 1.775598 -1.652597 -0.075162<br>H -0.395562 2.402542 -0.222221<br>H -0.082480 1.565391 -1.740971<br>H 1.261994 2.265635 -0.823054<br>H 2.466975 0.099564 -0.788521<br>H 1.151971 -0.597990 -1.746028<br>H 1.914805 1.247018 1.436275<br>H 1.117555 -0.232109 1.983888<br>H 0.229186 1.304281 1.980183<br>H -2.514124 -1.483301 0.260394<br>H 0.930981 -2.088441 0.074381
PRE(*aas*)<br><br>$\tilde{\nu}$: 54.7, 113.0, 145.5, 159.4, 183.4, 218.4, 245.7, 265.5, 280.6, 285.2, 343.1, 358.0, 396.6, 438.0, 506.0, 573.8, 602.6, 634.9, 682.3, 837.3, 915.1, 919.1, 960.8, 979.0, 1014.9, 1046.6, 1100.2, 1142.0, 1190.8, 1244.5, 1256.7, 1298.6, 1392.4, 1406.1, 1423.1, 1450.0, 1481.8, 1484.7, 1499.9, 1504.1, 1506.4, 1515.6, 1703.1, 3028.7, 3070.2, 3073.6, 3105.5, 3143.9, 3150.3, 3153.6, 3160.2, 3189.2, 3716.7, 3800.8<br><br>B: 1.9000000 0.0014298 1.3470793	C -0.898028 -0.445864 0.085132<br>C -1.263549 -1.820433 -0.452397<br>C -1.398744 0.647069 -0.877387<br>N 0.561666 -0.241988 0.155194<br>C -1.441464 -0.220897 1.490297<br>N 1.316178 -1.126976 -0.191181<br>O 2.607603 -0.774307 -0.171170<br>O -1.127352 1.948913 -0.439607<br>H -0.872598 -2.606379 0.191267<br>H -0.864933 -1.972496 -1.454370<br>H -2.349233 -1.904802 -0.486906<br>H -2.482525 0.549719 -0.955746<br>H -0.968065 0.455522 -1.866961<br>H -2.527916 -0.301949 1.472836<br>H -1.179042 0.772860 1.846914<br>H -1.046239 -0.969657 2.176199<br>H 2.604749 0.201871 0.026140<br>H -0.166375 2.060547 -0.386634<br>O 1.677364 1.651592 0.240126<br>H 1.457069 1.728677 1.180513
TS(*aas*)<br><br>$\tilde{\nu}$: -815.2, 57.0, 115.4, 166.8, 173.3, 228.6, 248.4, 260.5, 285.9, 328.3, 345.1, 353.0, 433.0, 444.3, 492.5, 563.5, 613.0, 656.0, 703.7, 830.2, 841.3, 919.9, 961.3, 979.9, 1015.4, 1051.3, 1144.7, 1183.5, 1232.1, 1249.1, 1295.0, 1309.2, 1358.1, 1393.3, 1404.1, 1424.7, 1458.1, 1481.3, 1485.6, 1500.2, 1504.7, 1512.6, 1645.8, 1869.7, 3028.3, 3071.0,	C -0.972421 -0.379215 0.077865<br>C -1.425613 -1.704150 -0.517554<br>C -1.303601 0.783406 -0.879120<br>N 0.490807 -0.328881 0.212029<br>C -1.563891 -0.134118 1.458843<br>N 1.251060 -1.197404 -0.161968<br>O 2.512463 -0.879049 -0.154489<br>O -0.878876 2.027793 -0.403861

3073.5, 3105.2, 3145.7, 3151.5, 3154.2, 3162.2, 3676.2, 3839.0<br><br>B: 1.9500000 0.0075712 1.3392389	H -1.140335 -2.534500 0.126038<br>H -0.986827 -1.864550 -1.501080<br>H -2.511113 -1.689858 -0.610516<br>H -2.388462 0.824435 -0.988460<br>H -0.869755 0.553495 -1.859041<br>H -2.650670 -0.114484 1.384109<br>H -1.228455 0.823919 1.849829<br>H -1.274831 -0.929020 2.145430<br>H 2.465210 0.208428 0.048994<br>H 0.089345 2.014181 -0.331465<br>O 1.882869 1.407315 0.229397<br>H 1.764335 1.547936 1.177150
POST(*aas*)<br><br>$\tilde{\nu}$: 41.0, 62.9, 119.0, 134.7, 186.1, 193.7, 214.9, 239.5, 261.3, 279.3, 311.4, 326.0, 337.1, 376.7, 391.3, 409.1, 484.1, 579.1, 593.1, 658.7, 833.2, 924.3, 948.7, 961.2, 1008.4, 1038.6, 1143.3, 1191.7, 1221.2, 1279.9, 1309.7, 1394.6, 1401.9, 1409.6, 1424.8, 1443.7, 1481.5, 1488.7, 1500.2, 1501.8, 1516.4, 1616.9, 1764.5, 3038.1, 3065.1, 3071.8, 3083.4, 3139.1, 3144.4, 3149.9, 3163.4, 3710.4, 3806.2, 3942.3<br><br>B: 2.1100000 0.0013678 1.1093638	C -1.276433 0.013707 0.062936<br>C -2.219062 0.472581 1.168611<br>C -0.445615 -1.177419 0.550618<br>N -0.431847 1.192039 -0.283277<br>C -2.042999 -0.341333 -1.204416<br>N 0.758780 1.093345 -0.097525<br>O 1.714094 1.814577 -0.264124<br>O 0.401178 -1.691781 -0.442186<br>H -2.783733 1.345862 0.845975<br>H -1.663862 0.732835 2.070159<br>H -2.920841 -0.326027 1.408107<br>H -1.153595 -1.956453 0.846546<br>H 0.118664 -0.883106 1.441201<br>H -2.753448 -1.138989 -0.987090<br>H -1.356802 -0.686004 -1.974752<br>H -2.589886 0.526690 -1.569884<br>H 2.997149 0.185375 0.118200<br>H 1.315644 -1.446102 -0.239577<br>O 2.958397 -0.751945 0.344417<br>H 3.817497 -1.123795 0.135375
HONNC(CH_3)$_2$$CH_2$OH (*aaa*)<br><br>$\tilde{\nu}$: 72.2, 126.1, 197.2, 217.7, 243.7, 275.5, 281.1, 335.5, 356.1, 406.4, 440.1, 486.7, 516.6, 570.9, 608.4, 828.8, 921.1, 951.2, 960.3, 1003.1, 1029.4, 1058.4, 1129.9, 1187.4, 1246.2, 1276.0, 1287.3, 1379.6, 1388.6, 1404.0, 1414.3, 1437.8, 1482.5, 1487.2, 1502.6, 1508.6, 1514.8, 1772.5, 3026.0, 3067.0, 3070.0, 3110.1, 3138.4, 3141.2, 3154.1, 3162.2, 3838.5, 3860.3<br><br>B: 2.8200000 0.0038539 1.4359577	C 0.514743 0.449412 0.014916<br>C 0.290649 1.740751 -0.754973<br>C 1.532756 -0.428026 -0.724456<br>N -0.687916 -0.410129 0.125163<br>C 0.977872 0.724041 1.442665<br>N -1.734657 0.157440 -0.084487<br>O -2.786839 -0.729357 0.053997<br>O 1.788779 -1.644626 -0.068235<br>H -0.411032 2.391948 -0.238131<br>H -0.102570 1.540699 -1.751636<br>H 1.241109 2.265282 -0.853046<br>H 2.480564 0.107803 -0.784670<br>H 1.171539 -0.598022 -1.745436<br>H 1.913631 1.282714 1.418932<br>H 1.143070 -0.206436 1.980858<br>H 0.229676 1.314466 1.971120<br>H -3.559255 -0.185964 -0.133655<br>H 0.939646 -2.068866 0.095918
PRE(*aaa*)	C 0.894316 0.479099 0.078593<br>C 0.233892 1.724730 -0.490119<br>C 1.999129 -0.003026 -0.871763

ṽ: 28.0, 67.1, 108.2, 125.5, 177.3, 209.5, 212.4, 245.0, 272.6, 288.5, 290.2, 335.2, 356.6, 400.0, 433.2, 515.0, 542.5, 561.7, 576.2, 613.2, 830.4, 918.5, 955.0, 961.0, 1004.9, 1035.8, 1071.5, 1131.3, 1187.0, 1247.4, 1273.0, 1287.3, 1379.8, 1402.0, 1411.8, 1434.8, 1459.0, 1483.1, 1487.3, 1503.4, 1509.1, 1515.4, 1774.9, 3025.8, 3065.0, 3069.2, 3111.1, 3138.2, 3139.6, 3143.7, 3164.3, 3638.7, 3758.0, 3842.4<br><br>B: 2.4700000 0.0037527 0.8253974	N -0.007124 -0.689383 0.205263<br>C 1.459105 0.736303 1.472602<br>N -1.185797 -0.434049 0.103857<br>O -1.949848 -1.565546 0.236414<br>O 2.664643 -1.148916 -0.404977<br>H -0.529249 2.111378 0.183955<br>H -0.227598 1.523149 -1.457253<br>H 0.989431 2.498819 -0.622255<br>H 2.748007 0.783943 -0.965917<br>H 1.559059 -0.175341 -1.861008<br>H 2.197671 1.536417 1.422477<br>H 1.940738 -0.156734 1.863463<br>H 0.661407 1.037417 2.151437<br>H -2.854329 -1.234931 0.126482<br>H 1.998432 -1.817674 -0.213767<br>O -3.813813 0.625751 -0.289368<br>H -2.859630 0.844641 -0.263889
TS(*aaa*)<br><br>ṽ: -2172.0, 34.9, 84.4, 119.9, 127.8, 175.4, 206.5, 241.6, 249.2, 274.4, 283.2, 338.2, 350.6, 364.9, 433.8, 462.2, 519.5, 582.6, 606.3, 785.5, 823.2, 917.8, 927.1, 959.7, 979.6, 1002.6, 1044.5, 1127.3, 1131.1, 1185.4, 1248.5, 1274.3, 1286.6, 1381.3, 1402.5, 1413.1, 1435.4, 1482.5, 1486.9, 1503.0, 1508.0, 1514.4, 1533.2, 1733.5, 3026.5, 3067.0, 3069.7, 3109.4, 3138.5, 3141.2, 3151.7, 3163.6, 3789.8, 3842.6<br><br>B: 2.7500000 0.0014344 0.7739207	C 1.015542 0.464319 0.035152<br>C 0.578702 1.753409 -0.640637<br>C 2.061873 -0.250360 -0.830412<br>N -0.066983 -0.536187 0.189423<br>C 1.562686 0.724992 1.435525<br>N -1.189230 -0.082405 0.095578<br>O -2.122095 -1.053619 0.258222<br>O 2.519681 -1.450996 -0.261665<br>H -0.146099 2.292099 -0.033539<br>H 0.130986 1.555092 -1.614712<br>H 1.449959 2.392632 -0.782552<br>H 2.929313 0.401268 -0.939411<br>H 1.636045 -0.420587 -1.826098<br>H 2.423869 1.389967 1.369591<br>H 1.877226 -0.203568 1.906015<br>H 0.799782 1.198466 2.053155<br>H -3.089083 -0.530402 0.283076<br>H 1.749548 -1.996327 -0.069578<br>O -3.993819 0.159758 -0.204280<br>H -3.511008 0.856195 -0.676937
POST(*aaa*)<br><br>ṽ: 20.3, 37.2, 60.8, 108.5, 124.8, 133.4, 204.5, 229.8, 252.9, 264.9, 275.0, 291.5, 315.2, 338.1, 354.6, 391.9, 404.6, 485.6, 580.5, 601.5, 830.6, 925.4, 939.4, 963.8, 1008.4, 1040.7, 1122.2, 1172.0, 1222.2, 1269.2, 1301.3, 1388.1, 1401.9, 1408.3, 1414.0, 1432.6, 1483.2, 1491.8, 1504.9, 1506.6, 1516.0, 1622.1, 1759.8, 3045.4, 3062.8, 3068.8, 3119.3, 3139.6, 3143.8, 3148.2, 3154.3, 3840.2, 3862.9, 3949.3<br><br>B: 2.1800000 0.0043287 1.0014039	C 0.736710 0.504264 -0.068590<br>C 0.317165 1.724526 -0.881325<br>C 2.213887 0.200287 -0.314855<br>N 0.012170 -0.663617 -0.648001<br>C 0.443327 0.696048 1.411165<br>N -0.969772 -1.041282 -0.043809<br>O -1.809029 -1.882904 -0.230793<br>O 2.657113 -0.955331 0.358422<br>H -0.743532 1.920515 -0.728531<br>H 0.496519 1.562737 -1.943891<br>H 0.889045 2.594358 -0.557046<br>H 2.808281 1.031737 0.063844<br>H 2.383926 0.118166 -1.392924<br>H 1.025489 1.539618 1.781613<br>H 0.723706 -0.190174 1.977974<br>H -0.614472 0.907121 1.565032<br>H -3.230805 -0.076508 0.129187

	H 2.222349 -1.718624 -0.032489<br>O -3.050193 0.863199 0.231834<br>H -3.906969 1.294877 0.225825
$HONNC(CH_3)_2CH_2OH$ (*ssa*)<br><br>$\tilde{\nu}$: 101.2, 162.3, 228.0, 243.4, 265.4, 301.8, 310.4, 325.3, 374.6, 404.9, 443.5, 499.2, 552.8, 634.3, 681.6, 704.2, 824.7, 876.0, 909.5, 963.5, 1020.4, 1036.5, 1146.5, 1177.6, 1225.9, 1259.2, 1281.9, 1313.5, 1386.8, 1404.0, 1421.9, 1451.8, 1482.0, 1489.5, 1498.5, 1502.4, 1515.8, 1809.7, 3037.6, 3064.2, 3076.5, 3113.6, 3137.2, 3146.2, 3155.5, 3178.3, 3866.2, 3868.8<br><br>B: 2.4200000 0.0056189 2.0338391	C 0.719279 -0.175470 0.020971<br>C 0.704211 0.027016 1.532612<br>C 0.335487 1.112617 -0.752209<br>N -0.142959 -1.353531 -0.302612<br>C 2.106961 -0.610353 -0.443110<br>N -1.339843 -1.392896 -0.273679<br>O -1.985495 -0.148106 0.091728<br>O -0.419374 2.060429 -0.048288<br>H 1.059836 -0.874885 2.029578<br>H -0.290193 0.269815 1.895356<br>H 1.368190 0.853994 1.781419<br>H -0.144559 0.826610 -1.694353<br>H 1.269877 1.615202 -1.000403<br>H 2.848093 0.127821 -0.139477<br>H 2.134017 -0.706215 -1.529538<br>H 2.364673 -1.574972 -0.010018<br>H -2.916139 -0.390988 0.056624<br>H -1.270862 1.657161 0.147740
PRE(*ssa*)<br><br>$\tilde{\nu}$: 39.8, 81.4, 102.8, 151.0, 162.7, 232.3, 236.5, 255.2, 277.2, 303.4, 314.3, 334.7, 378.5, 408.9, 492.2, 506.3, 538.9, 561.9, 644.1, 700.2, 766.2, 835.9, 891.2, 909.8, 962.4, 1019.2, 1035.9, 1146.9, 1177.2, 1226.6, 1258.1, 1285.6, 1383.3, 1401.0, 1412.1, 1421.1, 1451.0, 1481.7, 1489.9, 1498.1, 1501.8, 1516.2, 1802.8, 3036.2, 3064.7, 3077.0, 3113.1, 3137.7, 3147.4, 3155.8, 3176.3, 3646.2, 3729.5, 3851.0<br><br>B: 2.3600000 0.0078201 0.9239060	C 1.152698 -0.401406 0.009172<br>C 1.296790 -0.179268 1.511665<br>C 1.252079 0.921019 -0.795643<br>N -0.110618 -1.162484 -0.232410<br>C 2.240830 -1.345979 -0.494538<br>N -1.223633 -0.715329 -0.147486<br>O -1.323079 0.663979 0.188170<br>O 0.968897 2.100038 -0.093631<br>H 1.307477 -1.138834 2.027474<br>H 0.491776 0.433531 1.906695<br>H 2.238345 0.334823 1.700516<br>H 0.642618 0.828636 -1.701164<br>H 2.291118 1.015742 -1.109771<br>H 3.223190 -0.943957 -0.250320<br>H 2.172136 -1.466240 -1.576719<br>H 2.131961 -2.326785 -0.035476<br>H -2.284291 0.796025 0.202825<br>H 0.039999 2.070017 0.154469<br>O -4.041174 -0.201136 -0.070342<br>H -3.406105 -0.927505 -0.236776
TS(*ssa*)<br><br>$\tilde{\nu}$: -2263.6, 58.3, 91.5, 114.4, 136.9, 204.7, 228.2, 250.7, 260.3, 280.8, 304.0, 312.3, 375.7, 396.9, 426.0, 474.9, 542.4, 627.5, 685.0, 709.9, 788.6, 821.9, 865.3, 907.8, 962.7, 1015.1, 1034.2, 1089.7, 1143.1, 1172.3, 1228.6, 1257.3, 1280.9, 1384.6, 1402.7, 1420.3, 1443.6, 1481.4, 1488.4, 1497.2, 1501.4, 1514.5, 1544.1, 1745.7, 3038.5, 3065.1, 3076.0, 3115.6, 3138.3, 3147.3, 3155.9, 3170.7, 3774.7, 3859.3<br><br>B: 2.4100000 0.0001407 1.0080780	C 1.189559 -0.285387 0.009685<br>C 1.251098 -0.062798 1.517209<br>C 1.021336 1.041856 -0.776698<br>N 0.121363 -1.288906 -0.285084<br>C 2.463727 -0.968244 -0.479411<br>N -1.065543 -1.082831 -0.193851<br>O -1.439300 0.209549 0.198020<br>O 0.482161 2.125774 -0.068030<br>H 1.441759 -1.008630 2.023078<br>H 0.331408 0.371161 1.899765<br>H 2.064961 0.627040 1.737105<br>H 0.463597 0.839320 -1.697093

	H 2.022572 1.359930 -1.065379<br>H 3.333392 -0.371914 -0.206690<br>H 2.444192 -1.082135 -1.564226<br>H 2.555709 -1.957740 -0.035502<br>H -2.548148 0.140453 0.354145<br>H -0.447682 1.947354 0.095973<br>O -3.671387 -0.051526 -0.040474<br>H -3.578618 -0.885623 -0.529469
POST(*ssa*)<br><br>ṽ: 39.6, 77.3, 117.3, 125.5, 181.9, 193.4, 214.1, 227.8, 239.1, 260.2, 304.2, 320.1, 341.2, 394.6, 404.8, 420.7, 497.1, 585.5, 610.3, 679.9, 830.5, 854.9, 923.9, 962.6, 997.7, 1033.9, 1144.7, 1172.2, 1217.0, 1263.4, 1297.4, 1320.8, 1396.8, 1402.3, 1426.8, 1445.7, 1481.3, 1490.4, 1501.1, 1502.4, 1516.0, 1611.5, 1773.2, 3042.2, 3065.5, 3071.5, 3088.5, 3137.3, 3144.6, 3148.8, 3165.1, 3739.4, 3805.8, 3944.6<br><br>B: 2.0100000 0.0082933 1.2626430	C -1.151283 0.053104 0.029969<br>C -2.173611 0.883871 -0.722591<br>C -0.189386 0.951150 0.813561<br>N -0.393584 -0.707579 -1.046866<br>C -1.806331 -0.964316 0.953102<br>N 0.541055 -1.383457 -0.726997<br>O 1.159805 -1.696039 0.281114<br>O 0.465065 1.885614 -0.000001<br>H -2.808019 0.241523 -1.331476<br>H -1.669249 1.601830 -1.365243<br>H -2.798211 1.425117 -0.011596<br>H 0.523353 0.341482 1.374471<br>H -0.802501 1.488951 1.543376<br>H -2.375852 -0.435811 1.718268<br>H -1.066137 -1.591088 1.449392<br>H -2.492264 -1.598855 0.393681<br>H 2.726776 -0.384845 0.195353<br>H 1.367679 1.573198 -0.153556<br>O 2.940862 0.520834 -0.059096<br>H 3.759944 0.749629 0.383991
HONNC(CH_3)$_2$$CH_2$OH (*sss*)<br><br>ṽ: 101.2, 162.3, 228.0, 243.4, 265.4, 301.8, 310.4, 325.3, 374.6, 404.9, 443.5, 499.2, 552.8, 634.3, 681.6, 704.2, 824.7, 876.0, 909.5, 963.5, 1020.4, 1036.5, 1146.5, 1177.6, 1225.9, 1259.2, 1281.9, 1313.5, 1386.8, 1404.0, 1421.9, 1451.8, 1482.0, 1489.5, 1498.5, 1502.4, 1515.8, 1809.7, 3037.6, 3064.2, 3076.5, 3113.6, 3137.2, 3146.2, 3155.5, 3178.3, 3866.2, 3868.8<br><br>B: 2.4200000 0.0056211 2.0338390	C -0.719279 -0.175470 0.020971<br>C -2.106962 -0.610353 -0.443107<br>C -0.335491 1.112617 -0.752208<br>N 0.142960 -1.353530 -0.302616<br>C -0.704208 0.027015 1.532613<br>N 1.339843 -1.392893 -0.273682<br>O 1.985494 -0.148107 0.091731<br>O 0.419375 2.060428 -0.048289<br>H -2.364670 -1.574975 -0.010020<br>H -2.134022 -0.706209 -1.529535<br>H -2.848094 0.127817 -0.139467<br>H -1.269881 1.615204 -1.000396<br>H 0.144549 0.826612 -1.694356<br>H -1.368186 0.853993 1.781420<br>H 0.290196 0.269813 1.895355<br>H -1.059834 -0.874886 2.029579<br>H 2.916138 -0.390988 0.056625<br>H 1.270865 1.657159 0.147732
PRE(*sss*)<br><br>ṽ: 34.6, 56.3, 83.8, 129.8, 167.8, 201.3, 215.0, 251.1, 276.9, 293.1, 302.9, 337.3, 358.1, 400.9, 430.8, 476.8, 544.0, 561.4, 662.2, 704.0, 818.7, 876.1, 921.9, 934.3, 959.2, 1000.5, 1036.5, 1124.6, 1163.7, 1212.0, 1261.6, 1296.0, 1376.9, 1398.4, 1413.7, 1422.1, 1434.4, 1480.3, 1495.2, 1502.7,	C -1.130896 -0.371491 -0.045064<br>C -2.293334 -0.454839 -1.031879<br>C -1.238392 0.927011 0.771490<br>N 0.079382 -0.410497 -0.911965<br>C -1.153999 -1.582900 0.884927<br>N 1.211234 -0.266691 -0.517173<br>O 1.373828 -0.051671 0.856737<br>O -0.917740 2.085324 0.034291

1512.9, 1522.2, 1796.4, 3027.4, 3063.0, 3069.8, 3133.5, 3135.5, 3150.0, 3173.4, 3180.3, 3625.6, 3745.5, 3882.6<br><br>B: 2.3800000 0.0024799 0.9002094	H -2.215724 -1.362978 -1.627479<br>H -2.281238 0.387697 -1.724818<br>H -3.243010 -0.462640 -0.497479<br>H -0.559054 0.907052 1.615907<br>H -2.263794 0.973032 1.156528<br>H -2.102724 -1.597507 1.423043<br>H -0.341535 -1.550332 1.605071<br>H -1.080893 -2.502665 0.305514<br>H 2.333604 0.062207 0.933701<br>H -1.557942 2.206193 -0.670725<br>O 4.028947 0.048662 -0.275779<br>H 3.297438 -0.084949 -0.914140
TS(*sss*)<br><br>$\tilde{\upsilon}$: -2347.2, 33.0, 77.2, 101.6, 130.4, 197.6, 202.9, 215.3, 238.7, 264.2, 279.6, 303.8, 355.2, 391.0, 417.4, 456.4, 499.9, 649.2, 682.6, 776.9,<br>805.1, 811.5, 884.9, 932.3, 958.2, 997.3, 1035.8, 1093.4, 1126.2, 1163.2, 1212.3, 1261.6, 1296.4, 1376.2, 1400.0, 1412.9, 1433.3, 1480.4, 1494.1, 1501.9, 1511.3, 1520.7, 1528.2, 1733.3, 3026.8, 3063.4, 3069.4, 3134.0, 3137.3, 3149.9, 3162.0, 3165.0, 3779.6, 3881.6<br><br>B: 2.4300000 0.0024333 0.9663912	C -1.122626 -0.351081 0.012880<br>C -2.442921 -0.375562 -0.753118<br>C -1.023916 0.941896 0.837868<br>N -0.085233 -0.433109 -1.052947<br>C -1.032324 -1.568050 0.930964<br>N 1.106934 -0.351429 -0.846545<br>O 1.505271 -0.155510 0.458022<br>O -0.810441 2.095594 0.057575<br>H -2.512748 -1.283026 -1.350663<br>H -2.513412 0.468867 -1.440009<br>H -3.283373 -0.343157 -0.060140<br>H -0.195293 0.891941 1.536866<br>H -1.951388 1.018615 1.417526<br>H -1.866898 -1.547500 1.632935<br>H -0.103203 -1.577944 1.495562<br>H -1.102720 -2.485355 0.347618<br>H 2.599892 -0.232536 0.426646<br>H -1.568511 2.244776 -0.512227<br>O 3.740074 0.082659 0.049756<br>H 3.597241 0.271928 -0.892055
POST(*sss*)<br><br>$\tilde{\upsilon}$: 24.5, 58.2, 78.3, 104.6, 123.1, 141.0, 205.8, 210.1, 228.2, 253.1, 253.7, 295.2, 315.8, 343.1, 350.7, 396.1, 421.2, 494.7, 604.1, 686.3, 831.0, 851.3, 922.8, 958.2, 990.3, 1028.8, 1126.9, 1155.0, 1199.8, 1255.2, 1296.5, 1338.4, 1374.3, 1404.5, 1416.0, 1437.5, 1479.4, 1491.4, 1500.3, 1503.8, 1518.2, 1618.1, 1777.9, 3033.3, 3065.2, 3070.2, 3138.5, 3139.7, 3147.3, 3156.3, 3162.1, 3837.1, 3887.4, 3948.8<br><br>B: 1.7800000 0.0014954 1.0813148	C 0.871124 0.351709 -0.325103<br>C 2.356408 0.649293 -0.449713<br>C 0.401336 0.444801 1.128019<br>N 0.720692 -1.064555 -0.842785<br>C 0.041965 1.278366 -1.203827<br>N -0.345174 -1.605835 -0.736296<br>O -1.459308 -1.386320 -0.307029<br>O 1.005962 -0.500079 1.981232<br>H 2.667441 0.577298 -1.490650<br>H 2.949368 -0.066464 0.119610<br>H 2.564929 1.657452 -0.090575<br>H -0.671839 0.283971 1.195149<br>H 0.593617 1.471503 1.459743<br>H 0.188931 2.308506 -0.877122<br>H -1.021849 1.058827 -1.130852<br>H 0.360932 1.197586 -2.242048<br>H -2.814651 0.095162 0.138443<br>H 1.940883 -0.303527 2.071860<br>O -2.988566 1.015657 0.360753<br>H -3.876088 1.033334 0.724108

Table S7 Quantum chemistry results for the $(CH_3)_2C(\dot{N}H)CH_2OH + NO_2$ reaction. Electronic energies of reactants, intermediates and products (/Hartree), and relative energies including Zero Point Energies, $\Delta E_{v=0}$ (/kJ mol^{-1}), of stationary points on the potential energy surface.

	M06-2X/aug-cc-pVTZ		
Species	E_{Elec}	E_{ZPE}	$\Delta E_{v=0}$
$(CH_3)_2C(\dot{N}H)CH_2OH$	-288.337782	0.140666	
NO_2	-205.074622	0.009163	
Sum Reactants	-493.412404	0.149829	0
$O_2NNHC(CH_3)_2CH_2OH$	-493.502624	0.158894	-213.1
SP-18a	-493.420661	0.151139	-18.2
$CH_2{=}C(CH_3)CH_2OH$	-232.421432	0.114002	
HNN(O)OH	-261.028741	0.039836	
Sum products	-493.450174	0.153838	-88.6
SP-18b	-493.413428	0.149801	-2.8
$(CH_3)_2C{=}CHOH$	-232.428751	0.113649	
HNN(O)OH	-261.028741	0.039836	
Sum products	-493.457492	0.153485	-108.8
$ONONHC(CH_3)_2CH_2OH$	-493.466388	0.156291	-124.8
$(CH_3)_2C(NH\dot{O})CH_2OH$	-363.557515	0.146386	
NO	-129.893351	0.004707	
Sum products	-493.450866	0.151093	-97.7

Table S7, continued: Vibrational frequencies (/cm^{-1}), Rotational constants (B /GHZ) and Cartesian Coordinates (/Å) of the species listed above. Results from M06-2X/aug-cc-pVTZ calculations.

$(CH_3)_2(CH_2OH)C\dot{N}H$<br><br>$\tilde{\upsilon}$: 141.3, 214.6, 240.0, 264.1, 287.5, 336.7, 377.9, 402.7, 428.3, 477.5, 568.0, 785.0, 918.1, 931.8, 955.0, 982.7, 1021.2, 1113.3, 1135.6, 1182.3, 1195.6, 1286.7, 1343.4, 1384.0, 1389.8, 1405.1, 1438.0, 1480.5, 1486.9, 1498.3, 1511.1, 1515.1, 3025.9, 3054.2, 3059.9, 3105.9, 3126.2, 3130.4, 3134.3, 3155.8, 3430.7, 3823.8<br><br>B: 4.7912622 2.7527182 2.6614024	C<br>C<br>C<br>C<br>N<br>O<br>H<br>H<br>H<br>H<br>H<br>H<br>H<br>H<br>H<br>H	-1.612958<br>-0.389776<br>-0.521020<br>0.881788<br>-0.242437<br>2.043661<br>-1.563550<br>-1.660565<br>-2.530393<br>0.368307<br>-1.395539<br>-0.635317<br>0.888281<br>0.885224<br>-1.126842<br>1.969963	-0.865260<br>-0.004193<br>1.379283<br>-0.704632<br>0.171942<br>-0.016132<br>-1.814633<br>-1.073182<br>-0.350362<br>1.975388<br>1.904471<br>1.265015<br>-0.749098<br>-1.730713<br>0.541682<br>0.125702	-0.270175<br>0.043587<br>-0.605891<br>-0.445419<br>1.473533<br>-0.051089<br>0.263252<br>-1.340161<br>0.017841<br>-0.411935<br>-0.220080<br>-1.684590<br>-1.535245<br>-0.059721<br>1.832209<br>0.899800
NO_2<br>$\tilde{\upsilon}$: 783.5, 1465.2, 1775.4<br>B: 253.9647127 13.2906276 12.6296837	N<br>O<br>O	0.000000<br>1.090260<br>-1.090260	0.314443<br>-0.137566<br>-0.137572	0.000000<br>0.000000<br>0.000000

$O_2NNHC(CH_3)_2CH_2OH$<br><br>$\tilde{\nu}$: 80.3, 99.1, 156.9, 216.5, 246.6, 266.2, 303.0, 331.2, 355.8, 376.9, 453.5, 510.5, 540.6, 561.8, 602.0, 790.0, 812.7, 833.3, 904.8, 958.3, 967.1, 1020.1, 1039.0, 1118.8, 1151.8, 1189.9, 1238.3, 1266.9, 1281.4, 1389.1, 1404.7, 1423.9, 1437.5, 1439.6, 1460.9, 1485.6, 1488.1, 1499.2, 1514.1, 1516.8, 1697.5, 3040.4, 3060.9, 3068.8, 3108.3, 3130.5, 3141.8, 3155.9, 3164.0, 3596.3, 3848.9<br><br>B: 2.2600000 0.0016253 1.3777237	C -0.803365 -0.480239 0.030027<br>C -1.596812 -1.703354 -0.425712<br>C -1.354187 0.748355 -0.730374<br>N 0.582123 -0.805228 -0.382158<br>C -0.904325 -0.299727 1.539666<br>N 1.636843 -0.001961 -0.090351<br>O 1.426060 1.187684 0.062458<br>O -1.229997 1.977457 -0.071747<br>O 2.725994 -0.529841 -0.049479<br>H -1.187504 -2.621259 -0.000734<br>H -1.589618 -1.786686 -1.512968<br>H -2.626948 -1.609126 -0.088595<br>H -2.425961 0.592455 -0.856338<br>H -0.898314 0.765894 -1.725948<br>H 0.881642 -1.766176 -0.287150<br>H -1.926785 -0.033651 1.805115<br>H -0.257208 0.502356 1.886545<br>H -0.636222 -1.226341 2.048372<br>H -0.290163 2.160261 0.027770
SP-18a<br><br>$\tilde{\nu}$: -1201.0, 45.7, 77.2, 142.9, 202.9, 223.3, 230.2, 273.4, 358.9, 383.8, 441.1, 524.5, 558.7, 606.8, 618.3, 671.5, 739.8, 820.6, 841.1, 882.5, 929.3, 971.7, 1007.6, 1024.3, 1045.3, 1100.8, 1150.8, 1211.0, 1248.4, 1305.1, 1335.6, 1381.2, 1401.3, 1423.6, 1427.3, 1461.6, 1479.3, 1489.4, 1496.7, 1527.1, 1572.1, 1628.1, 3039.9, 3054.7, 3076.4, 3124.0, 3126.7, 3160.2, 3222.1, 3491.7, 3785.9<br><br>B: 2.3200000 0.0039634 1.1699600	C 1.007480 0.594860 0.275296<br>C 0.772696 2.023584 -0.094132<br>C 1.978856 -0.180929 -0.572075<br>N -0.643701 -0.248340 -0.982107<br>C 0.695122 0.134461 1.557657<br>N -1.683985 -0.275174 -0.210337<br>O -1.538908 -0.982344 0.849572<br>O 1.890890 -1.559816 -0.373942<br>O -2.708579 0.357957 -0.405239<br>H -0.153059 2.395400 0.342015<br>H 0.758659 2.176033 -1.171194<br>H 1.595167 2.613546 0.320267<br>H 2.983250 0.131348 -0.265918<br>H 1.853310 0.094057 -1.622926<br>H -0.877441 0.416526 -1.718884<br>H 1.292836 -0.691612 1.928258<br>H -0.401064 -0.499001 1.310965<br>H 0.380493 0.877846 2.281462<br>H 0.989495 -1.807776 -0.620538
CH_2=C(CH_3)CH_2OH<br><br>$\tilde{\nu}$: 119.0, 175.6, 286.6, 302.2, 392.9, 432.5, 545.1, 719.0, 853.1, 947.9, 969.9, 982.3, 1019.8, 1077.4, 1135.9, 1222.2, 1282.2, 1376.4, 1411.0, 1426.3, 1446.0, 1485.0, 1491.4, 1500.7, 1751.0, 3010.2, 3051.8, 3079.3, 3103.1, 3151.0, 3170.3, 3254.2, 3869.7<br><br>B: 8.9500000 0.0093345 3.7196983	C -0.414674 1.456782 -0.038486<br>C -0.494139 0.133585 -0.019860<br>C -1.793327 -0.612642 0.047746<br>C 0.723843 -0.749793 -0.067403<br>O 1.956911 -0.073630 -0.016976<br>H 0.537372 1.963627 -0.117547<br>H -1.305997 2.068641 0.002251<br>H -1.892354 -1.290385 -0.803180<br>H -2.641715 0.067855 0.045635<br>H -1.842308 -1.226337 0.949685<br>H 0.721248 -1.310238 -1.005311<br>H 0.649107 -1.491930 0.737966<br>H 1.989137 0.440216 0.794328
HNN(O)OH	N 0.553212 1.141221 0.000000

$\tilde{\nu}$: 451.6, 616.4, 667.7, 765.5, 832.8, 954.4, 1298.0, 1364.8, 1505.7, 1741.8, 3493.9, 3784.8<br><br>B: 12.8410030 12.0400028 6.2138047	N 0.111702 -0.028679 -0.000001<br>O 0.673398 -1.096555 0.000000<br>O -1.258043 -0.110758 0.000000<br>H 1.568593 1.055573 0.000001<br>H -1.545832 0.815131 0.000001
SP-18b<br><br>$\tilde{\nu}$: -1290.0, 57.7, 78.4, 130.8, 192.2, 200.8, 217.8, 235.0, 250.5, 284.7, 327.0, 388.5, 453.5, 565.2, 609.1, 664.6, 738.4, 820.3, 827.2, 886.1, 888.8, 932.8, 1010.7, 1025.7, 1085.1, 1095.9, 1170.3, 1248.3, 1269.1, 1302.5, 1319.4, 1350.0, 1409.1, 1417.1, 1448.9, 1467.3, 1477.0, 1491.1, 1502.2, 1537.7, 1580.1, 1634.5, 3048.5, 3053.5, 3089.0, 3120.8, 3137.2, 3161.9, 3195.5, 3481.6, 3875.7<br><br>B: 2.3000000 0.0036374 1.2068118	C 0.978039 -0.573054 0.206228<br>C 0.370503 -1.532898 1.178476<br>C 1.163574 0.746517 0.642005<br>N -0.870322 -0.353501 -1.069498<br>C 1.744286 -1.107529 -0.950696<br>N -1.692246 0.242528 -0.271832<br>O -1.303988 1.403159 0.126511<br>O 2.064426 1.526693 -0.083228<br>O -2.730637 -0.241040 0.157655<br>H -0.118794 -2.362704 0.674789<br>H -0.344495 -1.039363 1.836923<br>H 1.175227 -1.940361 1.796791<br>H 1.207406 0.877059 1.725315<br>H -0.039651 1.146101 0.434492<br>H -1.277269 -1.277276 -1.217526<br>H 2.744990 -1.358753 -0.583182<br>H 1.853707 -0.354896 -1.723859<br>H 1.286115 -2.006987 -1.354460<br>H 1.673916 2.385275 -0.263549
$(CH_3)_2C=CHOH$<br><br>$\tilde{\nu}$: 82.1, 207.9, 290.0, 297.6, 344.7, 369.2, 490.5, 579.0, 818.2, 910.0, 979.2, 1016.6, 1053.4, 1101.7, 1182.8, 1221.1, 1313.1, 1407.4, 1418.7, 1429.5, 1470.0, 1488.3, 1498.3, 1507.8, 1782.5, 3041.5, 3047.0, 3088.8, 3097.6, 3120.4, 3136.9, 3214.1, 3880.2<br><br>B: 8.3200000 0.0066030 3.7643843	C 0.396802 -0.032559 -0.000001<br>C -0.754349 -0.696308 0.000005<br>O -2.015569 -0.179191 0.000008<br>C 0.506675 1.463859 -0.000005<br>C 1.698054 -0.779052 -0.000005<br>H -0.783067 -1.777858 0.000008<br>H -1.975748 0.780639 0.000004<br>H 1.059645 1.805770 0.877847<br>H -0.451845 1.983200 0.000005<br>H 1.059625 1.805767 -0.877871<br>H 2.295399 -0.521447 -0.878099<br>H 1.542044 -1.856737 -0.000001<br>H 2.295406 -0.521443 0.878084
$ONONHC(CH_3)_2CH_2OH$<br><br>$\tilde{\nu}$: 57.3, 88.5, 136.5, 219.0, 224.9, 243.8, 280.5, 316.3, 343.0, 367.1, 385.0, 388.2, 472.0, 525.2, 577.5, 713.9, 786.3, 846.2, 883.7, 913.5, 952.3, 963.3, 1001.7, 1036.7, 1092.7, 1131.3, 1189.3, 1239.9, 1287.1, 1305.9, 1389.2, 1405.7, 1418.4, 1438.2, 1482.0, 1487.5, 1495.9, 1502.1, 1514.7, 1537.4, 1771.5, 3042.7, 3061.4, 3066.3, 3108.3, 3130.9, 3136.9, 3149.2, 3152.4, 3475.6, 3869.1<br><br>B: 2.3200000 0.0088683 1.2927419	C -0.809729 -0.478274 0.006536<br>C -1.495476 -1.776371 -0.404076<br>C -1.782809 0.680543 -0.229773<br>N 0.317681 -0.347986 -0.950562<br>C -0.365966 -0.530231 1.464300<br>O 1.115528 0.774973 -0.633038<br>N 2.304133 0.546039 0.058108<br>O -1.349652 1.923072 0.274894<br>O 2.544222 -0.576907 0.265956<br>H -0.809411 -2.620551 -0.317875<br>H -1.847310 -1.719843 -1.433766<br>H -2.344821 -1.968282 0.249882<br>H -2.714548 0.455455 0.290629<br>H -1.992978 0.737361 -1.302538<br>H 0.930328 -1.157036 -0.856235<br>H -1.241032 -0.592140 2.111570

	H 0.179408 0.369599 1.743270<br>H 0.263020 -1.402176 1.645105<br>H -0.532262 2.168137 -0.167282
$(CH_3)_2C(NH\dot{O})CH_2OH$<br><br>$\tilde{\upsilon}$: 78.1, 143.8, 218.5, 233.0, 234.2, 263.5, 326.3, 354.1, 383.8, 404.6, 466.1, 536.1, 603.5, 808.9, 915.9, 926.9, 954.7, 984.7, 1032.0, 1128.1, 1185.9, 1217.3, 1287.9, 1297.4, 1394.4, 1400.9, 1411.0, 1427.3, 1441.4, 1485.8, 1488.7, 1498.6, 1501.3, 1513.4, 1538.0, 3057.0, 3059.1, 3065.7, 3116.4, 3127.4, 3133.2, 3143.8, 3154.3, 3470.2, 3843.0<br><br>B: 2.8700000 0.0007650 2.5942906	C 0.519986 -0.112335 -0.010369<br>C 1.622841 0.340893 -0.964335<br>C 1.059060 -1.007851 1.093975<br>C -0.588092 -0.808966 -0.806216<br>O -1.718973 -1.101419 -0.023854<br>N -0.080691 1.061041 0.637403<br>O -0.974700 1.754232 0.070159<br>H 1.230367 1.072978 -1.669289<br>H 2.010987 -0.512247 -1.522278<br>H 2.448162 0.792483 -0.413367<br>H 0.240672 -1.409580 1.689082<br>H 1.737905 -0.453372 1.745659<br>H 1.619664 -1.834697 0.659695<br>H -0.194094 -1.757212 -1.173516<br>H -0.852993 -0.189469 -1.665739<br>H -2.160657 -0.265518 0.159821<br>H 0.351443 1.436400 1.479346

Table S8 Quantum chemistry results relevant to the atmospheric $(CH_3)_2(NH_2)C\dot{C}H_2$ + O_2 reaction. Electronic energies of reactants, intermediates and products (/Hartree), and relative energies including Zero Point Energies, $\Delta E_{v=0}$ (/kJ mol^{-1}), of stationary points on the potential energy surface.

	M06-2X/aug-cc-pVTZ		
Species	E_{Elec}	E_{ZPE}	$\Delta E_{v=0}$
$\dot{C}H_2(CH_3)C(NH_2)CH_2OH$	-288.335946	0.140329	
2 O_2	-300.649630	0.008010	
Sum reactants	-588.985576	0.148339	0
$\dot{O}OCH_2(CH_3)C(NH_2)CH_2OH$	-438.720445	0.152225	
O_2	-150.324815	0.004005	
Sum intermediates	-589.045260	0.156230	-136.0
SP-26a	-438.676247	0.145683	
O_2	-150.324815	0.004005	
Sum intermediates	-589.001062	0.149688	-37.1
$HOOCH_2(CH_3)C(NH_2)CH_2\dot{O}$	-438.688590	0.149876	
O_2	-150.324815	0.004005	
Sum intermediates	-589.013405	0.153881	-58.5
$HOOCH_2(CH_3)C(NH_2)CHO$	-438.153984	0.140442	
HO_2	-150.908095	0.014578	
Sum products	-589.062079	0.155021	-183.3
SP-28	-438.684995	0.149380	
O_2	-150.324815	0.004005	
Sum intermediates	-589.009810	0.153385	-50.4
$HOOCH_2(CH_3)\dot{C}(NH_2){\bullet}CH_2O$	-438.687520	0.147838	
O_2	-150.324815	0.004005	
Sum intermediates	-589.012335	0.151843	-61.1
$HOOCH_2(CH_3)\dot{C}(NH_2)$	-324.175001	0.116429	
CH_2O	-114.498970	0.027013	
O_2	-150.324815	0.004005	
Sum products	-588.998786	0.143441	-47.5
SP-29a	-438.657307	0.146312	
O_2	-150.324815	0.004005	
Sum intermediates	-588.982122	0.150317	14.3
$(CH_3)(\dot{N}H)(CH_2OH)CCH_2OOH$	-438.695826	0.150312	
O_2	-150.324815	0.004005	
Sum intermediates	-589.020641	0.154317	-76.4
SP-29b	-438.646774	0.145296	
O_2	-150.324815	0.004005	
Sum intermediates	-588.971589	0.149301	39.2
$(\dot{C}H_2)(NH_2)(CH_2OH)CCH_2OOH$	-438.690118	0.148542	
O_2	-150.324815	0.004005	

Sum intermediates	-589.014933	0.152547	-66.0
SP-29c	-438.660285	0.146669	
O_2	-150.324815	0.004005	
Sum intermediates	-588.985100	0.150674	7.4
$(CH_3)(NH_2)(CH_2OH)C\dot{C}HOOH$	-438.699864	0.151063	
O_2	-150.324815	0.004005	
Sum intermediates	-589.024679	0.155068	-85.0
SP-26b	-438.676423	0.146838	
O_2	-150.324815	0.004005	
Sum intermediates	-589.001238	0.150843	-34.6
$(CH_3)(NH_2)(\dot{C}HOH)CCH_2OOH$	-438.711048	0.151445	
O_2	-150.324815	0.004005	
Sum intermediates	-589.035863	0.155450	-113.4
SP-26c	-438.682344	0.146294	
O_2	-150.324815	0.004005	
Sum intermediates	-589.007159	0.150299	-51.5
$(CH_3)(\dot{N}H)(CH_2OH)CCH_2OOH$	-438.695826	0.150312	
O_2	-150.324815	0.004005	
Sum intermediates	-589.020641	0.154317	-76.4
SP-26d	-438.672154	0.145447	
O_2	-150.324815	0.004005	
Sum intermediates	-588.996969	0.149452	-27.0
$(\dot{C}H_2)(NH_2)(CH_2OH)CCH_2OOH$	-438.690305	0.149349	
O_2	-150.324815	0.004005	
Sum intermediates	-589.015120	0.153354	-64.4

Table S8, continued: Vibrational frequencies (/cm^{-1}), Rotational constants (B /GHZ) and Cartesian Coordinates (/Å) of the species listed above. Results from M06-2X/aug-cc-pVTZ calculations.

$(CH_3)(CH_2OH)(NH_2)C\dot{C}H_2$<br><br>$\tilde{\upsilon}$: 143.2, 167.1, 248.2, 255.0, 281.3, 344.5, 366.4, 404.7, 445.0, 513.5, 529.1, 603.8, 791.4, 871.8, 919.6, 937.8, 959.2, 1009.9, 1096.0, 1130.4, 1192.1, 1213.3, 1269.8, 1331.2, 1378.0, 1403.7, 1432.9, 1462.3, 1492.4, 1499.4, 1513.0, 1641.2, 2988.4, 3051.5, 3106.2, 3124.4, 3130.8, 3170.4, 3278.6, 3510.3, 3597.3, 3792.6<br><br>B: 4.7179851 2.7572390 2.7116290	C 1.667865 -0.799616 -0.157911<br>C 0.410506 0.048114 0.036793<br>C 0.443990 0.741319 1.355212<br>C -0.828718 -0.866607 -0.054554<br>N 0.226590 1.063887 -1.013604<br>O -2.025088 -0.141592 0.056045<br>H 1.643675 -1.303347 -1.125725<br>H 1.749514 -1.560085 0.620418<br>H 2.557679 -0.171029 -0.116644<br>H 0.900441 0.253118 2.203262<br>H -0.195786 1.593203 1.528115<br>H -0.807076 -1.600793 0.751208<br>H -0.783502 -1.407559 -1.010756<br>H 0.390992 0.657025 -1.928198<br>H 0.889651 1.820221 -0.894396<br>H -1.892868 0.665514 -0.457667
O_2	O 0.000000 0.000000 0.594925

$\tilde{\upsilon}$: 1754.5, B: 0.0000000 44.6355338 44.6355338	O 0.000000 0.000000 -0.594925
$\dot{O}OCH_2(CH_3)C(NH_2)CH_2OH$<br><br>$\tilde{\upsilon}$: 70.3, 126.1, 172.2, 217.0, 250.1, 267.8, 288.8, 342.0, 366.0, 399.8, 415.3, 484.3, 536.7, 610.3, 799.4, 859.5, 907.1, 942.7, 980.3, 982.8, 1027.7, 1082.4, 1126.5, 1171.1, 1219.0, 1248.7, 1293.7, 1316.1, 1363.3, 1366.8, 1399.1, 1408.8, 1438.9, 1473.8, 1493.2, 1501.2, 1507.1, 1642.8, 3029.3, 3059.8, 3081.2, 3123.1, 3126.8, 3146.1, 3152.9, 3526.1, 3608.9, 3866.2<br><br>B: 2.5300000 0.0028177 1.7691060	C 0.722426 -0.305283 0.057085<br>C 1.073253 1.130818 -0.347573<br>N 0.455856 -0.309099 1.482523<br>C 1.917041 -1.207106 -0.241908<br>C -0.449297 -0.841236 -0.784508<br>O -1.638128 -0.051256 -0.609304<br>O 0.125719 2.089211 0.070636<br>O -2.347849 -0.450628 0.406624<br>H 1.232134 1.173220 -1.431215<br>H 2.006397 1.400447 0.145396<br>H 0.049546 -1.185624 1.785931<br>H -0.188130 0.435098 1.724531<br>H 2.172291 -1.195302 -1.302671<br>H 1.700687 -2.236574 0.046889<br>H 2.775254 -0.868310 0.334950<br>H -0.695457 -1.864922 -0.503828<br>H -0.228930 -0.781594 -1.850769<br>H -0.713253 1.925481 -0.369105
SP-26a<br><br>$\tilde{\upsilon}$: -1960.4, 136.4, 199.2, 222.5, 257.6, 275.7, 300.7, 326.2, 378.6, 411.3, 490.1, 499.0, 570.7, 614.6, 812.0, 867.7, 896.0, 938.3, 943.8, 1000.7, 1040.3, 1064.8, 1079.9, 1109.0, 1195.7, 1201.7, 1256.2, 1281.7, 1301.7, 1353.4, 1364.1, 1410.7, 1414.8, 1422.6, 1467.9, 1494.2, 1501.0, 1537.3, 1645.7, 2993.6, 3043.1, 3055.4, 3060.9, 3116.7, 3128.8, 3144.2, 3519.7, 3601.5<br><br>B: 2.8400000 0.0080554 1.7989371	C 0.840278 -0.062496 0.010859<br>C 0.258789 1.270063 -0.484493<br>N 1.081082 0.082378 1.439961<br>C 2.164401 -0.301371 -0.701770<br>C -0.092932 -1.233594 -0.323405<br>O -1.358558 -1.132921 0.317918<br>O -0.904795 1.649033 0.170159<br>O -2.129922 -0.217196 -0.370606<br>H 0.112425 1.256685 -1.571380<br>H 0.982422 2.063978 -0.251367<br>H 1.460538 -0.772521 1.829960<br>H 0.209583 0.276704 1.920030<br>H 2.029414 -0.367584 -1.781859<br>H 2.619538 -1.231940 -0.358687<br>H 2.851373 0.513998 -0.478772<br>H 0.335255 -2.159426 0.065176<br>H -0.253151 -1.328935 -1.400471<br>H -1.791980 0.745456 0.000725
$HOOCH_2(CH_3)C(NH_2)CH_2\dot{O}$<br><br>$\tilde{\upsilon}$: 81.9, 140.3, 199.5, 215.9, 223.6, 261.2, 274.4, 319.4, 365.6, 404.3, 424.5, 481.8, 526.6, 600.7, 739.5, 807.6, 878.5, 924.2, 925.8, 977.0, 1033.3, 1056.6, 1086.2, 1125.2, 1134.9, 1236.7, 1273.5, 1295.1, 1353.2, 1357.7, 1374.0, 1409.4, 1412.9, 1416.1, 1468.2, 1494.6, 1503.6, 1645.1, 2942.0, 3018.1, 3051.4, 3059.3, 3104.0, 3127.3, 3142.5, 3517.9, 3598.7, 3778.4<br><br>B: 2.7100000 0.0029202 1.7468458	C 0.837049 -0.111003 0.005144<br>C 0.397498 1.259569 -0.520219<br>N 1.084419 0.043129 1.432067<br>C 2.134853 -0.495116 -0.693912<br>C -0.201466 -1.199390 -0.304156<br>O -1.433723 -1.047789 0.370495<br>O -0.632954 1.843553 0.163046<br>O -2.256432 -0.180293 -0.400351<br>H 0.181119 1.241851 -1.597100<br>H 1.238820 1.962502 -0.381784<br>H 1.429018 -0.823174 1.829483<br>H 0.216708 0.269109 1.905453<br>H 2.001899 -0.545465 -1.775485<br>H 2.478723 -1.472267 -0.351126<br>H 2.909461 0.235877 -0.465683<br>H 0.183865 -2.155028 0.061005

	H -0.389612 -1.284799 -1.377390<br>H -2.263669 0.621364 0.141497
HOOCH2(CH3)C(NH2)CHO<br><br>$\tilde{\upsilon}$: 68.8, 98.6, 183.5, 205.1, 244.5, 259.2, 303.6, 324.4, 386.6, 398.9, 442.7, 538.2, 648.5, 702.0, 819.1, 882.8, 936.0, 942.7, 975.4, 1008.8, 1041.1, 1095.6, 1133.5, 1192.6, 1258.3, 1325.4, 1358.3, 1389.6, 1411.0, 1420.3, 1470.8, 1494.4, 1503.3, 1514.6, 1659.1, 1859.5, 2944.7, 3052.8, 3064.4, 3109.3, 3134.7, 3158.2, 3503.1, 3579.7, 3603.5<br><br>B: 3.4700000 0.0024972 1.3445355	C 1.786563 0.033777 -0.558241<br>C 0.434532 0.236214 0.106818<br>C 0.383273 1.645612 0.689007<br>O 2.526008 -0.875048 -0.303686<br>N 0.177281 -0.742974 1.158168<br>C -0.635079 0.084331 -0.992888<br>O -1.925097 0.364231 -0.510497<br>O -2.422082 -0.791843 0.153681<br>H 2.057280 0.788320 -1.322921<br>H -0.609654 1.830922 1.093394<br>H 0.587590 2.390720 -0.079964<br>H 1.121284 1.760740 1.483763<br>H 0.722091 -0.515219 1.982270<br>H 0.504114 -1.656208 0.854799<br>H -0.613207 -0.923360 -1.416806<br>H -0.456940 0.811869 -1.787839<br>H -1.799897 -0.865278 0.901966
HO_2<br>$\tilde{\upsilon}$: 1252.8, 1459.4, 3686.8<br>B: 628.5431720 34.6992321 32.8838526	H -0.880747 -0.865418 0.000000<br>O 0.055047 0.708193 0.000000<br>O 0.055047 -0.600015 0.000000
SP-28<br><br>$\tilde{\upsilon}$: -99.7, 52.6, 182.1, 219.7, 237.0, 260.7, 278.4, 322.7, 378.0, 381.3, 413.6, 459.1, 481.1, 499.0, 612.7, 673.9, 847.2, 864.0, 930.4, 982.3, 1020.5, 1065.0, 1067.2, 1150.1, 1211.1, 1251.4, 1291.7, 1317.8, 1340.9, 1371.0, 1411.5, 1421.5, 1472.3, 1492.6, 1500.7, 1514.2, 1546.9, 1637.7, 2967.4, 3058.0, 3063.2, 3064.3, 3115.2, 3134.4, 3154.3, 3546.3, 3651.3, 3655.7<br><br>B: 2.5200000 0.0056768 1.8641194	C 0.745544 -0.344320 0.050147<br>C 0.466599 1.295047 0.673129<br>N 0.748066 -1.252658 1.109875<br>C 2.078030 -0.244642 -0.661015<br>C -0.368736 -0.671572 -0.934022<br>O -1.617598 -0.953196 -0.352886<br>O -0.033789 1.973802 -0.307177<br>O -2.123603 0.185495 0.342018<br>H 1.492367 1.554825 0.973726<br>H -0.193831 1.094476 1.522810<br>H 1.465299 -1.117618 1.806658<br>H -0.165591 -1.425676 1.508539<br>H 2.064947 0.589798 -1.360267<br>H 2.277336 -1.169760 -1.203358<br>H 2.884603 -0.087704 0.054658<br>H -0.112047 -1.606473 -1.436573<br>H -0.446837 0.130148 -1.673092<br>H -1.831407 0.950707 -0.187312
HOOCH2(CH3)Ċ(NH2)•CH2O<br><br>$\tilde{\upsilon}$: 91.6, 98.9, 142.1, 172.6, 189.7, 213.4, 231.0, 261.7, 297.6, 346.2, 392.6, 412.6, 445.3, 490.1, 535.0, 594.9, 618.9, 854.5, 930.1, 992.5, 1002.3, 1056.3, 1085.0, 1125.3, 1135.3, 1264.5, 1274.4, 1323.5, 1404.2, 1412.2, 1440.7, 1473.5, 1481.1, 1489.4, 1504.0, 1514.3, 1641.8, 1725.2, 2976.1, 3007.7, 3018.0, 3056.5, 3057.1, 3082.5, 3130.0, 3565.6, 3654.1, 3681.3<br><br>B: 2.0600000 0.0082060 1.8863373	C 0.959677 -0.277862 0.015273<br>C -0.279129 1.841213 0.534504<br>N 1.062277 -1.026144 1.160969<br>C 2.198699 0.369064 -0.502773<br>C -0.016803 -0.777583 -1.001032<br>O -1.115107 -1.481593 -0.457699<br>O -1.122580 1.937791 -0.339456<br>O -1.802786 -0.661802 0.482409<br>H 0.666906 2.399713 0.473317<br>H -0.485844 1.322776 1.480395<br>H 1.683983 -0.690647 1.878816<br>H 0.197952 -1.421940 1.502797

	H 1.957264 1.075350 -1.297119<br>H 2.892893 -0.375191 -0.911996<br>H 2.725508 0.909867 0.285787<br>H 0.451562 -1.524237 -1.654314<br>H -0.359039 0.056308 -1.624417<br>H -2.018007 0.146850 -0.017907
$HOOCH_2(CH_3)\dot{C}(NH_2)$<br><br>$\tilde{\nu}$: 59.2, 108.9, 169.1, 172.7, 257.7, 324.5, 359.5, 366.7, 429.3, 586.8, 702.5, 844.2, 920.1, 993.7, 1002.0, 1051.6, 1069.7, 1085.4, 1268.4, 1333.2, 1388.9, 1394.0, 1421.3, 1436.8, 1465.9, 1474.2, 1502.8, 1636.6, 2979.7, 2985.7, 3065.2, 3082.0, 3129.8, 3552.2, 3653.5, 3832.5<br><br>B: 6.1000000 0.0052895 2.2478197	C -0.816672 0.016360 0.042773<br>N -0.942067 1.393398 -0.118084<br>C -2.049819 -0.758875 0.331856<br>C 0.300886 -0.597850 -0.712835<br>O 1.536298 0.085474 -0.515123<br>O 1.899355 -0.059357 0.856628<br>H -1.584674 1.838162 0.519863<br>H -0.055488 1.877922 -0.151095<br>H -1.806201 -1.790852 0.581436<br>H -2.743579 -0.775007 -0.520235<br>H -2.594957 -0.330177 1.176326<br>H 0.175339 -0.503952 -1.802058<br>H 0.406159 -1.655104 -0.459110<br>H 2.706281 -0.581526 0.778657
CH_2O<br>$\tilde{\nu}$: 1213.6, 1273.5, 1539.9, 1869.1, 2945.5, 3015.7<br><br>B: 284.7518606 39.4556659 34.6539588	C 0.000000 0.000000 -0.525505<br>H 0.000000 0.938356 -1.105549<br>H 0.000000 -0.938356 -1.105549<br>O 0.000000 0.000000 0.670516
SP-29a<br><br>$\tilde{\nu}$: -1968.3, 105.0, 172.8, 202.4, 235.2, 267.2, 289.2, 341.0, 379.0, 395.9, 466.7, 574.2, 604.3, 690.8, 713.6, 815.3, 906.8, 920.5, 948.9, 1006.7, 1015.3, 1046.0, 1078.7, 1145.7, 1178.0, 1184.9, 1220.5, 1272.3, 1316.8, 1348.5, 1383.6, 1394.3, 1410.6, 1464.5, 1491.8, 1500.3, 1501.5, 1523.2, 2012.9, 3039.1, 3046.1, 3060.1, 3099.5, 3104.1, 3129.0, 3150.7, 3501.6, 3568.3<br><br>B: 2.7700000 0.0063528 1.8440977	C 0.828835 -0.159998 -0.027476<br>C 0.432729 1.110997 -0.801796<br>N 0.859144 0.390563 1.336985<br>C 2.181871 -0.739346 -0.407810<br>C -0.231788 -1.267989 -0.158636<br>O -1.470424 -0.982509 0.438259<br>O -0.387511 1.802852 0.118488<br>O -2.218570 -0.134566 -0.421169<br>H -0.117169 0.877428 -1.714498<br>H 1.318261 1.704667 -1.047834<br>H 0.443687 -0.243708 2.017672<br>H 0.264823 1.369014 1.169019<br>H 2.155255 -1.116110 -1.431788<br>H 2.449935 -1.565417 0.251792<br>H 2.953249 0.024787 -0.336245<br>H 0.123923 -2.149160 0.383541<br>H -0.371253 -1.529798 -1.210766<br>H -1.892560 0.746157 -0.150101
$(CH_3)(\dot{N}H)(CH_2OH)CCH_2OOH$<br><br>$\tilde{\nu}$: 65.0, 128.7, 201.0, 208.6, 245.2, 264.2, 276.1, 333.1, 361.5, 388.0, 464.1, 498.7, 524.2, 575.7, 599.0, 805.2, 914.2, 923.6, 961.4, 985.0, 1021.1, 1092.2, 1118.6, 1136.3, 1156.1, 1208.9, 1240.4, 1319.3, 1345.0, 1372.6, 1396.4, 1410.4, 1443.2, 1471.8, 1491.6, 1502.8, 1508.8, 1515.2, 3013.0, 3059.8, 3064.6, 3085.9, 3118.5, 3134.6, 3145.1, 3434.4, 3644.5, 3806.0	C 0.705530 -0.346755 0.047600<br>C 1.190402 1.074590 -0.297207<br>N 0.261966 -0.382982 1.424724<br>C 1.859607 -1.332002 -0.157527<br>C -0.467899 -0.753076 -0.848159<br>O -1.596054 0.082820 -0.658971<br>O 0.290469 2.084346 0.083278<br>O -2.354414 -0.405453 0.440959<br>H 1.410324 1.110271 -1.371726<br>H 2.121687 1.269425 0.238859

B: 2.5500000 0.0071887 1.7639105	H -1.722840 -0.326175 1.179506<br>H 1.014661 -0.011354 2.010125<br>H 2.185616 -1.313640 -1.198469<br>H 1.546671 -2.345311 0.090175<br>H 2.707683 -1.065467 0.473043<br>H -0.758699 -1.789409 -0.665188<br>H -0.193391 -0.635320 -1.898637<br>H -0.591324 1.837602 -0.221117
SP-29b<br><br>$\tilde{\nu}$: -1952.8, 39.0, 132.1, 169.5, 202.1, 211.9, 255.3, 299.9, 359.8, 400.8, 449.1, 468.1, 556.2, 626.5, 641.3, 802.6, 885.1, 920.6, 936.3, 970.9, 996.3, 1041.6, 1061.1, 1096.2, 1113.3, 1181.3, 1199.5, 1234.0, 1278.0, 1308.2, 1335.9, 1370.1, 1397.4, 1414.2, 1453.3, 1465.0, 1518.2, 1649.7, 1769.5, 3053.9, 3056.1, 3105.1, 3111.5, 3134.9, 3193.9, 3503.5, 3583.1, 3825.8<br><br>B: 2.7700000 0.0010927 1.6275016	C 0.541173 0.458644 -0.073501<br>C 0.782908 -0.851998 -0.840369<br>N 0.398529 1.595868 -0.968304<br>C 1.836182 0.514406 0.730538<br>C -0.623717 0.332401 0.914430<br>O -1.896970 0.440874 0.302152<br>O 1.424804 -1.686053 0.105625<br>O -2.232349 -0.814712 -0.280331<br>H 1.429965 -0.639442 -1.695415<br>H -0.128828 -1.344044 -1.176008<br>H 0.531615 2.464575 -0.462184<br>H -0.544816 1.617875 -1.342575<br>H 1.960152 -0.765225 0.783692<br>H 1.800242 0.924147 1.737185<br>H 2.695469 0.841943 0.151282<br>H -0.600589 1.183841 1.600092<br>H -0.559315 -0.595834 1.486586<br>H -2.956742 -1.100506 0.289315
$(\dot{C}H_2)(NH_2)(CH_2OH)CCH_2OOH$<br><br>$\tilde{\nu}$: 23.1, 69.7, 100.1, 179.2, 216.2, 221.3, 264.1, 281.0, 317.6, 342.6, 361.4, 397.8, 457.5, 475.9, 563.3, 607.0, 792.2, 878.1, 906.5, 912.5, 962.9, 986.9, 1072.4, 1110.8, 1124.5, 1170.5, 1220.2, 1248.6, 1310.0, 1339.7, 1372.9, 1397.7, 1410.6, 1414.6, 1457.1, 1461.7, 1511.4, 1650.2, 3057.2, 3064.2, 3113.4, 3144.0, 3166.6, 3275.8, 3509.4, 3591.6, 3820.9, 3869.6<br><br>B: 2.4700000 0.0058152 1.7837287	C -0.706305 -0.412345 -0.016682<br>C -0.674364 0.931093 -0.778657<br>N -0.760248 -1.453747 -1.040481<br>C -1.940498 -0.478064 0.815627<br>C 0.509783 -0.555107 0.910782<br>O 1.730062 -0.751601 0.218897<br>O -0.596802 2.047350 0.082624<br>O 2.204091 0.507354 -0.244800<br>H -1.562140 0.969860 -1.414192<br>H 0.208332 0.963293 -1.414245<br>H -0.925872 -2.358303 -0.614268<br>H 0.135467 -1.506395 -1.514388<br>H -1.444103 2.149625 0.523669<br>H -1.930185 -0.206769 1.861090<br>H -2.881523 -0.712285 0.339789<br>H 0.398647 -1.468594 1.500335<br>H 0.593745 0.304048 1.579804<br>H 2.898868 0.703469 0.395595
SP-29c<br><br>$\tilde{\nu}$: -1535.5, 123.3, 146.5, 199.5, 260.6, 281.5, 315.6, 337.7, 364.9, 415.9, 432.7, 451.4, 595.5, 614.4, 673.5, 780.4, 837.3, 922.4, 961.3, 971.5, 1006.7, 1025.4, 1071.6, 1084.7, 1168.3, 1196.2, 1202.7, 1257.8, 1296.0, 1337.1, 1348.7, 1398.3, 1411.8, 1480.9, 1494.9, 1502.2, 1528.6, 1648.8, 1808.9, 3041.2, 3056.7, 3090.5, 3127.1, 3128.5, 3150.2, 3535.1, 3625.7, 3669.5	C -0.760493 -0.245706 -0.103783<br>C -0.486682 1.125597 -0.787058<br>N -0.714339 -1.323752 -1.050649<br>C -2.112353 -0.222205 0.593767<br>C 0.324874 -0.275356 1.009311<br>O 1.572693 -0.764414 0.707461<br>O 0.171809 1.925143 0.156067<br>O 2.040210 -0.191331 -0.503833<br>H -1.434173 1.593709 -1.068419

B: 2.6300000 0.0050087 1.8674437	H 0.117362 0.969505 -1.683298<br>H -1.085406 -2.186759 -0.676342<br>H 0.226229 -1.480918 -1.391485<br>H -2.176111 0.631293 1.269262<br>H -2.265402 -1.134228 1.174011<br>H -2.904438 -0.143734 -0.148819<br>H 0.043663 -0.753182 1.947350<br>H 0.383679 0.957592 1.091308<br>H 2.025209 0.763819 -0.310004
$(CH_3)(NH_2)(CH_2OH)C\dot{C}HOOH$<br><br>$\tilde{\upsilon}$: 76.6, 150.7, 181.0, 213.1, 258.3, 265.2, 314.7, 338.0, 359.2, 386.6, 416.8, 423.4, 508.7, 560.9, 603.0, 674.2, 827.2, 887.4, 922.6, 962.4, 988.5, 1001.9, 1044.6, 1115.1, 1173.2, 1206.0, 1233.4, 1271.6, 1337.0, 1346.7, 1408.4, 1412.0, 1436.7, 1488.3, 1490.6, 1506.5, 1528.2, 1649.6, 3057.7, 3066.0, 3124.0, 3151.1, 3154.6, 3193.8, 3508.1, 3591.8, 3643.0, 3850.9<br><br>B: 2.4800000 0.0028149 1.8658362	C 0.742394 -0.222100 0.066524<br>C 0.434279 1.186071 0.633941<br>N 0.862587 -1.107167 1.231006<br>C 2.087777 -0.194901 -0.656620<br>C -0.282147 -0.683460 -0.936591<br>O -1.558387 -0.990103 -0.592087<br>O -0.364079 1.992384 -0.221341<br>O -2.040520 -0.143067 0.442436<br>H 1.376779 1.688073 0.864527<br>H -0.128138 1.085578 1.557871<br>H 1.195845 -2.021364 0.946532<br>H -0.051004 -1.236303 1.653818<br>H 2.078941 0.529834 -1.472583<br>H 2.323873 -1.172235 -1.079994<br>H 2.870893 0.076076 0.048783<br>H 0.013705 -1.314595 -1.765343<br>H 0.007910 1.980727 -1.108533<br>H -1.916845 0.747007 0.062289
SP-26b<br><br>$\tilde{\upsilon}$: -1892.8, 67.0, 210.4, 237.9, 276.0, 286.9, 331.7, 358.6, 392.3, 422.9, 458.2, 542.2, 550.5, 604.9, 669.9, 812.5, 836.3, 934.0, 960.4, 963.3, 993.4, 1011.1, 1081.0, 1114.6, 1158.2, 1196.3, 1219.9, 1244.8, 1297.8, 1298.7, 1330.5, 1389.9, 1411.5, 1431.9, 1490.0, 1498.0, 1504.6, 1634.2, 1651.1, 3059.3, 3065.3, 3129.7, 3132.3, 3151.4, 3154.7, 3518.5, 3607.0, 3762.8<br><br>B: 2.5700000 0.0098401 1.8995177	C 0.773873 -0.163954 0.017968<br>C 0.020521 0.854934 -0.827196<br>N 1.840400 0.547023 0.722255<br>C 1.406737 -1.194280 -0.908460<br>C -0.254341 -0.868571 0.964619<br>O -1.577535 -0.406779 0.731355<br>O -0.457548 1.954162 -0.159967<br>O -1.867468 -0.651442 -0.605666<br>H -1.029440 0.118680 -1.114085<br>H 0.524276 1.152375 -1.742990<br>H 2.328141 -0.069321 1.361345<br>H 1.469693 1.327351 1.251103<br>H 0.640203 -1.706891 -1.489776<br>H 1.944480 -1.943068 -0.324326<br>H 2.115596 -0.710749 -1.578041<br>H -0.056297 -0.626467 2.008412<br>H -0.228799 -1.951514 0.825904<br>H -1.050983 1.644152 0.539320
$(CH_3)(NH_2)(\dot{C}HOH)CCH_2OOH$<br><br>$\tilde{\upsilon}$: 104.0, 131.7, 165.2, 234.5, 249.6, 254.5, 319.6, 342.1, 375.8, 405.2, 421.3, 483.4, 549.0, 645.6, 688.2, 755.6, 808.1, 899.5, 917.5, 938.1, 985.5, 1020.6, 1048.6, 1126.1, 1185.9, 1231.6, 1240.8, 1290.9, 1332.1, 1359.9, 1389.0, 1404.3, 1439.1, 1456.7, 1471.5, 1494.8, 1500.4, 1644.7, 3051.5,	C 0.493342 -0.420178 -0.041551<br>C 0.615514 1.013895 -0.491750<br>N 0.792681 -0.423312 1.395444<br>C 1.529961 -1.292509 -0.757133<br>C -0.904306 -0.969596 -0.341353<br>O -1.914204 -0.329117 0.412110<br>O 1.639454 1.721039 0.031730<br>O -2.330364 0.839813 -0.277681

3058.6, 3111.8, 3126.5, 3148.6, 3184.9, 3509.3, 3600.8, 3672.3, 3701.6<br><br>B: 2.7000000 0.0098885 1.5755321	H 0.369549 1.284167 -1.511388<br>H 1.051416 -1.349298 1.712103<br>H -0.030981 -0.135518 1.914871<br>H 1.354973 -1.288913 -1.832329<br>H 1.477258 -2.324895 -0.403510<br>H 2.531696 -0.908841 -0.568524<br>H -0.948368 -2.017076 -0.033222<br>H -1.144020 -0.898463 -1.404782<br>H 1.853550 1.284203 0.874481<br>H -1.629988 1.474264 -0.054364
SP-26c<br><br>$\tilde{\nu}$: -1884.9, 80.4, 151.3, 217.2, 249.9, 294.2, 336.0, 360.2, 441.1, 455.2, 488.4, 503.5, 572.8, 608.6, 743.9, 802.7, 902.3, 940.9, 976.8, 986.1, 1033.1, 1063.2, 1116.3, 1133.1, 1143.6, 1175.3, 1243.3, 1253.7, 1309.6, 1348.7, 1368.7, 1398.9, 1403.4, 1441.1, 1475.4, 1491.6, 1503.5, 1516.6, 1714.9, 3027.8, 3063.7, 3068.0, 3096.6, 3139.8, 3143.5, 3158.2, 3454.5, 3818.2<br><br>B: 2.5100000 0.0072306 1.9291553	C 0.685980 -0.309695 0.073591<br>C 1.129331 1.113280 -0.293458<br>N 0.105518 -0.376876 1.394146<br>C 1.868292 -1.277785 -0.040149<br>C -0.405691 -0.814687 -0.915425<br>O -1.576094 -0.063113 -0.734326<br>O 0.176737 2.092219 0.049195<br>O -2.093346 -0.396666 0.511592<br>H 1.381112 1.149978 -1.359620<br>H 2.032507 1.358556 0.268003<br>H -1.136111 -0.530212 1.165572<br>H 0.150379 0.553853 1.815062<br>H 2.264692 -1.272004 -1.056885<br>H 1.554289 -2.287639 0.216007<br>H 2.659474 -0.979459 0.645236<br>H -0.611029 -1.870667 -0.726832<br>H -0.087935 -0.658691 -1.945964<br>H -0.671856 1.848225 -0.338635
$(CH_3)(\dot{N}H)(CH_2OH)CCH_2OOH$<br><br>$\tilde{\nu}$: 104.0, 131.7, 165.2, 234.5, 249.6, 254.5, 319.6, 342.1, 375.8, 405.2, 421.3, 483.4, 549.0, 645.6, 688.2, 755.6, 808.1, 899.5, 917.5, 938.1, 985.5, 1020.6, 1048.6, 1126.1, 1185.9, 1231.6, 1240.8, 1290.9, 1332.1, 1359.9, 1389.0, 1404.3, 1439.1, 1456.7, 1471.5, 1494.8, 1500.4, 1644.7, 3051.5, 3058.6, 3111.8, 3126.5, 3148.6, 3184.9, 3509.3, 3600.8, 3672.3, 3701.6<br><br>B: 2.7000000 0.0098885 1.5755321	C 0.493342 -0.420178 -0.041551<br>C 0.615514 1.013895 -0.491750<br>N 0.792681 -0.423312 1.395444<br>C 1.529961 -1.292509 -0.757133<br>C -0.904306 -0.969596 -0.341353<br>O -1.914204 -0.329117 0.412110<br>O 1.639454 1.721039 0.031730<br>O -2.330364 0.839813 -0.277681<br>H 0.369549 1.284167 -1.511388<br>H 1.051416 -1.349298 1.712103<br>H -0.030981 -0.135518 1.914871<br>H 1.354973 -1.288913 -1.832329<br>H 1.477258 -2.324895 -0.403510<br>H 2.531696 -0.908841 -0.568524<br>H -0.948368 -2.017076 -0.033222<br>H -1.144020 -0.898463 -1.404782<br>H 1.853550 1.284203 0.874481<br>H -1.629988 1.474264 -0.054364
SP-26d<br><br>$\tilde{\nu}$: -1846.3, 80.7, 153.8, 215.0, 234.8, 286.3, 306.6, 321.9, 351.6, 403.5, 454.6, 504.2, 546.2, 565.8, 666.4, 792.0, 867.5, 927.5, 945.5, 965.9, 1007.6, 1033.8, 1100.4, 1119.4, 1121.1, 1133.7, 1156.2, 1188.7, 1270.0, 1283.3, 1303.1, 1362.7, 1372.4, 1456.9, 1459.2, 1477.6, 1511.5, 1582.3, 1633.6,	C 0.332018 0.512328 -0.009676<br>C 1.179731 -0.382791 0.883633<br>N 1.233530 1.390691 -0.755198<br>C -0.614343 1.316022 0.840991<br>C -0.501432 -0.376261 -0.975509<br>O -1.452566 -1.118681 -0.249864<br>O 2.052659 -1.128122 0.051343

3041.2, 3058.6, 3089.9, 3114.9, 3149.5, 3204.3, 3514.6, 3601.5, 3906.1<br><br>B: 2.9700000 0.0078446 1.5869109	O -2.419828 -0.232513 0.204534<br>H 0.520408 -1.042620 1.454812<br>H 1.745189 0.254891 1.568131<br>H 0.718098 1.972412 -1.404982<br>H 1.890897 0.828993 -1.285493<br>H -1.757816 0.568338 0.731926<br>H -0.926405 2.265296 0.415647<br>H -0.411591 1.354698 1.907205<br>H 0.145429 -1.101965 -1.465825<br>H -1.004830 0.254657 -1.712671<br>H 2.627953 -1.670797 0.592897
$(\dot{C}H_2)(NH_2)(CH_2OH)CCH_2OOH$<br><br>$\tilde{\upsilon}$: 69.9, 133.3, 182.3, 201.1, 222.1, 229.3, 291.9, 298.3, 310.3, 332.7, 372.7, 407.6, 462.1, 545.9, 570.7, 654.8, 790.6, 855.4, 918.2, 933.2, 973.7, 994.2, 1108.0, 1113.4, 1119.5, 1155.3, 1190.0, 1274.2, 1289.7, 1319.8, 1369.1, 1389.3, 1411.0, 1454.4, 1460.0, 1474.9, 1509.3, 1629.8, 3041.6, 3060.6, 3089.3, 3130.3, 3157.0, 3262.5, 3509.9, 3600.4, 3779.3, 3907.8<br><br>B: 2.8600000 0.0007934 1.5263994	C -0.416803 0.534875 0.000916<br>C -1.147673 -0.466220 -0.886812<br>N -1.435411 1.244047 0.787609<br>C 0.290132 1.530873 -0.859213<br>C 0.556667 -0.208683 0.929746<br>O 1.509672 -1.010025 0.257748<br>O -1.889122 -1.333243 -0.044922<br>O 2.587793 -0.186433 -0.164470<br>H -0.415063 -1.024360 -1.475914<br>H -1.811683 0.088351 -1.555603<br>H -1.002330 1.867636 1.458093<br>H -2.005233 0.573376 1.292540<br>H 2.319412 0.065314 -1.058917<br>H 1.062066 2.152545 -0.428207<br>H -0.171610 1.857730 -1.780415<br>H -0.010266 -0.915033 1.536964<br>H 1.072785 0.500643 1.581829<br>H -2.350875 -1.981995 -0.578298

Table S9 Quantum chemistry results for reactions relevant to the atmospheric fate of the $\dot{O}CH_2C(CH_3)(NH_2)CH_2OH$ radical. Electronic energies of reactants, intermediates and products (/Hartree), and relative energies including Zero Point Energies, $\Delta E_{v=0}$ (/kJ mol^{-1}), of stationary points on the potential energy surface.

	M06-2X/aug-cc-pVTZ		
Species	E_{Elec}	E_{ZPE}	$\Delta E_{v=0}$
$\dot{O}OCH_2C(CH_3)(NH_2)CH_2OH$	-438.720445	0.152225	
NO	-129.893351	0.004707	
O_2	-150.324815	0.004005	
Sum reactants	-718.938612	0.160937	49.6
$\dot{O}CH_2C(CH_3)(NH_2)CH_2OH$	-363.556369	0.146068	
NO_2	-205.074622	0.009163	
O_2	-150.324815	0.004005	
Sum intermediates	-718.955806	0.159236	0
SP-35	-363.543570	0.143488	
NO_2	-205.074622	0.009163	
O_2	-150.324815	0.004005	
Sum intermediates	-718.943007	0.156656	26.8
$\dot{C}(CH_3)(NH_2)CH_2OH{\bullet}CH_2O$	-363.551665	0.142611	
NO_2	-205.074622	0.009163	
O_2	-150.324815	0.004005	
Sum intermediates	-718.951102	0.155780	3.3
$\dot{C}(CH_3)(NH_2)CH_2OH$	-249.040526	0.113072	
CH_2O	-114.498984	0.027101	
NO_2	-205.074622	0.009163	
O_2	-150.324815	0.004005	
Sum intermediates	-718.938947	0.153341	28.8
$CHOC(CH_3)(NH_2)CH_2OH$	-363.022105	0.136428	
NO_2	-205.074622	0.009163	
HO_2	-150.908108	0.014608	
Sum products	-719.004835	0.160200	-126.2

Table S9, continued: Vibrational frequencies (/cm^{-1}), Rotational constants (B /GHZ) and Cartesian Coordinates (/Å) of the species listed above. Results from M06-2X/aug-cc-pVTZ calculations.

$\dot{O}OCH_2C(CH_3)(NH_2)CH_2OH$<br><br>$\tilde{\upsilon}$: 70.3, 126.1, 172.2, 217.0, 250.1, 267.8, 288.8, 342.0, 366.0, 399.8, 415.3, 484.3, 536.7, 610.3, 799.4, 859.5, 907.1, 942.7, 980.3, 982.8, 1027.7, 1082.4, 1126.5, 1171.1, 1219.0, 1248.7, 1293.7, 1316.1, 1363.3, 1366.8, 1399.1, 1408.8, 1438.9, 1473.8, 1493.2, 1501.2, 1507.1, 1642.8, 3029.3, 3059.8, 3081.2, 3123.1, 3126.8, 3146.1, 3152.9, 3526.1, 3608.9, 3866.2<br><br>B: 2.5300000 0.0028177 1.7691060	C	0.722426	-0.305283	0.057085
	C	1.073253	1.130818	-0.347573
	N	0.455856	-0.309099	1.482523
	C	1.917041	-1.207106	-0.241908
	C	-0.449297	-0.841236	-0.784508
	O	-1.638128	-0.051256	-0.609304
	O	0.125719	2.089211	0.070636
	O	-2.347849	-0.450628	0.406624
	H	1.232134	1.173220	-1.431215
	H	2.006397	1.400447	0.145396
	H	0.049546	-1.185624	1.785931
	H	-0.188130	0.435098	1.724531

	H 2.172291 -1.195302 -1.302671<br>H 1.700687 -2.236574 0.046889<br>H 2.775254 -0.868310 0.334950<br>H -0.695457 -1.864922 -0.503828<br>H -0.228930 -0.781594 -1.850769<br>H -0.713253 1.925481 -0.369105
NO<br>$\tilde{\nu}$: 2066.0, B: 0.0000000 52.3561936 52.3561936	N 0.000000 0.000000 -0.606410<br>O 0.000000 0.000000 0.530609
O_2<br>$\tilde{\nu}$: 1754.5, B: 0.0000000 44.6355338 44.6355338	O 0.000000 0.000000 0.594925<br>O 0.000000 0.000000 -0.594925
$\dot{O}CH_2C(CH_3)(NH_2)CH_2OH$<br><br>$\tilde{\nu}$: 111.3, 181.9, 225.4, 241.5, 253.9, 279.0, 329.6, 369.4, 413.2, 508.7, 543.6, 559.8, 735.9, 790.1, 876.0, 929.0, 952.7, 994.2, 1079.9, 1082.9, 1111.9, 1126.6, 1191.4, 1247.1, 1261.6, 1339.6, 1369.9, 1371.3, 1388.4, 1404.7, 1442.7, 1492.5, 1501.5, 1515.0, 1647.1, 2940.4, 2978.8, 3007.7, 3050.6, 3118.2, 3121.3, 3133.2, 3508.3, 3586.8, 3801.8<br><br>B: 2.9800000 0.0014993 2.3812137	C 0.076422 0.440427 -0.011216<br>C 0.994191 1.300009 0.849959<br>C -0.969034 -0.253363 0.862921<br>C 0.899733 -0.618585 -0.752436<br>N -0.656367 1.196620 -1.032612<br>O -1.871452 -1.013665 0.095760<br>O 1.669040 -1.400627 0.052627<br>H 1.516991 0.688642 1.586197<br>H 1.739013 1.801993 0.229820<br>H 0.419167 2.062435 1.377952<br>H -1.497810 0.508909 1.450115<br>H -0.471143 -0.931193 1.556118<br>H 0.210519 -1.315187 -1.263696<br>H 1.512111 -0.166798 -1.549677<br>H -1.090817 2.013741 -0.617244<br>H -0.031252 1.534490 -1.755661<br>H -2.100787 -0.469960 -0.668105
NO_2<br>$\tilde{\nu}$: 783.5, 1465.2, 1775.4<br>B: 253.9647127 13.2906276 12.6296837	N 0.000000 0.314443 0.000000<br>O 1.090260 -0.137566 0.000000<br>O -1.090260 -0.137572 0.000000
SP-35<br><br>$\tilde{\nu}$: -206.5, 63.9, 89.3, 167.9, 174.8, 240.9, 279.8, 286.2, 319.4, 377.8, 406.8, 507.0, 554.6, 680.2, 777.9, 834.9, 937.1, 994.5, 1029.9, 1067.6, 1127.0, 1138.9, 1224.0, 1245.9, 1329.4, 1382.1, 1388.6, 1410.2, 1439.6, 1471.7, 1486.3, 1491.9, 1499.8, 1637.5, 1643.3, 2905.0, 2964.2, 2985.6, 3036.3, 3103.3, 3110.6, 3136.6, 3525.6, 3636.1, 3873.7<br><br>B: 2.5900000 0.0050387 2.4243238	C 0.116600 0.622677 0.079170<br>C -0.232098 1.998362 -0.390632<br>C 1.127544 -0.127610 -0.762837<br>C -1.669577 -0.431099 -0.343997<br>N 0.288258 0.457240 1.439042<br>O 1.716457 -1.215926 -0.096082<br>O -1.479561 -1.582469 0.045855<br>H -0.453524 1.992368 -1.457808<br>H -1.100320 2.392818 0.138382<br>H 0.605515 2.682927 -0.224734<br>H 1.893555 0.589285 -1.092305<br>H 0.648545 -0.525968 -1.656910<br>H -2.247092 0.285601 0.267860<br>H -1.618977 -0.177264 -1.418265<br>H -0.364408 0.969248 2.014873<br>H 0.338548 -0.519531 1.706699<br>H 2.330368 -0.876991 0.560499
$\dot{C}(CH_3)(NH_2)CH_2OH \bullet CH_2O$	C 0.366710 0.601567 0.095068<br>C -0.277915 1.806377 -0.490981

$\tilde{\nu}$: 65.7, 68.5, 78.5, 127.6, 153.5, 196.6, 236.5, 256.7, 276.4, 370.4, 376.5, 384.8, 441.6, 533.8, 752.7, 830.8, 978.5, 1003.8, 1051.1, 1075.0, 1096.7, 1145.7, 1217.2, 1254.9, 1262.8, 1362.5, 1397.3, 1427.9, 1459.6, 1478.0, 1502.3, 1505.8, 1528.4, 1644.7, 1785.4, 2950.4, 2955.2, 3007.0, 3031.8, 3055.4, 3074.0, 3131.8, 3532.8, 3635.7, 3896.7<br><br>B: 3.0800000 0.0051321 1.7501574	C 1.641303 0.086333 -0.469381<br>C -1.583798 -1.005602 -0.598182<br>N 0.180982 0.385291 1.444505<br>O 1.810679 -1.263027 -0.044829<br>O -2.438590 -0.599616 0.152452<br>H -0.180294 1.812598 -1.576102<br>H -1.341806 1.825459 -0.241112<br>H 0.166794 2.731659 -0.104561<br>H 2.493325 0.689769 -0.116600<br>H 1.609092 0.155965 -1.560443<br>H -1.499341 -0.632037 -1.632779<br>H -0.891144 -1.813180 -0.317172<br>H -0.773402 0.512215 1.757002<br>H 0.569649 -0.488964 1.770964<br>H 2.725738 -1.521430 -0.170862
$\dot{C}(CH_3)(NH_2)CH_2OH$<br><br>$\tilde{\nu}$: 111.1, 170.1, 236.4, 256.6, 355.1, 365.5, 395.3, 524.1, 711.2, 829.9, 975.6, 994.5, 1046.0, 1067.0, 1095.7, 1220.3, 1253.0, 1356.0, 1386.3, 1427.6, 1458.6, 1476.2, 1501.1, 1504.8, 1631.2, 2928.0, 2981.0, 3056.1, 3073.2, 3131.1, 3556.9, 3659.0, 3898.3<br><br>B: 8.6800000 0.0079432 3.6653279	C 0.472416 0.028879 -0.152954<br>C 1.787180 -0.661427 -0.079597<br>H 1.724328 -1.660719 -0.508771<br>H 2.553358 -0.108323 -0.626877<br>H 2.143135 -0.762941 0.955557<br>C -0.756667 -0.714609 0.221810<br>H -0.830994 -0.813616 1.318589<br>H -0.718545 -1.720852 -0.204916<br>N 0.448502 1.383407 0.172654<br>H -0.445793 1.817803 -0.007435<br>H 1.212821 1.923718 -0.203255<br>O -1.890220 0.004016 -0.258160<br>H -2.673637 -0.308107 0.198254
CH_2O<br>$\tilde{\nu}$: 1213.6, 1273.5, 1539.9, 1869.1, 2945.5, 3015.7<br><br>B: 284.7518606 39.4556659 34.6539588	C 0.000000 0.000000 -0.525505<br>H 0.000000 0.938356 -1.105549<br>H 0.000000 -0.938356 -1.105549<br>O 0.000000 0.000000 0.670516
$CHOC(CH_3)(NH_2)CH_2OH$<br><br>$\tilde{\nu}$: 101.0, 133.1, 232.7, 240.1, 256.1, 319.0, 325.0, 376.6, 399.6, 543.3, 565.2, 642.6, 809.9, 872.8, 901.9, 937.3, 970.8, 1042.0, 1089.5, 1135.9, 1178.5, 1220.1, 1289.0, 1337.8, 1381.4, 1399.4, 1417.3, 1438.6, 1496.9, 1499.4, 1515.7, 1630.7, 1864.1, 2962.5, 3017.3, 3055.0, 3094.5, 3126.6, 3139.9, 3525.3, 3617.5, 3783.0<br><br>B: 3.0800000 0.0079901 2.4112749	C -0.091632 0.409936 0.041384<br>C -0.693661 1.757877 -0.367940<br>H -0.674138 1.891372 -1.450802<br>H -1.724922 1.834772 -0.024976<br>H -0.114451 2.566167 0.080691<br>C 1.357880 0.312913 -0.443654<br>H 1.922230 1.151074 -0.016853<br>H 1.402119 0.396381 -1.531416<br>C -0.892955 -0.669434 -0.661500<br>H -0.743187 -0.716309 -1.756641<br>N -0.117192 0.149856 1.471582<br>H -0.083041 1.005592 2.008731<br>H -0.955298 -0.363925 1.719745<br>O 1.918285 -0.923045 -0.088853<br>H 1.659666 -1.075174 0.830166<br>O -1.661588 -1.402793 -0.107330
HO_2<br>$\tilde{\nu}$: 1252.8, 1459.4, 3686.8<br>B: 628.5431720 34.6992321 32.8838526	H -0.880747 -0.865418 0.000000<br>O 0.055047 0.708193 0.000000<br>O 0.055047 -0.600015 0.000000

Table S10 Quantum chemistry results for reactions relevant to the Ċ(CH_3)(NH_2)CH_2OH + O_2 reaction. Electronic energies of reactants, intermediates and products (/Hartree), and relative energies including Zero Point Energies, $\Delta E_{v=0}$ (/kJ mol^{-1}), of stationary points on the potential energy surface.

	M06-2X/aug-cc-pVTZ		
Species	E_{Elec}	E_{ZPE}	$\Delta E_{v=0}$
Ċ(CH_3)(NH_2)CH_2OH	-249.040526	0.113072	
O_2	-150.324815	0.004005	
NO	-129.893351	0.004707	
Sum reactants	-529.258693	0.121784	0
ȮOC(CH_3)(NH_2)CH_2OH	-399.427915	0.123345	
NO	-129.893351	0.004707	
Sum intermediates	-529.321267	0.128052	-147.8
SP-37a	-399.389290	0.117676	
NO	-129.893351	0.004707	
Sum intermediates	-529.282641	0.122382	-61.3
HN=C(CH_3)CH_2OH•HO_2	-399.406209	0.119889	
NO	-129.893351	0.004707	
Sum intermediates	-529.299560	0.124596	-99.9
HN=C(CH_3)CH_2OH	-248.482795	0.102996	
HO_2	-150.908108	0.014608	
NO	-129.893351	0.004707	
Sum products	-529.284254	0.122310	-65.7
SP-37b	-399.375767	0.116885	
NO	-129.893351	0.004707	
Sum intermediates	-529.269118	0.121592	-27.9
CH_2=C(NH_2)CH_2OH•HO_2	-399.394099	0.120107	
NO	-129.893351	0.004707	
Sum intermediates	-529.287451	0.124814	-67.5
CH_2=C(NH_2)CH_2OH	-248.473416	0.102966	
HO_2	-150.908108	0.014608	
NO	-129.893351	0.004707	
Sum intermediates	-529.274875	0.122280	-41.2
SP-37c	-399.379651	0.118330	
NO	-129.893351	0.004707	
Sum intermediates	-529.273002	0.123037	-34.3
CH_3(NH_2)C=CHOH•HO_2	-399.405355	0.120333	
NO	-129.893351	0.004707	
Sum intermediates	-529.298707	0.125040	-96.5
CH_3(NH_2)C=CHOH	-248.478596	0.103258	
HO_2	-150.908108	0.014608	
NO	-129.893351	0.004707	
Sum products	-529.280055	0.122572	-54.0

$\dot{O}C(CH_3)(NH_2)CH_2OH$	-324.259599	0.118579	
NO_2	-205.074622	0.009163	
Sum products	-529.334221	0.127742	-182.7

Table S10, continued: Vibrational frequencies (/cm^{-1}), Rotational constants (B /GHZ) and Cartesian Coordinates (/Å) of the species listed above. Results from M06-2X/aug-cc-pVTZ calculations.

$\dot{C}(CH_3)(NH_2)CH_2OH$<br><br>$\tilde{\nu}$: 111.1, 170.1, 236.4, 256.6, 355.1, 365.5, 395.3, 524.1, 711.2, 829.9, 975.6, 994.5, 1046.0, 1067.0, 1095.7, 1220.3, 1253.0, 1356.0, 1386.3, 1427.6, 1458.6, 1476.2, 1501.1, 1504.8, 1631.2, 2928.0, 2981.0, 3056.1, 3073.2, 3131.1, 3556.9, 3659.0, 3898.3<br><br>B: 8.6800000 0.0079432 3.6653279	C 0.472416 0.028879 -0.152954<br>C 1.787180 -0.661427 -0.079597<br>H 1.724328 -1.660719 -0.508771<br>H 2.553358 -0.108323 -0.626877<br>H 2.143135 -0.762941 0.955557<br>C -0.756667 -0.714609 0.221810<br>H -0.830994 -0.813616 1.318589<br>H -0.718545 -1.720852 -0.204916<br>N 0.448502 1.383407 0.172654<br>H -0.445793 1.817803 -0.007435<br>H 1.212821 1.923718 -0.203255<br>O -1.890220 0.004016 -0.258160<br>H -2.673637 -0.308107 0.198254
O_2<br>$\tilde{\nu}$: 1754.5, B: 0.0000000 44.6355338 44.6355338	O 0.000000 0.000000 0.594925<br>O 0.000000 0.000000 -0.594925
NO<br>$\tilde{\nu}$: 2066.0, B: 0.0000000 52.3561936 52.3561936	N 0.000000 0.000000 -0.606410<br>O 0.000000 0.000000 0.530609
$\dot{O}OC(CH_3)(NH_2)CH_2OH$<br><br>$\tilde{\nu}$: 130.0, 139.1, 203.5, 241.7, 288.1, 359.7, 375.1, 401.3, 433.4, 465.9, 511.9, 642.1, 780.4, 832.2, 848.0, 964.7, 1028.5, 1062.9, 1123.1, 1135.7, 1200.4, 1295.5, 1327.8, 1363.1, 1384.9, 1433.0, 1443.3, 1488.7, 1498.1, 1518.3, 1660.2, 3041.7, 3079.2, 3130.3, 3154.6, 3174.7, 3530.7, 3619.0, 3831.7<br><br>B: 3.7200000 0.0048493 2.1011409	C 0.041536 0.311501 0.042123<br>C -0.814167 -0.816636 -0.533421<br>O 1.391026 0.139277 -0.574982<br>N 0.072994 0.194627 1.455638<br>C -0.395557 1.682559 -0.411563<br>O -2.137419 -0.725561 -0.072033<br>O 2.025431 -0.875105 -0.082777<br>H -0.348589 -1.769678 -0.259654<br>H -0.835894 -0.741936 -1.619578<br>H 0.504658 0.993181 1.901632<br>H 0.558757 -0.649491 1.735834<br>H -1.439998 1.830182 -0.148495<br>H -0.286532 1.766805 -1.491005<br>H 0.211808 2.453144 0.062369<br>H -2.098344 -0.618042 0.884934
SP-37a<br><br>$\tilde{\nu}$: -946.5, 103.1, 121.0, 155.2, 197.9, 210.7, 287.3, 341.3, 410.3, 486.9, 553.2, 635.3, 653.3, 828.7, 874.4, 956.7, 1045.8, 1070.0, 1098.9, 1136.9, 1200.6, 1342.7, 1370.0, 1378.1, 1408.3, 1418.2, 1456.3, 1475.6, 1491.4, 1516.0, 1647.2, 1805.0, 3067.3, 3077.8, 3136.4, 3151.6, 3161.0, 3519.6, 3863.7<br><br>B: 3.7200000 0.0072609 1.8263400	C -0.231273 0.397197 0.172627<br>C -0.925979 -0.789642 -0.452362<br>O 1.569974 -0.123976 -0.874023<br>N 0.316819 0.199168 1.347427<br>C -0.579558 1.742113 -0.368258<br>O -2.262458 -0.832233 0.013525<br>O 2.179502 -0.661036 0.108741<br>H -0.382149 -1.703282 -0.205952<br>H -0.969870 -0.681749 -1.533572<br>H 0.615412 1.068747 1.780255<br>H 1.329521 -0.437012 1.018099

	H -1.660162 1.880258 -0.299707<br>H -0.305075 1.792909 -1.421174<br>H -0.076724 2.539557 0.174325<br>H -2.243959 -1.013647 0.957760
HN=C(CH_3)CH_2OH•HO_2<br><br>$\tilde{\upsilon}$: 32.0, 55.6, 69.2, 105.3, 153.8, 174.0, 274.6, 277.2, 285.4, 430.0, 514.2, 576.4, 789.9, 837.9, 868.2, 972.5, 1031.1, 1078.7, 1118.9, 1159.7, 1216.4, 1290.2, 1381.1, 1382.0, 1406.5, 1428.7, 1465.0, 1477.8, 1480.1, 1635.9, 1763.6, 2913.9, 3072.1, 3078.9, 3130.4, 3137.5, 3164.8, 3504.6, 3891.0<br><br>B: 3.6900000 0.0077462 1.3525438	C -0.778218 -0.577279 -0.007147<br>C -0.595604 0.921269 0.088057<br>O 2.503066 0.580702 0.631155<br>N 0.219335 -1.288956 -0.336540<br>C -2.133069 -1.109791 0.341328<br>O -1.773139 1.632875 -0.234335<br>O 2.656883 -0.197507 -0.408453<br>H 0.259031 1.228599 -0.513733<br>H -0.355091 1.153928 1.127421<br>H 0.011486 -2.284770 -0.317679<br>H 1.770519 -0.682245 -0.485617<br>H -2.864583 -0.766606 -0.389431<br>H -2.450993 -0.706956 1.303507<br>H -2.132873 -2.196774 0.376771<br>H -1.825973 1.743761 -1.185835
HN=C(CH_3)CH_2OH<br><br>$\tilde{\upsilon}$: 75.5, 147.9, 300.1, 405.7, 422.0, 481.7, 606.5, 821.4, 854.5, 987.6, 1026.6, 1099.1, 1101.6, 1167.5, 1275.9, 1282.6, 1385.5, 1417.0, 1467.2, 1479.9, 1485.9, 1503.6, 1769.5, 3018.3, 3038.7, 3064.6, 3127.5, 3156.7, 3510.0, 3729.2<br><br>B: 9.6800000 0.0074144 3.8364528	C 0.469181 0.139807 0.000001<br>C -0.736634 -0.767587 -0.000002<br>H -0.673782 -1.419267 0.879970<br>H -0.673781 -1.419264 -0.879975<br>C 1.808186 -0.537385 -0.000001<br>H 1.904879 -1.177526 -0.877942<br>H 2.618898 0.187921 0.000006<br>H 1.904875 -1.177540 0.877930<br>N 0.255045 1.386251 0.000004<br>H 1.116802 1.926902 0.000005<br>O -1.940683 -0.063688 -0.000001<br>H -1.702132 0.875509 0.000001
HO_2<br>$\tilde{\upsilon}$: 1252.8, 1459.4, 3686.8<br>B: 628.5431720 34.6992321 32.8838526	H -0.880747 -0.865418 0.000000<br>O 0.055047 0.708193 0.000000<br>O 0.055047 -0.600015 0.000000
SP-37b<br>$\tilde{\upsilon}$: -1086.2, 89.8, 124.2, 163.4, 199.6, 255.4, 314.4, 402.1, 435.9, 454.0, 517.2, 573.5, 623.6, 664.9, 745.1, 880.0, 974.4, 984.5, 1042.7, 1091.2, 1111.6, 1203.4, 1325.4, 1341.9, 1363.0, 1401.1, 1452.2, 1459.6, 1514.2, 1596.6, 1630.6, 1668.9, 3052.5, 3136.7, 3159.8, 3222.5, 3575.3, 3684.0, 3871.5<br><br>B: 3.9300000 0.0082370 1.6515400	C 0.265611 0.423510 0.088277<br>C 1.084350 -0.812442 -0.171711<br>O -1.523611 -0.660721 -0.772007<br>N 0.403916 1.405688 -0.838201<br>C -0.349851 0.647769 1.323173<br>O 2.421985 -0.624191 0.249159<br>O -2.347068 -0.466332 0.178926<br>H 1.029125 -1.075689 -1.232269<br>H 0.683359 -1.640627 0.405416<br>H -0.132794 2.248228 -0.706521<br>H 0.491204 1.117208 -1.800688<br>H -0.566706 1.676319 1.591626<br>H -0.007262 0.024796 2.140268<br>H -1.522510 0.124115 0.982851<br>H 2.787055 0.142774 -0.200332
CH_2=C(NH_2)CH_2OH•HO_2	C -0.473733 0.538847 0.063473<br>C -1.160536 -0.633703 -0.586095

$\tilde{\nu}$: 40.0, 60.4, 87.3, 106.3, 133.0, 217.9, 253.0, 313.6, 402.3, 413.4, 476.2, 545.4, 625.3, 718.7, 794.7, 864.1, 889.6, 983.6, 1020.3, 1100.0, 1136.6, 1224.1, 1280.6, 1337.9, 1384.0, 1425.0, 1459.1, 1520.3, 1527.3, 1647.7, 1721.4, 3038.9, 3142.4, 3164.4, 3264.7, 3327.0, 3556.0, 3657.4, 3860.9<br><br>B: 3.6600000 0.0042445 1.4141065	O 1.974913 -1.128448 0.194782<br>N -0.469366 0.454939 1.449227<br>C -0.014807 1.587279 -0.631823<br>O -2.538521 -0.661498 -0.267514<br>O 2.530047 -0.074212 -0.337634<br>H -0.665845 -1.558139 -0.268368<br>H -1.082286 -0.558783 -1.667928<br>H 0.011671 1.201306 1.927893<br>H -0.200977 -0.452724 1.804358<br>H 0.432840 2.441205 -0.137924<br>H -0.182981 1.647945 -1.696457<br>H 1.771264 0.519675 -0.565870<br>H -2.635171 -0.466333 0.669303
$CH_2=C(NH_2)CH_2OH$<br><br>$\tilde{\nu}$: 97.4, 256.6, 279.8, 401.9, 415.8, 454.2, 476.1, 628.9, 728.7, 839.4, 882.6, 985.3, 1004.2, 1112.5, 1126.8, 1212.8, 1353.5, 1383.8, 1422.6, 1455.2, 1504.6, 1633.0, 1733.1, 3064.0, 3104.7, 3181.3, 3272.7, 3600.9, 3716.8, 3867.4<br><br>B: 8.6500000 0.0054428 4.0407455	C 1.658338 -0.780869 -0.174698<br>C 0.577295 -0.033330 0.053714<br>N 0.549688 1.349159 0.022910<br>C -0.759754 -0.657596 0.361392<br>O -1.841558 0.022981 -0.247892<br>H 1.578464 -1.856537 -0.195840<br>H 2.632216 -0.340258 -0.338322<br>H -0.245449 1.792707 0.451167<br>H 1.423707 1.832696 0.135954<br>H -0.946932 -0.591577 1.434993<br>H -0.737707 -1.713349 0.085227<br>H -1.674926 0.079123 -1.192866
SP-37c<br><br>$\tilde{\nu}$: -1855.1, 182.9, 201.0, 267.6, 285.6, 305.9, 370.5, 378.5, 510.7, 566.7, 579.8, 669.0, 701.8, 811.5, 881.6, 896.1, 973.1, 1008.7, 1044.0, 1059.2, 1166.2, 1185.9, 1268.4, 1275.8, 1326.1, 1379.5, 1418.8, 1460.6, 1489.1, 1497.8, 1645.3, 1790.1, 3073.4, 3138.1, 3148.6, 3168.7, 3521.3, 3616.1, 3676.7<br><br>B: 3.1200000 0.0026144 2.6056120	C 0.141799 0.444462 0.015308<br>C -0.757149 -0.633046 -0.625391<br>O 1.429446 -0.028179 -0.361220<br>N -0.106812 0.405798 1.435712<br>C -0.035737 1.835326 -0.545854<br>O -1.986536 -0.777602 -0.084820<br>O 1.412191 -1.378046 -0.001163<br>H 0.148618 -1.521754 -0.337742<br>H -0.784379 -0.629462 -1.713516<br>H 0.234371 1.240471 1.893119<br>H 0.368600 -0.398424 1.832970<br>H -1.050908 2.178726 -0.354159<br>H 0.145193 1.829159 -1.617940<br>H 0.667763 2.522668 -0.075533<br>H -1.915856 -0.471797 0.836068
$CH_3(NH_2)C=CHOH•HO_2$<br><br>$\tilde{\nu}$: 40.0, 60.4, 87.3, 106.3, 133.0, 217.9, 253.0, 313.6, 402.3, 413.4, 476.2, 545.4, 625.3, 718.7, 794.7, 864.1, 889.6, 983.6, 1020.3, 1100.0, 1136.6, 1224.1, 1280.6, 1337.9, 1384.0, 1425.0, 1459.1, 1520.3, 1527.3, 1647.7, 1721.4, 3038.9, 3142.4, 3164.4, 3264.7, 3327.0, 3556.0, 3657.4, 3860.9<br><br>B: 3.6600000 0.0042445 1.4141065	C -0.473733 0.538847 0.063473<br>C -1.160536 -0.633703 -0.586095<br>O 1.974913 -1.128448 0.194782<br>N -0.469366 0.454939 1.449227<br>C -0.014807 1.587279 -0.631823<br>O -2.538521 -0.661498 -0.267514<br>O 2.530047 -0.074212 -0.337634<br>H -0.665845 -1.558139 -0.268368<br>H -1.082286 -0.558783 -1.667928<br>H 0.011671 1.201306 1.927893<br>H -0.200977 -0.452724 1.804358<br>H 0.432840 2.441205 -0.137924<br>H -0.182981 1.647945 -1.696457

	H 1.771264 0.519675 -0.565870<br>H -2.635171 -0.466333 0.669303
$CH_3(NH_2)C{=}CHOH$<br><br>$\tilde{\nu}$: 136.8, 223.2, 276.7, 291.4, 375.1, 492.6, 612.2, 674.4, 848.3, 901.4, 928.8, 1017.0, 1051.3, 1194.0, 1208.7, 1234.4, 1341.9, 1404.8, 1421.2, 1480.1, 1497.0, 1627.8, 1791.2, 3038.2, 3085.5, 3134.5, 3228.9, 3503.6, 3569.3, 3734.5<br><br>B: 8.9800000 0.0020062 3.9068275	C 1.769338 -0.632991 0.000001<br>C -0.720875 -0.743147 -0.000001<br>C 0.401808 -0.028635 0.000000<br>N 0.248865 1.402737 0.000001<br>O -1.958401 -0.200147 0.000000<br>H 2.337743 -0.319513 -0.879477<br>H 1.717973 -1.721182 0.000000<br>H 2.337741 -0.319514 0.879480<br>H -0.726561 -1.824211 -0.000003<br>H 0.689085 1.817554 -0.813897<br>H 0.689113 1.817556 0.813883<br>H -1.821566 0.759968 0.000005
$\dot{O}C(CH_3)(NH_2)CH_2OH$<br><br>$\tilde{\nu}$: 158.3, 217.3, 246.9, 331.5, 345.2, 396.8, 418.1, 423.7, 465.9, 608.3, 798.9, 821.4, 895.6, 937.7, 978.0, 1079.9, 1107.4, 1137.1, 1234.4, 1284.4, 1326.6, 1378.7, 1411.9, 1434.2, 1485.1, 1493.5, 1503.8, 1624.1, 3043.2, 3069.8, 3128.4, 3148.9, 3152.2, 3517.3, 3615.1, 3830.6<br><br>B: 5.2000000 0.0047670 2.9099189	C -0.412984 -0.010353 0.038503<br>C -1.695293 -0.803088 -0.094646<br>H -1.730415 -1.581008 0.667087<br>H -2.551538 -0.144712 0.045782<br>H -1.755127 -1.262470 -1.080554<br>C 0.830509 -0.856729 -0.224910<br>H 0.817621 -1.216391 -1.252988<br>H 0.814022 -1.717024 0.452193<br>N -0.401751 1.131292 -0.865442<br>H 0.504433 1.587110 -0.850420<br>H -1.132399 1.794024 -0.633865<br>O 2.001688 -0.093413 -0.058987<br>H 1.948703 0.312622 0.813746<br>O -0.306242 0.634641 1.256917
NO_2<br>$\tilde{\nu}$: 783.5, 1465.2, 1775.4<br>B: 253.9647127 13.2906276 12.6296837	N 0.000000 0.314443 0.000000<br>O 1.090260 -0.137566 0.000000<br>O -1.090260 -0.137572 0.000000

Table S11 Quantum chemistry results for reactions relevant to the atmospheric fate of the $(CH_3)_2(NH_2)CCH_2\dot{O}$ radical. Electronic energies of reactants, intermediates and products (/Hartree), and relative energies including Zero Point Energies, $\Delta E_{v=0}$ (/kJ mol^{-1}), of stationary points on the potential energy surface.

	M06-2X/aug-cc-pVTZ		
Species	E_{Elec}	E_{ZPE}	$\Delta E_{v=0}$
$(CH_3)_2C(NH_2)CH_2OH$	-289.007907	0.155148	
OH	-75.733810	0.008588	
O_2	-150.324815	0.004005	
Sum reactants	-515.066532	0.167741	52.5
$(CH_3)_2(NH_2)CH_2\dot{O}$	-288.329715	0.140288	
H_2O	-76.430107	0.021551	
O_2	-150.324815	0.004005	
Sum intermediates	-515.084637	0.165844	0.0
SP-41	-288.325641	0.138254	
H_2O	-76.430107	0.021551	
O_2	-150.324815	0.004005	
Sum intermediates	-515.080563	0.163810	5.4
$(CH_3)_2\dot{C}NH_2{\bullet}CH_2O$	-288.326591	0.137475	
H_2O	-76.430107	0.021551	
O_2	-150.324815	0.004005	
	-515.081513	0.163031	0.8
$(CH_3)_2\dot{C}NH_2$	-173.815617	0.107445	
CH_2O	-114.498970	0.027013	
H_2O	-76.430107	0.021551	
O_2	-150.324815	0.004005	
Sum Products	-515.069510	0.160013	24.4
$(CH_3)_2(NH_2)CCHO$	-287.792823	0.130398	
H_2O	-76.430107	0.021551	
HO_2	-150.908108	0.014608	
Sum Products	-515.131037	0.166556	-120.0

Table S11, continued: Vibrational frequencies (/cm^{-1}), Rotational constants (B /GHZ) and Cartesian Coordinates (/Å) of the species listed above. Results from M06-2X/aug-cc-pVTZ calculations.

$(CH_3)_2C(NH_2)CH_2OH$<br><br>$\tilde{\upsilon}$: 152.3, 223.0, 250.0, 260.7, 285.3, 348.6, 369.4, 415.9, 444.4, 524.9, 571.3, 779.0, 872.0, 916.4, 950.0, 958.0, 995.5, 1048.4, 1097.6, 1128.4, 1204.4, 1240.0, 1295.1, 1351.3, 1376.2, 1404.4, 1417.9, 1443.9, 1484.9, 1486.7, 1502.9, 1511.5, 1516.9, 1645.1, 2997.1, 3048.0, 3059.1, 3099.4, 3119.6, 3122.9, 3128.7, 3153.7, 3513.2, 3597.8, 3790.1<br><br>B: 4.5514025 2.7033291 2.6683275	C -1.618997 -0.812180 -0.391283<br>C -0.392830 0.004617 0.007282<br>C -0.477049 1.410149 -0.573757<br>C 0.870655 -0.703054 -0.492733<br>N -0.235875 0.124321 1.465523<br>O 2.042510 -0.032764 -0.100073<br>H -1.575536 -1.812755 0.043301<br>H -1.685999 -0.916200 -1.475631<br>H -2.530242 -0.326384 -0.039464<br>H 0.412768 1.983011 -0.319887<br>H -1.354675 1.930794 -0.185306<br>H -0.563283 1.366947 -1.659331

	H 0.865200 -0.748464 -1.582807<br>H 0.866794 -1.734170 -0.112844<br>H -0.396947 -0.770393 1.915290<br>H -0.908038 0.777629 1.848907<br>H 1.890326 0.244657 0.812639
OH<br>ṽ: 3769.6; B: 564.2863408	O 0.000000 0.000000 0.107992<br>H 0.000000 0.000000 -0.863937
O_2<br>ṽ: 1754.5, B: 0.0000000 44.6355338 44.6355338	O 0.000000 0.000000 0.594925<br>O 0.000000 0.000000 -0.594925
$(CH_3)_2(NH_2)CCH_2\dot{O}$<br><br>ṽ: 142.9, 224.0, 238.3, 277.2, 310.2, 337.2, 363.2, 414.6, 447.0, 566.8, 724.5, 778.9, 879.9, 911.2, 956.2, 1004.8, 1016.2, 1068.7, 1082.4, 1136.8, 1230.0, 1250.4, 1343.4, 1359.9, 1381.5, 1405.9, 1417.0, 1484.9, 1492.4, 1500.8, 1510.3, 1654.1, 2925.2, 2993.9, 3052.3, 3058.8, 3120.4, 3127.7, 3143.7, 3147.9, 3507.9, 3590.4<br><br>B: 4.5934692 2.7587965 2.7233931	C -1.561315 -0.760062 -0.516583<br>C -0.371762 0.013929 0.031556<br>C -0.454540 1.485022 -0.363210<br>C 0.922038 -0.578964 -0.557903<br>N -0.386842 -0.066061 1.488515<br>O 2.066953 -0.038668 -0.045779<br>H -1.516807 -1.808698 -0.213585<br>H -1.583952 -0.721914 -1.606278<br>H -2.487220 -0.333739 -0.133361<br>H 0.403228 2.030808 0.033160<br>H -1.363035 1.925874 0.043661<br>H -0.459281 1.596913 -1.448229<br>H 0.935115 -0.544719 -1.657738<br>H 0.981499 -1.651354 -0.290994<br>H 0.458818 0.351694 1.861862<br>H -0.402627 -1.032641 1.794963
SP-41<br><br>ṽ: -168.1, 108.4, 181.4, 193.8, 223.7, 257.7, 322.5, 392.9, 402.1, 429.5, 454.5, 679.3, 724.3, 814.7, 956.6, 1001.7, 1045.4, 1071.2, 1077.1, 1150.7, 1242.3, 1365.4, 1376.9, 1414.3, 1419.7, 1462.2, 1479.4, 1484.6, 1494.5, 1505.7, 1612.6, 1661.1, 2911.6, 2973.2, 3042.1, 3048.5, 3107.7, 3121.5, 3142.3, 3153.6, 3538.8, 3641.1<br><br>B: 4.4300000 0.0003170 2.5579051	C -1.534456 -0.945220 -0.422041<br>C -0.571603 0.088922 0.073927<br>C -0.488549 1.378440 -0.686517<br>C 1.254734 -0.729977 -0.417687<br>N -0.525630 0.236992 1.446121<br>O 2.098103 0.036317 0.068192<br>H -1.461666 -1.866447 0.157236<br>H -1.343095 -1.176422 -1.469043<br>H -2.560157 -0.573910 -0.340109<br>H 0.387426 1.945900 -0.373711<br>H -1.384451 1.976368 -0.498572<br>H -0.414840 1.191860 -1.756345<br>H 1.046837 -0.722214 -1.501931<br>H 1.006796 -1.681489 0.085434<br>H 0.232064 0.825925 1.767742<br>H -0.575077 -0.622044 1.974815
$(CH_3)_2\dot{C}NH_2{\bullet}CH_2O$<br><br>ṽ: 64.3, 89.6, 138.7, 163.3, 187.5, 214.7, 296.3, 365.0, 383.7, 405.3, 431.3, 499.0, 723.8, 814.8, 960.7, 995.3, 1043.1, 1079.2, 1097.4, 1121.9, 1253.7, 1371.6, 1382.3, 1418.5, 1421.8, 1472.7, 1485.0, 1492.6, 1498.5, 1515.4, 1655.9, 1741.2, 2942.8, 3003.7, 3006.6, 3015.7, 3081.5, 3083.3, 3127.9, 3137.8, 3526.0, 3635.1<br><br>B: 4.4200000 0.0064248 2.2493785	C -1.625281 -0.964368 -0.462520<br>C -0.719254 0.081101 0.087628<br>C -0.640089 1.417491 -0.567186<br>C 1.511350 -0.620973 -0.567242<br>N -0.521277 0.079788 1.453315<br>O 2.214016 0.068258 0.143196<br>H -1.434136 -1.936746 -0.004184<br>H -1.490473 -1.059966 -1.539974<br>H -2.679448 -0.718397 -0.283150<br>H 0.286931 1.924212 -0.288985<br>H -1.477214 2.054894 -0.259498

	H -0.669800 1.322024 -1.651572<br>H 1.274681 -0.335341 -1.605636<br>H 1.207879 -1.639513 -0.271281<br>H 0.269468 0.636964 1.754586<br>H -0.511435 -0.832217 1.886834
$(CH_3)_2\dot{C}NH_2$<br><br>ṽ: 182.2, 182.6, 327.4, 342.9, 374.4, 413.9, 687.1, 812.6, 961.3, 986.9, 1030.5, 1067.9, 1090.8, 1354.1, 1363.1, 1413.7, 1421.3, 1471.3, 1483.5, 1488.0, 1502.8, 1641.5, 2973.5, 2978.8, 3073.2, 3073.4, 3129.6, 3132.3, 3550.4, 3651.7<br><br>B: 8.9500000 0.0030434 8.1894265	C 0.201439 0.092794 0.000000<br>C 0.201439 -0.661842 -1.284079<br>H 0.417994 -0.008026 -2.131675<br>H 0.951089 -1.451805 -1.267973<br>H -0.773441 -1.132327 -1.479102<br>C 0.201439 -0.661842 1.284079<br>H 0.951089 -1.451805 1.267973<br>H 0.417994 -0.008026 2.131675<br>H -0.773441 -1.132327 1.479102<br>N -0.557631 1.271394 0.000000<br>H -0.456885 1.834946 0.831590<br>H -0.456885 1.834946 -0.831590
CH_2O<br>ṽ: 1213.6, 1273.5, 1539.9, 1869.1, 2945.5, 3015.7<br><br>B: 284.7518606 39.4556659 34.6539588	C 0.000000 0.000000 -0.525505<br>H 0.000000 0.938356 -1.105549<br>H 0.000000 -0.938356 -1.105549<br>O 0.000000 0.000000 0.670516
$(CH_3)_2(NH_2)CCHO$<br><br>ṽ: 74.1, 216.7, 245.0, 272.2, 284.5, 334.3, 375.0, 417.7, 427.1, 607.9, 785.9, 829.2, 924.4, 957.2, 986.1, 1006.2, 1052.6, 1100.0, 1255.1, 1280.5, 1327.8, 1396.9, 1399.5, 1424.2, 1481.1, 1485.3, 1501.2, 1512.2, 1653.9, 1862.5, 2958.9, 3057.3, 3062.5, 3128.9, 3134.5, 3146.1, 3149.7, 3520.3, 3603.3<br><br>B: 4.6200000 0.0023569 2.8448082	C 0.221434 1.478436 -0.396810<br>O -2.077625 -0.055657 -0.005905<br>C -1.038114 -0.580284 0.278530<br>C 0.337962 0.020880 0.026328<br>N 1.100356 -0.193823 1.255969<br>C 0.975406 -0.826748 -1.075439<br>H -0.242171 2.069515 0.393219<br>H -0.396166 1.577956 -1.288127<br>H 1.213157 1.882333 -0.603089<br>H -1.002158 -1.579905 0.752767<br>H 0.847662 0.486432 1.962812<br>H 2.092997 -0.094666 1.081639<br>H 1.020225 -1.874368 -0.777869<br>H 1.993308 -0.477641 -1.258403<br>H 0.411525 -0.741345 -2.003144
HO_2<br>ṽ: 1252.8, 1459.4, 3686.8<br>B: 628.5431720 34.6992321 32.8838526	H -0.880747 -0.865418 0.000000<br>O 0.055047 0.708193 0.000000<br>O 0.055047 -0.600015 0.000000

Table S12 Quantum chemistry results for reactions relevant to the $(CH_3)_2(NH_2)\dot{C}$ + O_2 reaction. Electronic energies of reactants, intermediates and products (/Hartree), and relative energies including Zero Point Energies, $\Delta E_{v=0}$ (/kJ mol^{-1}), of stationary points on the potential energy surface.

	M06-2X/aug-cc-pVTZ		
Species	E_{Elec}	E_{ZPE}	$\Delta E_{v=0}$
$(CH_3)_2(NH_2)\dot{C}$	-173.815617	0.107445	
O_2	-150.324815	0.004005	
NO	-129.893351	0.004707	
Sum reactants	-454.033784	0.116156	0
$(CH_3)_2(NH_2)CO\dot{O}$	-324.203364	0.117513	
NO	-129.893351	0.004707	
Sum intermediates	-454.096716	0.122220	-149.3
SP-44a	-324.167361	0.112047	
NO	-129.893351	0.004707	
Sum intermediates	-454.060713	0.116753	-69.1
$(CH_3)_2C{=}NH{\bullet}HO_2$	-324.186363	0.114500	
NO	-129.893351	0.004707	
Sum intermediates	-454.079714	0.119207	-112.6
$(CH_3)_2C{=}NH$	-173.254901	0.097327	
HO_2	-150.908108	0.014608	
NO	-129.893351	0.004707	
Sum products	-454.056360	0.116641	-58.0
SP-44b	-324.155908	0.111224	
NO	-129.893351	0.004707	
Sum intermediates	-454.049259	0.115931	-41.2
$(CH_3)(NH_2)CCH_2{\bullet}HO_2$	-324.174469	0.114369	
NO	-129.893351	0.004707	
Sum intermediates	-454.067820	0.119076	-81.7
$CH_3(NH_2)C{=}CH_2$	-173.250646	0.097658	
HO_2	-150.908108	0.014608	
NO	-129.893351	0.004707	
Sum products	-454.052105	0.116973	-46.0
$(CH_3)_2(NH_2)C\dot{O}$	-249.031588	0.112840	
NO_2	-205.074635	0.009168	
Sum products	-454.106223	0.122008	-174.8

Table S12, continued: Vibrational frequencies (/cm^{-1}), Rotational constants (B /GHZ) and Cartesian Coordinates (/Å) of the species listed above. Results from M06-2X/aug-cc-pVTZ calculations.

$(CH_3)_2(NH_2)\dot{C}$	C	0.201439	0.092794	0.000000
	C	0.201439	-0.661842	-1.284079
$\tilde{\upsilon}$: 182.2, 182.6, 327.4, 342.9, 374.4, 413.9, 687.1, 812.6,	H	0.417994	-0.008026	-2.131675
961.3, 986.9, 1030.5, 1067.9, 1090.8, 1354.1, 1363.1,	H	0.951089	-1.451805	-1.267973

1413.7, 1421.3, 1471.3, 1483.5, 1488.0, 1502.8, 1641.5, 2973.5, 2978.8, 3073.2, 3073.4, 3129.6, 3132.3, 3550.4, 3651.7<br><br>B: 8.9500000 0.0030434 8.1894265	H -0.773441 -1.132327 -1.479102<br>C 0.201439 -0.661842 1.284079<br>H 0.951089 -1.451805 1.267973<br>H 0.417994 -0.008026 2.131675<br>H -0.773441 -1.132327 1.479102<br>N -0.557631 1.271394 0.000000<br>H -0.456885 1.834946 0.831590<br>H -0.456885 1.834946 -0.831590
O_2<br>$\tilde{\nu}$: 1754.5, B: 0.0000000 44.6355338 44.6355338	O 0.000000 0.000000 0.594925<br>O 0.000000 0.000000 -0.594925
NO<br>$\tilde{\nu}$: 2066.0, B: 0.0000000 52.3561936 52.3561936	N 0.000000 0.000000 -0.606410<br>O 0.000000 0.000000 0.530609
$(CH_3)_2(NH_2)CO\dot{O}$<br><br>$\tilde{\nu}$: 128.8, 208.5, 259.5, 281.0, 339.2, 376.1, 393.7, 430.0, 473.3, 584.0, 724.3, 819.0, 842.8, 953.6, 1014.9, 1039.3, 1099.0, 1154.1, 1306.3, 1332.6, 1373.4, 1417.3, 1429.7, 1484.8, 1489.9, 1502.1, 1505.3, 1666.0, 3075.5, 3078.5, 3151.4, 3156.2, 3162.5, 3168.4, 3537.0, 3624.0<br><br>B: 4.7000000 0.0028944 3.0060757	C 0.372948 0.031938 0.072441<br>O -0.809013 -0.518164 -0.690353<br>N 0.279469 -0.269006 1.452733<br>C 0.365031 1.528122 -0.160279<br>C 1.540433 -0.675898 -0.576491<br>O -1.931849 -0.159799 -0.162694<br>H 0.278287 -1.266368 1.624269<br>H -0.552850 0.138672 1.859573<br>H 0.377216 1.744931 -1.226787<br>H 1.242334 1.967843 0.309337<br>H -0.531208 1.966109 0.277019<br>H 1.483102 -1.748854 -0.393834<br>H 2.468917 -0.294981 -0.157060<br>H 1.534348 -0.505577 -1.651294
SP-44a<br><br>$\tilde{\nu}$: -912.2, 119.8, 151.7, 191.9, 230.1, 255.8, 377.1, 465.1, 492.5, 560.0, 660.6, 834.2, 853.8, 951.3, 1007.1, 1083.6, 1103.5, 1148.8, 1342.0, 1378.5, 1408.5, 1422.2, 1455.5, 1475.5, 1477.9, 1486.8, 1519.8, 1646.8, 1827.9, 3059.2, 3063.7, 3131.2, 3136.4, 3162.6, 3174.6, 3526.7<br><br>B: 4.8200000 0.0044057 2.6245361	C -0.665314 -0.008251 -0.238505<br>O 1.162384 -0.029246 0.961199<br>N -0.024564 -0.500239 -1.271874<br>C -0.750181 1.479102 -0.110299<br>C -1.599805 -0.820973 0.595867<br>O 1.981547 -0.113789 -0.013704<br>H -0.120238 -1.509730 -1.331526<br>H 1.123447 -0.252698 -0.988034<br>H -0.799832 1.779843 0.933505<br>H -1.660551 1.815582 -0.613184<br>H 0.102720 1.952281 -0.590705<br>H -1.414710 -1.886870 0.482949<br>H -2.630073 -0.604937 0.304126<br>H -1.488471 -0.546796 1.643651
$(CH_3)_2C{=}NH{\bullet}HO_2$<br><br>$\tilde{\nu}$: 7.2, 66.9, 119.3, 123.7, 161.4, 194.0, 265.8, 409.9, 484.7, 553.7, 798.4, 838.6, 861.6, 947.8, 1036.0, 1101.7, 1122.9, 1166.6, 1298.3, 1379.0, 1413.5, 1443.0, 1471.1, 1474.5, 1488.6, 1498.8, 1661.6, 1761.6, 2915.8, 3059.4, 3065.8, 3119.1, 3129.0, 3157.2, 3172.8, 3496.7<br><br>B: 7.2700000 0.0017023 1.4932836	C 1.162257 -0.056748 -0.001098<br>O -2.559707 0.608359 0.026679<br>N 0.287155 -0.977928 0.000376<br>C 0.736327 1.377566 -0.014484<br>C 2.641606 -0.313073 0.009565<br>O -2.388268 -0.686078 -0.019841<br>H 0.706184 -1.905437 0.009472<br>H -1.384286 -0.821647 -0.013713<br>H 1.145041 1.871616 -0.897759<br>H 1.150747 1.890499 0.855170<br>H -0.347291 1.460394 -0.011412

	H 2.870117 -1.376564 0.014792<br>H 3.090064 0.153896 0.887398<br>H 3.101999 0.148013 -0.865184
$(CH_3)_2C{=}NH$<br><br>ṽ: 119.9, 186.8, 390.7, 474.6, 520.1, 826.6, 832.7, 937.3, 1051.0, 1091.3, 1115.2, 1137.6, 1358.1, 1405.0, 1427.9, 1471.1, 1474.6, 1487.5, 1493.8, 1764.7, 3056.7, 3062.1, 3114.7, 3120.7, 3151.7, 3176.0, 3473.0<br><br>B: 9.7798437 8.5762282 4.8407204	C -1.171848 -0.790283 0.000001<br>C 1.353576 -0.476341 0.000005<br>C -0.005561 0.162941 -0.000001<br>N -0.096294 1.426459 -0.000007<br>H -1.126482 -1.438630 -0.876775<br>H -2.122136 -0.260221 -0.000012<br>H -1.126494 -1.438609 0.876794<br>H 1.469605 -1.115448 -0.877301<br>H 1.469601 -1.115439 0.877318<br>H 2.126358 0.286428 0.000003<br>H -1.073392 1.718804 -0.000010
HO_2<br>ṽ: 1252.8, 1459.4, 3686.8<br>B: 628.5431720 34.6992321 32.8838526	H -0.880747 -0.865418 0.000000<br>O 0.055047 0.708193 0.000000<br>O 0.055047 -0.600015 0.000000
SP-44b<br><br>ṽ: -1078.0, 102.0, 175.1, 200.9, 212.8, 366.8, 423.6, 459.3, 500.7, 535.6, 567.0, 613.5, 713.4, 873.2, 975.7, 1009.9, 1053.0, 1063.4, 1112.3, 1343.2, 1349.5, 1396.4, 1442.9, 1462.2, 1478.7, 1495.6, 1589.1, 1618.2, 1672.0, 3062.8, 3132.7, 3134.8, 3176.3, 3218.9, 3589.5, 3700.9<br><br>B: 4.6000000 0.0058782 2.5432283	C 0.756370 0.204244 -0.089709<br>O -1.243229 -0.803891 0.493334<br>N 1.213484 0.472559 1.151318<br>C 0.086367 1.183999 -0.843676<br>C 1.220265 -1.078372 -0.696468<br>O -2.052023 0.046234 -0.009962<br>H 1.412175 -0.305858 1.758286<br>H 0.863442 1.295188 1.614745<br>H 0.168758 1.084173 -1.919152<br>H 0.154665 2.204568 -0.480816<br>H -1.136540 0.805615 -0.568135<br>H 1.183167 -1.888965 0.029773<br>H 2.250845 -0.959619 -1.038632<br>H 0.593098 -1.340977 -1.543150
$(CH_3)(NH_2)CCH_2{\bullet}HO_2$<br><br>ṽ: 43.6, 74.7, 104.6, 121.5, 177.2, 213.8, 384.4, 427.5, 470.1, 529.2, 571.2, 619.3, 779.5, 829.4, 879.4, 996.2, 1036.6, 1074.3, 1147.8, 1281.4, 1377.0, 1425.6, 1446.2, 1484.8, 1494.8, 1537.3, 1654.5, 1705.7, 3068.3, 3129.7, 3168.3, 3172.1, 3238.5, 3267.6, 3583.5, 3686.8<br><br>B: 4.3000000 0.0059092 2.1009235	C 0.996304 0.174840 0.030260<br>O -1.837894 -0.824200 0.077779<br>N 1.062824 -0.221536 1.349911<br>C 0.746728 1.441204 -0.346123<br>C 1.286968 -0.918598 -0.951300<br>O -2.087669 0.438079 -0.144139<br>H 0.779429 -1.171565 1.530877<br>H 0.703804 0.429991 2.029574<br>H 0.827013 1.726011 -1.383754<br>H 0.587145 2.223275 0.385594<br>H -1.195839 0.846208 -0.304622<br>H 0.576500 -1.735536 -0.820288<br>H 2.291002 -1.308733 -0.780692<br>H 1.215686 -0.554609 -1.972203
$CH_3(NH_2)C{=}CH_2$<br><br>ṽ: 185.3, 344.5, 421.9, 462.2, 506.6, 597.4, 742.7, 844.7, 874.5, 990.6, 1025.5, 1071.3, 1148.9, 1362.9, 1417.6, 1449.1, 1483.7, 1495.1, 1650.0, 1741.3, 3055.2, 3110.5, 3169.4, 3179.4, 3272.4, 3580.6, 3683.4	C 1.408334 0.126462 0.001966<br>N -0.599483 -1.222672 0.084952<br>C -0.088978 0.072652 -0.000235<br>C -0.855142 1.163293 -0.015235<br>H 1.760146 1.153808 -0.021747<br>H 1.811552 -0.397770 -0.867669

B: 9.6780274 9.0843100 4.8473191	H 1.801808 -0.364444 0.893266<br>H -1.578415 -1.314500 -0.135849<br>H -0.040168 -1.934032 -0.358227<br>H -1.934842 1.096091 -0.015598<br>H -0.408983 2.145108 -0.007817
$(CH_3)_2(NH_2)C\dot{O}$<br><br>$\tilde{\upsilon}$: 209.0, 260.8, 336.1, 342.8, 376.1, 423.9, 434.0, 500.4, 787.6, 829.9, 904.6, 932.6, 988.2, 1031.3, 1085.1, 1163.4, 1301.0, 1338.9, 1408.4, 1414.4, 1477.6, 1487.2, 1496.0, 1502.5, 1645.1, 3066.7, 3070.9, 3145.5, 3147.7, 3150.0, 3155.7, 3513.6, 3604.5<br><br>B: 5.2300000 0.0063681 5.2107748	C -1.263979 -0.830374 -0.171305<br>C 0.000000 -0.016912 0.047771<br>C 1.263979 -0.830373 -0.171306<br>N -0.000001 1.143119 -0.838902<br>O 0.000000 0.602014 1.288647<br>H -2.140143 -0.197683 -0.032059<br>H -1.307638 -1.641656 0.554155<br>H -1.276961 -1.249540 -1.176298<br>H 2.140143 -0.197681 -0.032060<br>H 1.276962 -1.249540 -1.176299<br>H 1.307640 -1.641654 0.554155<br>H 0.823853 1.712880 -0.679707<br>H -0.823855 1.712879 -0.679707
NO_2<br>$\tilde{\upsilon}$: 783.5, 1465.2, 1775.4<br>B: 253.9647127 13.2906276 12.6296837	N 0.000000 0.314443 0.000000<br>O 1.090260 -0.137566 0.000000<br>O -1.090260 -0.137572 0.000000

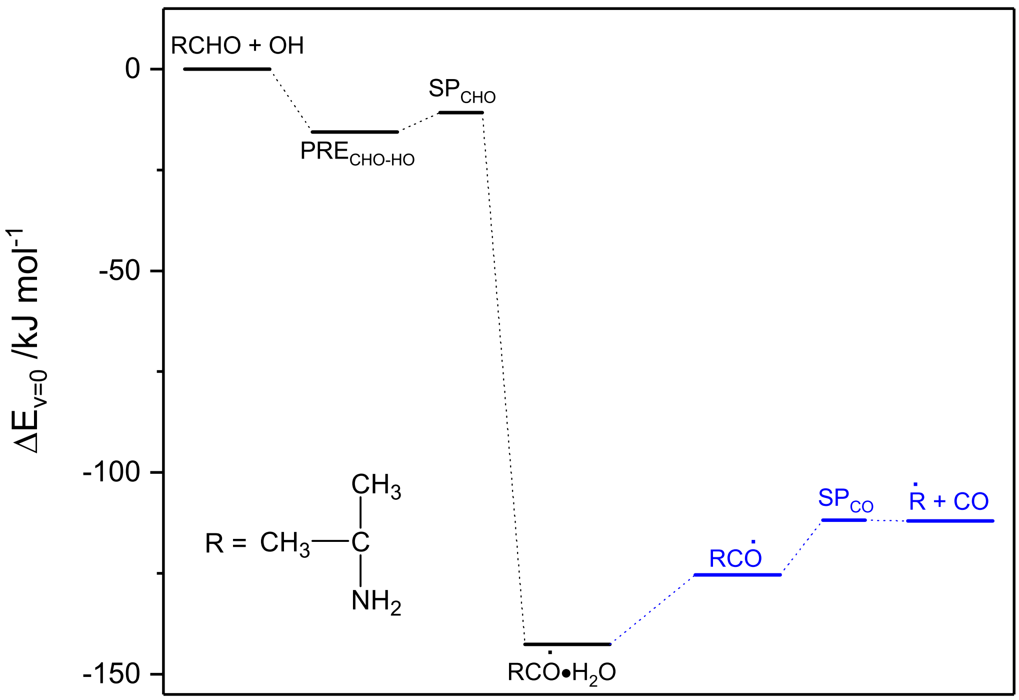

Figure S17 Relative energies of stationary points on the potential energy surface of the $(CH_3)_2(NH_2)CCHO$ + OH aldehydic H-abstraction reaction. Levels in blue color include the energy of H_2O. Results from M06-2X/aug-cc-pVTZ calculations.

Table S13 Quantum chemistry results for reactions relevant to the $(CH_3)_2(NH_2)CCHO$ + OH aldehydic H-abstraction reaction. Electronic energies of reactants, intermediates and products (/Hartree), and relative energies including Zero Point Energies, $\Delta E_{v=0}$ (/kJ mol^{-1}), of stationary points on the potential energy surface.

	M06-2X/aug-cc-pVTZ		
Species	E_{Elec}	E_{ZPE}	$\Delta E_{v=0}$
$(CH_3)_2(NH_2)CCHO$	-287.794367	0.130463	
OH	-75.733810	0.008588	
Sum reactants	-363.528177	0.139051	0
PRE$_{CHO-HO}$	-363.536836	0.141777	-15.6
SP$_{CHO}$	-363.532646	0.139407	-10.8
$(CH_3)_2(NH_2)C\dot{C}O{\bullet}H_2O$	-363.584946	0.141669	-142.2
$(CH_3)_2(NH_2)C\dot{C}O$	-287.146358	0.117998	
H_2O	-76.430092	0.021558	
Sum intermediates	-363.576450	0.139556	-125.4
SP$_{CO}$	-287.138150	0.114985	
H_2O	-76.430092	0.021558	

Sum intermediates	-363.568242	0.136543	-111.8
$(CH_3)_2(NH_2)\dot{C}$	-173.815617	0.107445	
CO	-113.320241	0.005176	
H_2O	-76.430092	0.021558	
Sum products	-363.565950	0.134179	-112.0

Table S13, continued: Vibrational frequencies (/cm^{-1}), Rotational constants (B /GHZ) and Cartesian Coordinates (/Å) of the species listed above. Results from M06-2X/aug-cc-pVTZ calculations.

$(CH_3)_2(NH_2)CCHO$<br><br>$\tilde{\upsilon}$: 79.6, 195.3, 249.4, 258.9, 293.0, 338.8, 348.1, 408.5, 437.6, 633.5, 816.6, 886.8, 913.2, 952.0, 964.5, 1027.6, 1035.8, 1111.6, 1241.3, 1298.4, 1346.3, 1396.6, 1402.6, 1422.3, 1483.1, 1490.4, 1501.2, 1511.7, 1665.2, 1860.7, 2924.5, 3058.2, 3060.7, 3129.7, 3131.4, 3146.3, 3148.5, 3512.3, 3584.3<br><br>B: 4.6200000 0.0017434 2.8249125	C -0.335956 -0.000002 -0.047719<br>C -1.087687 -1.253903 0.397883<br>C -1.087636 1.253972 0.397766<br>N -0.216392 -0.000071 -1.494545<br>C 1.011952 0.000011 0.660860<br>O 2.072474 -0.000006 0.101363<br>H -0.570870 -2.154371 0.062847<br>H -2.084709 -1.244415 -0.039568<br>H -1.179432 -1.293509 1.483688<br>H -0.570793 2.154385 0.062625<br>H -1.179360 1.293695 1.483569<br>H -2.084667 1.244473 -0.039666<br>H 0.321013 -0.807436 -1.792247<br>H 0.321090 0.807218 -1.792315<br>H 0.958637 0.000043 1.769239
$PRE_{CHO\text{-}HO}$<br><br>$\tilde{\upsilon}$: 34.3, 45.6, 80.1, 158.4, 220.5, 249.7, 281.5, 295.7, 341.2, 380.5, 419.1, 428.9, 443.7, 596.1, 615.8, 789.4, 824.4, 928.1, 957.2, 991.2, 1010.6, 1061.6, 1100.4, 1257.6, 1285.2, 1326.5, 1398.2, 1404.5, 1427.5, 1482.3, 1485.7, 1501.5, 1513.4, 1655.7, 1837.8, 2993.3, 3059.5, 3064.7, 3132.2, 3137.4, 3147.7, 3151.6, 3526.1, 3581.1, 3609.4<br><br>B: 4.0900000 0.0018106 1.1546560	C 1.515301 1.366866 -0.012154<br>O -1.221432 0.846166 -0.112855<br>C -0.508853 -0.106205 0.079203<br>C 1.007381 -0.067461 0.038687<br>N 1.455107 -0.830136 1.202001<br>C 1.408350 -0.843477 -1.218355<br>H 1.214149 1.911516 0.882884<br>H 1.110403 1.895813 -0.873517<br>H 2.603681 1.366958 -0.073854<br>H -0.933981 -1.103488 0.292014<br>H 1.419031 -0.268687 2.044102<br>H 2.411446 -1.139669 1.081729<br>H 1.014218 -1.859045 -1.187451<br>H 2.496878 -0.897478 -1.273314<br>H 1.043963 -0.343759 -2.114531<br>O -3.789656 -0.356525 0.051952<br>H -3.009913 0.233331 -0.029131
SP_{CHO}<br><br>$\tilde{\upsilon}$: -234.8, 54.5, 71.8, 81.4, 147.7, 213.0, 260.1, 265.7, 290.2, 347.3, 353.1, 408.1, 441.8, 531.5, 632.0, 821.6, 878.2, 894.7, 947.0, 965.4, 1028.6, 1038.5, 1100.6, 1220.7, 1266.1, 1336.2, 1343.7, 1399.7, 1416.9, 1487.8, 1490.3, 1504.4, 1511.5, 1660.6, 1862.7, 2342.8, 3060.7, 3062.4, 3134.6, 3135.9, 3152.0, 3154.2, 3515.6, 3591.2, 3769.6<br><br>B: 2.8300000 0.0090612 1.7979273	C 0.672052 -0.278542 -0.000017<br>C 0.368954 -1.095299 1.255176<br>C 0.368791 -1.095537 -1.255016<br>N 2.062639 0.126474 -0.000146<br>C -0.293196 0.906610 -0.000068<br>O 0.007373 2.059805 -0.000200<br>H 0.606513 -0.524880 2.154404<br>H 0.982780 -1.994557 1.244190<br>H -0.682871 -1.377914 1.289377<br>H 0.606237 -0.525290 -2.154383<br>H -0.683039 -1.378156 -1.289030<br>H 0.982616 -1.994795 -1.243938<br>H 2.261148 0.701739 0.810882

	H 2.261041 0.701584 -0.811311<br>H -1.378792 0.579659 0.000035<br>O -2.842564 -0.329078 0.000218<br>H -3.412188 0.458082 0.000198
$(CH_3)_2(NH_2)C\dot{C}O{\bullet}H_2O$<br><br>$\tilde{\nu}$: 39.7, 64.7, 81.4, 87.3, 120.1, 231.0, 256.2, 260.9, 289.3, 301.0, 344.1, 366.1, 400.3, 417.2, 501.4, 592.3, 610.7, 839.6, 853.6, 970.7, 1024.9, 1038.5, 1071.1, 1172.2, 1291.0, 1341.8, 1405.5, 1421.6, 1486.9, 1491.8, 1506.1, 1508.4, 1614.8, 1657.3, 1976.9, 3062.2, 3063.7, 3136.1, 3137.0, 3158.7, 3161.0, 3526.5, 3609.0, 3761.6, 3933.2<br>B: 2.7800000 0.0048718 1.6974986	C -0.632255 -0.362790 0.000269<br>C -0.116290 -1.053095 -1.255331<br>C -0.120082 -1.045775 1.261508<br>N -2.073751 -0.338107 -0.001949<br>C -0.013463 1.086542 -0.002234<br>O -0.594045 2.102522 -0.001998<br>H -0.468298 -0.542295 -2.152889<br>H -0.496591 -2.073651 -1.266594<br>H 0.972269 -1.070782 -1.263887<br>H -0.475059 -0.529902 2.154991<br>H 0.968454 -1.063063 1.273234<br>H -0.500238 -2.066322 1.277371<br>H -2.434873 0.134620 -0.821779<br>H -2.437273 0.140716 0.813274<br>H 2.228278 0.634030 -0.003946<br>O 2.931211 -0.028571 -0.004216<br>H 3.754790 0.462496 0.028307
$(CH_3)_2(NH_2)C\dot{C}O$<br><br>$\tilde{\nu}$: 30.7, 221.4, 253.7, 282.6, 305.0, 342.9, 376.4, 410.2, 475.0, 594.1, 632.4, 840.1, 868.4, 966.4, 1017.3, 1034.4, 1071.7, 1174.9, 1287.5, 1342.2, 1400.2, 1415.5, 1483.7, 1487.2, 1499.1, 1508.2, 1660.1, 1971.9, 3063.6, 3065.4, 3136.7, 3137.6, 3154.6, 3157.3, 3523.1, 3603.8<br><br>B: 4.7100000 0.0098083 2.8543563	C 1.066284 1.257894 -0.421923<br>O -2.106362 -0.000001 -0.198659<br>C -1.050048 -0.000002 -0.704684<br>C 0.338011 -0.000000 0.031105<br>N 0.206212 -0.000003 1.469409<br>C 1.066290 -1.257888 -0.421927<br>H 0.532580 2.153243 -0.100373<br>H 1.152752 1.280029 -1.506886<br>H 2.060371 1.267495 0.022889<br>H -0.303618 -0.815497 1.788371<br>H -0.303620 0.815489 1.788374<br>H 0.532592 -2.153242 -0.100380<br>H 2.060378 -1.267486 0.022884<br>H 1.152758 -1.280020 -1.506891
H_2O<br>$\tilde{\nu}$: 1619.7, 3868.5, 3971.5<br>B: 833.6235141 431.1572925 284.1779820	O 0.000000 0.000000 0.116391<br>H 0.000000 0.762576 -0.465563<br>H 0.000000 -0.762576 -0.465563
SP_{CO}<br><br>$\tilde{\nu}$: -256.5, 35.7, 182.8, 201.2, 224.2, 237.2, 287.7, 377.3, 387.2, 430.1, 446.7, 708.4, 817.5, 962.3, 999.0, 1044.3, 1072.3, 1105.8, 1360.4, 1367.3, 1415.0, 1419.2, 1477.2, 1484.0, 1492.5, 1504.8, 1653.6, 2028.5, 3034.2, 3037.6, 3099.9, 3103.7, 3141.0, 3147.3, 3543.2, 3643.4<br><br>B: 4.5700000 0.0036324 2.5482349	C -1.352112 -1.032187 -0.599595<br>O 2.188066 -0.096138 -0.026529<br>C 1.342217 -0.269902 -0.782322<br>C -0.536517 0.020154 0.080459<br>N -0.378144 -0.152806 1.450094<br>C -0.755013 1.439221 -0.344686<br>H -1.026029 -2.032118 -0.308912<br>H -1.249455 -0.943402 -1.679484<br>H -2.409601 -0.929411 -0.337385<br>H 0.268027 0.492780 1.883468<br>H -0.189500 -1.102168 1.738834<br>H -0.022142 2.104242 0.115720<br>H -1.752671 1.777112 -0.047268<br>H -0.667596 1.527995 -1.426536
$(CH_3)_2(NH_2)\dot{C}$	C 0.201439 0.092794 0.000000<br>C 0.201439 -0.661842 -1.284079<br>H 0.417994 -0.008026 -2.131675

$\tilde{\upsilon}$: 182.2, 182.6, 327.4, 342.9, 374.4, 413.9, 687.1, 812.6, 961.3, 986.9, 1030.5, 1067.9, 1090.8, 1354.1, 1363.1, 1413.7, 1421.3, 1471.3, 1483.5, 1488.0, 1502.8, 1641.5, 2973.5, 2978.8, 3073.2, 3073.4, 3129.6, 3132.3, 3550.4, 3651.7<br><br>B: 8.9500000 0.0030434 8.1894265	H 0.951089 -1.451805 -1.267973<br>H -0.773441 -1.132327 -1.479102<br>C 0.201439 -0.661842 1.284079<br>H 0.951089 -1.451805 1.267973<br>H 0.417994 -0.008026 2.131675<br>H -0.773441 -1.132327 1.479102<br>N -0.557631 1.271394 0.000000<br>H -0.456885 1.834946 0.831590<br>H -0.456885 1.834946 -0.831590
CO<br>$\tilde{\upsilon}$: 2272.2<br>B: 0.0000000 58.6165459 58.6165459	C 0.000000 0.000000 -0.640794<br>O 0.000000 0.000000 0.480595

Table S14 Quantum chemistry results for the $(CH_3)_2C(\dot{N}H)CHO$ dissociation. Electronic energies of reactants, intermediates and products (/Hartree), and relative energies including Zero Point Energies, $\Delta E_{v=0}$ (/kJ mol^{-1}), of stationary points on the potential energy surface.

	M06-2X/aug-cc-pVTZ			CCSD(T*)/ aug-cc-pVTZ	
Species	E_{Elec}	E_{ZPE}	$\Delta E_{v=0}$	E_{Elec}	$\Delta E_{v=0}$
$(CH_3)_2C(\dot{N}H)CHO$	-287.123149	0.116005	0	-286.794242	0.0
SP-CH3	-287.080508	0.112610	103.0	-286.752389	101.0
SP-CHO	-287.097307	0.113879	62.3	-286.767701	64.1

Table S14, continued: Vibrational frequencies (/cm^{-1}), Rotational constants (B /GHZ) and Cartesian Coordinates (/Å) of the species listed above. Results from M06-2X/aug-cc-pVTZ calculations.

$(CH_3)_2(CHO)C\dot{N}H$<br><br>T_1 = 0.01444, D_1 = 0.0460<br>$\tilde{\upsilon}$: 70.9, 211.4, 249.6, 257.6, 304.5, 331.8, 347.1, 400.6, 428.1, 631.7, 826.5, 891.8, 946.2, 965.2, 979.2, 1039.9, 1147.0, 1172.9, 1281.7, 1332.9, 1383.8, 1403.1, 1421.3, 1477.9, 1487.5, 1499.2, 1513.0, 1859.0, 2943.6, 3060.4, 3064.5, 3137.4, 3139.5, 3153.0, 3154.6, 3406.1<br><br>B: 4.8700000 0.0033264 2.8855105	C 0.335370 0.004518 0.089887<br>C 0.893357 -1.262159 -0.591651<br>C 1.281592 1.182971 -0.138366<br>N 0.218264 -0.267915 1.496604<br>C -1.004467 0.287206 -0.574618<br>O -2.065523 0.094318 -0.051539<br>H 0.239968 -2.115176 -0.412994<br>H 1.878789 -1.478055 -0.183263<br>H 0.982387 -1.102373 -1.667281<br>H 0.903268 2.080770 0.348847<br>H 1.401733 1.383459 -1.203811<br>H 2.254689 0.945484 0.287522<br>H -0.756612 -0.538903 1.667834<br>H -0.942997 0.670439 -1.612289
SP-CH3<br><br>T_1 = 0.02128, D_1 = 0.08336<br>$\tilde{\upsilon}$: -572.9, 129.9, 149.9, 189.4, 209.8, 233.6, 298.7, 388.1, 408.5, 577.0, 606.6, 649.6, 816.4, 867.5, 940.2, 985.7, 1026.1, 1058.7, 1122.1, 1340.4, 1405.4, 1411.0, 1422.0,	C -0.337299 -0.387361 0.125298<br>C -0.440283 1.806675 0.179224<br>C -1.598021 -0.637609 -0.660429<br>N -0.280733 -0.721395 1.374039<br>C 0.937764 -0.193818 -0.672920<br>O 2.028721 -0.202341 -0.175620<br>H 0.415097 2.034976 0.797084

1429.1, 1480.6, 1493.1, 1523.0, 1844.9, 2960.8, 3068.5, 3108.5, 3133.8, 3166.7, 3268.0, 3287.6, 3428.9<br><br>B: 4.2500000 0.0050379 2.8767705	H -1.411852 1.845137 0.647642<br>H -0.384780 2.096119 -0.861857<br>H -1.627099 -1.684593 -0.966330<br>H -1.653368 -0.020247 -1.556026<br>H -2.465362 -0.446481 -0.033717<br>H 0.688494 -0.635458 1.693817<br>H 0.801263 -0.048285 -1.760960
SP-CHO<br><br>T_1 = 0.01986, D_1 = 0.0690<br>$\tilde{\upsilon}$: -481.4, 108.5, 161.7, 174.5, 218.7, 245.3, 305.1, 393.5, 449.4, 507.9, 725.2, 809.4, 832.1, 947.9, 1005.5, 1073.8, 1081.9, 1127.2, 1148.1, 1361.9, 1407.1, 1417.9, 1466.1, 1478.1, 1481.5, 1497.1, 1561.1, 1912.3, 2922.8, 3059.7, 3063.2, 3127.6, 3134.1, 3156.2, 3160.3, 3464.4<br><br>B: 4.7600000 0.0034001 2.6659173	C -0.646026 0.071021 0.187300<br>C -0.513997 1.489141 -0.312794<br>C -1.487718 -0.852594 -0.657482<br>N -0.461362 -0.270631 1.436529<br>C 1.223772 -0.653486 -0.243580<br>O 2.153557 0.062157 -0.246910<br>H 0.251785 2.027022 0.242930<br>H -1.470203 1.999056 -0.179853<br>H -0.260599 1.512546 -1.371558<br>H -1.376119 -1.879560 -0.315614<br>H -1.231249 -0.778319 -1.713108<br>H -2.536825 -0.572053 -0.541412<br>H -0.018882 0.493731 1.948338<br>H 1.186984 -1.729753 0.009193

EXPERIMENTAL RESULTS

Table S15 Summary of initial experimental conditions of the AMP photo-oxidation experiments.

#	Date	Experiment description	AMP	IPN (rate, total)	NO	NO_2	RH	T
			[ppbv]	[µl min^{-1}; µl]	[ppbv]	[ppbv]	[%]	[K]
1	08.06.2015	product study (high NO_x)	~103	1.2; 200	150	140	1	309
2	09.06.2015	product study (medium NO_x)	~102	0.3; 100	30	10	0.8	294
3	12.06.2015	product study (no NO_x)	~92	0.3; 100	0	0	1.4	303
4	15.06.2015	product study (low NO_x)	~100	0.3 (first 10 mins) + 0.1; 18	10	4	1.5	297
5	17.06.2015	product study (no NO_x)	~86	0.1; continuous	0	0	1.5	297
6	18.06.2015	product study (high NO_x)	~93	0.2; continuous	63	69	1.4	300
7	19.06.2015	product study (high NO_x)	~100	0.2; 37	69	43	1	295

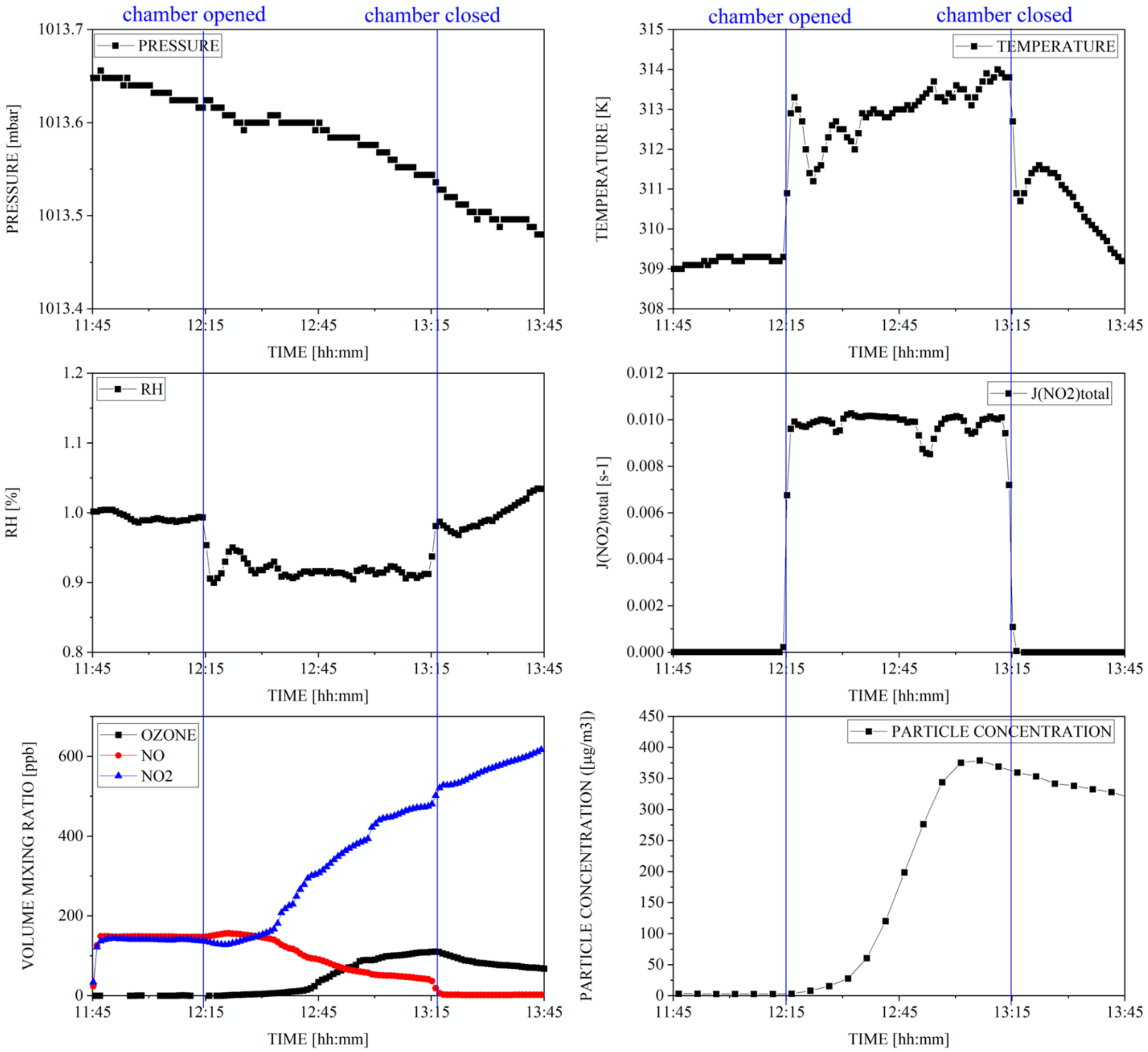

Figure S18 Experimental conditions on photo-oxidation experiments on June 8, 2015.

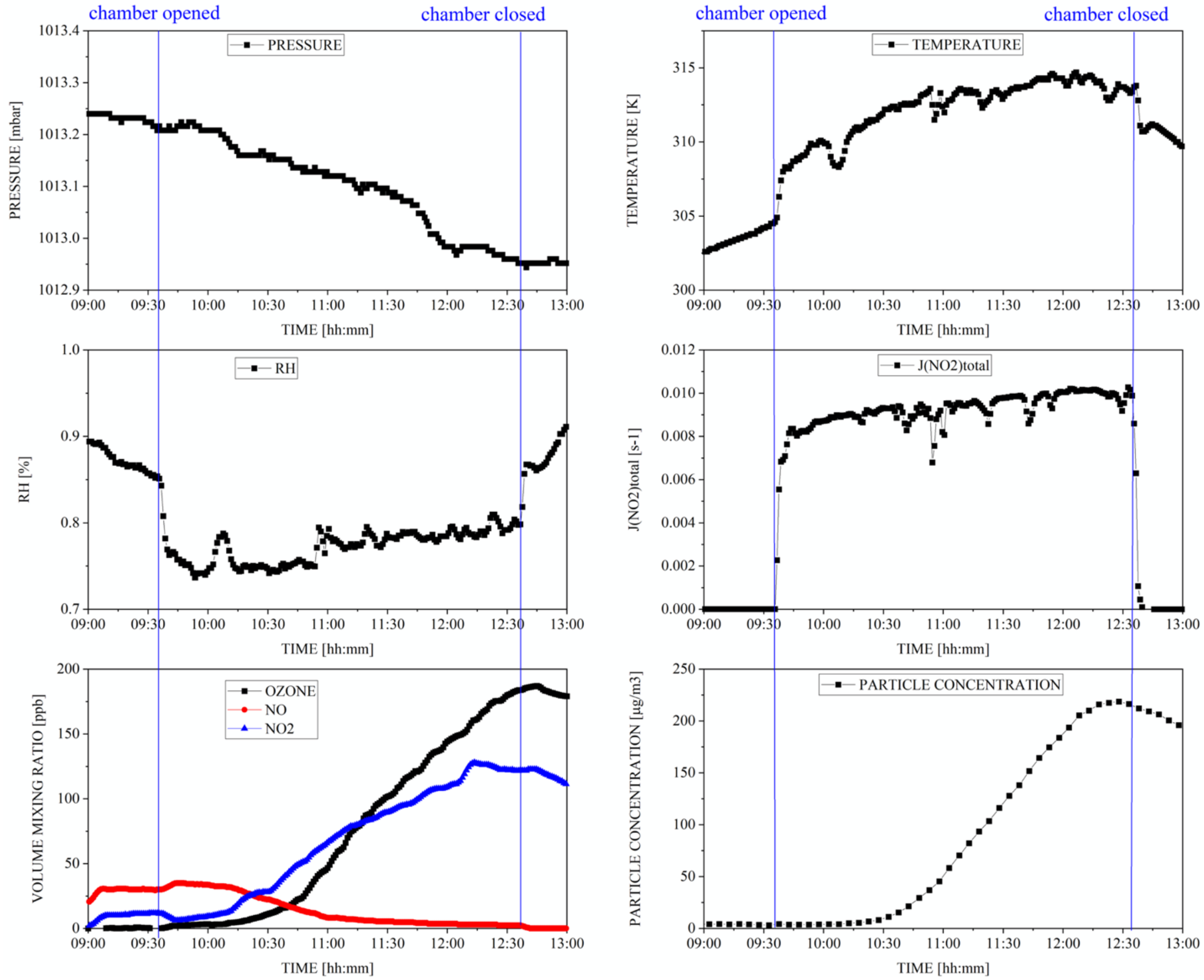

Figure S19 Experimental conditions on photo-oxidation experiments on June 9, 2015.

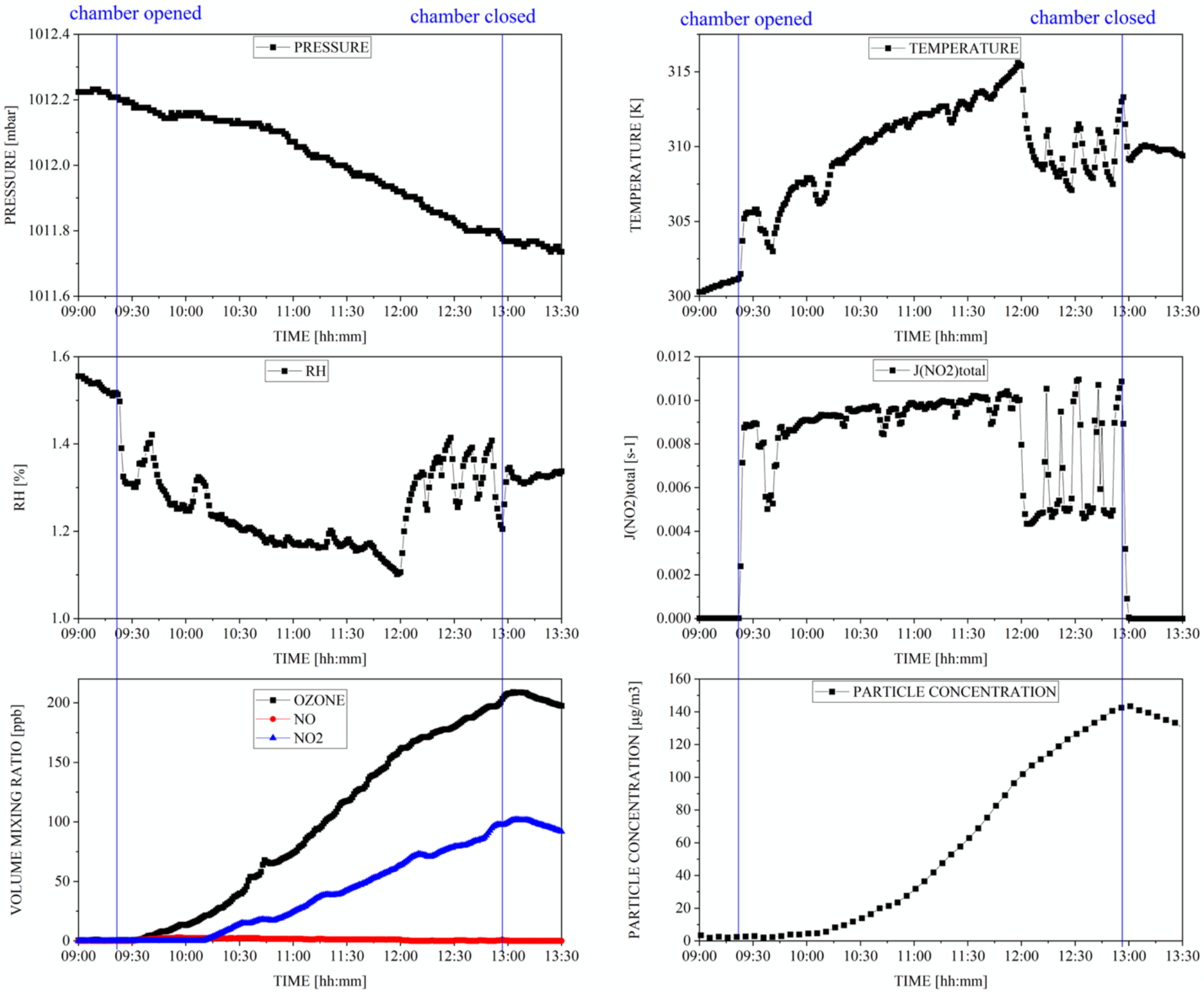

Figure S20 Experimental conditions on photo-oxidation experiments on June 12, 2015.

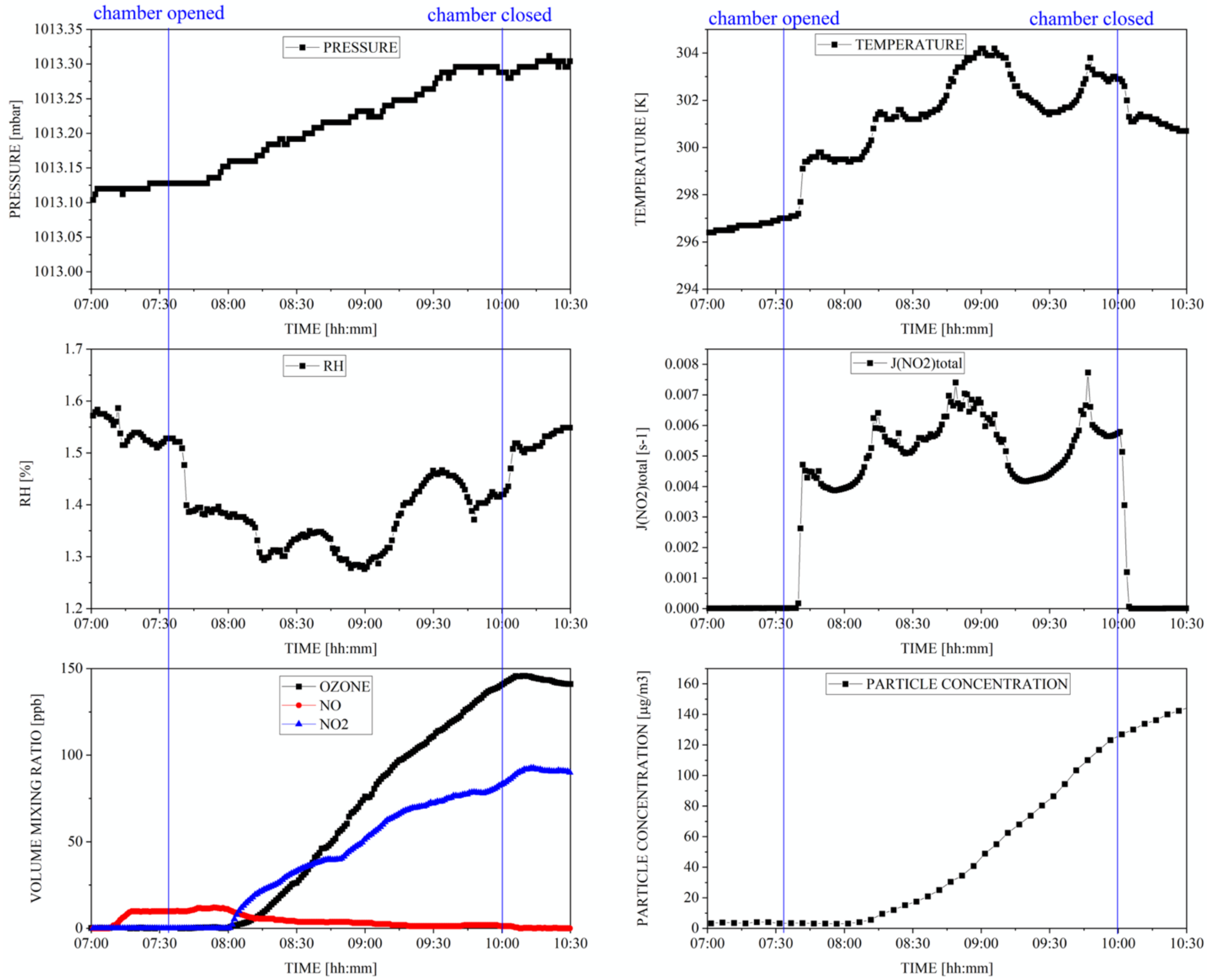

Figure S21 Experimental conditions on photo-oxidation experiments on June 15, 2015.

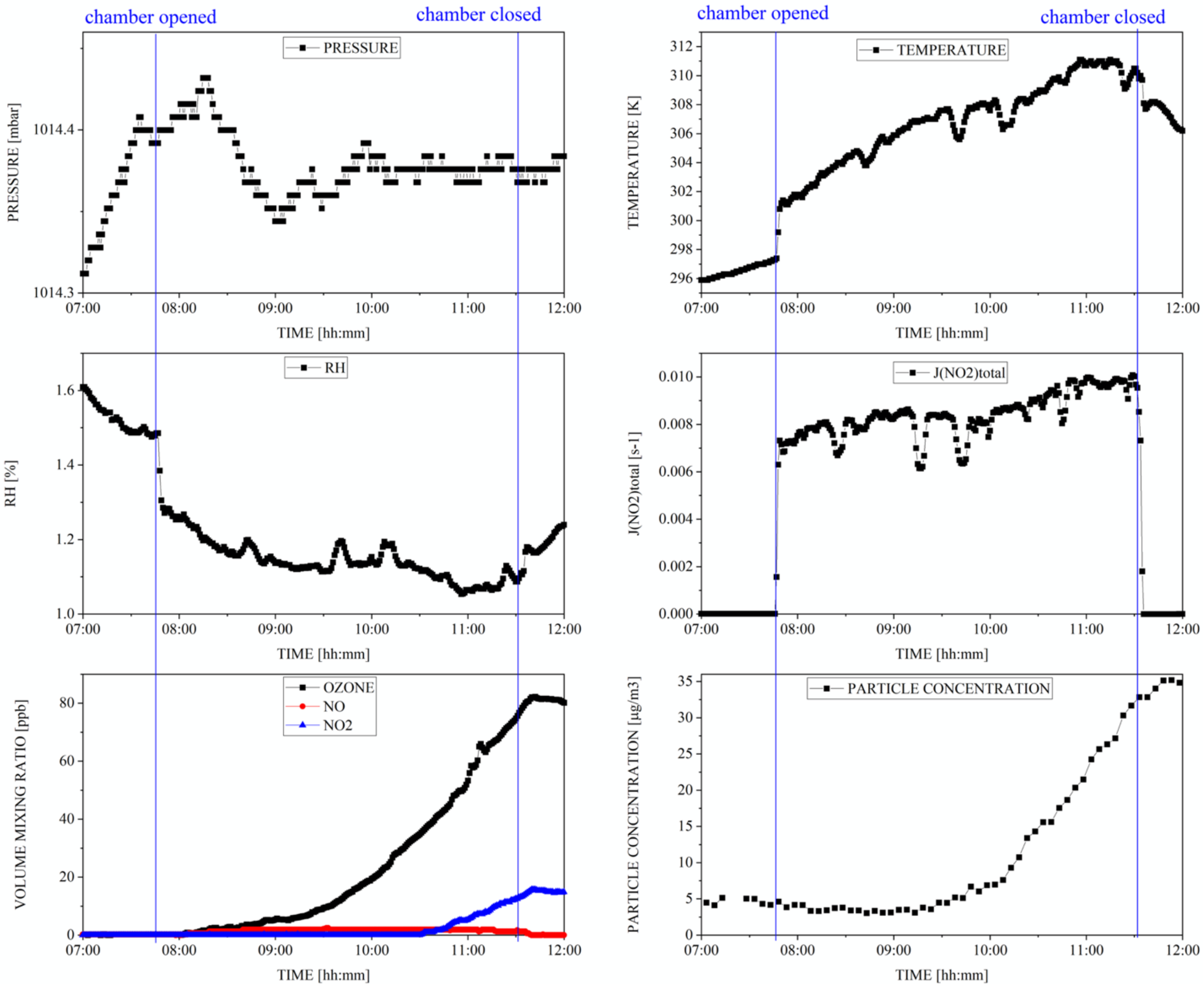

Figure S22 Experimental conditions on photo-oxidation experiments on June 17, 2015.

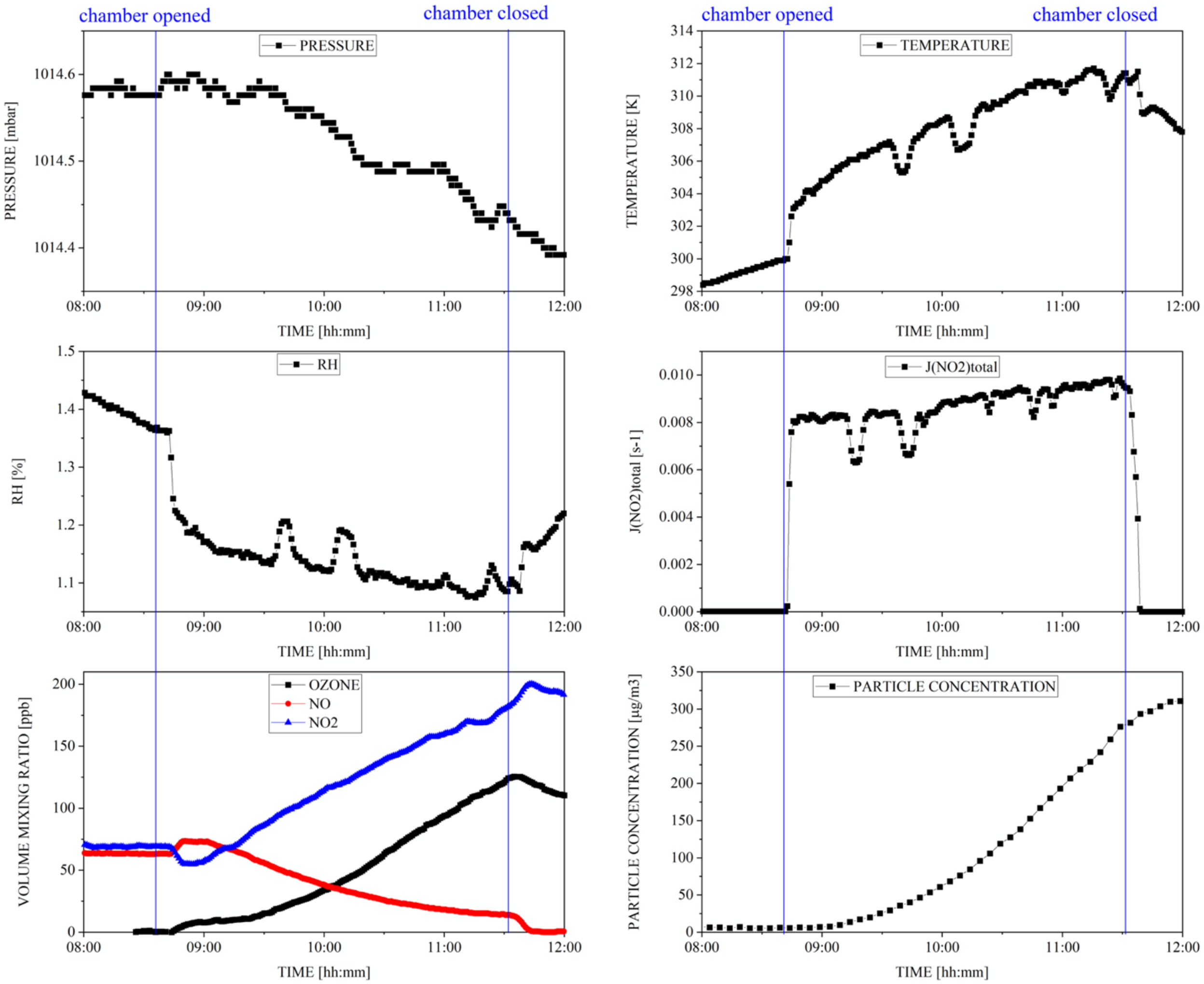

Figure S23 Experimental conditions on photo-oxidation experiments on June 18, 2015.

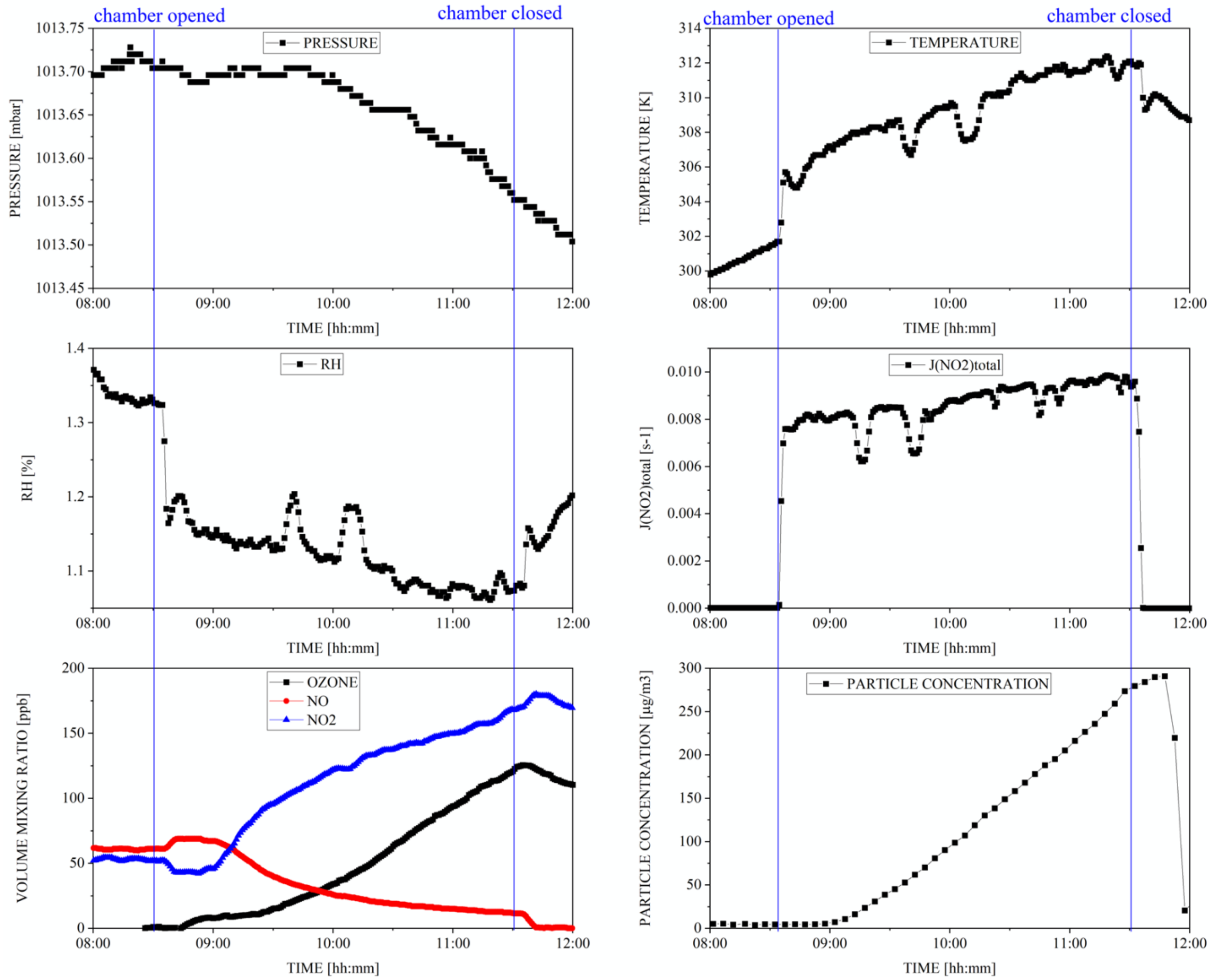

Figure S24 Experimental conditions on photo-oxidation experiments on June 19, 2015.

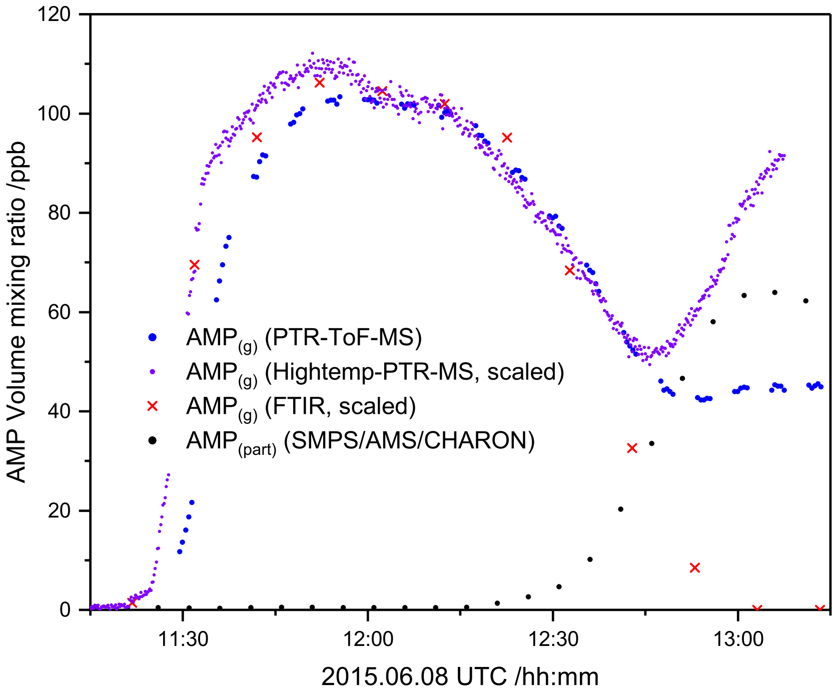

Figure S25 Comparison of the AMP gas phase and particle phase time profiles obtained from FTIR (10 min. average), High-Temperature PTR-QMS (10 s average), PTR-ToF-MS (30 s average) and SMPS/AMS/CHARON (SMPS: 5 min scan time).

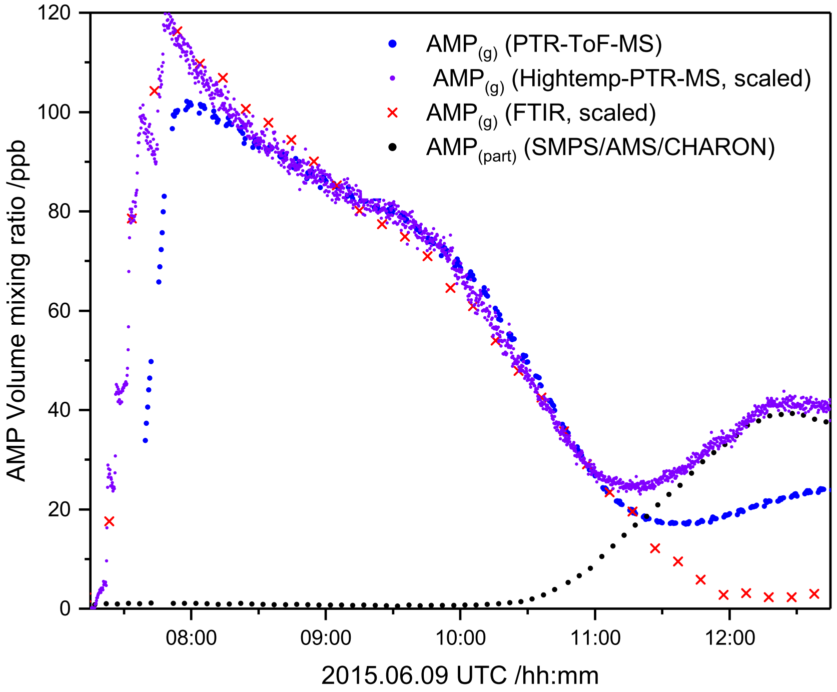

Figure S26 Comparison of the AMP gas phase and particle phase time profiles obtained from FTIR (10 min. average), High-Temperature PTR-QMS (10 s average), PTR-ToF-MS (30 s average) and SMPS/AMS/CHARON (SMPS: 5 min scan time).

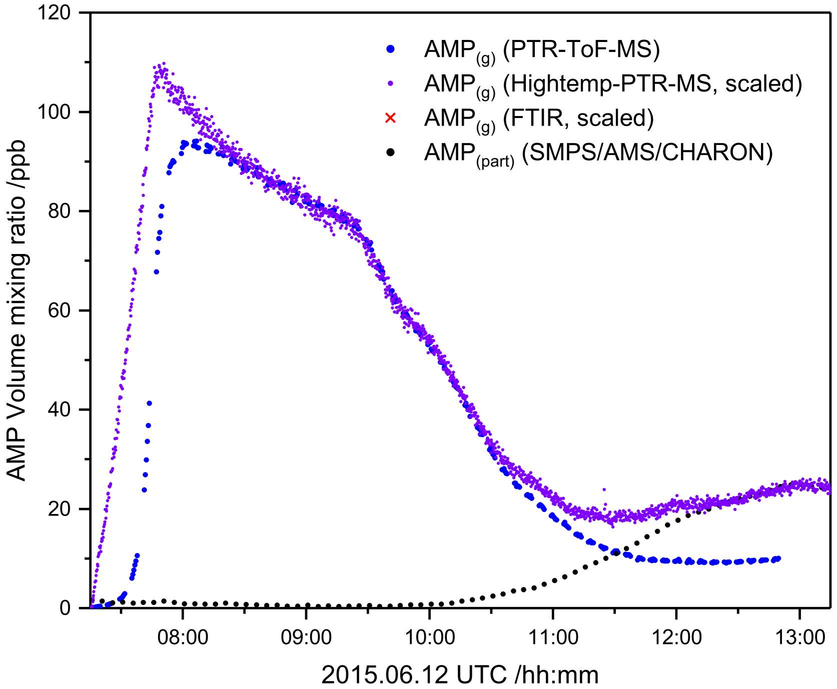

Figure S27 Comparison of the AMP gas phase and particle phase time profiles obtained from FTIR (10 min. average), High-Temperature PTR-QMS (10 s average), PTR-ToF-MS (30 s average) and SMPS/AMS/CHARON (SMPS: 5 min scan time).

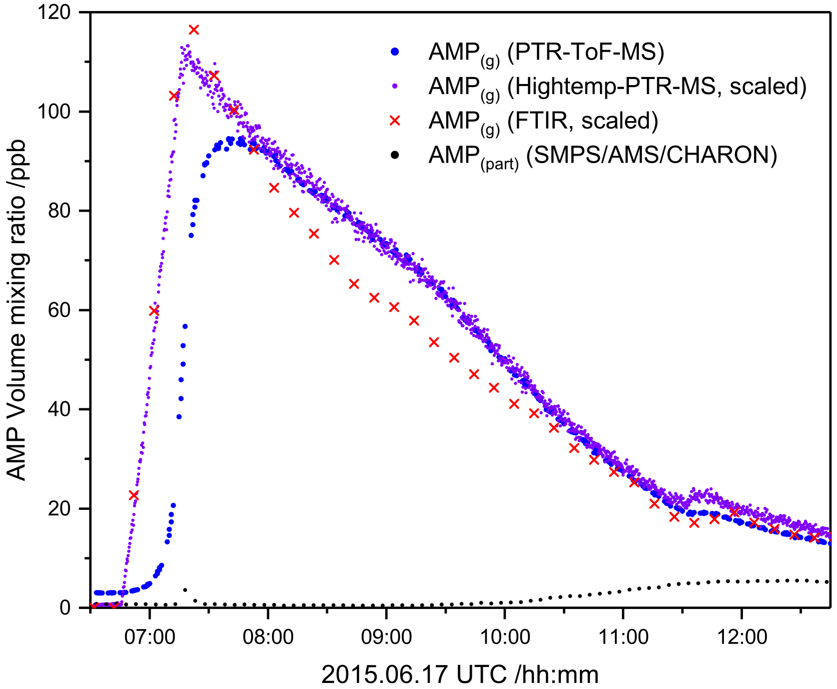

Figure S28 Comparison of the AMP gas phase and particle phase time profiles obtained from FTIR (10 min. average), High-Temperature PTR-QMS (10 s average), PTR-ToF-MS (30 s average) and SMPS/AMS/CHARON (SMPS: 5 min scan time).

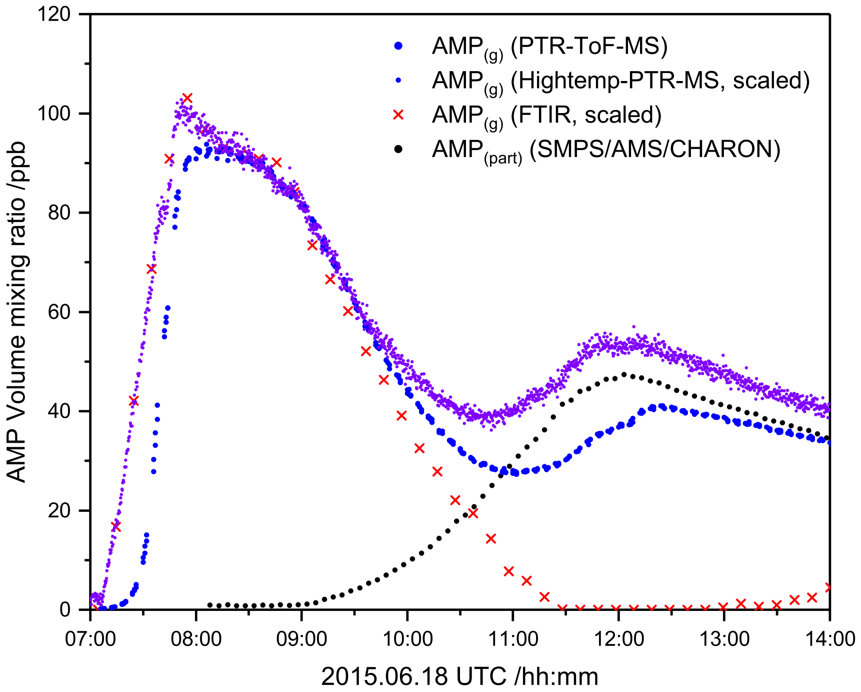

Figure S29 Comparison of the AMP gas phase and particle phase time profiles obtained from FTIR (10 min. average), High-Temperature PTR-QMS (10 s average), PTR-ToF-MS (30 s average) and SMPS/AMS/CHARON (SMPS: 5 min scan time).

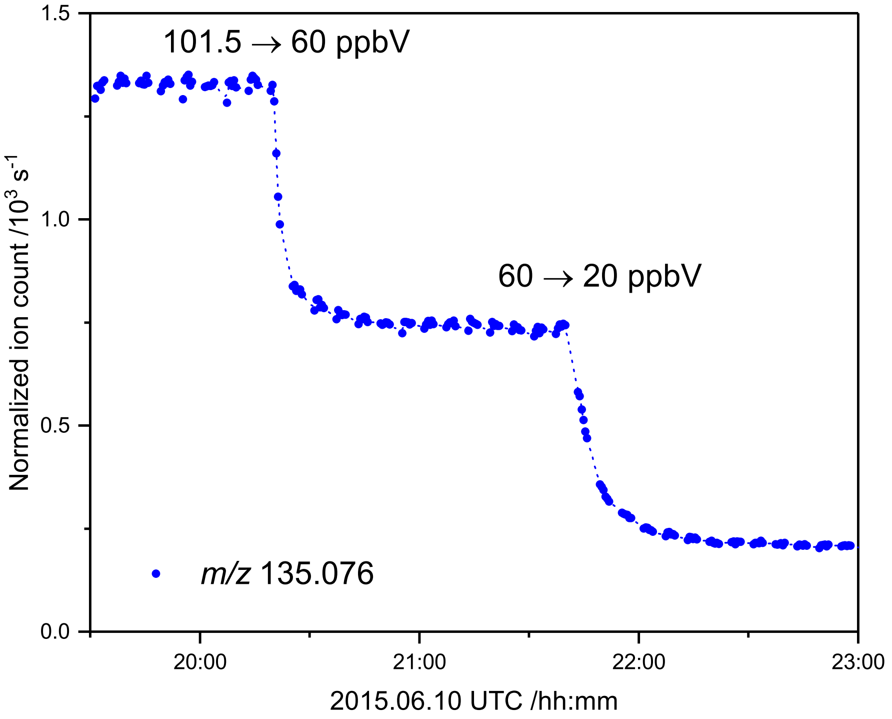

Figure S30 The *m/z* 135.076 time profile during $AMPNO_2$ calibration. The time constant is around 4 min for the 101.5 → 60 ppbV LCU step change and around 10 min for the 60 → 20 ppb LCU step change.

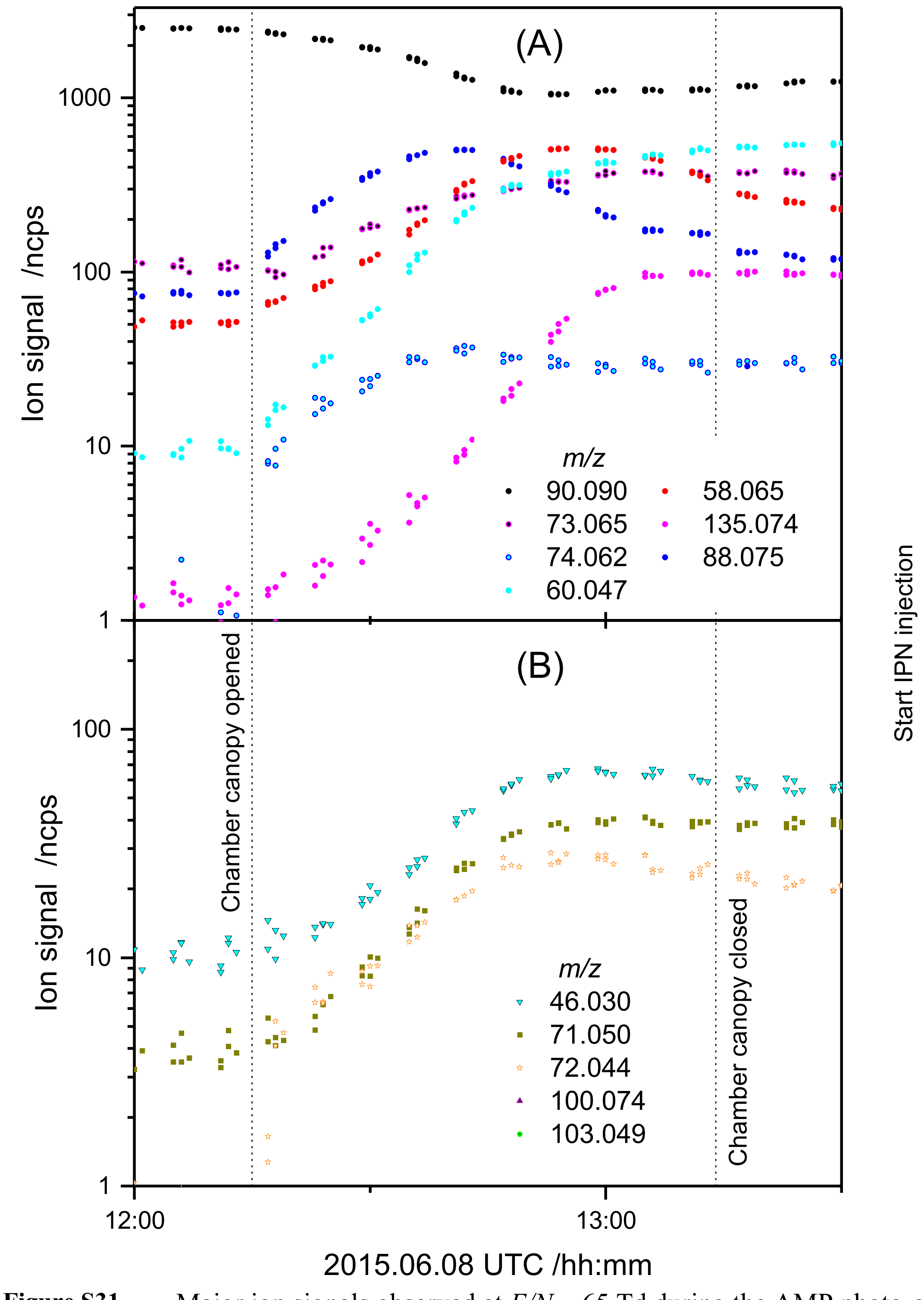

Figure S31 Major ion signals observed at *E/N* = 65 Td during the AMP photo-oxidation experiment on 2015.06.08. (A) Ion signal of AMP and primary products in the AMP + OH reaction. For clarity, the AMP fragment ion signal at *m/z* 72.081 is omitted. (B) Ion signals of secondary products. The *m/z* 74.062 raw signal shown is not corrected for the isotope contribution of *m/z* 73.065..

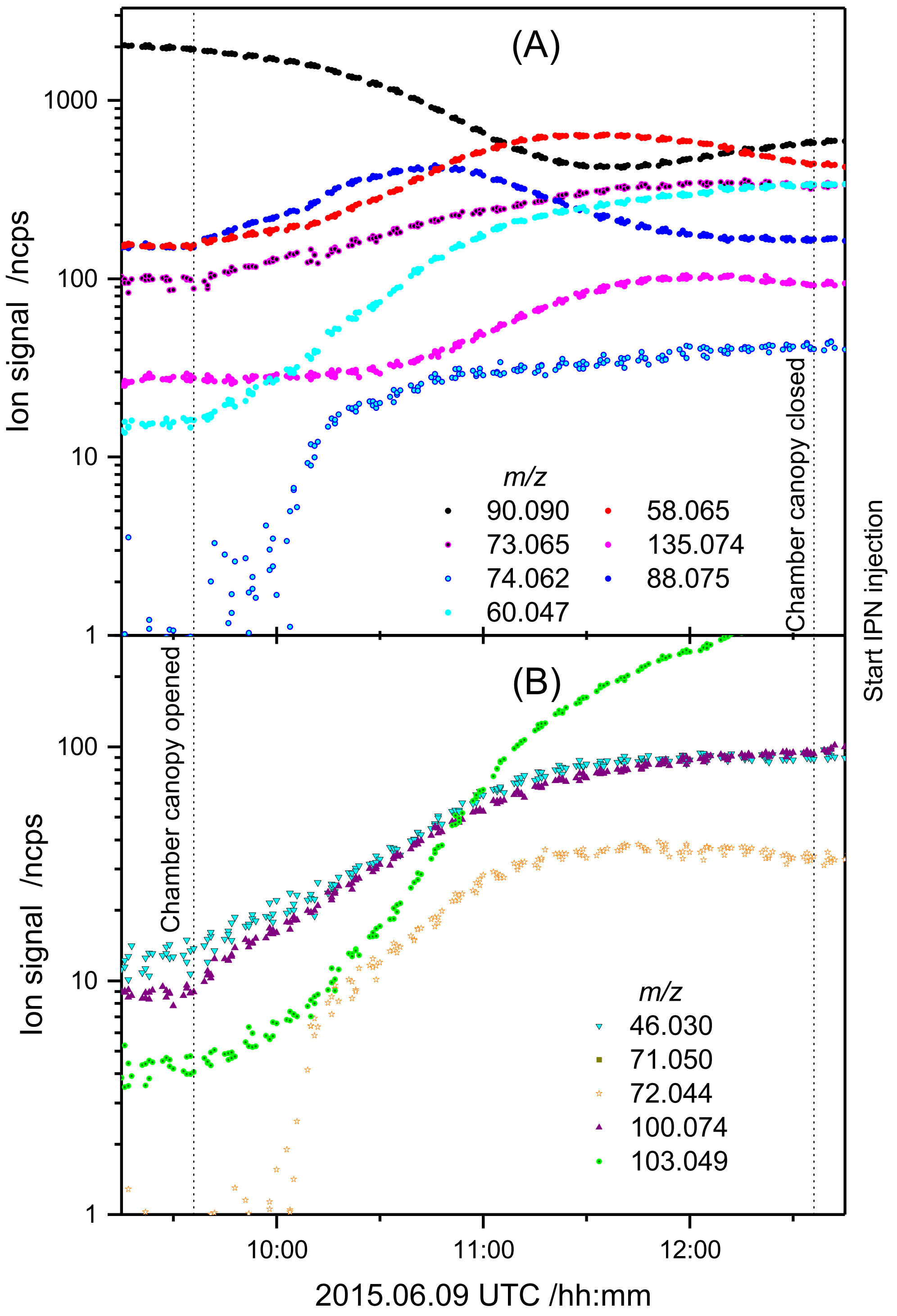

Figure S32 Major ion signals observed at E/N = 65 Td during the AMP photo-oxidation experiment on 2015.06.09. (A) Ion signal of AMP and primary products in the AMP + OH reaction. For clarity, the AMP fragment ion signal at *m/z* 72.081 is omitted. (B) Ion signals of secondary products. The *m/z* 74.062 raw signal shown is not corrected for the isotope contribution of *m/z* 73.065..

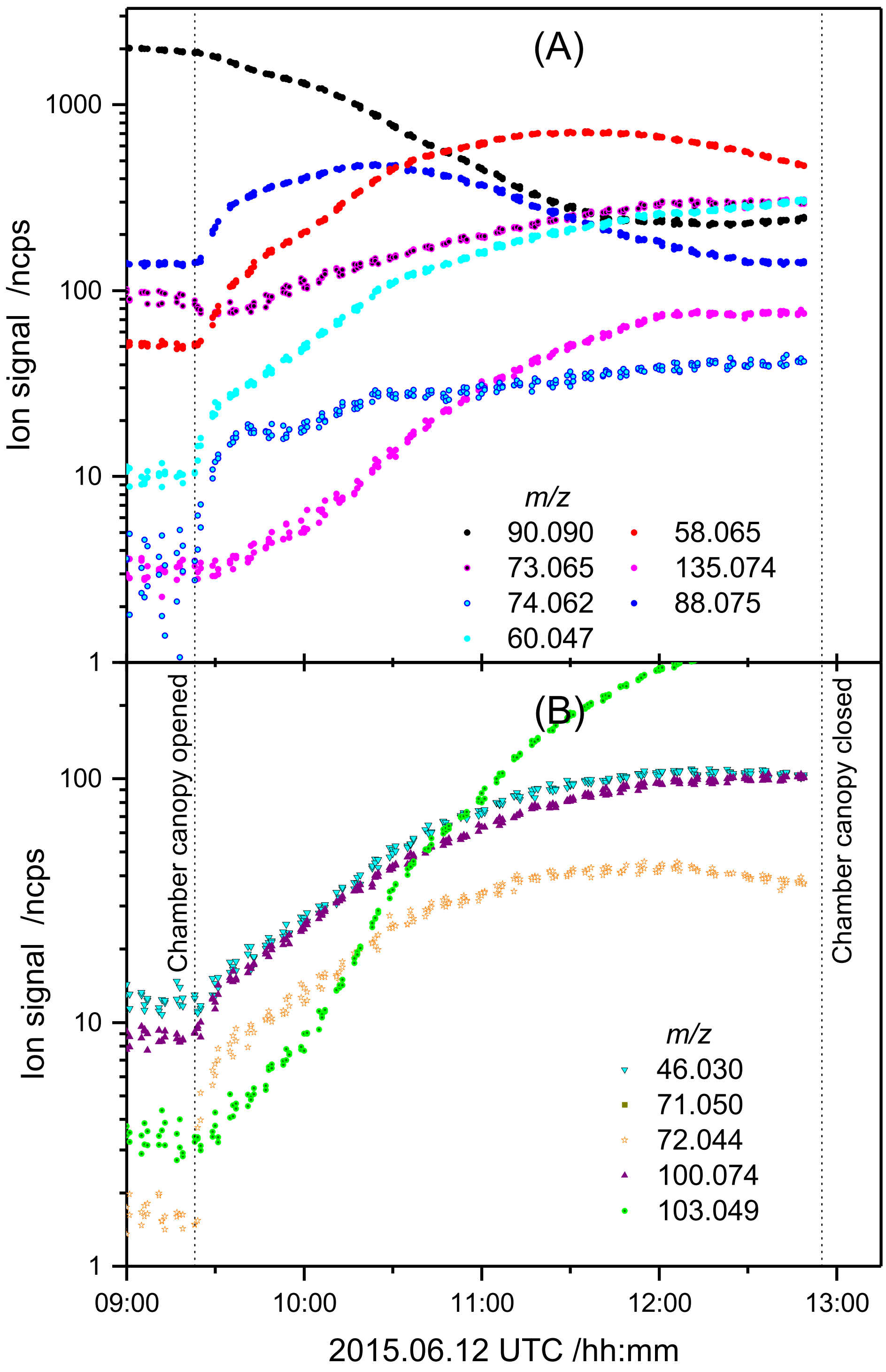

Figure S33 Major ion signals observed at *E/N* = 65 Td during the AMP photo-oxidation experiment on 2015.06.12. (A) Ion signal of AMP and primary products in the AMP + OH reaction. For clarity, the AMP fragment ion signal at *m/z* 72.081 is omitted. (B) Ion signals of secondary products. The *m/z* 74.062 raw signal shown is not corrected for the isotope contribution of *m/z* 73.065..

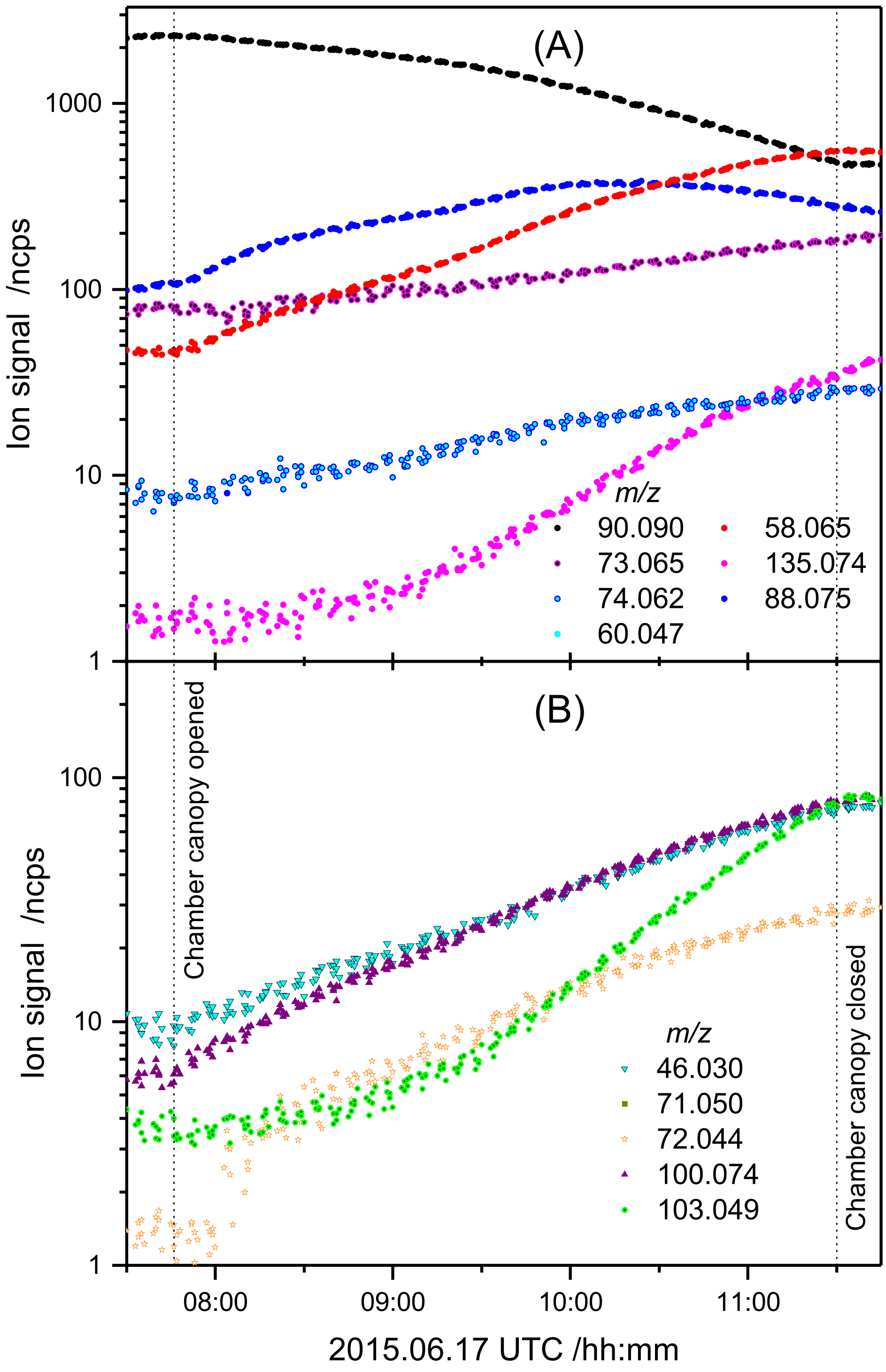

Figure S34 Major ion signals observed at *E/N* = 65 Td during the AMP photo-oxidation experiment on 2015.06.17. (A) Ion signal of AMP and primary products in the AMP + OH reaction. For clarity, the AMP fragment ion signal at *m/z* 72.081 is omitted. (B) Ion signals of secondary products. The *m/z* 74.062 raw signal shown is not corrected for the isotope contribution of *m/z* 73.065..

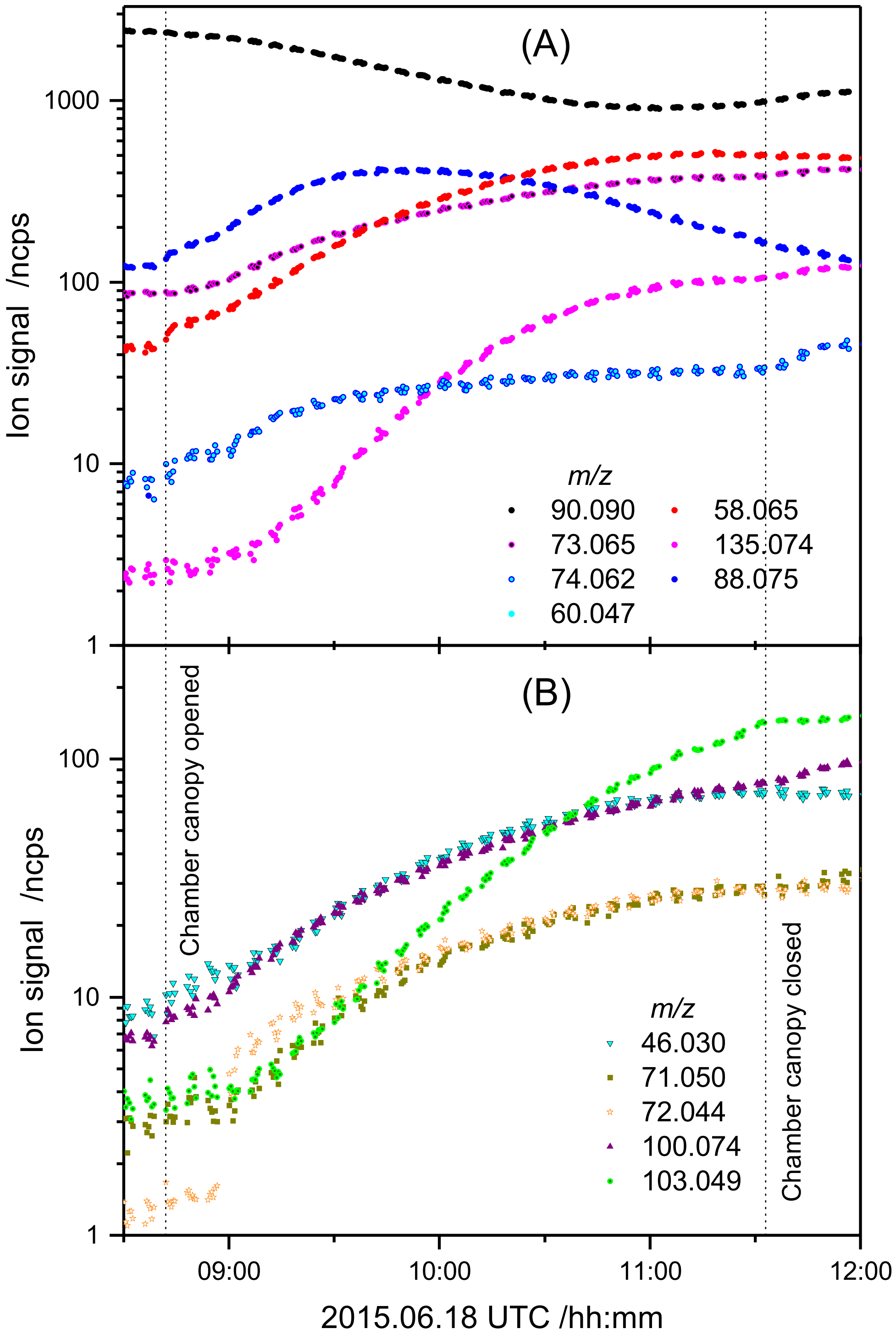

Figure S35 Major ion signals observed at *E/N* = 65 Td during the AMP photo-oxidation experiment on 2015.06.18. (A) Ion signal of AMP and primary products in the AMP + OH reaction. For clarity, the AMP fragment ion signal at *m/z* 72.081 is omitted. (B) Ion signals of secondary products. The *m/z* 74.062 raw signal shown is not corrected for the isotope contribution of *m/z* 73.065..

Scheme S5 Mechanisms for the surface reactions of AMP with formic acid (A), the *intra* molecular deamination reaction of AMP (B), the nitration of propan-2-imine (C), the nitrification and nitric acid esterification of AMP (D), and the nitrification of AMP by the AMP nitric acid ester (E).

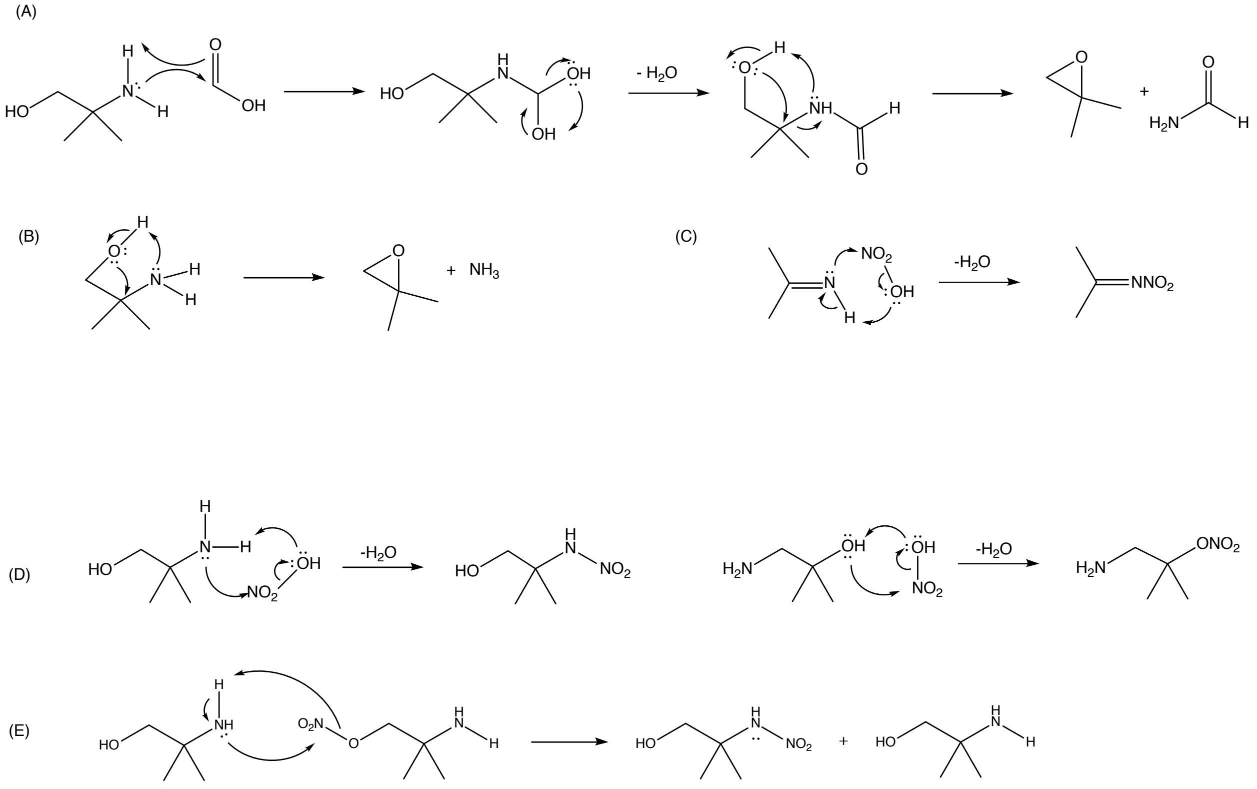

Particle-phase Products

Table S16 $AMPNO_2$, AMP^+ and NO_3^- concentrations determined from aerosol samples collected at the end of each AMP photo-oxidation experiment. The total aerosol concentration in the chamber recorded by the SMPS is also shown. Reported concentrations are raw data that have not been corrected for particle wall loss and chamber dilution.

Date	Sampling time [HH:MM]	$AMPNO_2$ [µg m^{-3}]	AMP^+ [µg m^{-3}]	NO_3^- [µg m^{-3}]	NH_4^+ [µg m^{-3}]	Total organics [µg m^{-3}]	SMPS aerosol [µg m^{-3}]
2015.06.09	14:21 – 15:21	10.4	58.0	32.1			117
				37.3 [a]	3.73 [a]	92.1 [a]	
2015.06.12	13:55 – 14:55	12.4	44.6	26.1			110
				35.7 [a]	3.35 [a]	76.6 [a]	127
2015.06.15	12:25 – 13:00	0.10 [b]	79.9 [b]	31.9 [a]	4.28 [a]	63.5 [a]	137
	15:09 – 16:10	24.8	30.8	17.1			
2015.06.18	13:00 – 13:45	0.50 [b]	136.1 [b]	83.1	7.66	149.5	277
	15:27 – 16:27	18.1	65.6	34.1			161
2015.06.19	11:00 – 11:45	0.55 [b]	146.6 [b]	83.3 [a]	11.6 [a]	168.8 [a]	290
	12:58 – 13:58	26.1	97.9	55.4			

[a] From AMS. [b] From CHARON-PTR-ToF-MS.

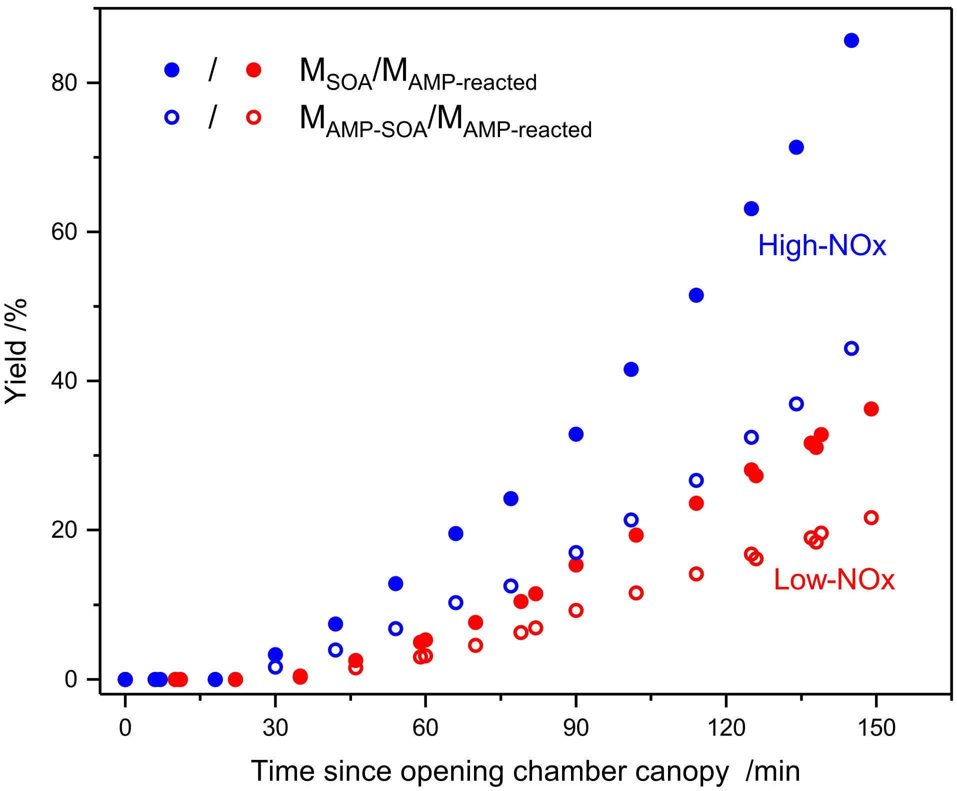

Figure S36 The particle yield (•) and AMP fraction (o) in particles formed under high-NOx (2015.06.18) and low-NOx conditions (2015.06.15).

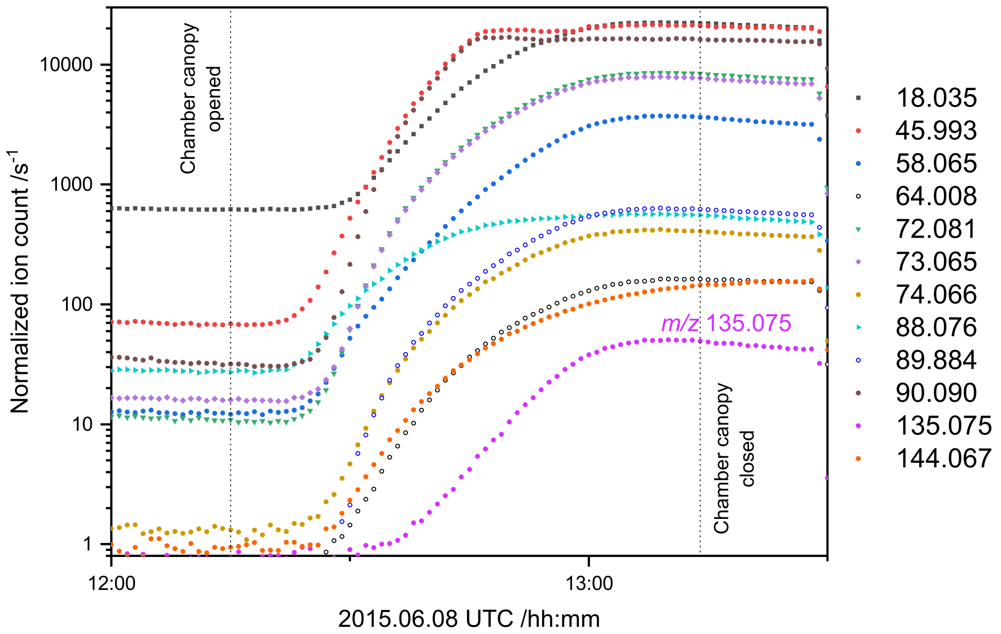

Figure S37 Time evolution of the CHARON-PTR-ToF-MS mass spectrum during the AMP photo-oxidation experiment on 2015.06.08. With the exception of *m/z* 135.075, ion signals with intensity less than 0.5% of the AMP signal m/z 90.090 at 14:00 UTC, and ion signals related to the ion source and to isotopes are excluded.

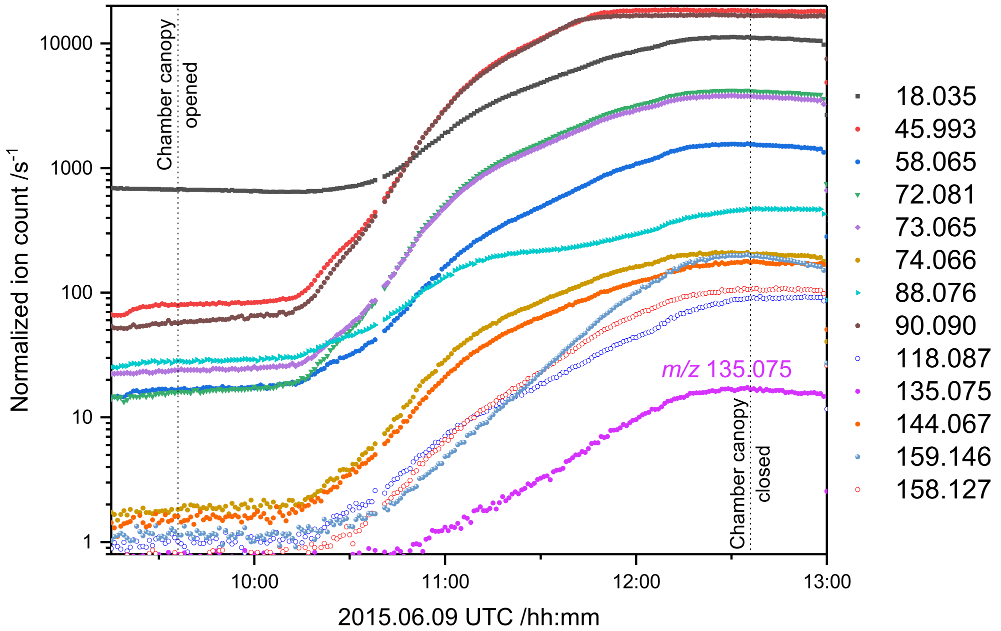

Figure S38 Time evolution of the CHARON-PTR-ToF-MS mass spectrum during the AMP photo-oxidation experiment on 2015.06.09. With the exception of *m/z* 135.075, ion signals with intensity less than 0.5% of the AMP signal m/z 90.090 at 12:00 UTC, and ion signals related to the ion source and to isotopes are excluded.

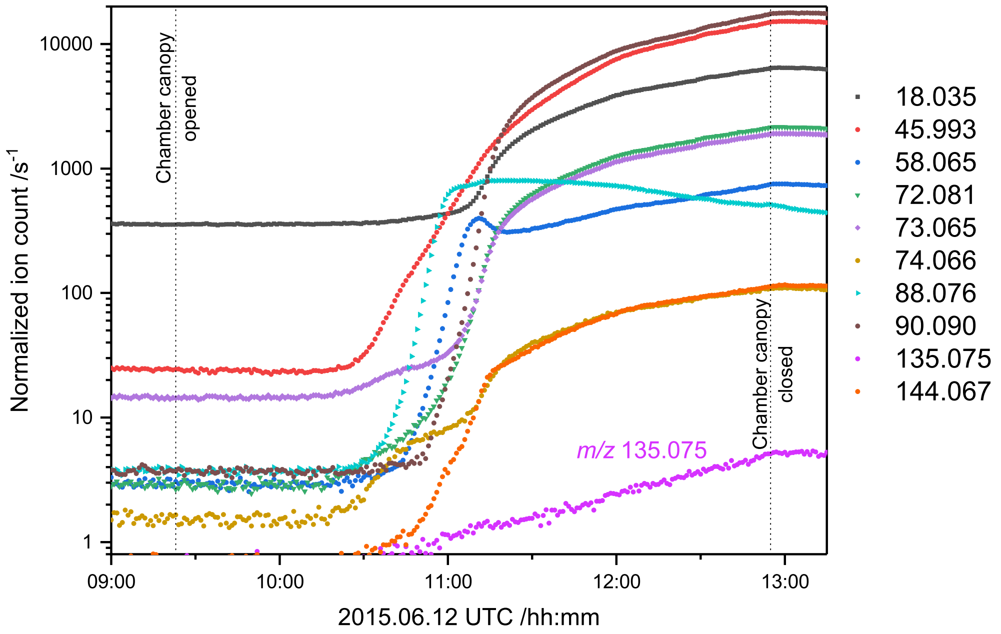

Figure S39 Time evolution of the CHARON-PTR-ToF-MS mass spectrum during the AMP photo-oxidation experiment on 2015.06.12. With the exception of *m/z* 135.075, ion signals with intensity less than 0.5% of the AMP signal m/z 90.090 at 13:00 UTC, and ion signals related to the ion source and to isotopes are excluded.

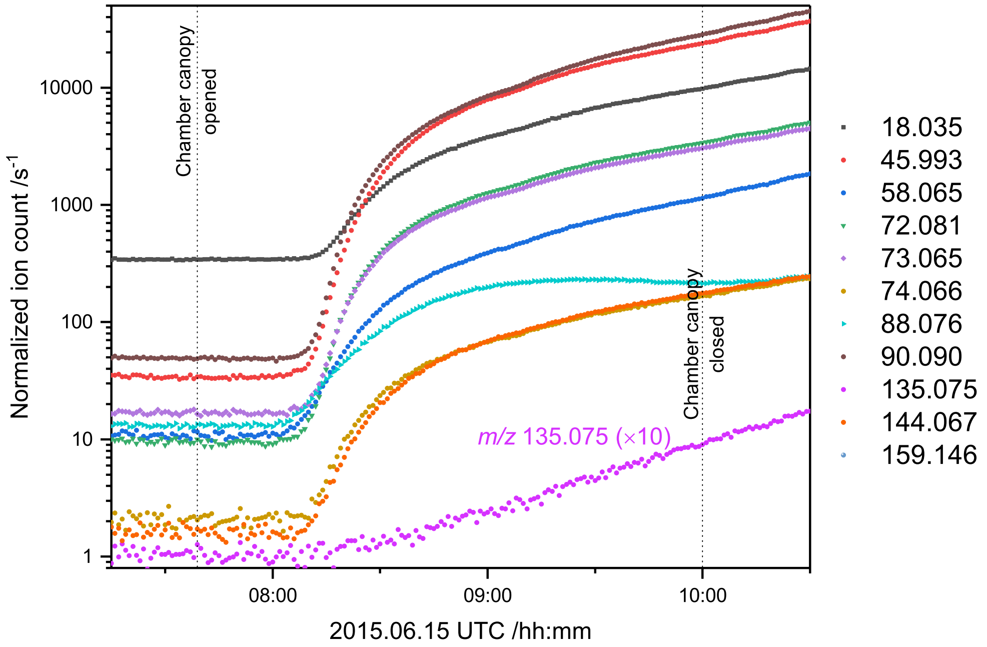

Figure S40 Time evolution of the CHARON-PTR-ToF-MS mass spectrum during the AMP photo-oxidation experiment on 2015.06.15. With the exception of *m/z* 135.075, ion signals with intensity less than 0.5% of the AMP signal m/z 90.090 at 11:00 UTC, and ion signals related to the ion source and to isotopes are excluded.

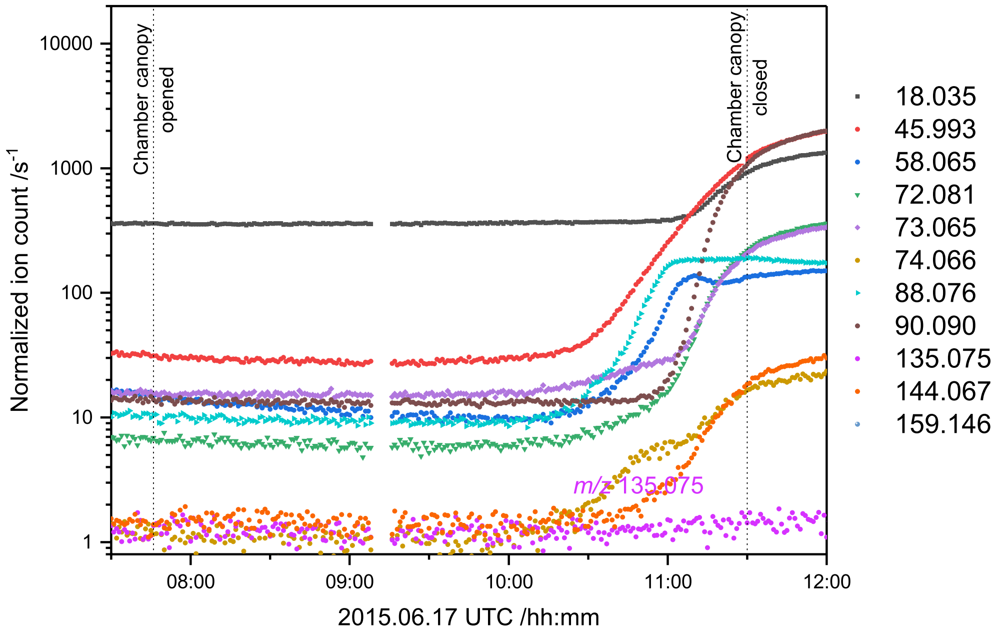

Figure S41 Time evolution of the CHARON-PTR-ToF-MS mass spectrum during the AMP photo-oxidation experiment on 2015.06.17. With the exception of *m/z* 135.075, ion signals with intensity less than 0.5% of the AMP signal m/z 90.090 at 11:00 UTC, and ion signals related to the ion source and to isotopes are excluded.

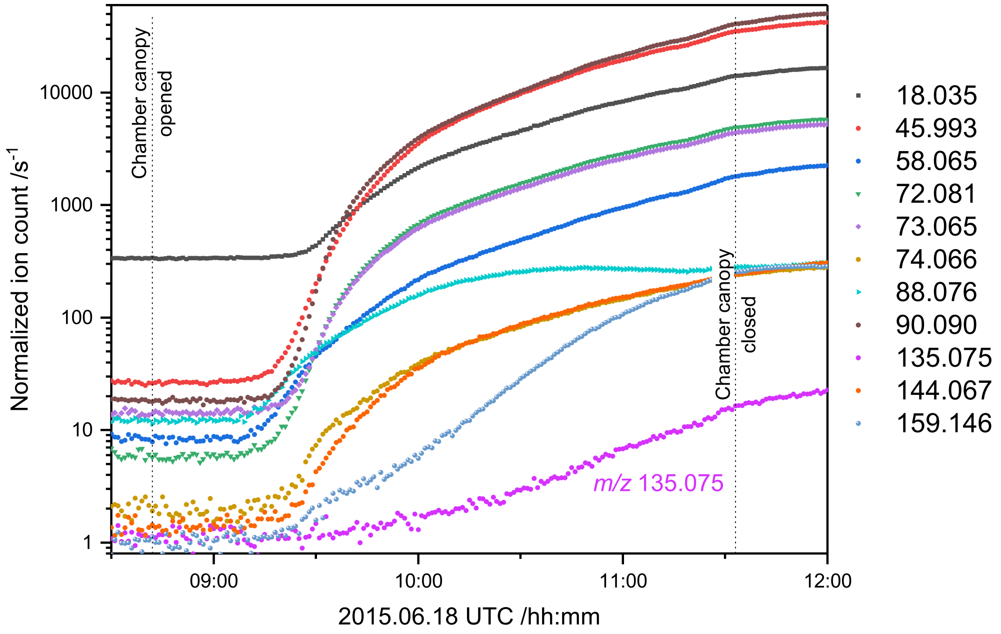

Figure S42 Time evolution of the CHARON-PTR-ToF-MS mass spectrum during the AMP photo-oxidation experiment on 2015.06.18. With the exception of *m/z* 135.075, ion signals with intensity less than 0.5% of the AMP signal m/z 90.090 at 11:00 UTC, and ion signals related to the ion source and to isotopes are excluded.

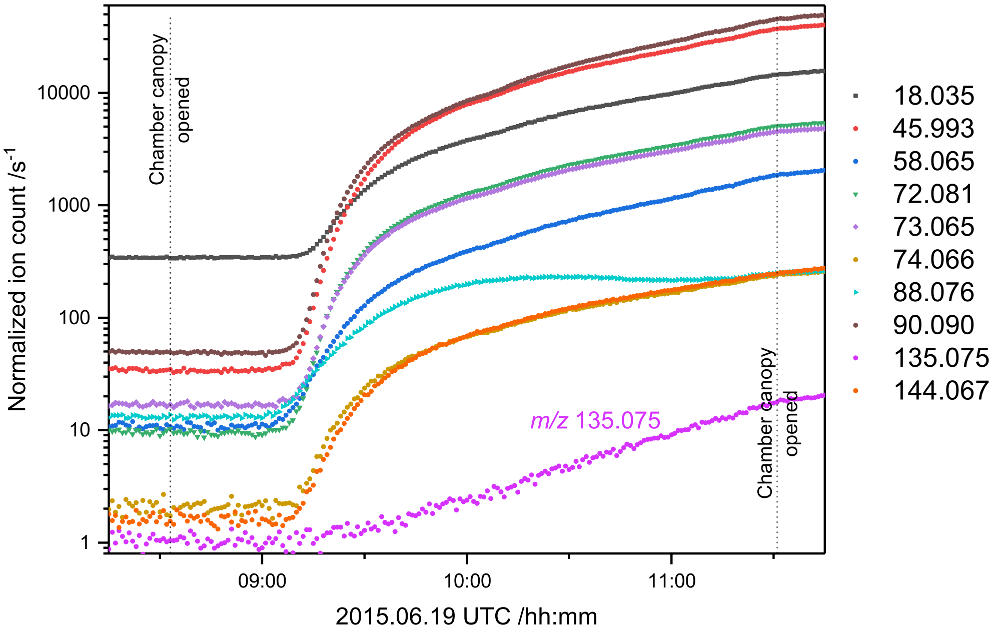

Figure S43 Time evolution of the CHARON-PTR-ToF-MS mass spectrum during the AMP photo-oxidation experiment on 2015.06.19. With the exception of *m/z* 135.075, ion signals with intensity less than 0.5% of the AMP signal m/z 90.090 at 11:00 UTC, and ion signals related to the ion source and to isotopes are excluded.

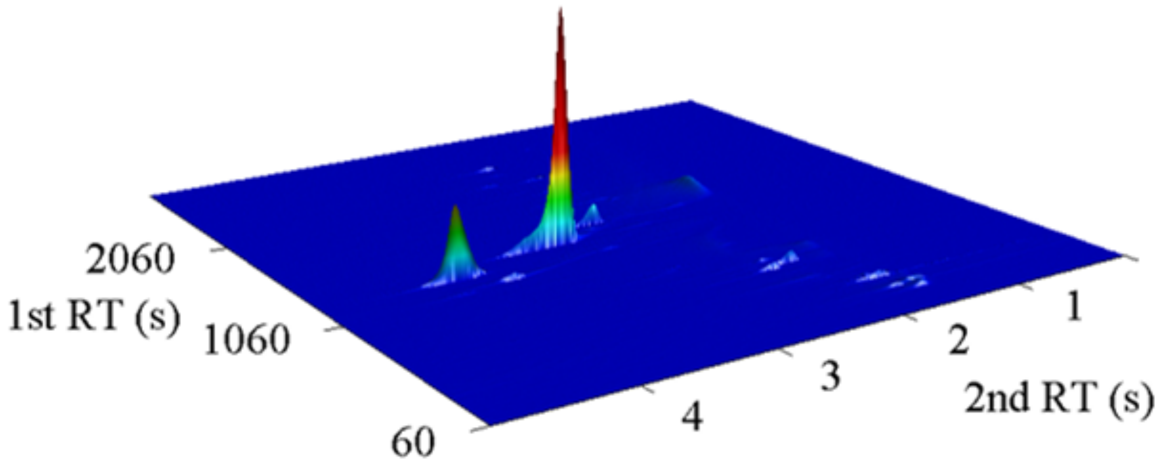

Figure S44 GC×GC-NCD chromatogram to show the detection of $AMPNO_2$ in an aerosol sample collected at the end of the AMP photo-oxidation experiment on 2015.06.09.

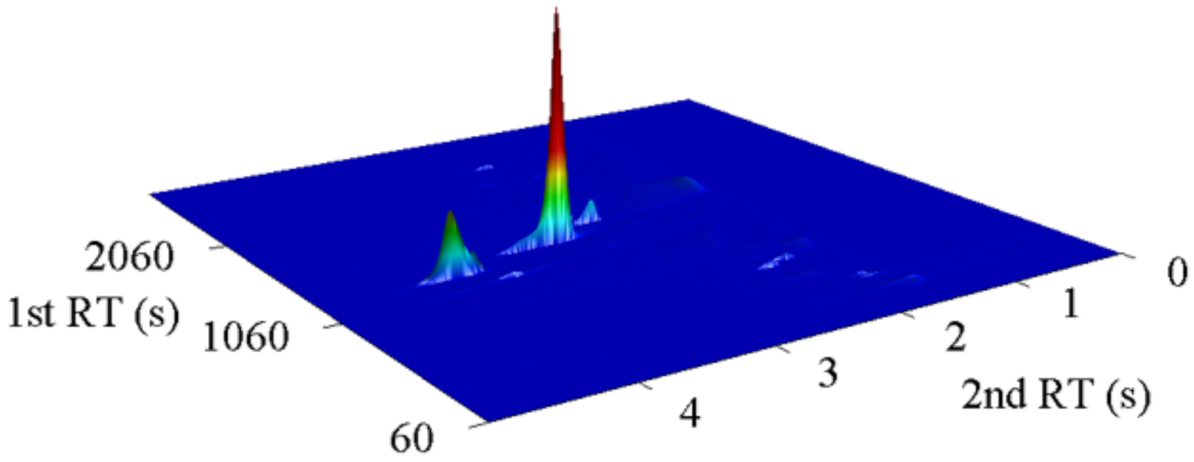

Figure S45 GC×GC-NCD chromatogram to show the detection of $AMPNO_2$ in an aerosol sample collected at the end of the AMP photo-oxidation experiment on 2015.06.12.

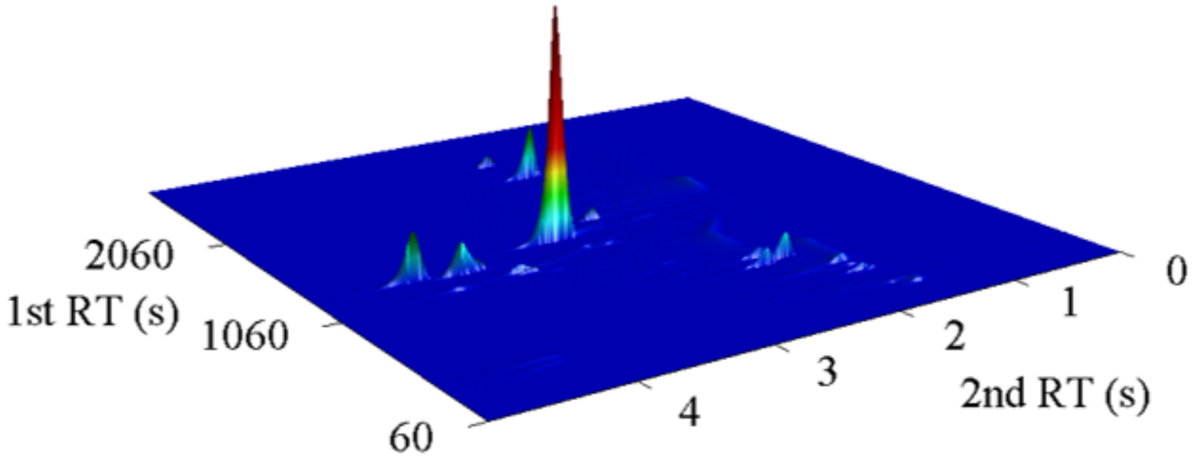

Figure S46 GC×GC-NCD chromatogram to show the detection of $AMPNO_2$ in an aerosol sample collected at the end of the AMP photo-oxidation experiment on 2015.06.18.

Table S17 Duration of the AMP photo-oxidation experiments (min), and the per cent AMP transfer to the particle phase (AMP_{part}), AMP wall loss (AMP_{wall}) and AMP undergoing gas phase photo-oxidation (AMP_{react}) in the experiments.

Date	Duration	AMP_{part}	AMP_{wall}	AMP_{react}
2015.06.08	59	57	7	37
2015.06.09	180	41	21	38
2015.06.12	212	29	15	53
2015.06.15	143	21	18	65
2015.06.17	224	5	51	44
2015.06.18	171	47	30	23

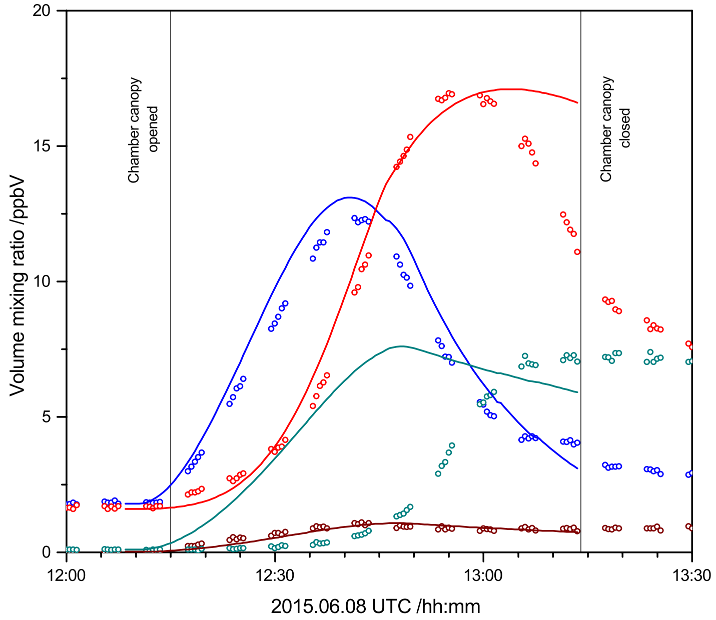

Figure S47 Observed and modelled temporal profiles of products in the OH-initiated AMP photo-oxidation experiment on 2015.06.08. $(CH_3)_2(CHO)CNH_2$ (blue colour), $(CH_3)_2C{=}NH$ (red colour), $CH_3(CH_2OH)C{=}NH$ (wine colour), $(CH_3)_2(CH_2OH)NHNO_2$ (dark cyan colour).

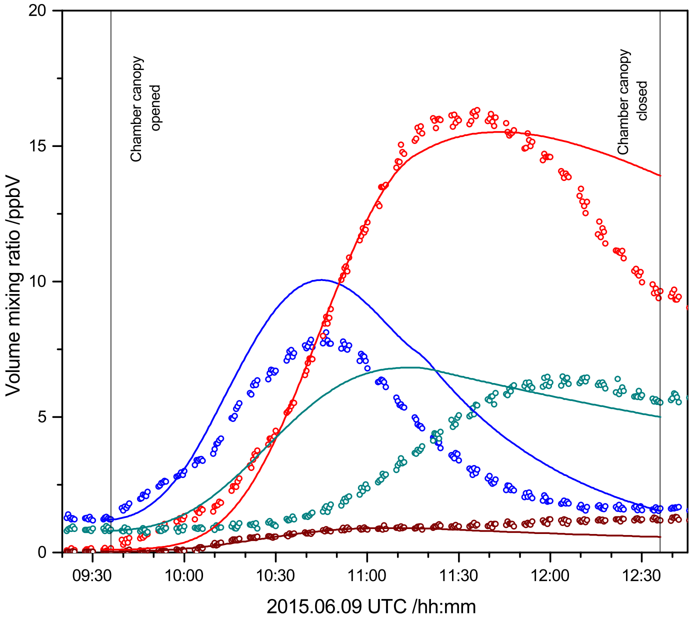

Figure S48 Observed and modelled temporal profiles of products in the OH-initiated AMP photo-oxidation experiment on 2015.06.09. $(CH_3)_2(CHO)CNH_2$ (blue colour), $(CH_3)_2C{=}NH$ (red colour), $CH_3(CH_2OH)C{=}NH$ (wine colour), $(CH_3)_2(CH_2OH)NHNO_2$ (dark cyan colour).

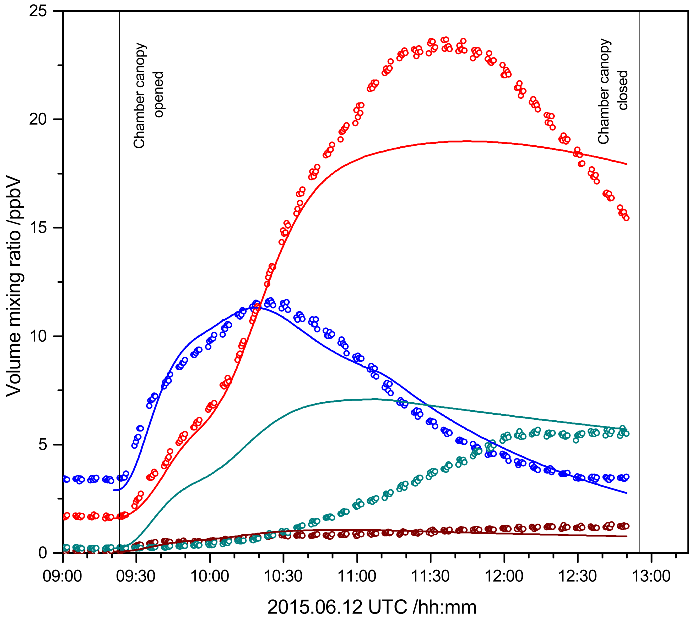

Figure S49 Observed and modelled temporal profiles of products in the OH-initiated AMP photo-oxidation experiment on 2015.06.12. $(CH_3)_2(CHO)CNH_2$ (blue colour), $(CH_3)_2C{=}NH$ (red colour), $CH_3(CH_2OH)C{=}NH$ (wine colour), $(CH_3)_2(CH_2OH)NHNO_2$ (dark cyan colour).

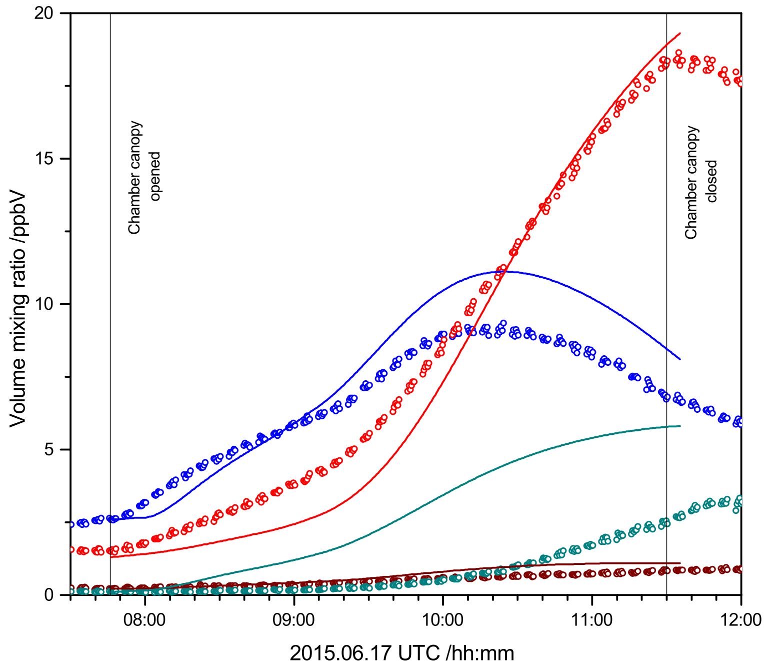

Figure S50 Observed and modelled temporal profiles of products in the OH-initiated AMP photo-oxidation experiment on 2015.06.17. $(CH_3)_2(CHO)CNH_2$ (blue colour), $(CH_3)_2C{=}NH$ (red colour), $CH_3(CH_2OH)C{=}NH$ (wine colour), $(CH_3)_2(CH_2OH)NHNO_2$ (dark cyan colour).

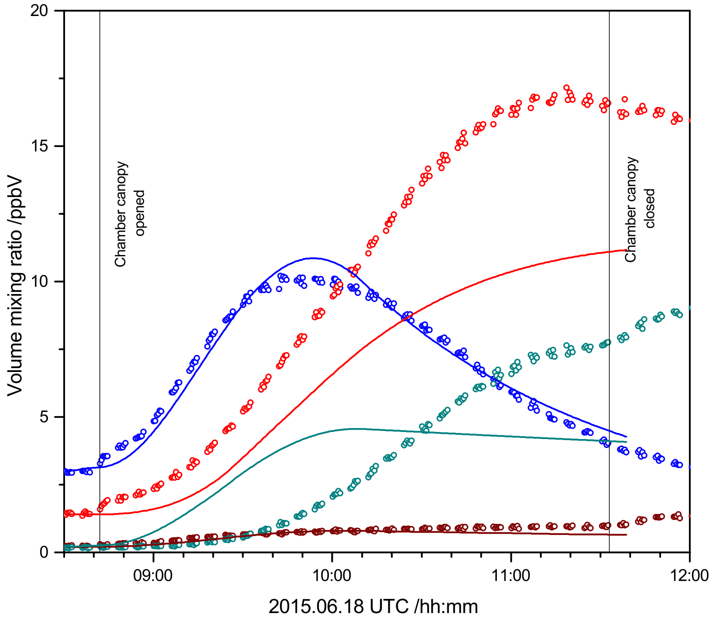

Figure S51 Observed and modelled temporal profiles of products in the OH-initiated AMP photo-oxidation experiment on 2015.06.18. $(CH_3)_2(CHO)CNH_2$ (blue colour), $(CH_3)_2C{=}NH$ (red colour), $CH_3(CH_2OH)C{=}NH$ (wine colour), $(CH_3)_2(CH_2OH)NHNO_2$ (dark cyan colour).

REFERENCES

(1) Becker, K. H., *The European Photoreactor EUPHORE: Design and Technical Development of the European Photoreactor and First Experimental Results: Final Report of the EC-Project: Contract EV5V-CT92-0059: Funding Period, January 1993-December 1995*. 1996.

(2) Griffith, D. W. T. Synthetic calibration and quantitative analysis of gas-phase FT-IR spectra. *Appl. Spectrosc.* **1996,** *50*, 59-70.

(3) Rothman, L. S.; Gordon, I. E.; Barbe, A.; ChrisBenner, D.; Bernath, P. F.; Birk, M.; Boudon, V.; Brown, L. R.; Campargue, A.; Champion, J.-P., et al. The HITRAN 2008 molecular spectroscopic database. *Journal of Quantitative Spectroscopy & RadiativeTransfer* **2009,** *110*, 533-572.

(4) Jordan, A.; Haidacher, S.; Hanel, G.; Hartungen, E.; Märk, L.; Seehauser, H.; Schottkowsky, R.; Sulzer, P.; Märk, T. D. A high resolution and high sensitivity proton-transfer-reaction time-of-flight mass spectrometer (PTR-TOF-MS). *International Journal of Mass Spectrometry* **2009,** *286*, 122-128.

(5) Müller, M.; Eichler, P.; D’Anna, B.; Tan, W.; Wisthaler, A. Direct Sampling and Analysis of Atmospheric Particulate Organic Matter by Proton-Transfer-Reaction Mass Spectrometry. *Anal. Chem.* **2017,** *89*, 10889-10897.

(6) Mikoviny, T.; Kaser, L.; Wisthaler, A. Development and characterization of a High-Temperature Proton-Transfer-Reaction Mass Spectrometer (HT-PTR-MS). *Atmos. Meas. Tech.* **2010,** *3*, 537-544.

(7) Karl, M.; Dye, C.; Schmidbauer, N.; Wisthaler, A.; Mikoviny, T.; D'Anna, B.; Müller, M.; Borrás, E.; Clemente, E.; Muñoz, A., et al. Study of OH-initiated degradation of 2-aminoethanol. *Atmos. Chem. Phys.* **2012,** *12*, 1881-1901.

(8) Eichler, P.; Muller, M.; D'Anna, B.; Wisthaler, A. A novel inlet system for online chemical analysis of semi-volatile submicron particulate matter. *Atmospheric Measurement Techniques* **2015,** *8*, 1353-1360.

(9) Eichler, P.; Müller, M.; Rohmann, C.; Stengel, B.; Orasche, J. r.; Zimmermann, R.; Wisthaler, A. Lubricating oil as a major constituent of ship exhaust particles. *Environ. Sci. Technol. Lett.* **2017,** *4*, 54-58.

(10) Drewnick, F.; Hings, S. S.; DeCarlo, P.; Jayne, J. T.; Gonin, M.; Fuhrer, K.; Weimer, S.; Jimenez, J. L.; Demerjian, K. L.; Borrmann, S., et al. A New Time-of-Flight Aerosol Mass Spectrometer (TOF-AMS)—Instrument Description and First Field Deployment. *Aerosol Science and Technology* **2005,** *39*, 637-658.

(11) Müller, M.; George, C.; D'Anna, B. Enhanced spectral analysis of C-TOF Aerosol Mass Spectrometer data: Iterative residual analysis and cumulative peak fitting. *International Journal of Mass Spectrometry* **2011,** *306*, 1-8.

(12) Farren, N. J.; Ramírez, N.; Lee, J. D.; Finessi, E.; Lewis, A. C.; Hamilton, J. F. Estimated Exposure Risks from Carcinogenic Nitrosamines in Urban Airborne Particulate Matter. *Environ. Sci. Technol.* **2015,** *49*, 9648-9656.

(13) Jonsson, Å. M.; Hallquist, M.; Saathoff, H. Volatility of secondary organic aerosols from the ozone initiated oxidation of α-pinene and limonene. *J. Aerosol Sci* **2007,** *38*, 843-852.

(14) Salo, K.; Jonsson, Å. M.; Andersson, P. U.; Hallquist, M. Aerosol Volatility and Enthalpy of Sublimation of Carboxylic Acids. *J. Chem. Phys. A* **2010,** *114*, 4586-4594.

(15) Salo, K.; Westerlund, J.; Andersson, P. U.; Nielsen, C.; D'Anna, B.; Hallquist, M. Thermal Characterization of Aminium Nitrate Nanoparticles. *J. Phys. Chem. A* **2011,** *115*, 11671-11677.

(16) Antonsen, S.; Aursnes, M.; Gallantree-Smith, H.; Dye, C.; Stenstroem, Y. Safe synthesis of alkylhydroxy and alkylamino nitramines. *Molecules* **2016,** *21*, 1738/1-1738/8.

(17) Gever, G.; O'Keefe, C.; Drake, G.; Ebetino, F.; Michels, J.; Hayes, K. Chemotherapeutic Nitrofurans. I. Some Derivatives of 3-Amino-2-oxazolidone1. *Journal of the American Chemical Society* **1955,** *77*, 2277-2281.

(18) Curtiss, L. A.; Redfern, P. C.; Raghavachari, K. Gaussian-4 theory. *J. Chem. Phys.* **2007,** *126*, 084108.

(19) Lazarou, Y. G.; Kambanis, K. G.; Papagiannakopoulos, P. Gas-Phase Reactions of $(CH_3)_2N$ Radicals with NO and NO_2. *Journal of Physical Chemistry* **1994,** *98*, 2110-2115.

(20) Lindley, C. R. C.; Calvert, J. G.; Shaw, J. H. Rate Studies of the Reactions of the $(CH_3)_2N$ Radical with O_2, NO, and NO_2. *Chemical Physics Letters* **1979,** *67*, 57-62.

(21) Atkinson, R.; Baulch, D. L.; Cox, R. A.; Crowley, J. N.; Hampson, R. F.; Hynes, R. G.; Jenkin, M. E.; Rossi, M. J.; Troe, J. Evaluated kinetic and photochemical data for atmospheric chemistry: Volume I - gas phase reactions of O(x), HO(x), NO(x) and SO(x) species. *Atmos. Chem. Phys.* **2004,** *4*, 1461-1738.

(22) Tang, Y.; Hanrath, M.; Nielsen, C. J. Do primary nitrosamines form and exist in the gas phase? A computational study of CH_3NHNO and $(CH_3)_2NNO$. *Phys. Chem. Chem. Phys.* **2012,** *14*, 16365-16370.

(23) Tang, Y.; Nielsen, C. J. Theoretical Study on the Formation and Photolysis of Nitrosamines (CH_3CH_2NHNO and $(CH_3CH_2)_2NNO$) under Atmospheric Conditions. *J. Chem. Phys. A* **2013,** *117*, 126-132.

(24) da Silva, G. Formation of Nitrosamines and Alkyldiazohydroxides in the Gas Phase: The CH_3NH + NO Reaction Revisited. *Environ. Sci. Technol.* **2013,** *47*, 7766-7772.

(25) Tan, W.; Zhu, L.; Mikoviny, T. s.; Nielsen, C. J.; Wisthaler, A.; Eichler, P.; Müller, M.; D'Anna, B.; Farren, N. J.; Hamilton, J. F. Theoretical and Experimental Study on the Reaction of tert-Butylamine with OH Radicals in the Atmosphere. *J. Chem. Phys. A* **2018,** *122*, 4470-4480.

(26) Georgievskii, Y.; Klippenstein, S. J. Long-range transition state theory. *J. Chem. Phys.* **2005,** *122*, 194103.

(27) Atkinson, R.; Baulch, D. L.; Cox, R. A.; Crowley, J. N.; Hampson, R. F.; Hynes, R. G.; Jenkin, M. E.; Rossi, M. J.; Troe, J. Evaluated kinetic and photochemical data for atmospheric chemistry: Volume II - gas phase reactions of organic species. *Atmos. Chem. Phys.* **2006,** *6*, 3625-4055.

(28) Sehested, J.; Nielsen, O. J.; Wallington, T. J. Absolute rate constants for the reaction of NO with a series of peroxy radicals in the gas phase at 295 K. *Chemical Physics Letters* **1993,** *213*, 457-464.

(29) Langer, S.; Ljungström, E.; Ellermann, T.; Nielsen, O. J.; Sehested, J. Pulse radiolysis study of reactions of alkyl and alkylperoxy radicals originating from methyl tert-butyl ether in the gas phase. *Chemical Physics Letters* **1995,** *240*, 499-505.

(30) Peeters, J.; Vertommen, J.; Langhans, I. Rate Constants of the Reactions of CF3O2, i-C3H7O2 and t-C4H9O2 with NO. *Berichte der Bunsengesellschaft für physikalische Chemie* **1992,** *96*, 431-436.
